# Synthesis of unnatural α-amino esters using ethyl nitroacetate and condensation or cycloaddition reactions

**DOI:** 10.3762/bjoc.14.263

**Published:** 2018-11-15

**Authors:** Glwadys Gagnot, Vincent Hervin, Eloi P Coutant, Sarah Desmons, Racha Baatallah, Victor Monnot, Yves L Janin

**Affiliations:** 1 Unité de Chimie et Biocatalyse, Département de Biologie Structurale et Chimie, Institut Pasteur, 28 rue du Dr. Roux, 75724 Paris Cedex 15, France; 2 Unité Mixte de Recherche 3523, Centre National de la Recherche Scientifique, 28 rue du Dr. Roux, 75724 Paris Cedex 15, France; 3 Université Paris Descartes, Sorbonne Paris Cité, 12 rue de l'École de Médecine, 75006 Paris, France

**Keywords:** α-amino ester, α-nitro esters, cerium ammonium nitrate, cycloaddition, gold(I) cyclization

## Abstract

We report here on the use of ethyl nitroacetate as a glycine template to produce α-amino esters. This started with a study of its condensation with various arylacetals to give ethyl 3-aryl-2-nitroacrylates followed by a reduction (NaBH_4_ and then zinc/HCl) into α-amino esters. The scope of this method was explored as well as an alternative with arylacylals instead. We also focused on various [2 + 3] cycloadditions, one leading to a spiroacetal, which led to the undesired ethyl 5-(benzamidomethyl)isoxazole-3-carboxylate. The addition of ethyl nitroacetate on a 5-methylene-4,5-dihydrooxazole using cerium(IV) ammonium nitrate was also explored and the synthesis of other oxazole-bearing α-amino esters was achieved using gold(I) chemistry.

## Introduction

**Scheme 1:**
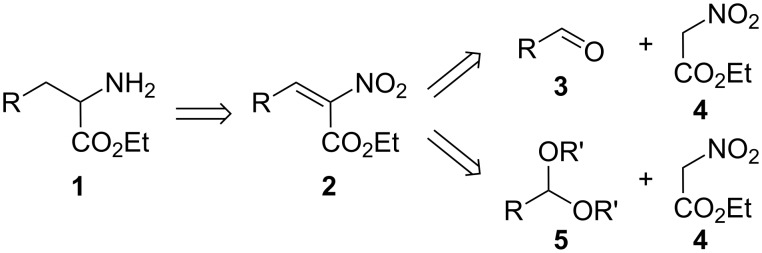
α-Amino esters from ethyl nitroacetate (**4**).

In the course of our work on an original synthesis of imidazo[1,2-*a*]pyrazin-3(7*H*)-one luciferins [1], a large variety of racemic α-amino esters was required to prepare a corresponding array of analogues. As we reviewed recently, nitroacetates are amongst the principal glycine templates used to prepare α-amino esters **1** [2]. The retrosynthetic pathway depicted in Scheme 1 requires a reduction of ethyl nitroacrylates **2**, which are made from condensation reactions between aldehydes **3** or acetals **5** and ethyl nitroacetate (**4**). However, the high yield condensations with aldehydes **3** which were reported for some examples [3–4] require more than stoichiometric amounts of titanium tetrachloride thus leading to considerable amounts of metal-containing chemical waste. Moreover, far lower yields were reported in many other instances when using this reagent [5–6]. In an attempt to improve the generality of this synthetic pathway and diminish its requirement for metals, we studied some alternatives, such as the condensations between ethyl nitroacetate (**4**) and acetals **5** or other approaches further described in the following.

## Results and Discussion

As depicted in Table 1, we first studied the scope of the condensation between ethyl nitroacetate (**4**) and aryldimethylacetals **5a–s** which are easily obtained in situ from the corresponding arylaldehydes **3a**–**s**. As seen by ^1^H NMR monitoring, the treatment of arylaldehydes **3a**–**s** with trimethyl orthoformate and an acid-bearing resin in dry methanol led to full conversion into the corresponding acetals **5a**–**s**. Then, as reported [7–8], heating the crude acetals **5a**–**s** and ethyl nitroacetate (**4**) in the presence of acetic anhydride afforded a mixture of compounds containing variable amounts of the expected acrylates **2a**–**s**. No attempts were made to purify these as they were immediately subjected to a reduction of its double bond using sodium borohydride [9–10] in refluxing isopropanol in order to properly isolate the corresponding α-nitro esters **6a**–**s**. Isopropanol was used instead of ethanol in order to decrease the incidence of a recurrent side product arising from a decarboxylation or a retro condensation of the partially reduced ester function (this side product was characterized in ^1^H NMR by two triplets at 4.6 ppm and 3.4 ppm, but eluded our purification efforts). Moreover, one minute in refluxing isopropanol was found to be sufficient to complete the reduction especially for the fairly insoluble acrylate **2e**, and also avoided most of the undesired transesterification byproducts that could form upon long reaction times. As seen in Table 1, modest yields of compounds **6a**–**i** were isolated anyway, especially for *meta*-substituted phenyl-bearing products **6c**, **6f** and **6h**. Even if the reduction was less than perfect, the low yields originate from the initial condensation between acetals **5** and **4**. Indeed, when considering the phenyl derivative of **6a**, a sobering 35% isolated yield was obtained, in stark contrast to the reported 95% yield published in 1980 [7]. In our hands, ^1^H NMR analysis of the crude condensation product pointed out the occurrence of the intermediate acrylate **2a** along with a large amount of methyl benzoate, a well-known side product in this reaction [7–8]. From the aryl acetals **5g**–**l** featuring electron-withdrawing groups, low to non-detectable amounts of the condensations products **2g**–**l** were observed by ^1^H NMR analysis, and this was reflected in the isolated yield of the corresponding α-nitroesters **6g**–**i** as well as the lack of compounds **6j**–**l**. Similarly, no condensation was observed when starting with the pyridyl-bearing acetal **5m**. These disappointing results are plausibly due to two factors: (i) As mentioned above, the condensation of ethyl nitroacetate (**4**) with aryl acetals **5** takes place along with an O-alkylation reaction which leads, via a rearrangement, to the corresponding aryl ester byproduct [7–8]. The proportion between the C-alkylation (leading to the expected acrylate) and this O-alkylation is most certainly governed by the electronic effects of the substituent on the aryl group. Indeed, the best overall yields are observed when starting from the electronically similar 2-methoxy acetal **5b** or the 4-methoxy analog **5d**, as well as the 4-benzyloxy acetal **5e**. (ii) Secondly, the reduction of acrylates **2a**–**s** to compounds **6a**–**s** was achieved with sodium borohydride and the resulting basic conditions could be detrimental to the stability of some of these α-nitro acrylates. In the past, such reductions have been achieved under fairly uncommon conditions (NaBH_4_ in a mixture of isopropanol and chloroform over a large proportion of silica gel) [11–12]. However, when tried, no real overall improvements were observed with these conditions. Other series of trials were made to improve the overall yields of the furan-bearing α-nitro esters **6n**–**r**. We first tried to avoid the preparation of the acetals **5n** or **5o** and used the previously reported direct condensation between furfural (**3n**) and ethyl nitroacetate (**4**) [13]. Unfortunately, we could not reproduce the 95% yield reported for compound **2n**, and under a thoroughly inert atmosphere (as advised) we obtained 48–53% isolated yields at best. In any case, this approach did shorten the synthetic pathway by one step and upon the reduction of the resulting acrylates **2n** or **2o** using sodium borohydride, the α-nitro esters **6n** and **6o** were isolated in the rather modest yields indicated in Table 1. We then focused on the model reduction of acrylate **2n** into **6n**. Since all our attempts to achieve a palladium-catalyzed hydrogenation failed, we tried other borohydride salts. The use of tetramethylammonium borohydride did not increase the overall yield of **6n**, however, a rather substantial improvement was observed when using sodium cyanoborohydride. Indeed, from pure acrylate **2n**, a 70% yield of **6n** was obtained, and in one pot starting from aldehyde **3n**, a 48% overall yield of **6n** was achieved. Moreover, without using an inert atmosphere for the initial condensation between furfural (**3n**) and ethyl nitroacetate (**4**) the overall yield of **6n** dropped to 37% even when using sodium cyanoborohydride. The optimized conditions were then applied to aldehyde **3r** and afforded a significantly improved 46% yield of **6r**, in comparison with our single trial via **5r**, which ended up with less than 20% of an impure sample of **6r**. Finally, from the isolated α-nitro esters **6a**–**s**, their reduction into the corresponding α-amino esters **1a**–**s**, using zinc and hydrochloric acid in ethanol usually proceeded in good yield, although care had to be taken during work-up as zinc complexes required the addition of an excess of ammonia to fully break in the course of the extraction.

**Table 1:** Synthesis and reductions of α-nitro acrylates into α-amino esters.

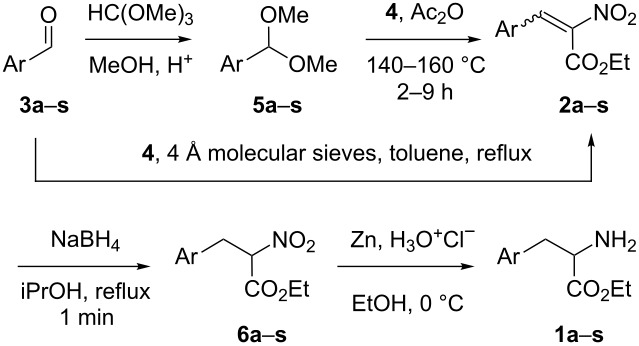				

	Ar	% **6**^a^	% **6**^b^	% **1**

**a**	C_6_H_5_	35^c^		–
**b**	2-MeOC_6_H_4_	53		90
**c**	3-MeOC_6_H_4_	21		92
**d**	4-MeOC_6_H_4_	55		95
**e**	4-BnOC_6_H_4_	51		67
**f**	3-MeC_6_H_4_	16		80
**g**	2-FC_6_H_4_	6		94
**h**	3-FC_6_H_4_	0.9		–
**i**	4-FC_6_H_4_	13		94
**j**	2-CF_3_C_6_H_4_	0^d^		–
**k**	3-CF_3_C_6_H_4_	<5^d^		–
**l**	4-CF_3_C_6_H_4_	<5^d^		–
**m**	2-pyridyl	0^d^		–
**n**	furan-2-yl	27	38/48^e^/70^f^	94
**o**	furan-3-yl	–	34	88
**p**	5-methyl-furan-2-yl	60	–	75
**q**	5-ethyl-furan-2-yl	39	–	56
**r**	4,5-dimethyl-furan-2-yl	<20	46^e^	79
**s**	thiophen-2-yl	33	–	58

^a^Isolated yield from **3a**–**s**, via acetals **5a**–**s**. ^b^Isolated yield via the direct condensation between **3a**–**s** and **4**. ^c^39% yield from (diethoxymethyl)benzene. ^d^As seen by ^1^H NMR analysis. ^e^Using NaBH_3_CN; see text. ^f^Also using NaBH_3_CN but from pure **2n**; see text.

**Scheme 2:**
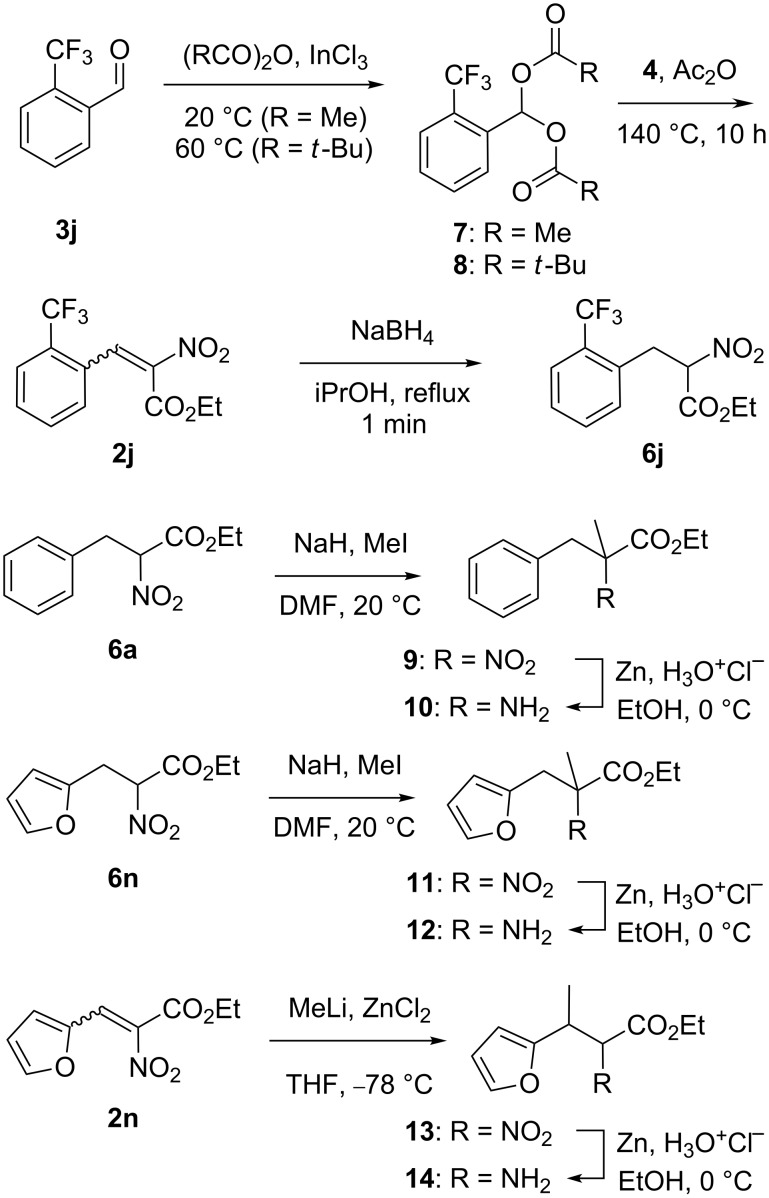
Preparations of α-amino esters **10**, **12** and **14**.

In an attempt to overcome the lack of condensation between ethyl nitroacetate (**4**) and electron-poor substrates **5j**–**l**, we focused on the model preparation of the trifluoromethyl-bearing α-nitro ester **6j** from acylals **7** depicted in Scheme 2. As well reviewed [14], acylals can be prepared from aldehydes and anhydrides using a variety of acids as catalysts. In our case, ^1^H NMR monitoring of the reaction between 2-(trifluoromethyl)benzaldehyde (**3j**) and acetic anhydride using indium(III) chloride [15] as a Lewis acid catalyst [16] without any solvent at room temperature pointed out a complete conversion into acylal **7** overnight. A similar reaction using pivaloyl anhydride and either indium(III) chloride or tetrafluoroboric acid as a catalyst had to be heated at 60 °C for a few hours to secure a similar conversion into pivalal **8**. From intermediates **7** and **8**, and as previously reported in the case of malonates [17–19], we then hoped for an improvement of their condensation with ethyl nitroacetate (**4**). The ^1^H NMR monitoring of the reaction between compounds **7** or **8** and ethyl nitroacetate (**4**) in the presence of a catalytic amount of indium(III) chloride pointed out the occurrence of tangibly more of the expected acrylate **2j,** although along with many byproducts. Indeed, in a typical experiment, upon reduction of the crude reaction product obtained from **8** and ethyl nitroacetate (**4**), a discouraging 13% yield of the corresponding α-nitro ester **6j** was isolated. The use of α-nitro esters to obtain disubstituted α-amino esters such as compound **10** via an alkylation step has been described [20]. In order to reach such α-amino esters, we tried their preparation via a C-methylation of α-nitroester **6a** in DMF using sodium hydride and methyl iodide. Upon purification, this gave 46% of the nitro compound **9** with 94% purity (as assessed by ^1^H NMR). Despite this modest yield, the ensuing reduction using zinc and hydrochloric acid in ethanol overnight gave a sufficient amount of the (pure) target phenyl-bearing α-amino ester **10** which had been previously obtained by catalytic hydrogenation using palladium [20]. The same transformation sequences were used starting with compound **6n** and provided the furan-bearing α-amino ester **12** in 32% overall yield via the nitro compound **11**. We also investigated the reported [4] 1,4-addition of a methyl on compound **2n** to prepare the β-methylated derivative **13**. In our hands, a rather modest 43% yield of the expected adduct **13** was achieved from purified acrylate **2n**. Again, the ensuing reduction of **13** using zinc and hydrochloric acid gave the target α-amino ester **14** in an 85% yield.

**Scheme 3:**
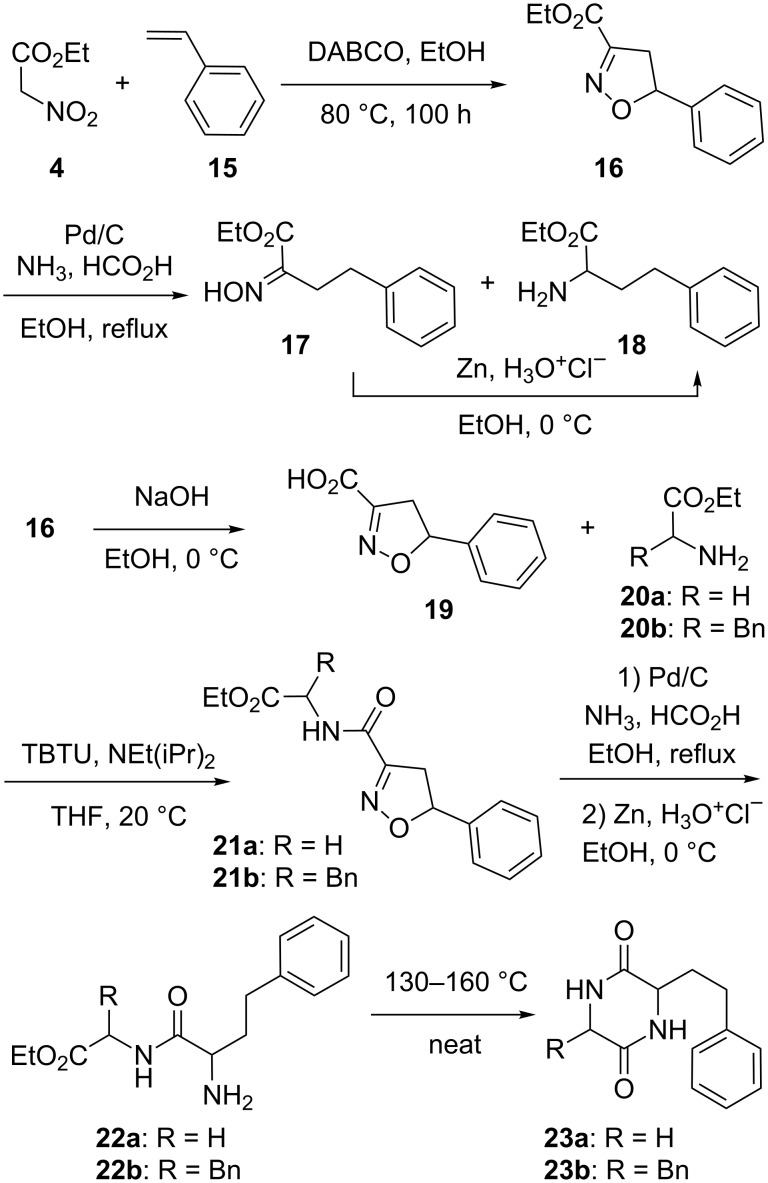
Syntheses of α-amino ester **18** and piperazinediones **23a**,**b**.

To avoid the recourse to more rare and/or expensive heterocyclic aldehydes, we also tried synthetic approaches based on [2 + 3] cycloadditions. As depicted in Scheme 3, the carbon dioxide-producing reaction [21] between two equivalents of ethyl nitroacetate (**4**) and styrene (**15**), gave the isoxazoline **16** in a 72% yield as a latent α-amino ester [22–24]. From this compound, a reductive cleavage of the isoxazoline ring was initiated using palladium over charcoal and a large excess of ammonium formate in refluxing ethanol. The analysis of the resulting mixture by LC/MS and ^1^H NMR pointed out the occurrence of the expected [25] oxime **17** but along with an unexpected sizable proportion of the α-amino ester **18**. Accordingly, the (filtrated) ethanolic solution was then treated with zinc and hydrochloric acid in ethanol to complete the reduction and the amino ester **18** was isolated in a 72% yield. In order to illustrate the synthetic potential of the isoxazoline **16** as an already protected amino acid moiety, we prepared the piperazine-2,5-diones **23a**,**b** in four steps. This was achieved by the hydrolysis of the ester function of **16**, followed by its coupling with glycine or phenylalanine ethyl esters (respectively **20a** and **20b**) using 2-(1*H*-benzotriazole-1-yl)-1,1,3,3-tetramethylaminium tetrafluoroborate (TBTU) as a coupling agent to give the corresponding amides **21a**,**b**. The isoxazole ring of these compounds was then cleaved using palladium and ammonium formate to give the corresponding oximes which were immediately reduced into amines **22a**,**b**, using zinc and hydrochloric acid, and a thermal cyclization of these crude products gave the piperazine-2,5-diones **23a**,**b** in, respectively, 31 and 40% overall yield from compound **16**.

**Scheme 4:**
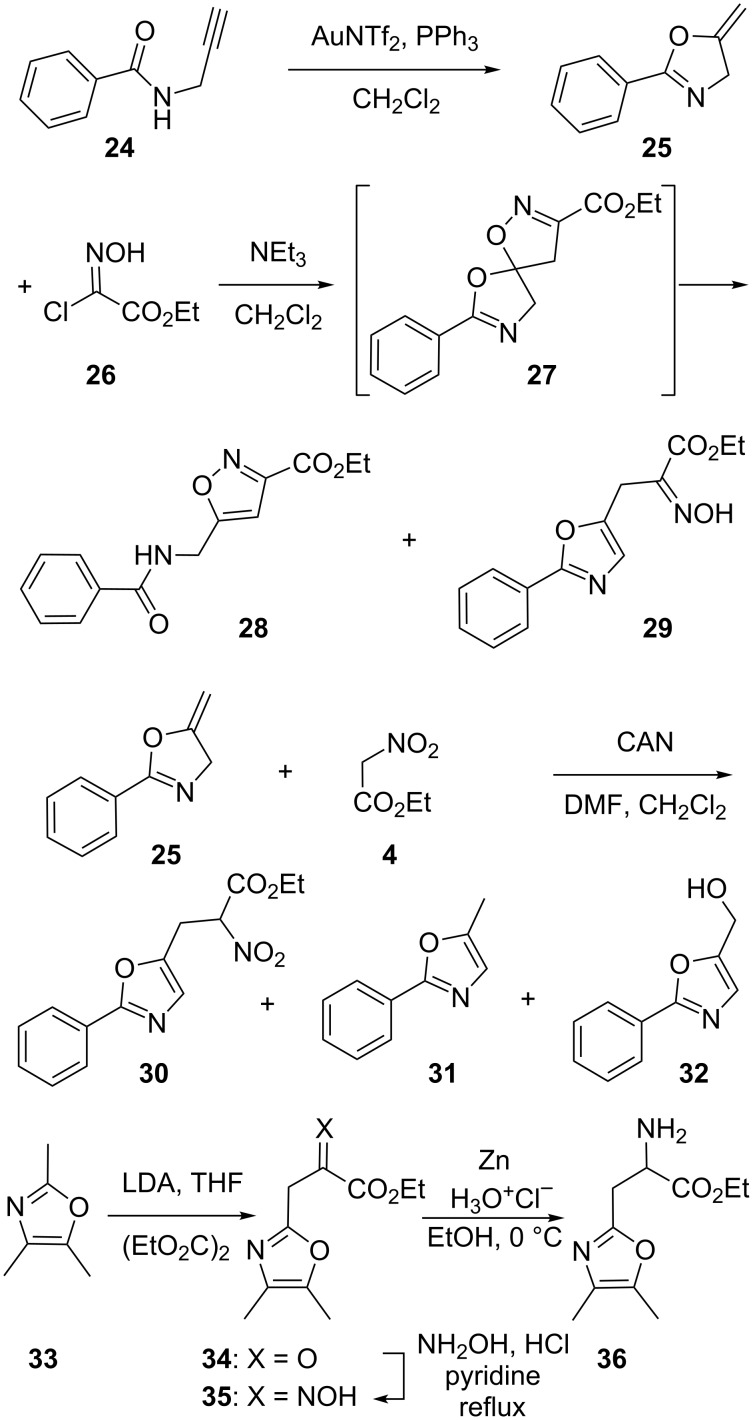
Syntheses of α-hydroximino ester **29** and α-amino ester **36**.

A second [2 + 3] cycloaddition-based approach is described in Scheme 4. It started with the preparation of the methylene-bearing dipolarophile **25** from propargylamide **24** using gold(I) chemistry, which turned out to be tolerant to a wide variety of dry solvents (dichloromethane, tetrahydrofuran, toluene, dimethylformamide, or acetonitrile) [26–27]. As for a related report [28] describing [2 + 3] cycloadditions between chlorooxime **26** and other methylene-bearing compounds, its reaction with compound **25** gave the spiroacetal **27**. However, in the present case this cycloadduct was only detectable by ^1^H NMR analysis of the crude reaction mixture. Indeed, a slow ring-opening reaction took place upon standing in solution to mainly give the isoxazole isomer **28** along with much less of the target oxazole-bearing α-hydroximino ester **29**. Extensive trials to alter the selectivity of the ring opening using heat, adsorption over silica, acids (BF_3_·OEt_2_, AcOH) or bases (NEt_3_, LDA, EtONa) all failed to change the ratio of compounds **28** and **29**, which were isolated in 5 and 28% yield, respectively. Despite the potential synthetic interest [29–30] of isoxazole **28**, we did not pursue this further, but focused on another approach involving an oxidative addition of ethyl nitroacetate (**4**) on the methylene-bearing dipolarophiles **25** mediated by cerium(IV) ammonium nitrate (CAN). This was inspired by reports describing CAN-mediated carbon–carbon bond formation reactions between ethyl nitroacetate (**4**) and tri-*O*-acetylglycals [31–32] or other sugar-derived alkenes [33]. We first tried the conditions described in the literature (0 °C, mixture of methanol and dimethylformamide as a solvent), without much success in our case. Quite a few trials followed, changing the solvent to a dichloromethane/dimethylformamide mixture or dimethylformamide alone, or modifying the reaction conditions (at room temperature, 0 °C or −20 °C), but none resulted in a flagrant improvement. Indeed, as precisely described in the experimental part, the (impure) target nitroester **30** was isolated once in a disappointing 22% yield and quite a few byproducts were noticed. A control experiment omitting the ethyl nitroacetate (**4**) allowed us to identify amongst these: the oxazole derivative **31** resulting from an isomerization of **25** as well as alcohol **32** resulting from an oxidation of compound **25**. Accordingly, this greatly dampened our hope to improve this transformation (trials with manganese(III) acetate were not successful either). We then resorted to a different approach to prepare the oxazole-bearing α-amino ester **36** from 2,4,5-trimethyloxazole (**33**). Deprotonation of this compound was achieved using lithium diisopropylamide (LDA) and this was followed by the addition of diethyl oxalate to give a mixture of compounds including the ketoester **34**. Then, treatment of this mixture with hydroxylamine allowed the isolation of the target α-hydroximino ester **35** in a 7% overall yield. Two dimensional NMR experiments confirmed the depicted structure for compound **35** and trace amounts of the other isomers were detected in other chromatographic fractions but could not be fully purified. In any case, from the α-hydroximino ester **35**, a reduction using zinc and hydrochloric acid gave the target oxazole-bearing α-amino ester **36**.

**Scheme 5:**
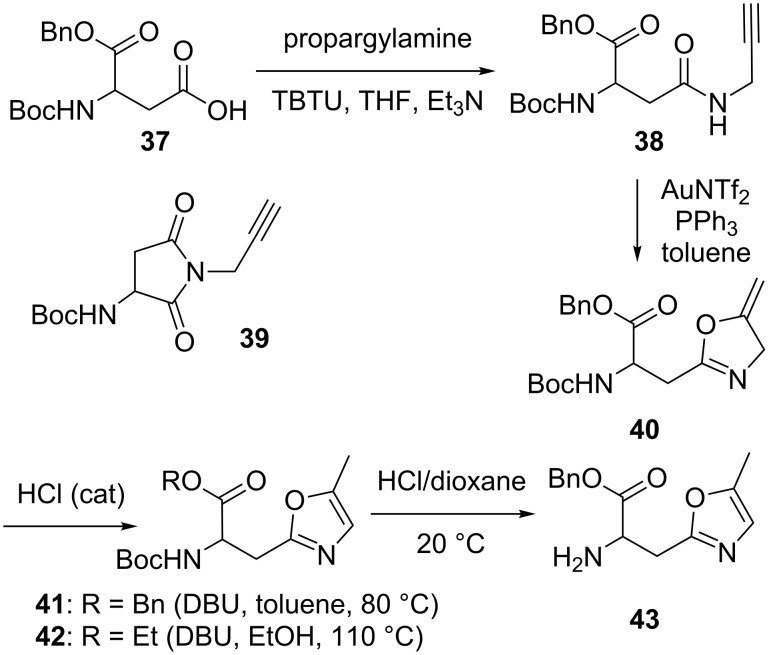
Synthesis of α-amino ester **43**.

Finally, as depicted in Scheme 5, the oxazole-bearing α-amino ester **43** was prepared from the aspartic acid derivative **37** through the propargylamide **38** followed by a gold(I)-catalyzed cyclization to form the oxazoline derivative **40**. Concerning the amidation step, propargylamide **38** was obtained in 78% yield provided that an excess of triethylamine was avoided (otherwise, as determined by a control experiment, substantial amounts of the relatively stable succinyl derivative **39** [34] resulting from a triethylamine-triggered cyclization of **38** were isolated) [35–37]. The treatment of compound **38** with a catalytic amount of gold(I) in warm toluene provided us with the oxazoline **40** in an 80% yield. However, this compound turned out to be unstable, either on standing, probably because of an autoxidation, as reported in other instances [27], or in CDCl_3_, probably because of acid traces. To achieve its isomerization, a literature search pointed out the use of an excess of DBU and heat [38–39]. However, boiling compound **40** in toluene in the presence of an excess of DBU led, after chromatography, to only 24% of the benzyl ester **41**. Since, amongst few side reactions, we suspected a benzylester cleavage, we undertook this reaction under argon in ethanol at 110 °C using a microwave reactor along with only one equivalent of DBU and these changes provided us with the ethyl ester **42** in a 51% yield. Finally, a far more simple procedure was found by just adding a catalytic amount of hydrogen chloride in 1,4-dioxane to the toluene solution containing compound **40**. This afforded, after overnight stirring, the isomerized compound **41** in a 69% yield. Finally, the deprotection of the amine function was achieved with the use of an excess of hydrogen chloride in 1,4-dioxane to give the target α-amino ester **43**.

## Conclusion

In the course of our attempts to extend the use of ethyl nitroacetate (**4**) to prepare α-amino ethyl esters via condensation reactions with aldehydes **3** or dimethylacetals **5**, some severe limitations were encountered. Indeed, the ubiquitous occurrence of aryl methyl esters, arising from an unwanted O-alkylation of ethyl nitroacetate (**4**), plagued all our efforts to improve the latter synthetic pathway [2]. This side reaction pretty much limited the approach to electron-rich substrates and even our attempts to use the acylals **7** or **8**, easily made from 2-trifluoromethylbenzaldehyde (**3j**), were very moderately successful. Such phenomenon probably accounts for the modest yields reported in many instances even when using titanium tetrachloride to achieve this condensation [5–6]. Concerning the reduction of the nitroacrylates **2** into the α-nitro esters **6**, tangible but still modest yield improvements were observed when using sodium cyanoborohydride instead of sodium borohydride in some cases. This actually illustrates the sensitivity of this reduction which, along with the condensation, are quite limiting. As described above, the recourse to cycloaddition-based approaches allowed us to explore some original chemistry aiming at the preparation of oxazole-bearing α-amino esters which was of interest per se. Indeed, the previously unreported acid-catalyzed conditions to achieve the isomerization of the methylene-bearing oxazoline **40** into oxazole **41** should be useful in many other instances. In any case, as described in a following report [40], to overcome some of the limitations described here, we then focused on an exhaustive investigation of malonate-based strategies and reached an even more diverse set of α-amino esters.

## Supporting Information

File 1 Experimental and copies of spectra.
