# Supplementary material for: Synthesis of unnatural α-amino esters using ethyl nitroacetate and condensation or cycloaddition reactions
Source: Beilstein J Org Chem. 2018 Nov 15;14:2846–52. doi: 10.3762/bjoc.14.263 (PMC6244114; doi:10.3762/bjoc.14.263)
Supplement: File 1 — Experimental and copies of spectra. [file Beilstein_J_Org_Chem-14-2846-s001.pdf]

BEILSTEIN JOURNAL OF ORGANIC CHEMISTRY

Supporting Information

for

Synthesis of unnatural α-amino esters using ethyl nitroacetate and condensation or cycloaddition reactions

Glwadys Gagnot, Vincent Hervin, Eloi P. Coutant, Sarah Desmons, Racha Baatallah, Victor Monnot and Yves L. Janin

Beilstein J. Org. Chem. **2018,** *14,* 2846–2852. doi:10.3762/bjoc.14.263

Experimental and copies of spectra

License and Terms: This is a supporting information file under the terms of the Creative Commons Attribution License (http://creativecommons.org/licenses/by/4.0). Please note that the reuse, redistribution and reproduction in particular requires that the authors and source are credited.
The license is subject to the *Beilstein Journal of Organic Chemistry* terms and conditions: (https://www.beilstein-journals.org/bjoc)

Experimental

The ^{1}H NMR and ^{13}C NMR spectra were recorded on a Bruker Avance 400 spectrometer at 400 MHz and 100 MHz, respectively. Chemical shifts (δ) are given in ppm with respect to TMS and cross-coupling constants (*J*) are given in hertz. Column chromatography was performed either on Merck silica gel 60 (0.035–0.070 mm) or neutral alumina using a solvent pump and an automated collecting system driven by a UV detector set to 254 nm unless required otherwise. Sample deposition was carried out by absorption of the mixture to be purified on a small amount of the solid phase followed by its deposition on the top of the column. The low resolution mass spectra were obtained on an Agilent 1200 series LC/MSD system using an Agilent Jet-Stream atmospheric electrospray ionization system and the high resolution mass spectra (HRMS) were obtained using a Waters Micromass Q-TOF with an electrospray ion source.

Preparation of ethyl α-nitroesters 6 via dimethylacetals 5 or directly from aldehydes 3, general methods: Step i: preparation of dimethylacetals **5**. The corresponding aldehyde **3** (0.044 mol) and trimethyl orthoformate (5.8 mL, 0.053 mol) were dissolved in methanol (7.3 mL, 0.24 mol, dried over 3 Å MS). To this was added DOWEX 50WX8-100 ion-exchange resin (0.2 g) and the solution was stirred overnight under a calcium chloride-protected atmosphere. The resin was then removed by filtration, washed with dry methanol and the filtrate concentrated to dryness (no lower than 30 mbar) to yield the corresponding and often volatile acetals **5** (usually not fully stable in $CDCl_3$). Step iii: condensation with ethyl nitroacetate (**4**). In order to remove eventual traces of water, prior to this reaction, ethyl nitroacetate (5.7 g, 0.042 mol) was stirred in acetic anhydride (5 mL, 0.053 mol) for 15 minutes under a calcium chloride-protected atmosphere. To this solution was added the crude acetal **5** dissolved in acetic anhydride (5.1 mL, 0.053 mol) and the solution was heated at the temperatures and times indicated below while allowing the resulting lower boiling methyl acetate to distil off. This was then thoroughly concentrated to dryness to yield the crude 3-aryl-2-nitroacrylates **2**. Step iv: reduction with sodium borohydride. The crude acrylate was dispersed/dissolved in isopropanol (100 mL, dried over 4 Å MS). To this was added portion-wise sodium borohydride (2 g, 0.053 mol) and the suspension was heated to reflux for a maximum of one minute before allowing it to cool back to room temperature. Acetic acid was then cautiously added (3.3 mL, 0.053 mol) and the isopropanol was removed under vacuum. The crude residue was dispersed in water (100 mL) and 10% hydrochloric acid (5.7 mL, 0.057 mol) was added to help the hydrolysis of half-reacted boron hydrides. The resulting solution was extracted with ethyl acetate, the organic layer was washed with water, brine, dried over magnesium sulfate and concentrated to dryness. The residue was then purified as described in each case below to yield the corresponding α-nitroesters **6**.

Alternative for the preparation of furan-bearing 2-nitroacrylates **2** via step ii from aldehydes **3** and ethyl nitroacetate (**4**): As based on the described procedure [1], 4 Å MS (32 g) was dispersed in

toluene (120 mL) and this was extensively degassed by bubbling a stream of argon into the solution. The considered furan-bearing aldehydes (0.0416 mol) and ethyl nitroacetate (5.54 g, 0.0416 mol) were added followed by piperidine (0.35 g, 0.0041 mol) and this was heated to reflux under an inert atmosphere for the times described below. The suspension was cooled, filtered, the solid washed with toluene and concentrated to dryness (see below for the specific purification of compound **2n**) before undertaking a reduction using sodium borohydride in refluxing isopropanol as described above. Alternatively for the reduction step, the suspension was cooled, filtered and the solid washed with dry ethanol (150 mL). To this solution, sodium cyanoborohydride (2.51 g, 0.0416 mol) was added and this was stirred overnight under a calcium chloride-protected atmosphere. This was diluted in water made acid with 1 N hydrochloric acid (50 mL) and extracted with ethyl acetate. The organic layer was washed with water brine, dried over magnesium sulfate, concentrated to dryness and the residue purified as described below. In the specific case of compound **2n**, a chromatography over silica gel (cyclohexane/dichloromethane 1:1) led to the isolation of the pure compound as colorless oil (which slowly darkened over weeks) containing a 2:3 mixture of isomers (11.7 g, 53%). Ethyl 3-(furan-2-yl)-2-nitroacrylate (**2n**): ^{1}H NMR ($CDCl_3$, minor isomer): 7.86 (s, 1H), 7.66 (d, 1H, *J* = 1.8 Hz), 7.07 (d, 1H, *J* = 3.7 Hz), 6.63 (dd, 1H, *J* = 1.8, 3.7 Hz), 4.49 (q, 2H, *J* = 7.2 Hz), 1.42 (t, 3H, *J* = 7.2 Hz). ^{1}H NMR ($CDCl_3$, major isomer): 7.63 (d, 1H, *J* = 1.8 Hz), 7.36 (s, 1H), 6.93 (d, 1H, *J* = 3.7 Hz), 6.57 (dd, 1H, *J* = 1.8, 3.7 Hz), 4.37 (q, 2H, *J* = 7.2 Hz), 1.36 (t, 3H, *J* = 7.2 Hz).

Ethyl 2-nitro-3-phenylpropanoate (6a): Obtained as an oil (3.18 g, 35% from (dimethoxymethyl)benzene) after heating at 160 °C for 9 hours in the course of the condensation step and a chromatography over silica gel (cyclohexane – ethyl acetate 95/5). ^{1}H NMR ($CDCl_3$): 7.33 (m, 3H), 7.23 (m, 2H), 5.35 (dd, 1H, *J* = 5.9, 9.4 Hz), 4.30 (m, 2H), 3.58 (dd, 1H, *J* = 9.4, 14.6 Hz), 3.50 (dd, 1H, *J* = 5.9, 14.6 Hz), 1.29 (t, 3H, *J* = 7.1 Hz). ^{13}C NMR ($CDCl_3$): 164.0, 134.1, 129.0, 128.9, 127.8, 89.2, 63.1, 36.3, 13.8. Similar to reported data [2,3].

Ethyl 3-(2-methoxyphenyl)-2-nitropropanoate (6b): Obtained as an oil (6.12 g, 53% from 2-methoxybenzaldehyde) after heating at 150 °C for 9 hours in the course of the condensation step and a chromatography over silica gel (cyclohexane – ethyl acetate 95/5 to 9/1). ^{1}H NMR ($CDCl_3$): 7.28 (m, 1H), 7.14 (m, 1H), 6.91 (m, 2H), 5.57 (dd, 1H, *J* = 6.2, 9.1 Hz), 4.28 (q, 2H, *J* = 7.1 Hz), 3.82 (s, 3H), 3.55 (dd, 1H, *J* = 6.2, 14.1 Hz), 3.51 (dd, 1H, *J* = 9.1, 14.1 Hz), 1.28 (t, 3H, *J* = 7.1 Hz). ^{13}C NMR ($CDCl_3$): 164.5, 157.4, 131.3, 129.3, 122.2, 120.8, 110.3, 87.1, 62.8, 55.3, 32.3, 13.8. HRMS (*m/z*): $[M+Na]^+$ calcd for $C_{12}H_{15}NO_5Na$: 276.0848, found, 276.0831.

Ethyl 3-(3-methoxyphenyl)-2-nitropropanoate (6c): Obtained as an oil (2.34 g, 21% from 3-methoxybenzaldehyde) after heating at 150 °C for 9 hours in the course of the condensation step and a chromatography over silica gel (cyclohexane – ethyl acetate 95/5 to 9/1). ^{1}H NMR ($CDCl_3$): 7.25 (t, 1H, *J* = 7.9 Hz), 6.81 (m, 3H), 5.35 (dd, 1H, *J* = 5.8, 9.3 Hz), 4.31 (q, 2H, *J* = 7.2 Hz), 3.81 (s, 3H), 3.56 (dd, 1H, *J* = 9.3, 14.7 Hz), 3.47 (dd, 1H, *J* = 5.8, 14.7 Hz), 1.31 (t, 3H, *J* = 7.2 Hz). ^{13}C

NMR ($CDCl_3$): 164.5, 157.4, 131.3, 129.3, 122.2, 120.8, 110.3, 87.1, 62.8, 55.3, 32.3, 13.8. HRMS (*m/z*): $[M+Na]^+$ calcd for $C_{12}H_{15}NO_5Na$: 276.0848, found, 276.0831.

Ethyl 3-(4-methoxyphenyl)-2-nitropropanoate (6d): Obtained as an oil (6.95 g, 55% from 4-methoxybenzaldehyde) after heating at 150 °C for 9 hours in the course of the condensation step and a chromatography over silica gel (cyclohexane – ethyl acetate 95/5 to 9/1). ^{1}H NMR ($CDCl_3$): 7.15 (m, 2H), 6.86 (m, 2H), 5.31 (dd, 1H, *J* = 5.9, 9.5 Hz), 4.30 (m, 2H), 3.81 (s, 3H), 3.52 (dd, 1H, *J* = 9.5, 14.6 Hz), 3.44 (dd, 1H, *J* = 5.9, 14.6 Hz), 1.31 (t, 3H, *J* = 7.1 Hz). ^{13}C NMR ($CDCl_3$): 164.1, 159.2, 130.0, 126.0, 114.4, 89.4, 63.1, 55.2, 35.5, 13.9. HRMS (*m/z*): $[M+Na]^+$ calcd for $C_{12}H_{15}NO_5Na$: 276.0848, found, 276.0840.

Ethyl 3-(4-(benzyloxy)phenyl)-2-nitropropanoate (6e): Obtained as an yellow solid (4.08 g, 51% from 4-benzyloxybenzaldehyde) after heating at 150 °C for 9 hours in the course of the condensation step and a chromatography over silica gel (cyclohexane – ethyl acetate 92/8 to 9/1). ^{1}H NMR ($CDCl_3$): 7.46-7.33 (m, 5H), 7.15 (m, 2H), 6.94 (m, 2H), 5.31 (dd, 1H, *J* = 6.0, 9.3 Hz), 5.06 (s, 2H), 4.30 (m, 2H), 3.52 (dd, 1H, *J* = 9.3, 14.6 Hz), 3.44 (dd, 1H, *J* = 6.0, 14.6 Hz), 1.30 (t, 3H, *J* = 7.1 Hz). ^{13}C NMR ($CDCl_3$): 164.1, 158.4, 136.8, 130.0, 128.6, 128.4, 128.0, 127.4, 126.3, 115.3, 89.4, 70.1, 63.1, 35.5, 13.9. HRMS (*m/z*): $[M+Na]^+$ calcd for $C_{18}H_{19}NO_5Na$: 352.1161, found, 352.1150.

Ethyl 2-nitro-3-*m*-tolylpropanoate (6f): Obtained as an oil (1.05 g, 16% from 3-methylbenzaldehyde) after heating at 160 °C for 8 hours in the course of the condensation step and two chromatography over silica gel (cyclohexane – dichloromethane 2/1 to 1/6) and (cyclohexane – ethyl acetate 97/3). ^{1}H NMR ($CDCl_3$): 7.22 (m, 1H), 7.60 (m, 1H), 7.01 (m, 2H), 5.33 (dd, 1H, *J* = 5.8, 9.4 Hz), 4.30 (q, 2H, *J* = 7.1 Hz), 3.54 (dd, 1H, *J* = 9.4, 14.6 Hz), 3.46 (dd, 1H, *J* = 5.8, 14.6 Hz), 2.34 (s, 3H), 1.30 (t, 3H, *J* = 7.1 Hz). ^{13}C NMR ($CDCl_3$): 164.1, 138.7, 130.0, 129.6, 128.8, 128.5, 125.8, 89.2, 63.1, 36.2, 21.3, 13.8. HRMS (*m/z*): $[M-H]^-$ calcd for $C_{12}H_{14}NO_4$: 236.0923, found, 236.0983.

Ethyl 3-(2-fluorophenyl)-2-nitropropanoate (6g): Obtained as an oil (0.73 g, 6% from 2-fluorobenzaldehyde) after heating at 190 °C for 8 hours in the course of the condensation step and two chromatography over silica gel (cyclohexane – ethyl acetate 95/5) and (cyclohexane – dichloromethane 2/1). ^{1}H NMR ($CDCl_3$): 7.30 (m, 1H), 7.21 (m, 1H), 7.10 (m, 2H), 5.45 (dd, 1H, *J* = 5.6, 8.9 Hz), 4.31 (q, 2H, *J* = 7.2 Hz), 3.58 (m, 2H), 1.31 (t, 3H, *J* = 7.2 Hz). ^{13}C NMR ($CDCl_3$): 163.9, 161.2 (246 Hz), 131.5 (4 Hz), 129.9 (8 Hz), 124.6 (4 Hz), 121.1 (15 Hz), 115.6 (21 Hz), 87.3, 63.2, 30.4 (2 Hz), 13.8. HRMS (*m/z*): $[M-H]^-$ calcd for $C_{11}H_{11}FNO_4$: 240.0672, found, 240. 0675.

Ethyl 3-(3-fluorophenyl)-2-nitropropanoate (6h): Obtained as an oil (0.11 g, 0.9% from 3-fluorobenzaldehyde) after heating at 180 °C for 8 hours in the course of the condensation step and two chromatography over silica gel (cyclohexane – ethyl acetate 95/5) and (cyclohexane – dichloromethane from 2/1 to 1/1). Note: compound **6h** is pretty much invisible upon UV detection on TLC. ^{1}H NMR ($CDCl_3$): 7.32 (m, 1H), 6.98 (m, 3H), 5.34 (dd, 1H, *J* = 5.8, 9.4 Hz), 4.32 (m, 2H), 3.58

(dd, 1H, *J* = 9.3, 14.6 Hz), 3.50 (dd, 1H, *J* = 5.8, 14.6 Hz), 1.31 (t, 3H, *J* = 7.2 Hz). ^{13}C NMR ($CDCl_3$): 163.8, 162.9 (248 Hz), 136.5 (7 Hz), 130.6 (9 Hz), 124.6 (3 Hz), 116.0 (21 Hz), 114.9 (21 Hz), 88.8, 63.3, 35.9, 13.8. HRMS (*m/z*): $[M-H]^-$ calcd for $C_{11}H_{11}FNO_4$: 240.0672, found, 240. 0675.

Ethyl 3-(4-fluorophenyl)-2-nitropropanoate (6i): Obtained as an oil (2.72 g, 13% from 4-fluorobenzaldehyde) after heating at 150 °C for 4 hours and then 160 °C for another 4 hours in the course of the condensation step and two chromatography over silica gel (cyclohexane – ethyl acetate 9/1) and (cyclohexane – dichloromethane 2/1). ^{1}H NMR ($CDCl_3$): 7.21 (m, 2H), 7.02 (m, 2H), 5.32 (dd, 1H, *J* = 5.7, 9.4 Hz), 4.30 (m, 2H), 3.56 (dd, 1H, *J* = 9.4, 14.7 Hz), 3.47 (dd, 1H, *J* = 5.7, 14.7 Hz), 1.31 (m, 3H). ^{13}C NMR ($CDCl_3$): 163.8, 162.3 (247 Hz), 130.6 (8 Hz), 129.8 (3 Hz), 115.9 (21 Hz), 89.1, 63.2, 35.5, 13.8. HRMS (*m/z*): $[M-H]^-$ calcd for $C_{11}H_{11}FNO_4$: 240.0672, found, 240.0645.

Ethyl 3-(furan-2-yl)-2-nitropropanoate (6n): Obtained as an oil either via step ii (3.76 g, 38% from furfural) after heating at reflux for 3 hours in the course of the condensation step and a chromatography over silica gel (dichloromethane – methanol 99/1) or via step iii (6.03 g, 27%) after heating at 140 °C for 3h30 in the course of the condensation step and two chromatography over silica gel (cyclohexane ethyl acetate 95:5) and (cyclohexane – dichloromethane 3/2 to 1/1) or via step ii (4.26 g, 48% from furfural) after heating at reflux for 5h30 in the course of the condensation step, a reduction using sodium cyanoborohydride and a chromatography over silica gel (cyclohexane – dichloromethane 3/2). ^{1}H NMR ($CDCl_3$): 7.36 (m, 1H), 6.32 (m, 1H), 6.19 (m, 1H), 5.44 (dd, 1H, *J* = 5.5, 9.3 Hz), 4.32 (q, 2H, *J* = 7.2 Hz), 3.66 (dd, 1H, *J* = 9.3, 15.7 Hz), 3.54 (dd, 1H, *J* = 5.5, 15.7 Hz), 1.32 (t, 3H, *J* = 7.2 Hz). ^{13}C NMR ($CDCl_3$): 163.7, 147.8, 142.6, 110.4, 86.3, 63.3, 29.2, 13.8. HRMS (*m/z*): $[M+Na]^+$ calcd for $C_9H_{11}NO_5Na$: 236.0535, found, 236.0522.

Ethyl 3-(furan-3-yl)-2-nitropropanoate (6o): Obtained as an oil via step ii (0.74 g, 34% from furan-3-carbaldehyde) after heating at reflux for 4 hours in the course of the condensation step and a chromatography over silica gel (cyclohexane – ethyl acetate 95/5 to 9/1). ^{1}H NMR ($CDCl_3$): 7.40 (m, 1H), 7.34 (m, 1H), 6.30 (m, 1H), 5.26 (dd, 1H, *J* = 5.3, 9.4 Hz), 4.32 (q, 2H, *J* = 7.1 Hz), 3.39 (dd, 1H, *J* = 9.4, 15.2 Hz), 3.32 (dd, 1H, *J* = 5.4, 15.2 Hz), 1.32 (t, 3H, *J* = 7.1 Hz). ^{13}C NMR ($CDCl_3$): 163.9, 143.6, 140.8, 117.7, 110.4, 88.3, 63.2, 26.1, 13.8. HRMS (*m/z*): $[M+Na]^+$ calcd for $C_9H_{11}NO_5Na$, 236.0559, found, 236.0567.

Ethyl 3-(5-methylfuran-2-yl)-2-nitropropanoate (6p): Obtained as an oil via step iii (3.44 g, 60% from 5-methylfurfural) after heating at 140 °C for 2 hours in the course of the condensation step and a chromatography over silica gel (cyclohexane – dichloromethane 3/2). ^{1}H NMR ($CDCl_3$): 6.02 (d, 1H, *J* = 3.0 Hz), 5.86 (dd, 1H, *J* = 3.0, 0.9 Hz), 6.19 (m, 1H), 5.40 (dd, 1H, *J* = 5.5, 9.3 Hz), 4.30 (q, 2H, *J* = 7.1 Hz), 3.58 (dd, 1H, *J* = 9.3, 15.7 Hz), 3.45 (dd, 1H, *J* = 5.5, 15.7 Hz), 2.23 (s, 3H), 1.30 (t, 3H, *J* = 7.1 Hz). ^{13}C NMR ($CDCl_3$): 163.9, 152.4, 145.9, 109.3, 106.6, 86.7, 63.4, 29.5, 14.0, 13.6. HRMS (*m/z*): $[M+Na]^+$ calcd for $C_{10}H_{13}NO_5Na$: 250.0691, found, 250.0682.

Ethyl 3-(5-ethylfuran-2-yl)-2-nitropropanoate (6q): Obtained as an oil via step iii (3.68 g, 39% from 5-ethylfurfural) after heating at 140 °C for 2 hours in the course of the condensation step and two chromatography over silica gel (cyclohexane – dichloromethane 2/1 to 1/6) and (cyclohexane – ethyl acetate 97/3). ^{1}H NMR ($CDCl_3$): 6.05 (d, 1H, *J* = 3.0 Hz), 5.88 (m, 1H), 6.19 (m, 1H), 5.42 (dd, 1H, *J* = 5.7, 9.1 Hz), 4.31 (q, 2H, *J* = 7.0 Hz), 3.61 (dd, 1H, *J* = 9.1, 15.7 Hz), 3.48 (dd, 1H, *J* = 5.7, 15.7 Hz), 2.60 (q, 2H, *J* = 7.0 Hz), 1.32 (t, 3H, *J* = 7.0 Hz), 1.21 (t, 3H, *J* = 7.5 Hz). ^{13}C NMR ($CDCl_3$): 163.8, 157.9, 145.7, 108.9, 104.8, 86.5, 63.2, 29.3, 21.3, 13.8, 12.0. HRMS (*m/z*): $[M-H]^-$ calcd for $C_{11}H_{14}NO_5$: 240.0872, found, 240.0857.

Ethyl 3-(4,5-dimethylfuran-2-yl)-2-nitropropanoate (6r): Obtained as an oil via step ii (2.64 g, 46% from 4,5-dimethylfuran-2-carbaldehyde) after heating at reflux for 9 hours in the course of the condensation step, a reduction using sodium cyanoborohydride and a chromatography over silica gel (cyclohexane – ethyl acetate 95/5). ^{1}H NMR ($CDCl_3$): 5.93 (s, 1H), 5.40 (dd, 1H, *J* = 5.4 and 9.4 Hz), 4.31 (q, 2H, *J* = 7.4), 3.55 (dd, 1H, *J* = 9.4 and 15.6 Hz), 3.42 (dd, 1H, *J* = 5.4 and 15.6 Hz), 2.15 (s, 3H), 1.88 (s, 3H), 1.32 (t, 3H *J* = 7.4 Hz). ^{13}C NMR ($CDCl_3$): 163.8, 147.4, 144.5, 114.8, 111.6, 86.6, 63.2, 29.3, 13.8, 11.2, 9.7. HRMS (*m/z*): $[M+Na]^+$ calcd for $C_{11}H_{15}NO_5Na$: 264.0848, found, 236.0522.

Ethyl 2-nitro-3-(thiophen-2-yl)propanoate (6s): Obtained as an oil via step iii (3.43 g, 33% from thiophene-2-carbaldehyde) after heating at 140 °C for 4 hours in the course of the condensation step and two chromatography over silica gel (cyclohexane – dichloromethane 3/2) and (cyclohexane – ethyl acetate 97/3 to 95/5). ^{1}H NMR ($CDCl_3$): 7.23 (dd, 1H,, *J* = 1.3, 5.1 Hz), 6.96 (m, 1H), 6.92 (m, 1H), 5.35 (dd, 1H, *J* = 5.6, 9.2 Hz), 4.32 (q, 2H, *J* = 7.1 Hz), 3.83 (dd, 1H, *J* = 9.2, 15.4 Hz), 3.70 (dd, 1H, *J* = 5.6, 15.4 Hz), 1.32 (t, 3H, *J* = 7.1 Hz). ^{13}C NMR ($CDCl_3$): 163.6, 135.5, 127.3, 125.5, 88.9, 63.3, 30.5, 13.8. HRMS (*m/z*): $[M-H]^-$ calcd for $C_9H_8NO_4$, 228.0331, found, 228.0348.

General procedure for the reduction of the substituted nitroesters: The considered nitroester **6** (0.023 mol) was dissolved in ethanol (75 mL) and 37% hydrochloric acid (29 mL, 0.34 mol) and cooled to 0 °C. Powdered zinc (9.05 g, 0.13 mol, less than 10 µm in size) was added portion wise. The mixture was left to stir overnight, part of the ethanol was removed under vacuum and the residue was dispersed in water and ethyl acetate. Then, the suspension was made basic with 22% ammonia, extracted with ethyl acetate and the organic layer was washed with water, brine, dried over sodium carbonate and concentrated to dryness to yield the α-amino esters which were in some cases further purified as described below. Note: these oily α-amino esters are not very stable, and unless the corresponding hydrochloride is made (by adding 4 N HCl in dioxane and concentration to dryness), many of them will slowly dimerize into the corresponding solid piperazine-2,5-diones even when stored at −20 °C.

Ethyl 2-amino-3-(2-methoxyphenyl)propanoate (1b): Obtained as an oil (5.16 g, 90%). ^{1}H NMR ($CDCl_3$): 7.23 (m, 1H), 7.14 (m, 1H), 6.86-6.92 (m, 2H), 4.19 (q, 2H, *J* = 7.2 Hz), 3.84 (s, 3H), 3.81

(dd, 1H, *J* = 5.8, 8.1 Hz), 3.10 (dd, 1H, *J* = 5.8, 13.3 Hz), 2.84 (dd, 1H, *J* = 8.1, 13.3 Hz), 1.51 (s, 2H), 1.31 (t, 3H, *J* = 7.1 Hz). ^{13}C NMR ($CDCl_3$): 175.4, 157.8, 131.2, 128.1, 125.9, 120.4, 110.4, 60.7, 55.2, 54.5, 36.3, 14.1. HRMS (*m/z*): $[M+H]^+$ calcd for $C_{12}H_{18}NO_3$: 224.1287, found, 224.1218.

Ethyl 2-amino-3-(3-methoxyphenyl)propanoate (1c): Obtained as an oil (0.81 g, 92%). ^{1}H NMR ($CDCl_3$): 7.23 (t, 1H, *J* = 7.9 Hz), 6.77-6.82 (m, 3H), 4.19 (q, 2H, *J* = 7.1 Hz), 3.81 (s, 3H), 3.73 (dd, 1H, *J* = 5.2, 7.9 Hz), 3.08 (dd, 1H, *J* = 5.2, 13.5 Hz), 2.84 (dd, 1H, *J* = 7.9, 13.5 Hz), 1.51 (s, 2H), 1.31 (t, 3H, *J* = 7.1 Hz). ^{13}C NMR ($CDCl_3$): 174.9, 159.7, 138.9, 129.5, 121.6, 115.0, 112.2, 60.9, 55.8, 55.1, 41.2, 14.2. HRMS (*m/z*): $[M+H]^+$ calcd for $C_{12}H_{18}NO_3$: 224.1287, found, 224.1260.

Ethyl 2-amino-3-(4-methoxyphenyl)propanoate (1d): Obtained as an oil (4.2 g, 95%). ^{1}H NMR ($CDCl_3$): 7.15 (m, 2H), 6.88 (m, 2H), 4.19 (q, 2H, *J* = 7.1 Hz), 3.80 (s, 3H), 3.68 (dd, 1H, *J* = 5.3, 7.7 Hz), 3.03 (dd, 1H, *J* = 5.3, 13.7 Hz), 2.83 (dd, 1H, *J* = 7.7, 13.7 Hz), 1.48 (s, 2H), 1.31 (t, 3H, *J* = 7.1 Hz). ^{13}C NMR ($CDCl_3$): 175.1, 158.5, 130.3, 129.3, 114.0, 60.8, 56.0, 55.2, 40.2, 14.2. HRMS (*m/z*): $[M+H]^+$ calcd for $C_{12}H_{18}NO_3$: 224.1287, found, 224.1238.

Ethyl 2-amino-3-(4-(benzyloxy)phenyl)propanoate (1e): Obtained as an oil (2.65 g, 67%) after a chromatography over silica gel (dichloromethane – ethanol 96/4). ^{1}H NMR ($CDCl_3$): 7.46-7.33 (m, 5H), 7.13 (m, 2H), 6.93 (m, 2H), 5.06 (s, 2H), 4.18 (q, 2H, *J* = 7.1 Hz), 3.68 (dd, 1H, *J* = 5.3, 7.8 Hz), 3.04 (dd, 1H, *J* = 5.3, 13.7 Hz), 2.84 (dd, 1H, *J* = 7.8, 13.7 Hz), 1.47 (s, 2H), 1.27 (t, 3H, *J* = 7.1 Hz). ^{13}C NMR ($CDCl_3$): 175.1, 157.8, 133.1, 130.3, 130.0, 129.6, 128.6, 127.9, 127.4, 114.9, 70.0, 60.8, 56.0, 40.3, 14.2. HRMS (*m/z*): $[M+H]^+$ calcd for $C_{18}H_{22}NO_3$: 300.1600, found, 300.1591.

Ethyl 2-amino-3-*m*-tolylpropanoate (1f): Obtained as an oil (0.73 g, 80%). ^{1}H NMR ($CDCl_3$): 7.22 (m, 1H), 7.02 (m, 3H), 4.19 (q, 2H, *J* = 7.1 Hz), 3.72 (dd, 1H, *J* = 8.0, 5.2 Hz), 3.07 (dd, 1H, *J* = 13.6, 5.2 Hz), 2.84 (dd, 1H, *J* = 13.6, 8.0 Hz), 2.34 (s, 3H), 1.50 (s, 2H), 1.27 (t, 3H, *J* = 7.1 Hz). ^{13}C NMR ($CDCl_3$): 175.1, 138.1, 137.2, 130.1, 128.4, 127.5, 126.3, 60.9, 55.9, 41.1, 21.3, 14.2. HRMS (*m/z*): $[M+H]^+$ calcd for $C_{12}H_{18}NO_2$, 208.1338, found, 208.1334.

Ethyl 2-amino-3-(2-fluorophenyl)propanoate (1g): Obtained as an oil (0.34 g, 94%). ^{1}H NMR ($CDCl_3$): 7.23 (m, 2H), 7.06 (m, 2H), 4.17 (q, 2H, *J* = 7.1 Hz), 3.76 (dd, 1H, *J* = 7.9, 5.9 Hz), 3.11 (dd, 1H, *J* = 13.6, 5.9 Hz), 2.93 (dd, 1H, *J* = 13.6, 7.9 Hz), 1.24 (t, 3H, *J* = 7.1 Hz). ^{13}C NMR ($CDCl_3$): 174.9, 161.4 (245 Hz), 131.6 (5 Hz), 128.6 (8 Hz), 124.5 (16 Hz), 124.0, (4 Hz), 115.3 (22 Hz), 61.0, 54.8, 34.8, 13.9. HRMS (*m/z*): $[M+H]^+$ calcd for $C_{11}H_{15}FNO_2$, 212.1087, found, 212.1089.

Ethyl 2-amino-3-(4-fluorophenyl)propanoate (1i): Obtained as an oil (1.95 g, 94%). ^{1}H NMR ($CDCl_3$): 7.18 (m, 3H), 7.01 (m, 1H), 4.17 (q, 2H, *J* = 7.1 Hz), 3.68 (dd, 1H, *J* = 5.5, 7.6 Hz), 3.04 (dd, 1H, *J* = 5.5, 13.6 Hz), 2.86 (dd, 1H, *J* = 7.6, 13.6 Hz), 1.48 (s, 2H), 1.25 (t, 3H, *J* = 7.1 Hz). ^{13}C NMR ($CDCl_3$): 174.9, 161.8 (243 Hz), 132.0 (3 Hz), 130.7 (8 Hz), 115.3 (21 Hz), 61.0, 55.9, 40.2, 14.2. HRMS (*m/z*): $[M+H]^+$ calcd for $C_{11}H_{15}FNO_2$: 212.1087, found, 212.1069.

Ethyl 2-amino-3-(furan-2-yl)propanoate (1n): Obtained as an oil (4.84 g, 94%). ^{1}H NMR ($CDCl_3$): 7.34 (dd, 1H, *J* = 0.8, 1.9 Hz), 6.30 (dd, 1H, *J* = 1.9, 3.2 Hz), 6.12 (m, 1H), 4.20 (m, 2H), 3.80 (dd, 1H, *J* = 7.3, 5.2 Hz), 3.10 (dd, 1H, *J* = 14.9, 5.2 Hz), 2.99 (dd, 1H, *J* = 14.9, 7.3 Hz), 1.59 (s, 2H),

1.28 (t, 3H, *J* = 7.2 Hz). ^{13}C NMR ($CDCl_3$): 174.6, 151.5, 141.8, 110.3, 107.5, 61.0, 53.8, 33.5, 14.1. HRMS (*m/z*): $[M+H]^+$ calcd for $C_9H_{14}NO_3$, 184.0974, found, 184.0955.

Ethyl 2-amino-3-(furan-3-yl)propanoate (1o): Obtained as an oil (1.4 g, 88%). ^{1}H NMR ($CDCl_3$): 7.38 (m, 1H), 7.31 (m, 1H), 6.28 (m, 1H), 4.20 (q, 2H, *J* = 7.2 Hz), 3.65 (dd, 1H, *J* = 7.0, 5.3 Hz), 2.90 (dd, 1H, *J* = 14.3, 5.3 Hz), 2.79 (dd, 1H, *J* = 14.3, 7.0 Hz), 1.57 (s, 2H), 1.29 (t, 3H, *J* = 7.2 Hz). ^{13}C NMR ($CDCl_3$): 175.0, 143.1, 140.4, 120.0, 111.1, 60.9, 54.7, 30.2, 14.2. HRMS (*m/z*): $[M+H]^+$ calcd for $C_9H_{14}NO_3$, 184.0974, found, 184.0951.

Ethyl 2-amino-3-(5-methylfuran-2-yl)propanoate (1p): Obtained as an oil (1.26 g, 75%). ^{1}H NMR ($CDCl_3$): 5.97 (d, 1H, *J* = 2.9 Hz), 5.85 (dd, 1H, *J* = 2.9, 0.9 Hz), 4.24 – 4.14 (m, 2H), 3.72 (dd, 1H, *J* = 7.3, 5.0 Hz), 3.03 (dd, 1H, *J* = 14.9, 5.0 Hz), 2.91 (dd, 1H, *J* = 14.9, 7.3 Hz), 2.24 (s, 3H), 1.57 (s, 2H), 1.27 (t, 3H, *J* = 7.1 Hz). ^{13}C NMR ($CDCl_3$): 174.9, 151.5, 149.6, 108.5, 106.2, 61.1, 54.0, 33.8, 14.3, 13.6. HRMS (*m/z*): $[M+H]^+$ calcd for $C_{10}H_{16}NO_3$, 198.1130, found, 198.1089.

Ethyl 2-amino-3-(5-ethylfuran-2-yl)propanoate (1q): Obtained as an oil (1.83 g, 56%) after a chromatography over silica gel (dichloromethane – ethanol 98/2 to 97/3). ^{1}H NMR ($CDCl_3$): 6.00 (d, 1H, *J* = 3.0 Hz), 5.87 (d, 1H, *J* = 3.0 Hz), 4.20 (m, 2H), 3.74 (dd, 1H, *J* = 7.2, 5.1 Hz), 3.05 (dd, 1H, *J* = 14.9, 5.1 Hz), 2.94 (dd, 1H, *J* = 14.9, 7.2 Hz), 2.24 (q, 2H, *J* = 7.5 Hz), 1.58 (s, 2H), 1.29 (t, 3H, *J* = 7.2 Hz), 1.21 (t, 3H, *J* = 7.5 Hz). ^{13}C NMR ($CDCl_3$): 174.7, 157.1, 149.3, 108.1, 104.4, 60.9, 53.8, 33.7, 21.3, 14.2, 12.1. HRMS (*m/z*): $[M+H]^+$ calcd for $C_{11}H_{18}NO_3$, 212.1287, found, 212.1261.

Ethyl 2-amino-3-(4,5-dimethylfuran-2-yl)propanoate (1r): Obtained as an oil (1.72 g, 79%). ^{1}H NMR ($CDCl_3$): 5.88 (s, 1H), 4.20 (m, 2H), 3.00 (dd, 1H, *J* = 4.8, 14.9 Hz), 2.87 (dd, 1H, *J* = 7.5, 14.9 Hz), 2.15 (s, 3H), 1.89 (s, 3H), 1.64 (s, 2H), 1.29 (t, 3H, *J* = 7.1 Hz). ^{13}C NMR ($CDCl_3$): 174.8, 148.1, 146.5, 114.4, 110.9, 60.9, 53.8, 33.6, 14.2, 11.2, 9.8. HRMS (*m/z*): $[M+H]^+$ calcd for $C_{11}H_{18}NO_3$, 212.1287, found, 212.1246.

Ethyl 2-amino-3-(thiophen-2-yl)propanoate (1s): Obtained as an oil (2.5 g, 58%) after dilution of in ethyl acetate, extraction with 1 N hydrochloric acid and, upon basification of this aqueous phase with 22% ammonia an extraction with ethyl acetate. The organic layer was then washed with brine, dried over sodium carbonate and concentrated to dryness. ^{1}H NMR ($CDCl_3$): 7.19 (dd, 1H,, *J* = 1.1, 5.1 Hz), 6.96 (dd, 1H, *J* = 3.3, 5.1 Hz), 6.88 (m, 1H), 4.21 (q, 2H, *J* = 7.1 Hz), 3.72 (dd, 1H, *J* = 7.2, 4.8 Hz), 3.30 (dd, 1H, *J* = 14.7, 4.8 Hz), 3.17 (dd, 1H, *J* = 14.7, 7.2 Hz), 1.57 (s, 2H), 1.29 (t, 3H, *J* = 7.1 Hz). ^{13}C NMR ($CDCl_3$): 174.5, 139.0, 126.9, 126.5, 124.5, 61.1, 57.7, 35.1, 14.2. HRMS (*m/z*): $[M+H]^+$ calcd for $C_9H_{14}NO_2S$, 200.0745, found, 200.0726.

Preparation of (2-(trifluoromethyl)phenyl)methylene diacetate (7): In a glass tube fitted with a Teflon-covered screwcap, 2-(trifluoromethyl)benzaldehyde (1.10 g, 6.30 mmol), acetic anhydride (0.66 g, 6.44 mmol) and then indium chloride (0.014 g 0.0644 mmol) were added. The tube was stoppered and stirred for 12 hours at room temperature. The resulting gel was dispersed in dichloromethane, this was filtered on a silica gel plug and the filtrate concentrated to dryness to give pure compound **7** as a solid (1.65 g, 94%). ^{1}H NMR ($CDCl_3$): 8.03 (s, 1H), 7.79 (m, 1H), 7.73 (m,

1H), 7.64 (m, 1H), 7.54 (m, 1H), 2.15 (s, 6H). ^{13}C NMR ($CDCl_3$): 168.2, 134.1 (2 Hz), 132.2, 129.7, 128.1 (32 Hz), 127.8, 126.3 (6 Hz), 123.7 (274 Hz), 86.2 (2 Hz), 20.6.

Preparation of (2-(trifluoromethyl)phenyl)methylene bis(2,2-dimethylpropanoate) (8): In a glass tube fitted with a Teflon-covered screwcap, 2-(trifluoromethyl)benzaldehyde (1.0 g, 5.74 mmol), pivalic anhydride (1.09 g, 5.85 mmol) and then indium chloride (0.013 g 0.0574 mmol) were added. The tube was stoppered and heated at 60 °C for 3 hours. The resulting gel was dispersed in dichloromethane, this was filtered on a silica gel plug and the filtrate concentrated to dryness to give pure compound **8** as an oil (1.98 g, 95%). ^{1}H NMR ($CDCl_3$): 7.97 (s, 1H), 7.78 (m, 1H), 7.73 (m, 1H), 7.64 (m, 1H), 7.53 (m, 1H), 1.26 (s, 18H). ^{13}C NMR ($CDCl_3$): 175.7, 134.4 (2 Hz), 132.1, 129.5, 128.2 (32 Hz), 127.7, 126.3 (5 Hz), 123.8 (274 Hz), 86.2 (2 Hz), 38.7, 26.8.

Preparation of ethyl 2-nitro-3-(2-(trifluoromethyl)phenyl)propanoate (6j) via compound 8: In a glass tube fitted with a Teflon-covered screwcap, 2-(trifluoromethyl)benzaldehyde (1.17 g, 6.71 mmol), pivalic anhydride (1.27 g, 6.85 mmol) and then indium(III) chloride (0.015 g, 0.0671 mmol) were added. The tube was stoppered and heated at 60 °C for 3 hours. To this was added ethyl nitroacetate (0.89 g, 6.71 mmol), the tube stoppered and heated at 140 °C for 10 hours. The resulting oil was diluted in isopropanol (20 mL), sodium borohydride (0.30 g, 8.06 mmol) was added, the suspension brought to reflux for 10 seconds and left to cool for 15 minutes. This was cautiously made acid with acetic acid, diluted in water and extracted with ethyl acetate. The organic layer was washed with water, brine, dried over magnesium sulfate and concentrated to dryness. The residue was purified by two chromatography over silica gel (cyclohexane – dichloromethane 4/1) and (cyclohexane – ethyl acetate 9/1) to give compound **6j** as an oil (0.25 g, 13%). ^{1}H NMR ($CDCl_3$): 7.71 (m, 1H), 7.51 (m, 1H), 7.43 (m, 1H), 7.32 (m, 1H), 5.39 (dd, 1H, *J* = 5.8, 9.0 Hz), 4.31 (m, 2H), 3.76 (m, 2H), 1.30 (t, 3H, *J* = 7.05 Hz). ^{13}C NMR ($CDCl_3$): 163.7, 132.5 (2 Hz), 132.4, 131.7, 128.9 (29 Hz), 128.2, 126.7 (6 Hz), 124.3 (273 Hz), 88.6, 63.3, 31.1, 13.7. HRMS (*m/z*): $[M-H]^-$ calcd for $C_{12}H_{11}F_3NO_4$, 290.0640, found, 290.0671. *Note*: the indium chloride from the first step was found to be essential for the condensation reaction as the use of purified compound **8** instead did not lead to any reaction.

Preparation of ethyl 2-methyl-2-nitro-3-phenylpropanoate (9): Under argon, compound **6a** (3.84 g, 17.1 mmol) was dissolved in DMF (50 mL, dried over 4 Å MS). To this was added sodium hydride (0.70 g, 17.5 mmol, 60% over mineral oil). At the end of hydrogen evolution, methyl iodide (1.6 mL, 25.79 mmol) was added and this was stirred under an inert atmosphere at 20 °C overnight. The solution was concentrated to dryness, this was dispersed in water and ethyl acetate, the organic layer was washed with water, brine, dried over magnesium sulfate and concentrated to dryness and the residue purified by a chromatography over silica gel (cyclohexane – dichloromethane 2/1) to give compound **9** as a 94% pure oil (1.89 g, 46%) as seen from the NMR data which were identical with the reported one [4]. *Note*: the impurity was tentatively identified as (2-nitroethyl)benzene.

Preparation of ethyl 2-amino-2-methyl-3-phenylpropanoate (10): By using the reduction protocol described above for the preparation of compound **1**, compound **10** was obtained from compound **9** as a white solid (1.66 g, 90%) after the addition of a 4 N solution of hydrogen chloride in dioxane and a thorough concentration to dryness. The NMR data were identical with the reported one [4].

Ethyl 3-(furan-2-yl)-2-methyl-2-nitropropanoate (11): By using the protocol described above for the preparation of compound **9**, compound **11** was obtained as a 91% pure oil (0.6 g, 43%) after a chromatography over silica gel (cyclohexane – dichloromethane 2/1 to 1/1). ^{1}H NMR ($CDCl_3$): 7.33 (dd, 1H, *J* = 1.9, 0.8 Hz), 6.30 (dd, 1H, *J* = 3.2, 1.9 Hz), 6.15 (dd, 1H, *J* = 3.2, 0.8 Hz), 4.28 (q, 2H, *J* = 7.1 Hz), 3.67 (d, 1H, *J* = 15.2 Hz), 3.53 (d, 1H, *J* = 15.2 Hz), 1.74 (s, 3H), 1.29 (t, 3H, *J* = 7.1 Hz). ^{13}C NMR ($CDCl_3$): 166.9, 147.8, 142.9, 110.8, 109.9, 92.0, 63.2, 35.3, 21.3, 13.9. HRMS (*m/z*): $[M+Na]^+$ $C_{10}H_{13}NO_5Na$, 250.0691, found: 250.0702.

Ethyl 2-amino-3-(furan-2-yl)-2-methylpropanoate (12): By using the reduction protocol described above for the preparation of compound **1**, compound **12** was obtained from compound **11** as an oil (0.39 g, 75%). ^{1}H NMR ($CDCl_3$): 7.30 (dd, 1H, *J* = 1.9, 0.8 Hz), 6.28 (dd, 1H, *J* = 3.1, 1.9 Hz), 6.09 (dd, 1H, *J* = 3.1, 0.8 Hz), 4.16 (q, 2H, *J* = 7.2 Hz), 3.13 (d, 1H, *J* = 14.6 Hz), 2.87 (d, 1H, *J* = 14.6 Hz), 1.76 (s, 2H), 1.37 (s, 3H), 1.26 (t, 3H, *J* = 7.2 Hz). ^{13}C NMR ($CDCl_3$): 176.8, 151.6, 142.0, 110.3, 108.2, 61.3, 58.0, 39.5, 26.6, 14.2. HRMS (*m/z*): $[M+H]^+$ $C_{10}H_{16}NO_3$, 198.1130, found: 198.1075.

Ethyl 3-(furan-2-yl)-2-nitrobutanoate (13): Under an argon atmosphere, compound **2n** (0.5 g, 2.37 mmol) was dissolved in dry THF (10 mL) and cooled to 0 °C. In another flask, under argon, a 1.9 M 2-methyltetrahydrofuran solution of zinc chloride (3.7 mL, 7.11 mmol) was cooled to 0 °C and a THF solution of methyl lithium (6.7 mL, 10.67 mmol) was injected. The mixture was stirred for 15 minutes and the resulting whitish solution was then transferred to the solution of compound **2n** described above. After stirring the solution for 2 hours, saturated solution of ammonium chloride was cautiously added followed by ethyl acetate and an excess of ammonium hydroxide. The separated organic layer was washed with water, brine, dried over magnesium sulfate and concentrated to dryness. The residue was purified by a chromatography over silica gel (cyclohexane – ethyl acetate 95/5) to give pure compound **13** as an oily 45:55 mixture of diasteroisomers (0.23 g, 43%). ^{1}H NMR ($CDCl_3$, minor isomer): 7.33 (dd, 1H, *J* = 0.8, 1.8 Hz), 6.29 (dd, 1H, *J* = 1.8, 3.3 Hz), 6.16 (m, 1H), 5.33 (d, 1H, *J* = 8.7 Hz), 4.29 (q, 2H, *J* = 7.3 Hz), 3.93 (m, 1H), 1.41 (d, 3H, *J* = 7.2 Hz), 1.29 (t, 3H, *J* = 7.3 Hz). ^{1}H NMR ($CDCl_3$, major isomer): 7.34 (dd, 1H, *J* = 0.8, 1.8 Hz), 6.30 (dd, 1H, *J* = 1.8, 3.3 Hz), 6.17 (m, 1H), 5.34 (d, 1H, *J* = 8.7 Hz), 4.17 (m, 2H), 3.93 (m, 1H), 1.43 (d, 3H, *J* = 7.2 Hz), 1.19 (t, 3H, *J* = 7.3 Hz). HRMS (*m/z*): this compound did not ionize.

Ethyl 2-amino-3-(furan-2-yl)butanoate (14): By using the reduction protocol described above for the preparation of compounds **1**, compound **14** was obtained from **13** as an oily 45:55 mixture of diasteroisomers (0.52 g, 85%). ^{1}H NMR ($CDCl_3$, minor isomer): 7.33 (dd, 1H, *J* = 0.8, 1.8 Hz), 6.31 (dd, 1H, *J* = 1.8, 3.3 Hz), 6.10 (m, 1H), 4.20 (m, 2H), 3.60 (d, 1H, *J* = 4.35 Hz), 3.38 (m, 1H), 1.66

(s(br), 2H), 1.28 (m, 3H), 1.28 (m, 3H). ^{1}H NMR ($CDCl_3$, major isomer): 7.36 (dd, 1H, J = 0.8, 1.8 Hz), 6.32 (dd, 1H, J = 1.8, 3.3 Hz), 6.12 (m, 1H), 4.20 (m, 2H), 3.87 (d, 1H, J = 4.35 Hz), 3.38 (m, 1H), 1.66 (s(br), 2H), 1.37 (d, 3H, J = 7.0 Hz), 1.28 (m, 3H). HRMS (*m/z*): HRMS (*m/z*): $[M+H]^+$ calcd for $C_{10}H_{16}NO_3$, 198.1130, found, 198.1131.

Ethyl 5-phenyl-4,5-dihydroisoxazole-3-carboxylate (16): This compound was prepared using a protocol similar as previously reported [5]. In a 100 mL glass tube fitted with a Teflon-covered screw cap, styrene (3.42 g, 32.0 mmol), ethyl nitroacetate (8.74 g, 65.60 mmol) and 1,4-diazabicyclo[2.2.2]octane (DABCO, 0.37 g, 3.20 mmol) were dissolved in ethanol (20 mL; dried over 4 Å MS). The tube was stoppered and heated at 80 °C for 100 hours. This was cooled and opened with extra precautions (see note below). The solution was concentrated to dryness and purified by a chromatography over silica gel (cyclohexane – dichloromethane 1/2). The corresponding fraction was heated at 95 °C under a 4 mbar pressure for a while to completely remove traces of unreacted ethyl nitroacetate to yield compound **16** as an oil (5.24 g, 72%) with NMR spectra similar to the previously described one [6]. Note: Upon opening the tube a serious overpressure was observed along with the evolution of plenty of bubbles. The evolving gas was identified by bubbling it into a filtered solution of calcium hydroxide which clouded immediately thus proving it was carbon dioxide. This phenomenon is very much in accord with a previous report focusing on the thermolysis of ethyl nitroacetate to give formonitrile oxide [7]. Since two equivalents of ethyl nitroacetate are used in our preparation of **16**, we suggest that the evolving formonitrile oxide is actually the species responsible for the dehydration of the other equivalent of ethyl nitroacetate into the ethoxy-carbonylformonitrile oxide required to obtain a [2 + 3] cycloaddition. Moreover, we stopped using a closed reactor when running this reaction on a larger scale and it functioned as well in an open flask.

Preparation of ethyl 2-amino-4-phenylbutanoate (18): Compound **16** (0.47 g, 2.14 mmol), ammonium formate (2.7 g, 21.4 mmol) and 10% palladium over charcoal (0.11 g, 0.10 mmol) were heated to reflux in ethanol (50 mL) for one hour. This was filtered, the insoluble washed with a small amount of ethanol and 37% hydrochloric acid (1.6 mL, 13.6 mmol) was added before adding powdered zinc portion wise (0.42 g, 6.3 mmol). The mixture was stirred for 90 minutes, then the resulting solution was diluted in water, made basic with 22% ammonia and extracted with ethyl acetate. The organic layer was washed with water, brine, dried over sodium carbonate and concentrated to dryness to yield the amino ester **18** as an oil (0.32 g, 72%). ^{1}H NMR ($CDCl_3$): 7.31 (m, 2H), 7.22 (m, 3H), 4.19 (m, 2H), 3.46 (dd, 1H, J = 5.3, 7.9 Hz), 2.76 (m, 2H), 2.07 (m, 1H), 1.87 (m, 1H), 1.65 (s, 2H), 1.30 (t, 3H, J = 7.1 Hz). ^{13}C NMR ($CDCl_3$): 175.9, 141.3, 128.5, 128.4, 126.0, 60.8, 54.0, 36.5, 32.0, 14.2. NMR data identical with recently reported one [8].

5-Phenyl-4,5-dihydroisoxazole-3-carboxylic acid (19): Compound **16** (0.86 g, 3.93 mmol) and sodium hydroxide (2.11 g, 58.98 mmol) were dissolved in cold ethanol (5 mL) and water (5 mL). The solution was stirred at 0 °C for 2 hours, diluted in water and made acidic with 1 N hydrochloric acid. The mixture was extracted with ethyl acetate, the organic layer was washed with water, brine, dried

over magnesium sulfate and concentrated to dryness to yield acid **19** as a tan powder (0.64 g, 85%). ^{1}H NMR ($CDCl_3$): 8.43 (s(br), 1H), 7.45-7.33 (m, 5H), 5.89 (dd, 1H, *J* = 9.0, 11.6 Hz), 3.68 (dd, 1H, *J* = 11.6, 17.8 Hz), 3.26 (dd, 1H, *J* = 9.0, 17.8 Hz). ^{13}C NMR ($CDCl_3$): 163.10, 150.7, 139.0, 129.0, 128.9, 125.9, 86.1, 40.6. HRMS (*m/z*): $[M+H]^+$ calcd for $C_{10}H_9NO_3$, 192.0661, found, 192.0661.

Ethyl (5-phenyl-4,5-dihydroisoxazole-3-carbonyl)glycinate (21a): Under an inert atmosphere, compound **19** (1.70 g, 8.89 mmol), glycine ethyl ester hydrochloride (1.24 g, 8.89 mmol) and *N*,*N*-diisopropylethylamine (3.3 mL, 19.56 mmol) were dissolved in dry tetrahydrofuran (20 mL). Then, (1*H*-benzotriazol-1-yloxy)(dimethylamino)-*N*,*N*-dimethylmethaniminium tetrafluoroborate (TBTU, 3.16 g, 9.78 mmol) was added and the solution stirred overnight at room temperature. Afterward, it was diluted in ethyl acetate, the organic layer washed with water, brine, dried over magnesium sulfate and concentrated to dryness. The residue was purified by a chromatography over silica gel (dichloromethane – ethanol 99/1) to give compound **21a** an oil (1.38 g, 80%). ^{1}H NMR ($CDCl_3$): 7.43-7.33 (m, 5H), 7.18 (m, 1H), 5.78 (dd, 1H, *J* = 8.9, 11.4 Hz), 4.27 (q, 2H, *J* = 7.2 Hz), 4.16 (m, 2H), 3.68 (dd, 1H, *J* = 11.5, 17.9 Hz), 3.28 (dd, 1H, *J* = 8.9, 18.0 Hz), 1.32 (t, 3H, *J* = 7.0 Hz). ^{13}C NMR ($CDCl_3$): 169.1, 159.8, 153.1, 139.5, 128.9, 128.6, 125.9, 85.0, 61.7, 41.2, 41.0 14.1. HRMS (*m/z*): $[M+Na]^+$ calcd for $C_{14}H_{16}N_2O_4Na$, 299.1008, found, 299.1010.

Ethyl (5-phenyl-4,5-dihydroisoxazole-3-carbonyl)phenylalaninate (21b): Under an inert atmosphere, compound **19** (1.66 g, 8.68 mmol), phenylalanine ethyl ester hydrochloride (1.99 g, 8.68 mmol) and *N*,*N*-diisopropylethylamine (7.8 mL, 46.02 mmol) were dissolved in dry tetrahydrofuran (20 mL). Then, TBTU (5.85 g, 18.23 mmol) was added and the solution stirred for 20 hours at room temperature. Afterward, it was diluted in ethyl acetate, the organic layer washed with water, brine, dried over magnesium sulfate and concentrated to dryness. The residue was purified by a chromatography over silica gel (dichloromethane) to yield the mixture of diasteroisomers **21b** as colorless oil (2.65 g, 83%). ^{1}H NMR ($CDCl_3$): 7.42-7.26 (m, 8H), 7.21-7.18 (m, 2H), 7.14 (d, 1H, *J* = 7.9 Hz), 5.76 (dd, 1H, *J* = 8.9, 11.5 Hz), 4.95 (dt, 1H, *J* = 6.2, 8.2 Hz), 4.21 (m, 2H), 3.65 (dd, 1H, *J* = 11.5, 17.9 Hz), 3.24 (dd, 1H, *J* = 9.0, 17.9 Hz), 3.20 (m, 2H), 1.26 (t, 3H, *J* = 7.0 Hz). ^{13}C NMR ($CDCl_3$): 170.8, 159.2, 153.0, 139.6, 135.6, 129.3, 128.9, 128.7, 128.6, 127.2, 125.9, 84.9, 61.6, 53.3, 41.0, 38.2, 14.1. HRMS (*m/z*): $[M+Na]^+$ calcd for $C_{21}H_{22}N_2O_4Na$, 389.1477, found, 389.1480.

Ethyl (2-(hydroxyimino)-4-phenylbutanoyl)glycinate (22a): Compound **21a** (1.21 g, 4.38 mmol), ammonium formate (5.54 g, 87.59 mmol) and 10% palladium over charcoal (0.23 g, 0.22 mmol) were heated to reflux in ethanol (40 mL) for one hour. After filtration, the obtained solution was concentrated to dryness to yield compound **22a** (1.41 g) as colorless oil still containing some amounts of ammonium formate which was used without further purification in the next step.

Ethyl (2-(hydroxyimino)-4-phenylbutanoyl)phenylalaninate (22b): Using the protocol described above for the preparation of compound **21a**, compound **21b** (1.89 g) was obtained as a solid which was used without further purification in the next step.

3-Phenethylpiperazine-2,5-dione (23a): The crude compound **22a** described above (1.41 g) was dissolved in ethanol (30 mL), cooled to 0 °C and 37% hydrochloric acid (5.1 mL, 61.3 mmol) was added. To this solution, powdered zinc (1.72 g, 26.3 mmol) was added portion wise and the suspension stirred for 2 hours at room temperature. The resulting solution was diluted in water, made basic with 22% ammonia and extracted with ethyl acetate. The organic layer was washed with water, brine, dried over sodium carbonate and concentrated to dryness. The resulting oil was heated under an argon atmosphere at 160 °C for 10 hours. The resulting solid was dispersed in boiling toluene, cooled, filtered and dried under vacuum at 60 °C to yield compound **23a** (0.56 g, 59% from compound **21a**) as beige solid. ^{1}H NMR (DMSO-d_6): 8.30 (s(br), 1H), 8.02 (s(br), 1H), 7.36-7.16 (m, 5H), 3.85-3.77 (m, 2H), 3.71 (dd, 1H, J = 2.5, 17.3 Hz), 2.70-2.58 (m, 2H), 2.00-1.94 (m, 2H). ^{13}C NMR (DMSO-d_6): 168.3, 166.8, 141.7, 128.8 (two signals?), 126.3, 54.2, 44.8, 35.0, 30.7. HRMS (*m/z*): $[M+Na]^+$ calcd for $C_{12}H_{14}N_2O_2Na$, 241.0953, found, 241.0948.

3-Benzyl-6-phenethylpiperazine-2,5-dione (23b): The reduction step was undertaken as described for the preparation of compound **23a** described above and the resulting oil was heated at 140 °C under an argon atmosphere for 10 hours. The resulting solid was recrystallized in toluene to yield compound **23b** as yellow solid (1.02 g, 65% from compound **21b**). ^{1}H NMR (DMSO-d_6, mixture of diasteroisomers): 8.81-8.13 (m, 2H), 7.31-7.08 (m, 8H), 6.99 (m, 1H), 4.23 (s(br), 0.5H), 4.17 (s(br), 0.5H), 3.66 (m, 0.5H), 3.16 (m, 1H), 2.98 (m, 0.5H), 2.89 (m, 1H), 2.51 (m, 1H), 2.03 (m, 1H), 1.82 (m, 1H), 1.36 (m, 0.5H), 1.05 (m, 0.5H). ^{13}C NMR (DMSO-d_6): 168.0, 167.9, 167.2, 166.7, 141.9, 141.7, 136.5, 136.4, 130.9, 130.5, 128.8, 128.7, 128.6, 128.6, 128.5, 128.4, 127.2, 127.1, 126.2, 126.1, 56.1, 55.8, 54.0, 53.1, 39.1, 38.5, 35.4, 34.2, 30.2, 30.1. HRMS (*m/z*): $[M+Na]^+$ calcd for $C_{19}H_{20}N_2O_2$, 331.1422, found, 331.1429.

Synthesis of compounds 28 and 29: Step 1, preparation of compound **25** [9]. Under an inert atmosphere, *N*-(prop-2-yn-1-yl)benzamide (0.5 g, 3.1 mmol) was dissolved in dichloromethane (15 mL; dried over 4 Å MS) and [bis(trifluoromethanesulfonyl)imidate](triphenylphosphine)gold(I) 2:1 toluene adduct (0.093 g, 0.12 mmol) was added. The mixture was stirred overnight at room temperature. Then, ethyl 2-chloro-2-(hydroxyimino)acetate (**26**) [10] (0.57 g, 3.7 mmol) was added followed by a solution of triethylamine (0.55 mL, 3.9 mmol) in dry dichloromethane (1.5 mL) which was slowly injected during 3.5 hours. In this experiment, the mixture was stirred for three days, diluted in ethyl acetate, the organic layer washed with water, brine, dried over magnesium sulfate and concentrated to dryness. The residue was purified by two-fold chromatography over silica gel (cyclohexane – ethyl acetate 8/1 to 2/1) and (dichloromethane – ethanol 99/1 to 98/2) to yield, in this order, compound **28** as an oil (0.24 g, 28%) and compound **29** as an oil (0.04 g, 5%; 92 % pure) as described below.

Ethyl 5-(benzamidomethyl)isoxazole-3-carboxylate (28): ^{1}H NMR ($CDCl_3$): 7.84-7.81 (m, 2H), 7.55 (m, 1H), 7.49-7.44 (m, 1H), 6.83 (s(br), 1H), 6.68 (s(br), 1H), 4.82 (m, 2H), 4.44 (q, 2H, *J* = 7.3 Hz), 1.41 (t, 3H, *J* = 7.5 Hz). ^{13}C NMR ($CDCl_3$): 170.9, 167.5, 159.7, 159.6, 133.3, 132.1, 128.7, 127.1, 103.1, 62.2, 35.6, 14.1. HRMS *(m/z)*: $[M+Na]^+$ calcd for $C_{14}H_{14}N_2O_4$, 297.0851, found, 297.0845.

Ethyl (E)-2-(hydroxyimino)-3-(2-phenyloxazol-5-yl)propanoate (29): ^{1}H NMR ($CDCl_3$): 10.2 (s(br), 1H), 8.03-7.99 (m, 2H), 7.48-7.42 (m, 3H), 6.99 (s, 1H), 4.36 (q, 2H, *J* = 7.2 Hz), 4.15 (m, 2H), 1.37 (t, 3H, *J* = 7.6 Hz). ^{13}C NMR ($CDCl_3$): 162.8, 161.1, 147.5, 146.3, 130.2, 128.7, 127.3, 126.2, 125.4, 62.2, 21.4, 14.1. HRMS *(m/z)*: $[M+H]^+$ calcd for $C_{14}H_{14}N_2O_4$, 275.1032, found, 275.1032.

Synthesis of ethyl 2-nitro-3-(2-phenyloxazol-5-yl)propanoate (30): Under argon, to a solution of compound **25** (0.00189 mol) in dichloromethane (8 mL), prepared as described above, was added ethyl nitroacetate (0.21 mL, 0.00189 mol). The mixture was cooled to 0 °C and cerium(IV) ammonium nitrate (CAN, 4 g, 0.0075 mol) dissolved in dry DMF (20 mL; the dissolution is a slow process) was slowly injected over 4.30 hours while maintaining the temperature at 0 °C. At the end of this addition, an excess of a saturated solution of sodium thiosulfate was added leading to a white suspension. (Note: this reduction step is essential as otherwise a very low yield of compound **30** is obtained). The suspension was extracted with ethyl acetate, the organic layer washed with water, brine, dried over magnesium sulfate, concentrated to dryness and the residue purified by a chromatography over silica gel (cyclohexane – ethyl acetate 6/1 to 0/1) to yield compound **30** as yellow oil (0.12 g, 22%; contaminated with about 10% of compound **32**). ^{1}H NMR ($CDCl_3$): 8.00 (m, 2H), 7.47 (m, 3H), 7.04 (s(br), 1H), 5.48 (m, 1H), 4.34 (q, 2H, *J* = 7.2 Hz), 3.78 (m, 1H), 3.66 (m, 1H), 1.32 (t, 3H, *J* = 7.4 Hz). ^{13}C NMR ($CDCl_3$): 163.3, 162.0, 145.0, 130.6, 128.8, 127.1, 126.8, 126.3, 85.8, 63.6, 26.9, 13.8. HRMS *(m/z)*: $[M+H]^+$ calcd for $C_{14}H_{15}N_2O_5$: 291.0981, found, 291.0982.

Isolation of (2-phenyloxazol-5-yl)methanol (32): In a control experiment fairly similar to the one described above, ethyl nitroacetate was omitted from the reaction. An ^{1}H NMR analysis of the crude reaction product clearly pointed out the occurrence of compound **31** and **32** and upon a chromatography over silica gel (cyclohexane – ethyl acetate 6/1 to 3/2), if compound **31**: ^{1}H NMR ($CDCl_3$): 8.01 (m, 2H), 7.53 (m, 3H), 6.85 (t, 1H, *J* = 1.2 Hz), 4.74 (s, 2H), 2.41 (t, 3H, *J* = 1.2 Hz) could not be separated from unreacted compound **25**, a pure sample of compound **32** was isolated as a white solid (0.13 g, 22%). ^{1}H NMR ($CDCl_3$): ^{1}H NMR ($CDCl_3$): 8.03 (m, 2H), 7.45 (m, 3H), 7.08 (s, 1H), 4.74 (s, 2H), 2.59 (s(br), 1H). ^{13}C NMR ($CDCl_3$): 162.0, 151.0, 130.5, 128.8, 127.2, 126.4, 125.8, 55.2. HRMS *(m/z)*: $[M+H]^+$ calcd for $C_{10}H_{10}NO_2$: 176.0712, found, 176.0714.

Ethyl 3-(4,5-dimethyloxazol-2-yl)-2-(hydroxyimino)propanoate (35): Under an inert atmosphere a solution of 2,4,5-trimethyloxazole (1.05 g, 9.44 mmol) in dry tetrahydrofuran (10 mL) was cooled to −78 °C with a dry ice bath. At this temperature 2 N lithium diisopropylamide in tetrahydrofuran (4.7

mL, 9.44 mmol) was added and the mixture stirred for 5 minutes. Then, diethyl oxalate (1.28 mL, 9.92 mmol) was added and the solution allowed to warm to 20 °C and stirred for further 10 minutes. The mixture was diluted in water, extracted with ethyl acetate, the organic layer washed with water, brine, dried over magnesium sulfate and concentrated to dryness. The resulting oil was dissolved in ethanol (100 mL), hydroxylamine hydrochloride (2.76 g, 39.7 mmol) and dry pyridine (3.3 mL, 40.8 mmol) were added and the mixture heated to reflux for 6 hours. The resulting suspension was diluted in water, extracted with ethyl acetate, the organic layer washed with water, brine, dried over magnesium sulfate and concentrated to dryness. The residue was purified by a chromatography over silica gel (cyclohexane – ethyl acetate 1/1) and the corresponding fraction was dispersed in boiling cyclohexane and filtered, after cooling, to yield the target α-hydroximino ester **35** as white powder (0.15 g, 7%). ^{1}H NMR ($CDCl_3$): 11.99 (s, 1H), 4.30 (q, 2H, *J* = 7.2 Hz), 4.08 (s, 2H), 2.20 (d, 3H, *J* = 0.8 Hz), 2.05 (d, 3H, *J* = 0.8 Hz), 1.31 (t, 3H, *J* = 7.2 Hz). ^{13}C NMR ($CDCl_3$): 163.4, 157.1, 145.7, 143.2, 130.2, 61.7, 23.8, 14.0, 10.7, 9.8. Long distance correlation experiments established the regioselectivity of the reaction. HRMS (*m/z*): $[M+H]^+$ calcd for $C_{10}H_{14}N_2O_4$, 227.1032, found, 227.1035.

Ethyl 2-amino-3-(4,5-dimethyloxazol-2-yl)propanoate (36): Compound **35** (0.33 g, 1.45 mmol) was dissolved in ethanol (5 mL) and 37% hydrochloric acid (1.1 mL, 13.1 mmol) and the solution cooled to 0 °C. Powdered zinc (0.29 g, 4.37 mmol, less than 10 µm in size) was added portion wise and the mixture left to stir for 3 hours at room temperature. The resulting solution was diluted in water, made basic with 22% ammonia, extracted with ethyl acetate, washed with water, brine and concentrated to dryness to yield pure amino ester **36** as an oil (0.23 g, 74%). ^{1}H NMR ($CDCl_3$): 4.19 (m, 2H), 3.90 (dd, 1H, *J* = 4.5, 8.1 Hz), 3.14 (dd, 1H, *J* = 4.5, 15.0 Hz), 2.96 (dd, 1H, *J* = 8.1, 15.0 Hz), 2.27 (s, 3H), 2.05 (s, 3H), 1.84 (s(l), 2H), 1.26 (t, 3H, *J* = 7.3 Hz). ^{13}C NMR ($CDCl_3$): 174.0, 158.6, 143.1, 130.3, 61.2, 52.9, 33.5, 14.1, 11.0, 9.8. HRMS (*m/z*): $[M+H]^+$ calcd for $C_{10}H_{17}N_2O_3$, 213.1229, found, 213.1230.

Benzyl *N*2-(*tert*-butoxycarbonyl)-*N*4-(prop-2-yn-1-yl)asparaginate (38): Under an inert atmosphere, propargylamine (0.17 g, 3.09 mmol), 4-(benzyloxy)-3-({[(2-methyl-2-propanyl)oxy]carbonyl}amino)-4-oxobutanoic acid (1.0 g, 3.09 mmol) and TBTU (1.0 g, 3.15 mmol) were dissolved in dry dichloromethane (40 mL; dried over 4 Å MS). Triethylamine (0.42 mL, 3.00 mmol) was then added and the solution stirred for 16 hours at room temperature under a calcium chloride-protected atmosphere. The resulting solution was diluted in additional dichloromethane, the organic layer washed with water, brine, dried over magnesium sulfate and concentrated to dryness. The residue was purified by a chromatography over silica gel (cyclohexane – ethyl acetate 2/1; very weakly detectable by UV monitoring at 280 nm) to yield compound **38** as white powder (0.87 g, 78%). ^{1}H NMR ($CDCl_3$): 7.38-7.35 (m, 5H), 5.82 (s(br), 1H), 5.74 (m, 1H), 5.21 (q, 2H, *J* = 10.2 Hz), 4.58 (m, 1H), 4.01 (m, 2H), 2.92 (dd, 1H, *J* = 4.1, 16.1 Hz), 2.75 (dd, 1H, *J* = 4.5, 14.0 Hz), 2.24 (t, 1H, *J* = 2.7 Hz), 1.45 (s, 9H,). ^{13}C NMR ($CDCl_3$): 171.2, 169.4, 155.7, 135.4, 128.5, 128.3, 128.2,

80.1, 79.2, 71.8, 67.4, 50.5, 37.8, 29.2, 28.3. HRMS (*m/z*): $[M+Na]^+$ calcd for $C_{19}H_{24}N_2O_5Na$, 383.1583, found, 383.1565.

Benzyl 2-((*tert*-butoxycarbonyl)amino)-3-(5-methylene-4,5-dihydrooxazol-2-yl)propanoate (40): Under an inert atmosphere, compound **38** (1.78 g, 4.93 mmol) and [bis(trifluoromethanesulfonyl)imidate](triphenylphosphine)gold(I) 2:1 toluene adduct (0.15 g, 0.19 mmol) were dissolved in dry toluene (16 mL). This was stirred for 2.5 hours at 60 °C and concentrated to dryness. The residue was purified by a chromatography over silica gel (cyclohexane – ethyl acetate 3/1; weakly detectable by UV monitoring at 280 nm) to yield compound **40** as colorless oil (1.43 g, 80%) which, as explained in the text, turned out to be unstable. The following proton spectrum was obtained within minutes after diluting compound **40** in $CDCl_3$. ^1H NMR ($CDCl_3$): 5.37 (d, 1H, *J* = 6.3 Hz), 4.34 (m, 1H), 4.31 (d, 2H, *J* = 2.4 Hz), 3.13 (dd, 1H, *J* = 9.1, 17.8 Hz), 2.83 (dd, 1H, *J* = 5.9, 18.3 Hz), 2.22 (t, 1H, *J* =2.9 Hz), 1.44, (s, 9H).

Characterization of *tert*-butyl (2,5-dioxo-1-(prop-2-yn-1-yl)pyrrolidin-3-yl)carbamate (39): This compound was initially isolated as by product from the preparation of compound **40** using a batch of **38** as described above but with the addition two equivalents of triethylamine. The purification of crude compound **40** by a chromatography over silica gel (cyclohexane – ethyl acetate 3/1; very weakly detectable by UV monitoring at 280 nm) and a recrystallization in a mixture of toluene and cyclohexane led to the isolation of pure compound **39** (0.55 g, 15%) Subsequently, it was demonstrated on a small scale that treatment of compound **38** with triethylamine in dichloromethane overnight resulted in its complete conversion into **39**. ^1H NMR ($CDCl_3$; not very stable): 5.37 (d, 1H, *J* = 6.3 Hz), 4.34 (m, 1H), 4.31 (d, 2H, *J* = 2.4 Hz), 3.13 (dd, 1H, *J* = 9.1, 17.8 Hz), 2.83 (dd, 1H, *J* = 5.9, 18.3 Hz), 2.22 (t, 1H, *J* =2.9 Hz), 1.44, (s, 9H). HRMS *(m/z)*: $[M+Na]^+$ calcd for $C_{12}H_{16}N_2O_4$, 275.1008, found, 275.1008.

Benzyl 2-((*tert*-butoxycarbonyl)amino)-3-(5-methyloxazol-2-yl)propanoate (41): Under an argon atmosphere, to a solution of crude compound **40** (0.58 g, 1.61 mmol) in toluene (15 mL) obtained as described above, was added 1,8-diazabicyclo[5.4.0]undec-7-ene (DBU, 2.4 mL, 16.1 mmol). The mixture was heated at 80 °C in an oil bath for 33 hours. The resulting solution was diluted in water, extracted in ethyl acetate, the organic layer washed with diluted ammonium chloride in water, brine, dried over magnesium sulfate and concentrated to dryness. The residue was purified by two-fold chromatography over silica gel (dichloromethane – ethanol 96/4) and (cyclohexane – ethyl acetate 4/1) to yield the target product as colorless oil (0.14 mg, 24%). Alternatively, a 4 N hydrogen chloride solution in dioxane (0.18 mL, 0.7 mmol) was added to a toluene solution (15 mL) of crude compound **40** (1.01 g, 2.8 mmol) obtained as described above. The mixture was stirred for 14 hours, diluted in ethyl acetate, the organic layer was washed with saturated sodium hydrogen carbonate, water, brine, concentrated to dryness and purified by a chromatography over silica gel (cyclohexane – ethyl acetate 4/1, very weak UV signal) to yield compound **41** (0.69 g, 69%). ^1H NMR ($CDCl_3$): 7.38-7.28 (m, 5H), 6.58 (m, 1H), 5.71 (d, 1H, *J* = 8.3 Hz), 5.18 (dd, 2H, *J* = 12.2, 21.8

Hz), 4.77 (m, 1H), 3.32 (dd, 1H, *J* = 5.3, 15.6 Hz), 3.19 (dd, 1H, *J* = 5.1, 15.8 Hz), 2.23 (d, 3H, *J* = 1.2 Hz), 1.44 (s (br), 9H). ^{13}C NMR ($CDCl_3$): 170.9, 159.2, 155.3, 149.0, 135.3, 128.5, 128.3, 128.2, 122.6, 80.0, 67.3, 51.4, 30.8, 28.3, 10.7. HRMS *(m/z)*: $[M+H]^+$ calcd for $C_{19}H_{24}N_2O_5$, 361.1764, found, 361.1774.

Ethyl 2-((*tert*-butoxycarbonyl)amino)-3-(5-methyloxazol-2-yl)propanoate (42): In a 20 mL Biotage reaction vial, compound **40** (0.64 g, 1.78 mmol) and DBU (0.27 mL, 1.78 mmol) were dissolved in dry ethanol (10 mL; dried over 4 Å MS). The mixture was heated at 110 °C under microwave irradiation for 4 hours. The resulting solution was diluted in ethyl acetate, the organic layer washed with a 1 N solution of ammonium chloride, brine, dried over magnesium sulfate and concentrated to dryness. The residue was purified by a chromatography over silica gel (dichloromethane – ethanol 98/2, very weak UV signal). The corresponding fraction was lyophilized for 5 to 6 days to remove traces of benzyl alcohol and gave compound **42** as yellow oil (0.27 g, 51%). ^{1}H NMR ($CDCl_3$): 6.62 (m, 1H), 5.62 (d(br, 1H), *J* = 7.8 Hz), 4.71-4.67 (m, 1H), 4.77 (m, 1H), 4.25-4.17 (m, 2H), 3.29 (dd, 1H, *J* = 5.2, 15.8 Hz), 3.19 (dd, 1H, *J* = 5.0, 15.6 Hz), 2.28 (d, 3H, *J* = 1.2 Hz), 1.46 (s (br), 9H). ^{13}C NMR ($CDCl_3$): 171.0, 159.3, 155.3, 148.9, 122.7, 79.9, 61.6, 51.4, 30.9, 28.3, 14.0, 10.7. HRMS (*m/z*): $[M+H]^+$ calcd for $C_{14}H_{22}N_2O_5$, 299.1607, found, 299.1608.

Benzyl 2-amino-3-(5-methyloxazol-2-yl)propanoate (43): Compound **41** (1.1 g, 3.05 mmol) was stirred in a stoppered flask in 4 N HCl/dioxane (7 mL) at 20 °C for 20 hours. After concentration to to dryness, the residue was dissolved in water, filtered and the solution lyophilized to yield the hydrochloride salt of compound **43** as light yellow glass still containing some water and dioxane (0.98 g, quant.). ^{1}H NMR (DMSO-d_6): 8.94 (s(br), 3H), 7.33 (m, 5H), 6.75 (q, 1H, *J* = 1.5 Hz), 5.17 (m, 2H), 4.49 (s(br), 1H), 3.43 (dd, 1H, *J* = 3.4, 16.0 Hz), 3.37 (dd, 1H, *J* = 6.7, 16.0 Hz), 2.21 (d, 3H, *J* = 1.5 Hz). ^{13}C NMR (DMSO-d_6): 168.4, 157.9, 149.5, 135.4, 128.8, 128.7, 128.4, 123.1, 67.7, 50.4, 28.6, 10.8. HRMS (*m/z*): $[M+H]^+$ calcd for $C_{14}H_{17}N_2O_3$, 261.1239, found, 261.1237.

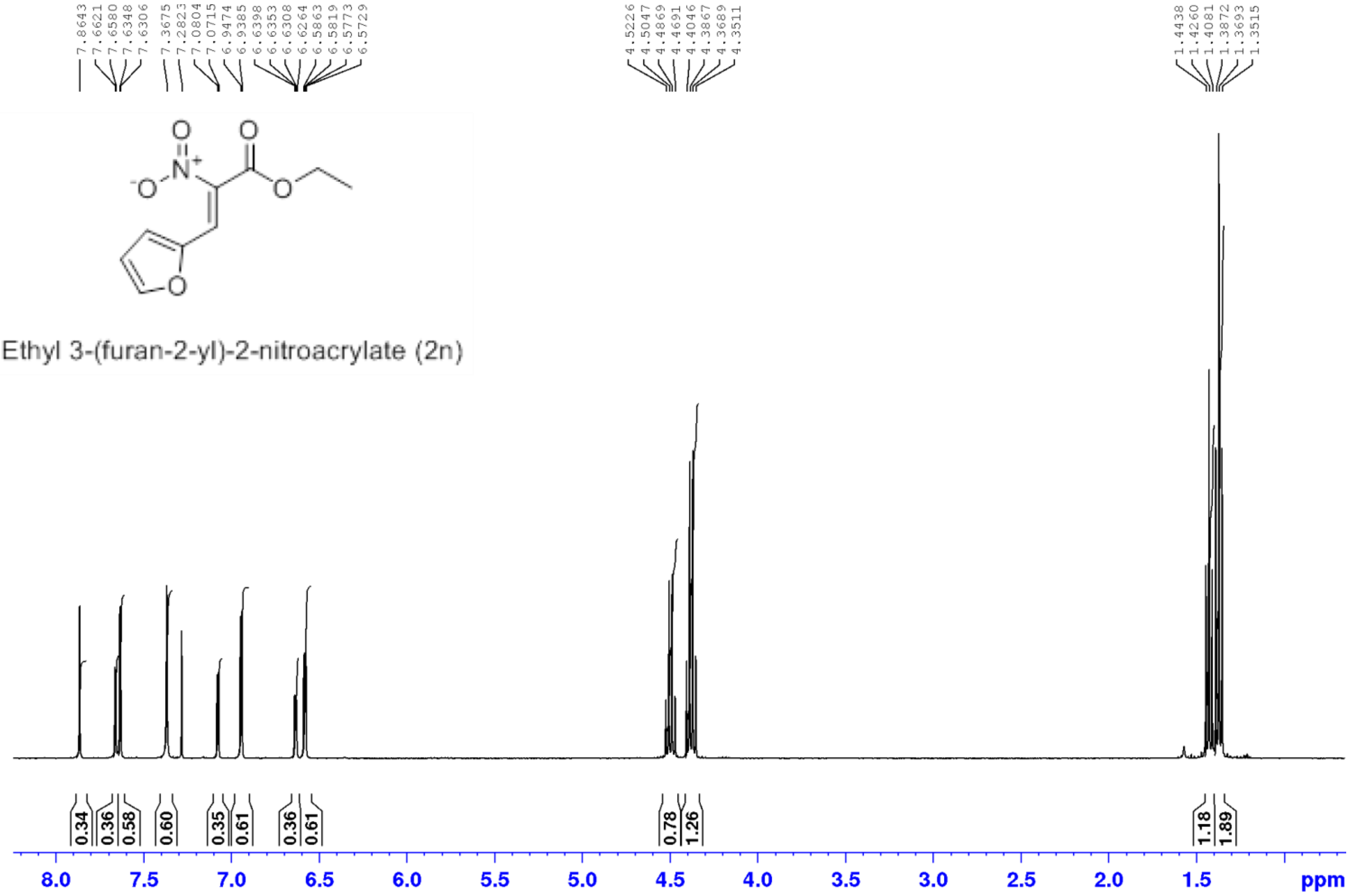
7.8643
7.6621
7.6580
7.6348
7.6306
7.3675
7.2823
7.0804
7.0715
6.9474
6.9385
6.6398
6.6353
6.6308
6.6264
6.5863
6.5819
6.5773
6.5729
4.5226
4.5047
4.4869
4.4691
4.4046
4.3867
4.3689
4.3511
1.4438
1.4260
1.4081
1.3872
1.3693
1.3515
Ethyl 3-(furan-2-yl)-2-nitroacrylate (2n)
0.34
0.36
0.58
0.60
0.35
0.61
0.36
0.61
0.78
1.26
1.18
1.89
8.0
7.5
7.0
6.5
6.0
5.5
5.0
4.5
4.0
3.5
3.0
2.5
2.0
1.5
ppm

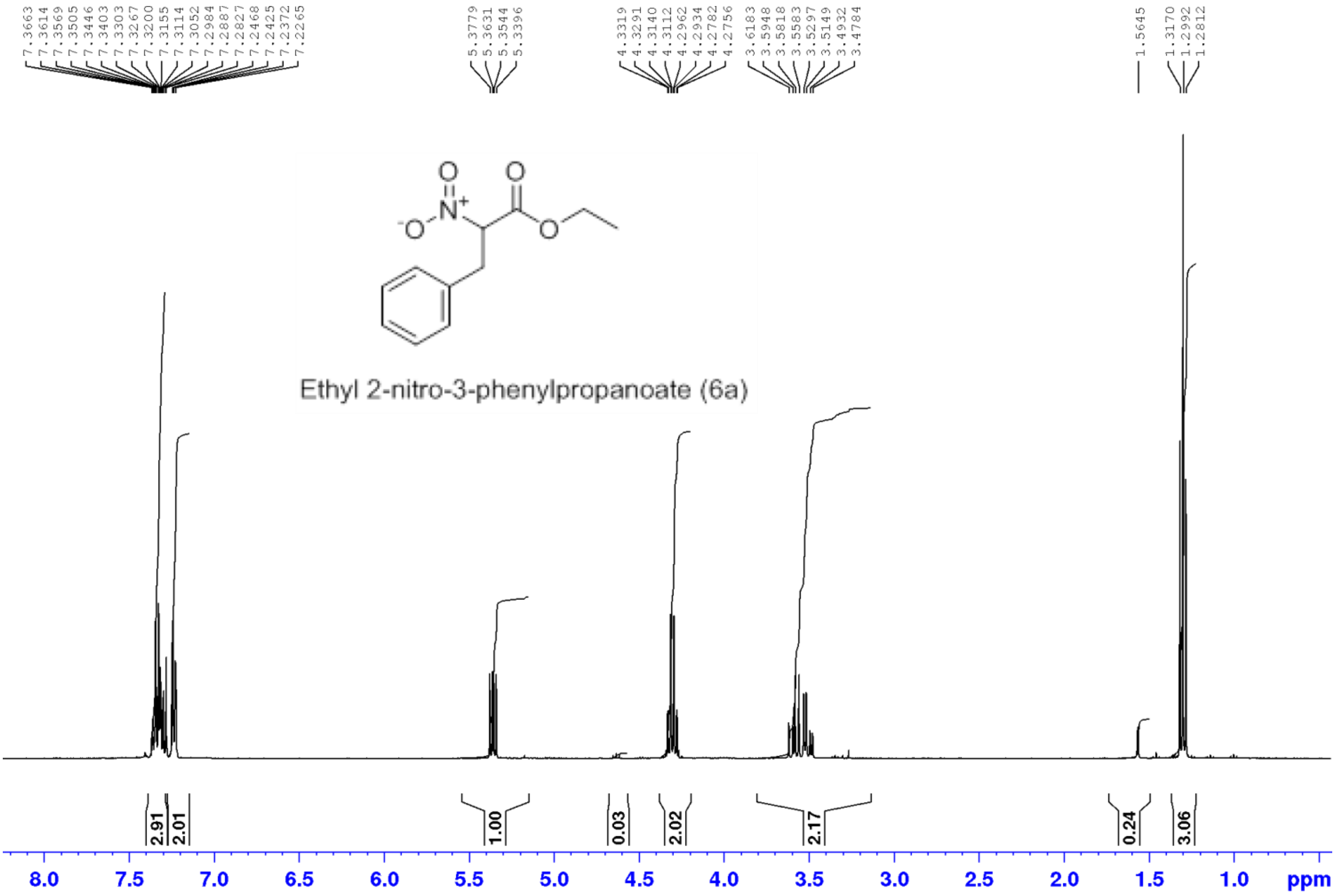
7.3663
7.3614
7.3569
7.3505
7.3446
7.3403
7.3303
7.3267
7.3200
7.3155
7.3114
7.3052
7.2984
7.2887
7.2827
7.2468
7.2425
7.2372
7.2265
5.3779
5.3631
5.3544
5.3396
4.3319
4.3291
4.3140
4.3112
4.2962
4.2934
4.2782
4.2756
3.6183
3.5948
3.5818
3.5583
3.5297
3.5149
3.4932
3.4784
1.5645
1.3170
1.2992
1.2812
Ethyl 2-nitro-3-phenylpropanoate (6a)
2.91
2.01
1.00
0.03
2.02
2.17
0.24
3.06
8.0
7.5
7.0
6.5
6.0
5.5
5.0
4.5
4.0
3.5
3.0
2.5
2.0
1.5
1.0
ppm

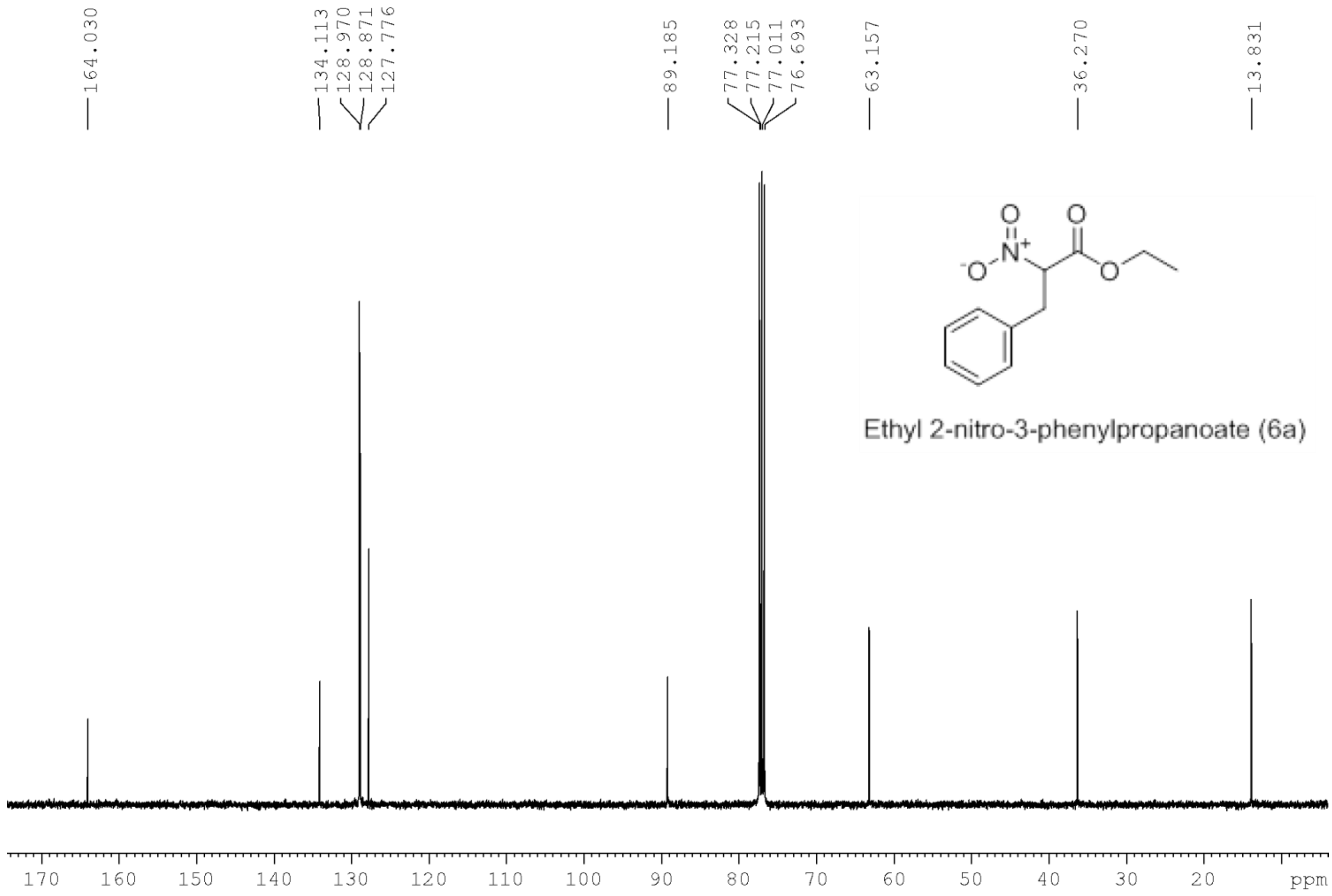

164.030
134.113
128.970
128.871
127.776
89.185
77.328
77.215
77.011
76.693
63.157
36.270
13.831
Ethyl 2-nitro-3-phenylpropanoate (6a)
170 160 150 140 130 120 110 100 90 80 70 60 50 40 30 20 ppm

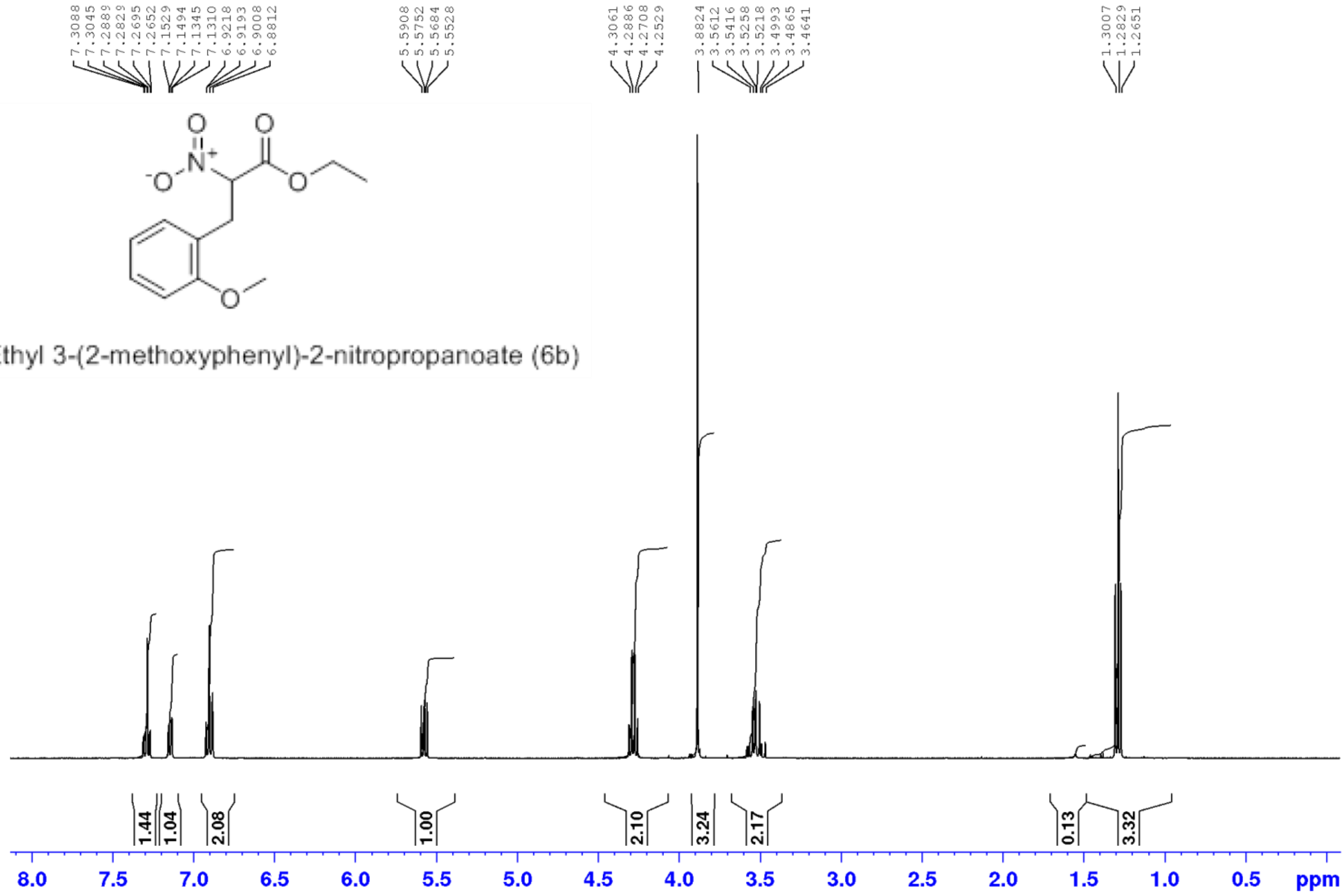

Ethyl 3-(2-methoxyphenyl)-2-nitropropanoate (6b)

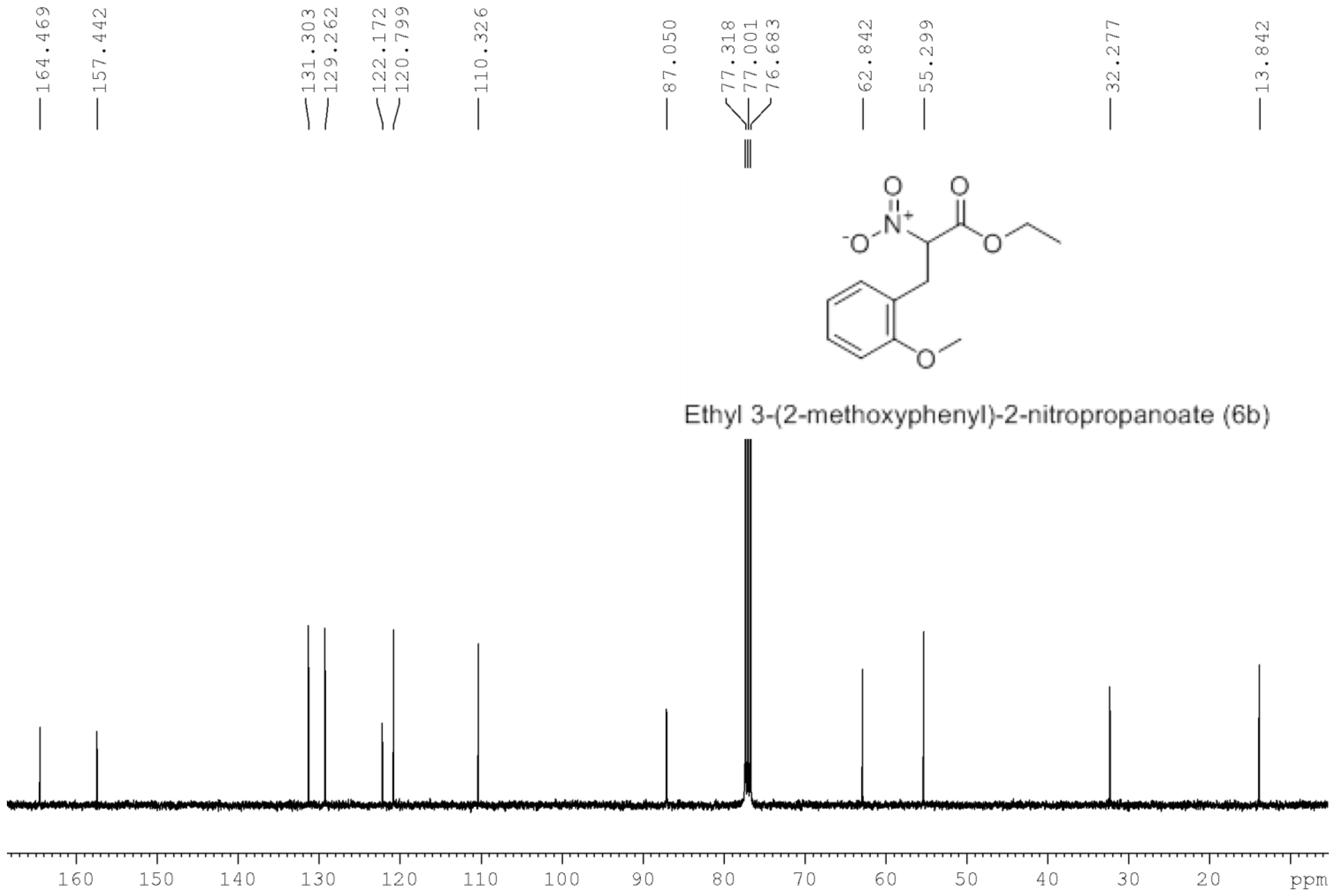
164.469
157.442
131.303
129.262
122.172
120.799
110.326
87.050
77.318
77.001
76.683
62.842
55.299
32.277
13.842
Ethyl 3-(2-methoxyphenyl)-2-nitropropanoate (6b)
160
150
140
130
120
110
100
90
80
70
60
50
40
30
20
ppm

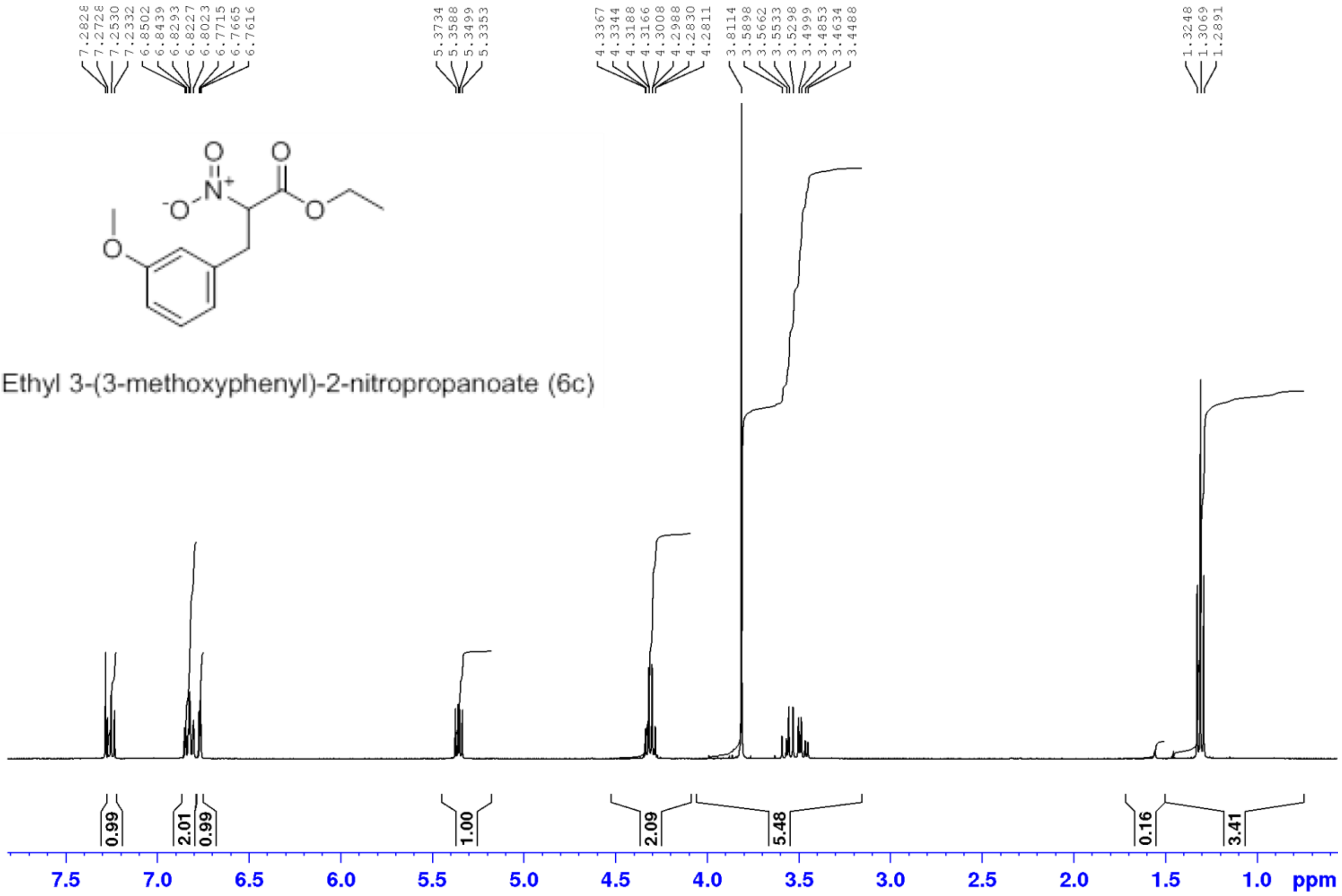
7.2828
7.2728
7.2530
7.2332
6.8502
6.8439
6.8293
6.8227
6.8023
6.7715
6.7665
6.7616
5.3734
5.3588
5.3499
5.3353
4.3367
4.3344
4.3188
4.3166
4.3008
4.2988
4.2830
4.2811
3.8114
3.5898
3.5662
3.5533
3.5298
3.4999
3.4853
3.4634
3.4488
1.3248
1.3069
1.2891
Ethyl 3-(3-methoxyphenyl)-2-nitropropanoate (6c)
0.99
2.01
0.99
1.00
2.09
5.48
0.16
3.41
7.5
7.0
6.5
6.0
5.5
5.0
4.5
4.0
3.5
3.0
2.5
2.0
1.5
1.0
ppm

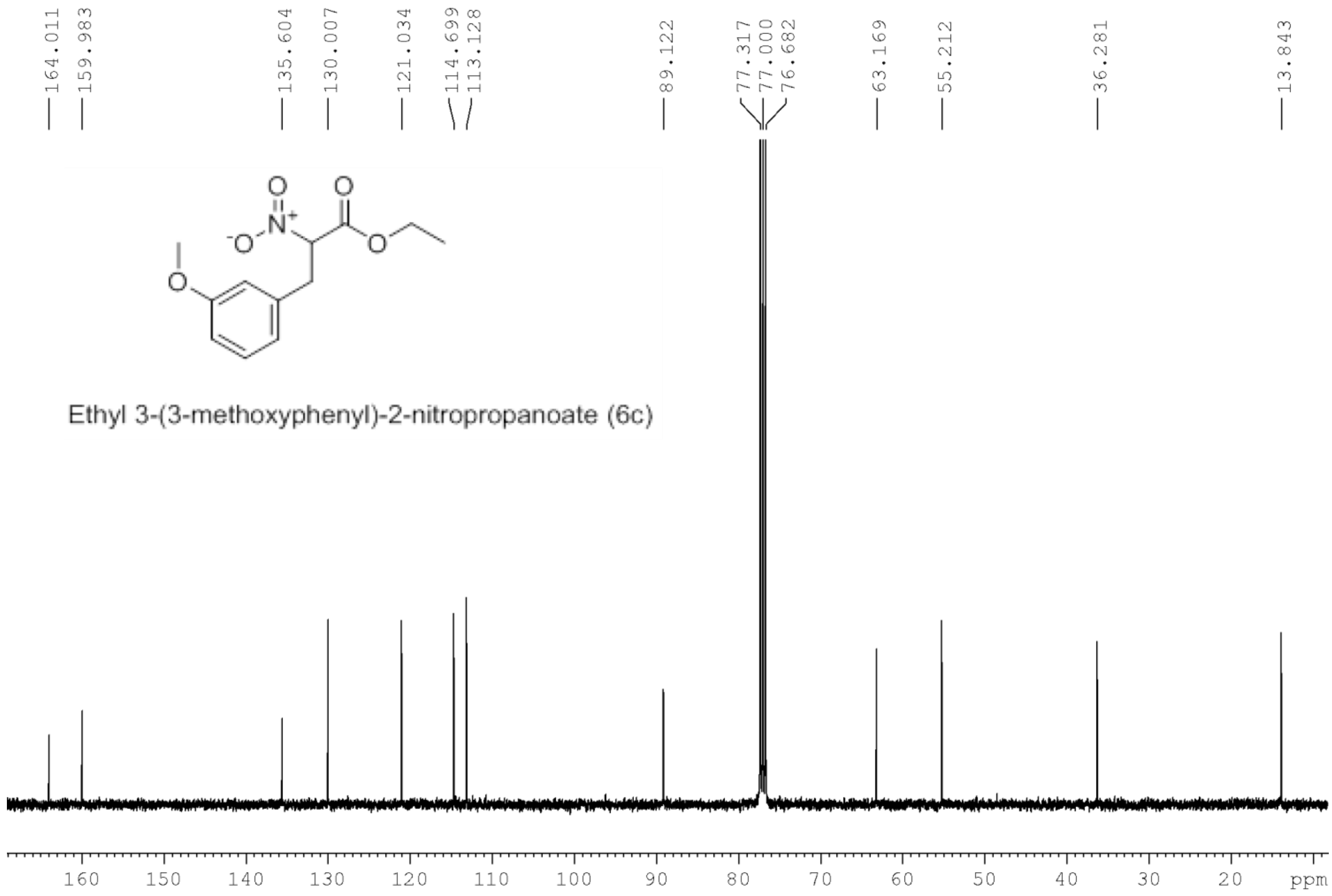
164.011
159.983
135.604
130.007
121.034
114.699
113.128
89.122
77.317
77.000
76.682
63.169
55.212
36.281
13.843
O
N+
-O
O
O
O
Ethyl 3-(3-methoxyphenyl)-2-nitropropanoate (6c)
160
150
140
130
120
110
100
90
80
70
60
50
40
30
20
ppm

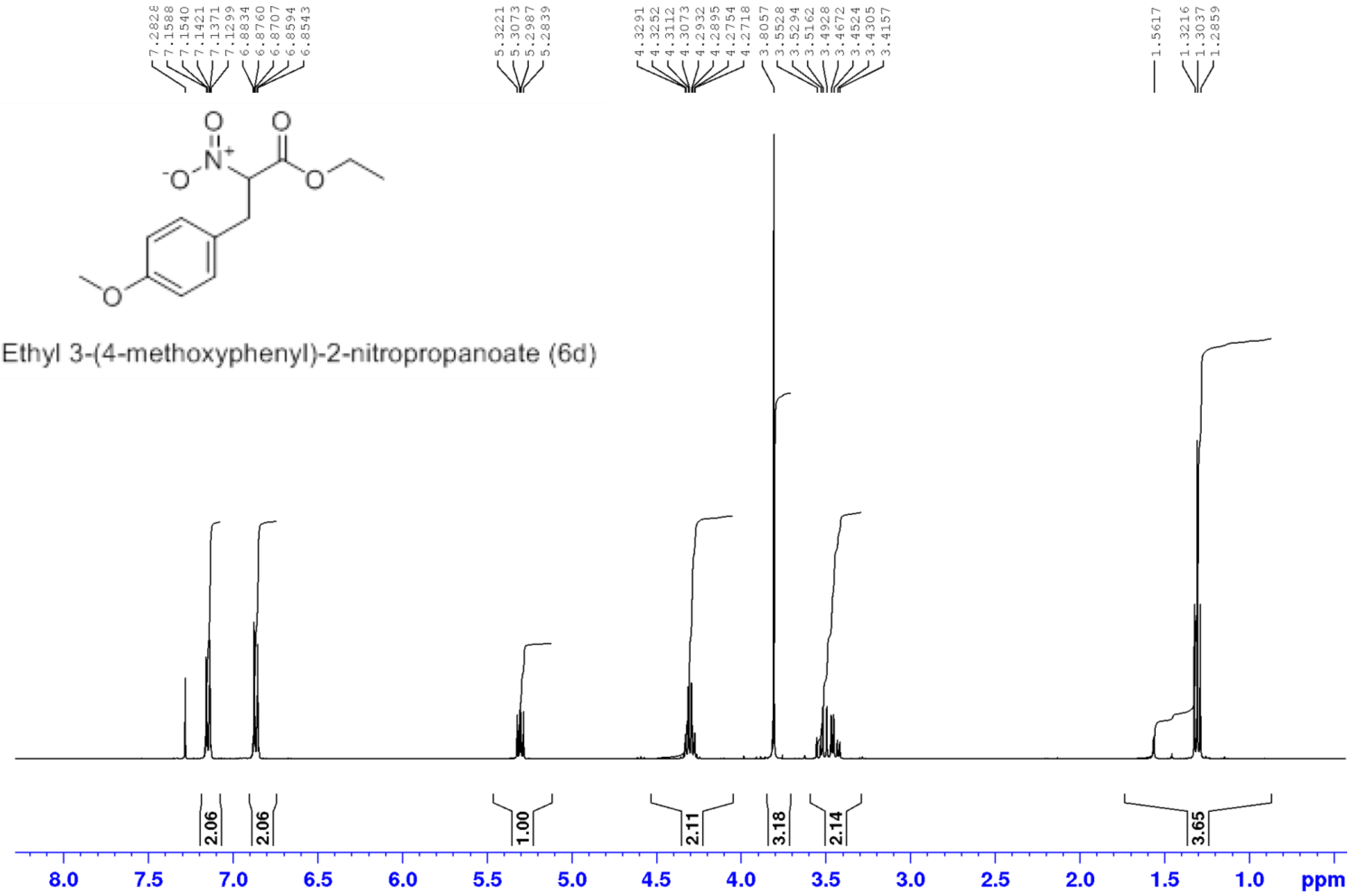

Ethyl 3-(4-methoxyphenyl)-2-nitropropanoate (6d)

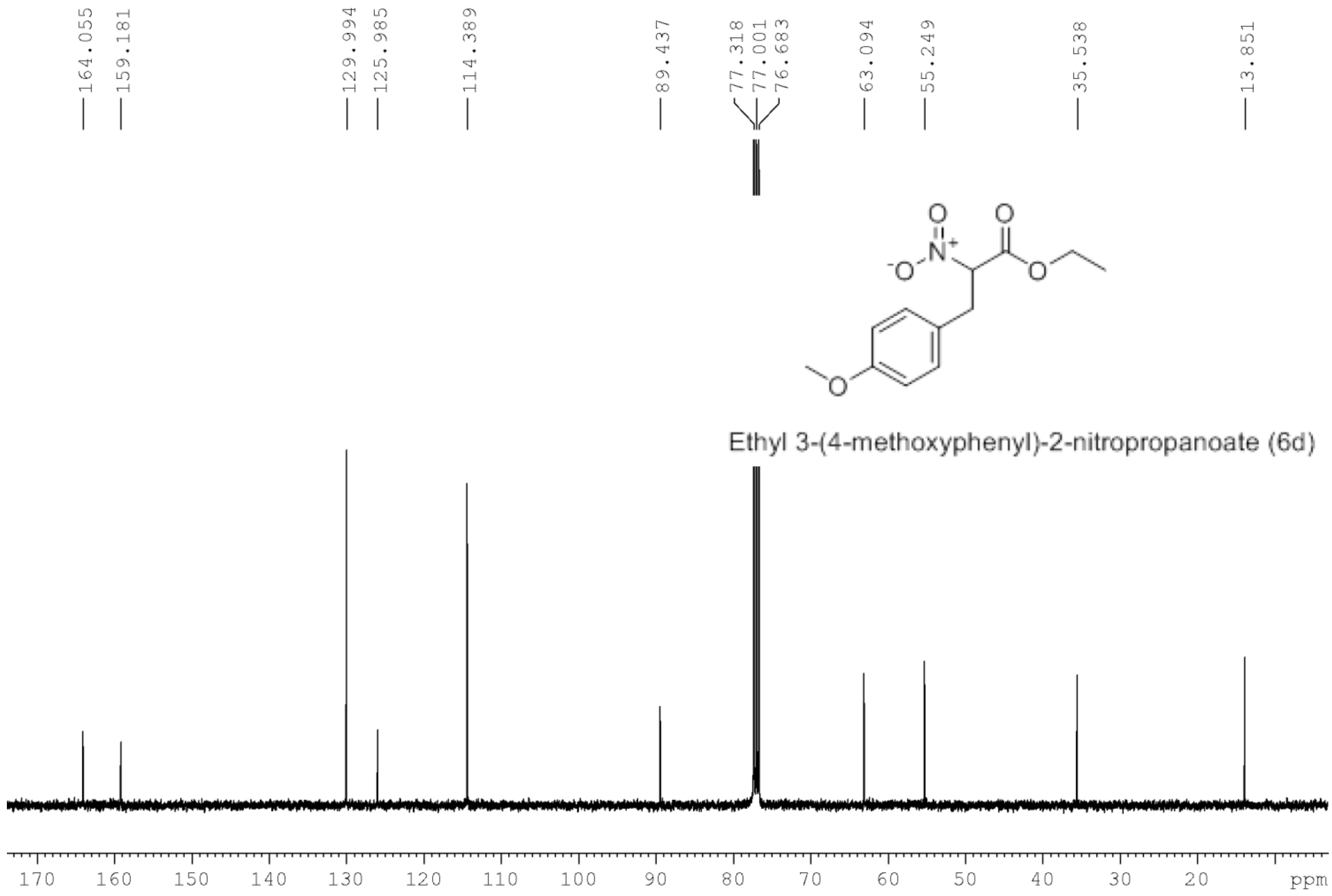

Ethyl 3-(4-methoxyphenyl)-2-nitropropanoate (6d)

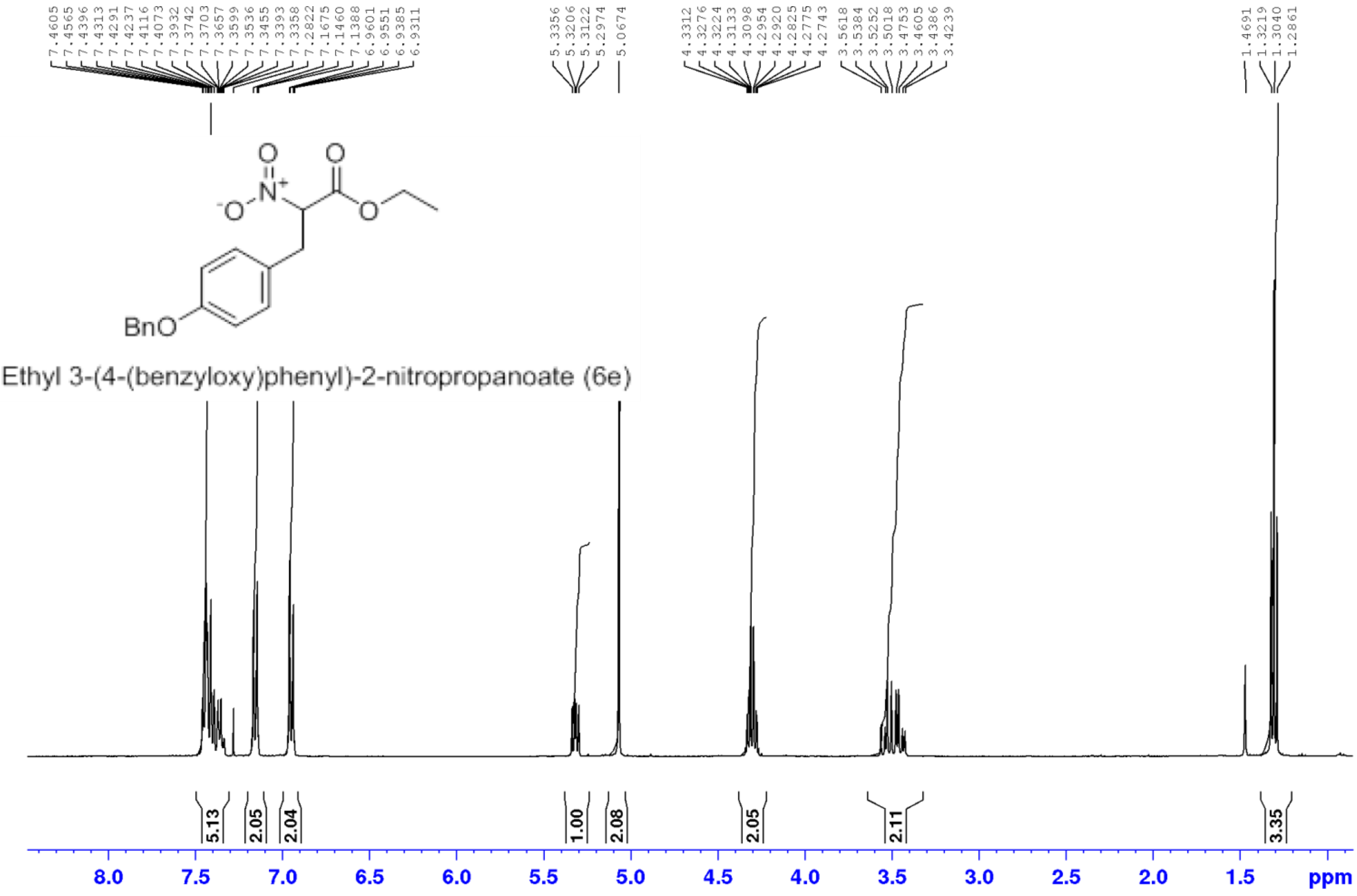
BnO
Ethyl 3-(4-(benzyloxy)phenyl)-2-nitropropanoate (6e)
5.13
2.05
2.04
1.00
2.08
2.05
2.11
3.35
8.0
7.5
7.0
6.5
6.0
5.5
5.0
4.5
4.0
3.5
3.0
2.5
2.0
1.5
ppm

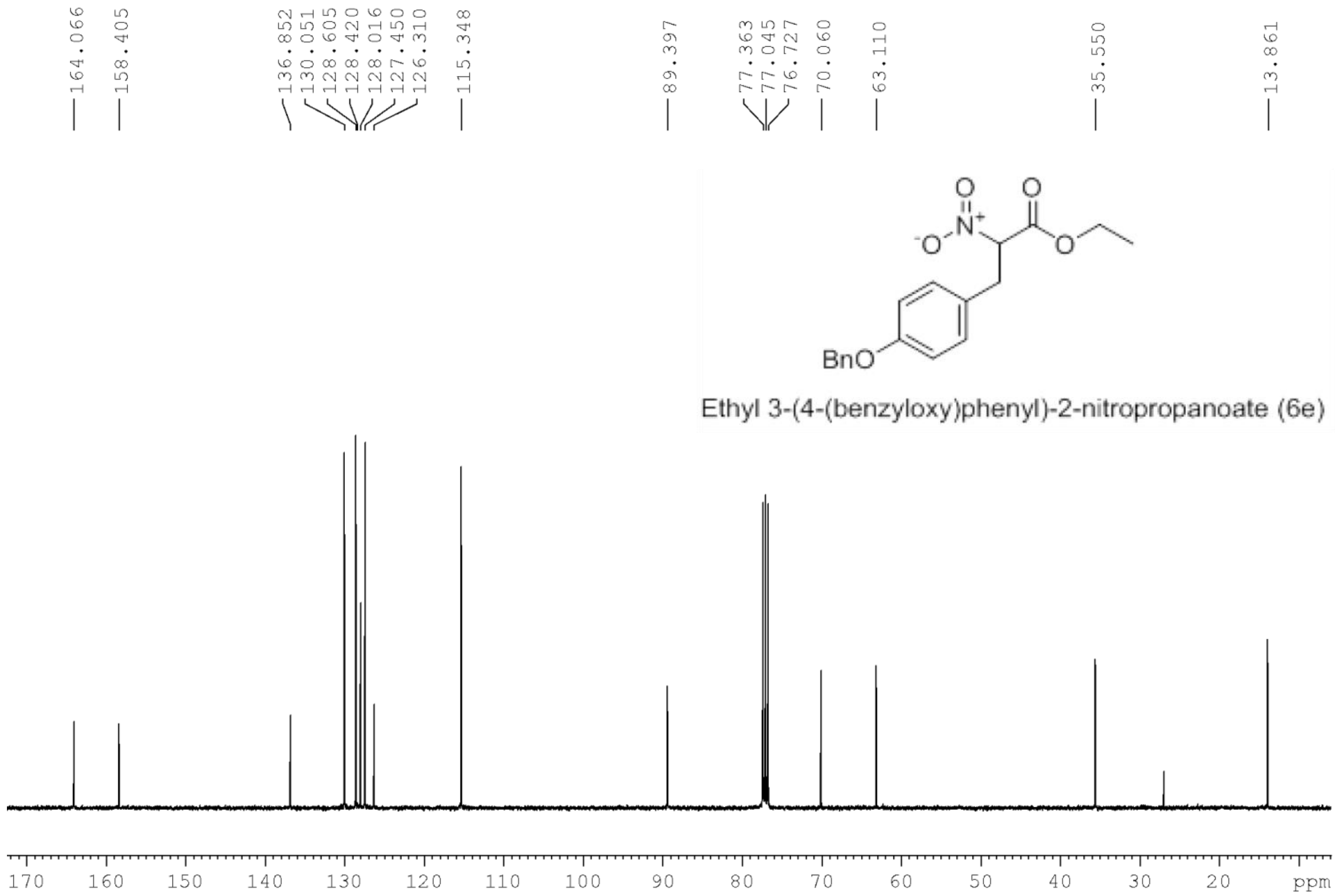

Ethyl 3-(4-(benzyloxy)phenyl)-2-nitropropanoate (6e)

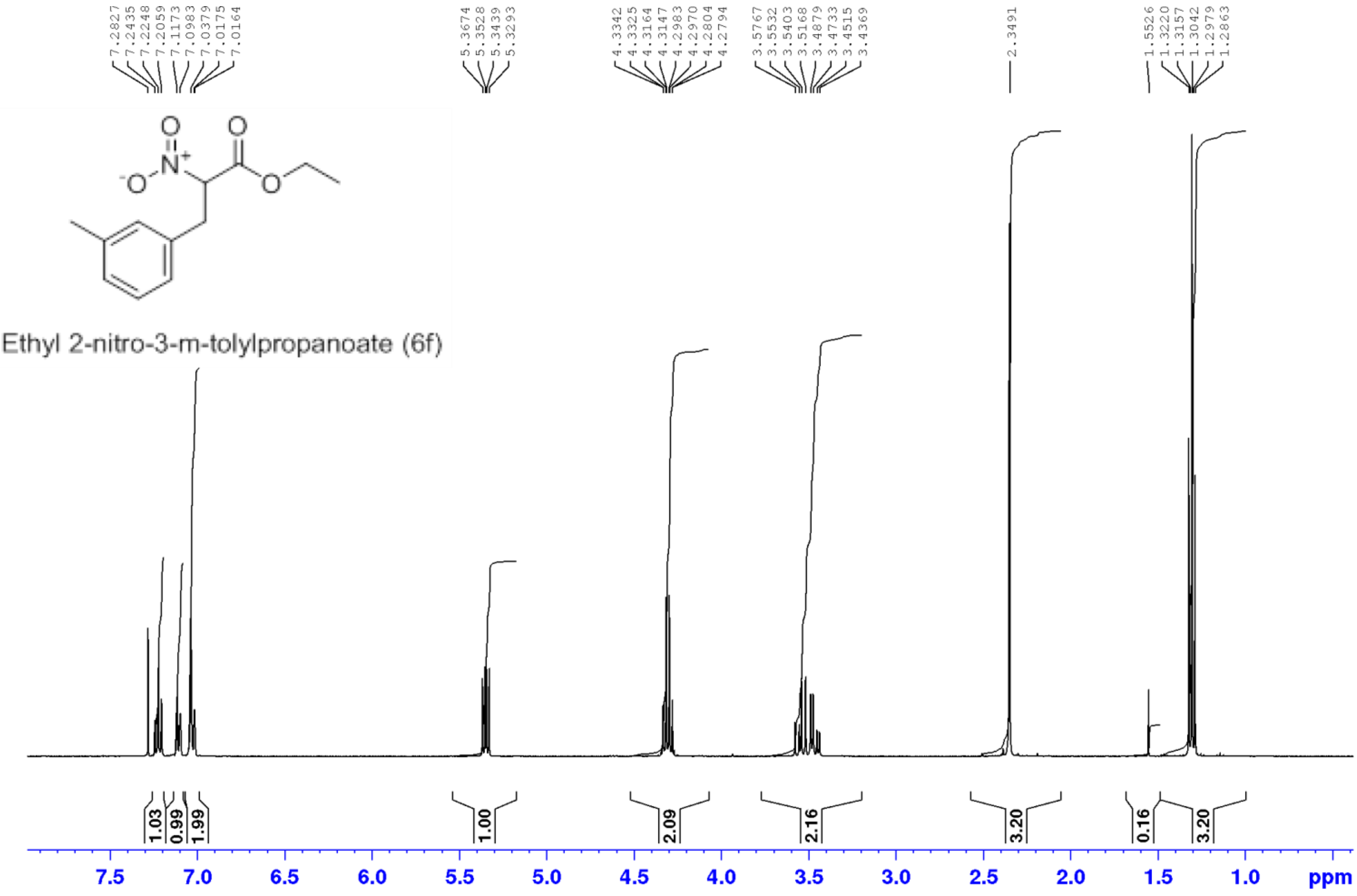

Ethyl 2-nitro-3-m-tolylpropanoate (6f)

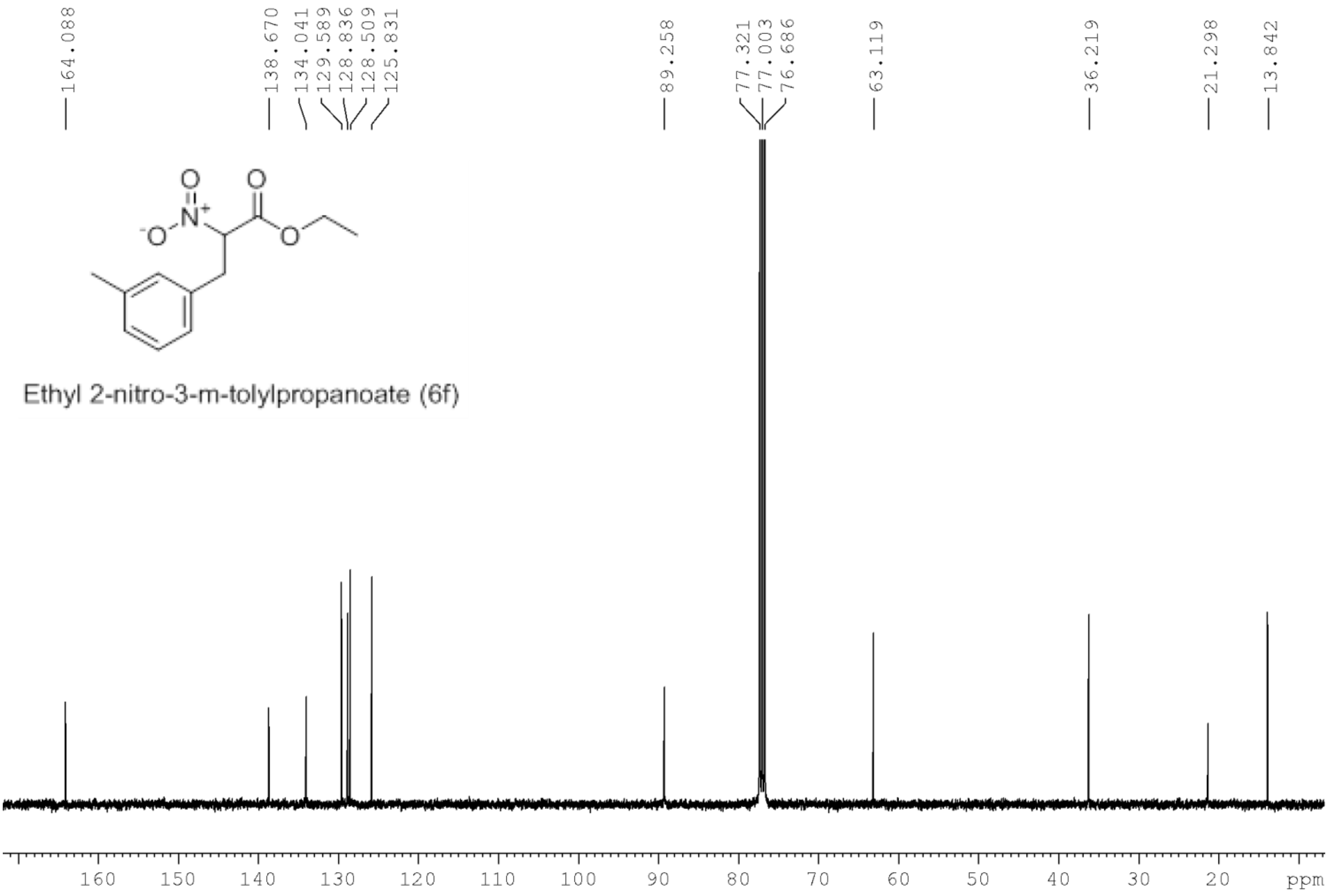

Ethyl 2-nitro-3-m-tolylpropanoate (6f)

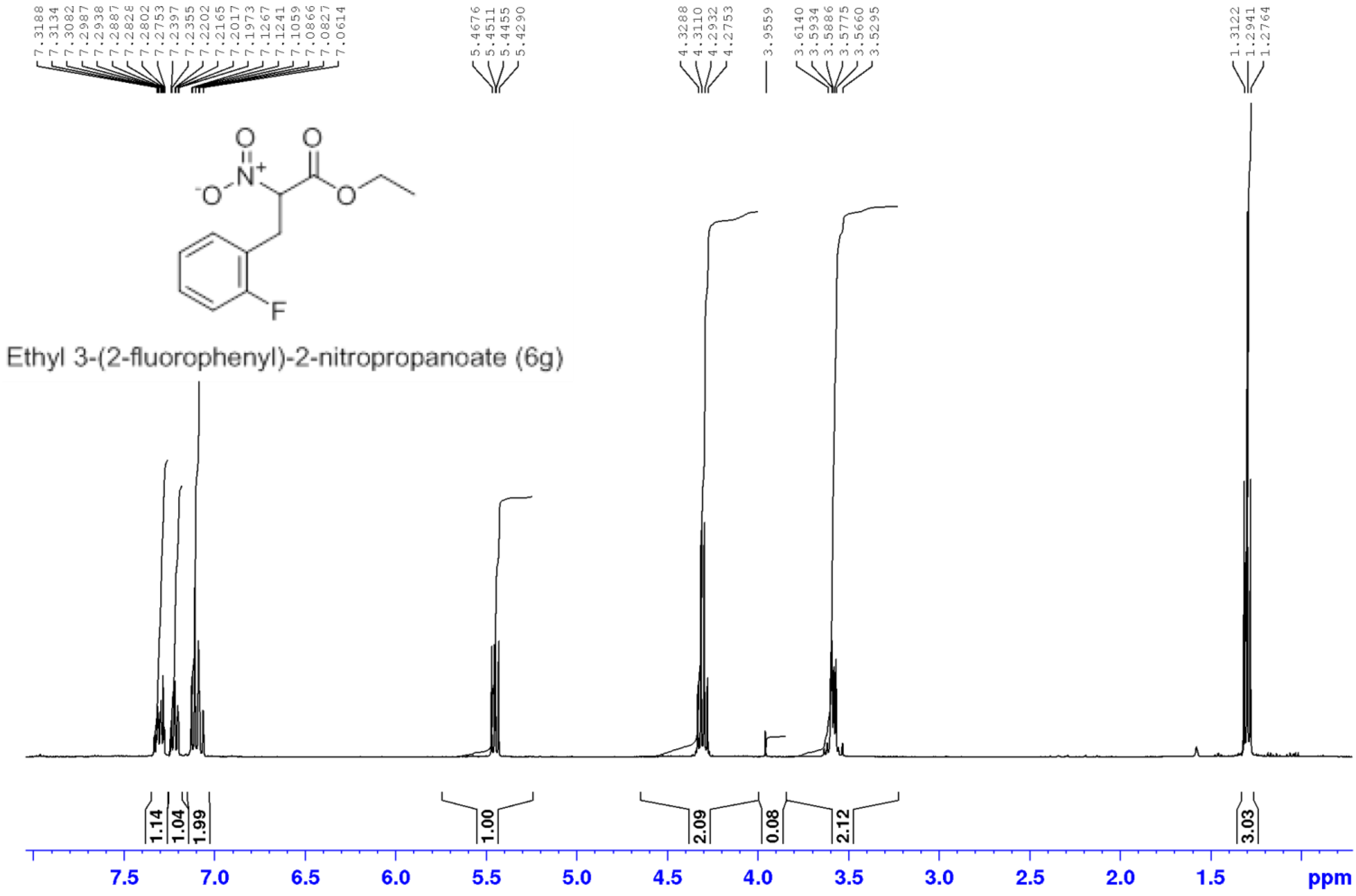

Ethyl 3-(2-fluorophenyl)-2-nitropropanoate (6g)

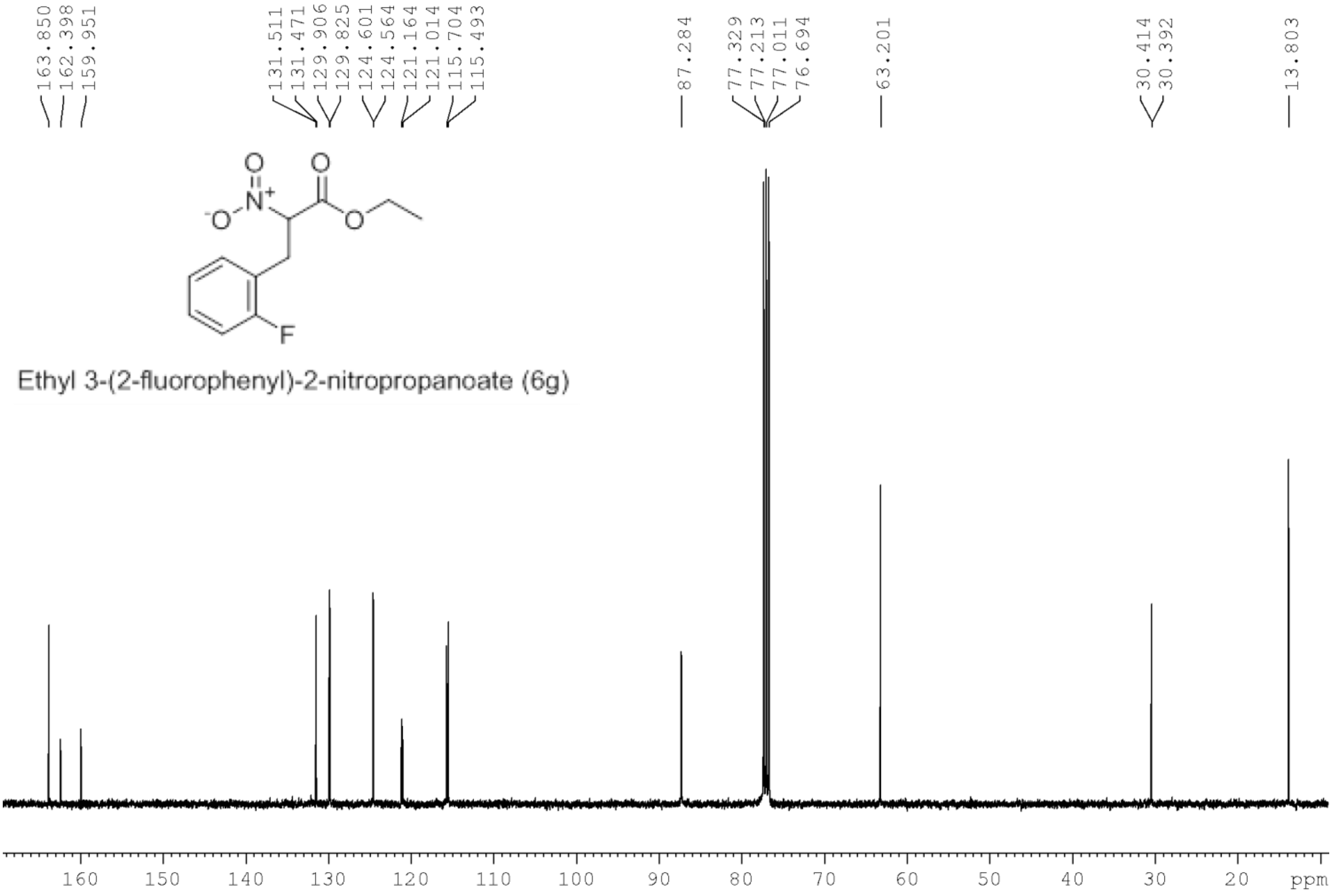

Ethyl 3-(2-fluorophenyl)-2-nitropropanoate (6g)

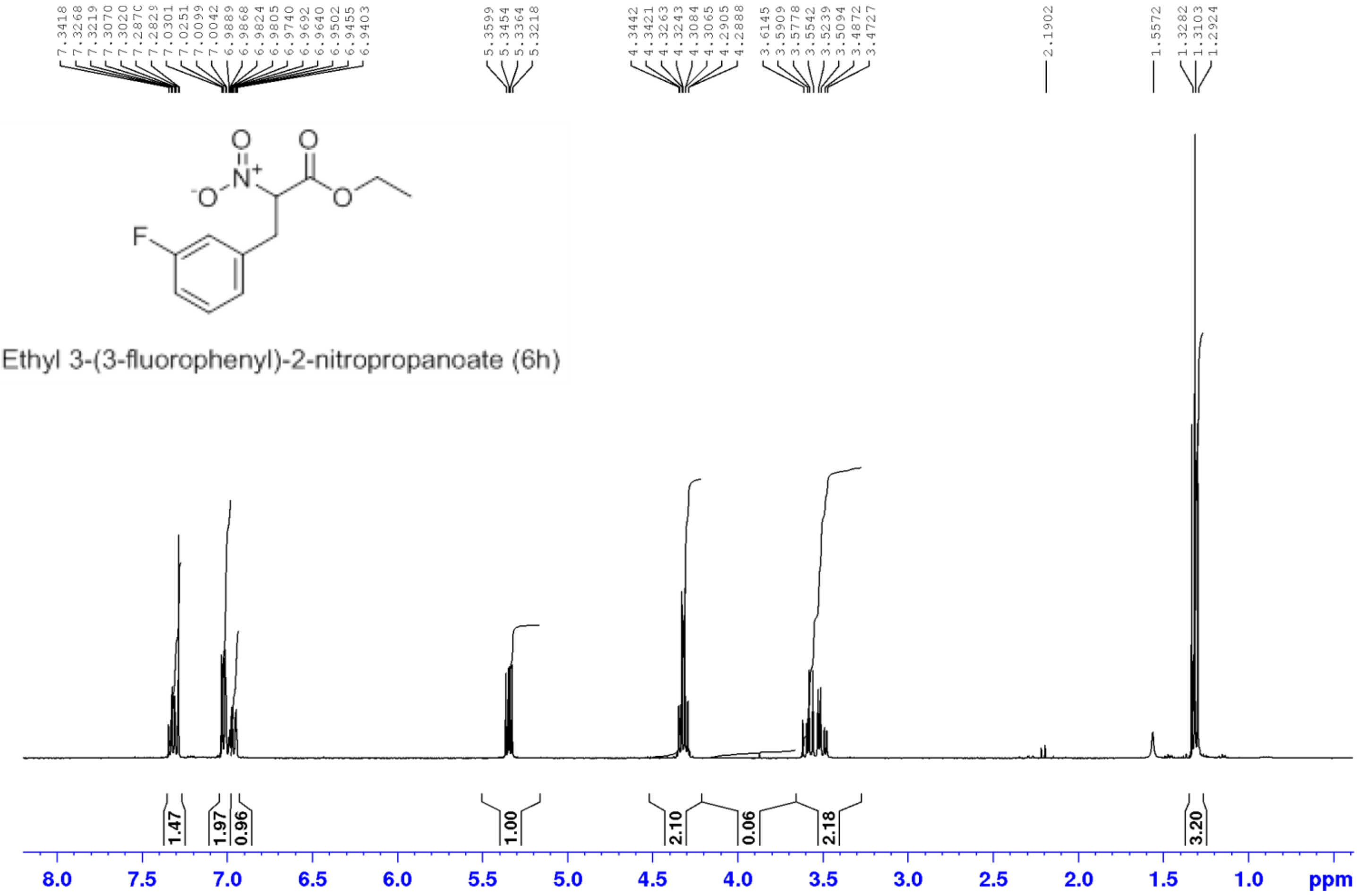
Ethyl 3-(3-fluorophenyl)-2-nitropropanoate (6h)
F
O
N+
-O
O
O
7.3418
7.3268
7.3219
7.3070
7.3020
7.2870
7.2829
7.0301
7.0251
7.0099
7.0042
6.9889
6.9868
6.9824
6.9805
6.9740
6.9692
6.9640
6.9502
6.9455
6.9403
5.3599
5.3454
5.3364
5.3218
4.3442
4.3421
4.3263
4.3243
4.3084
4.3065
4.2905
4.2888
3.6145
3.5909
3.5778
3.5542
3.5239
3.5094
3.4872
3.4727
2.1902
1.5572
1.3282
1.3103
1.2924
1.47
1.97
0.96
1.00
2.10
0.06
2.18
3.20
8.0
7.5
7.0
6.5
6.0
5.5
5.0
4.5
4.0
3.5
3.0
2.5
2.0
1.5
1.0
ppm

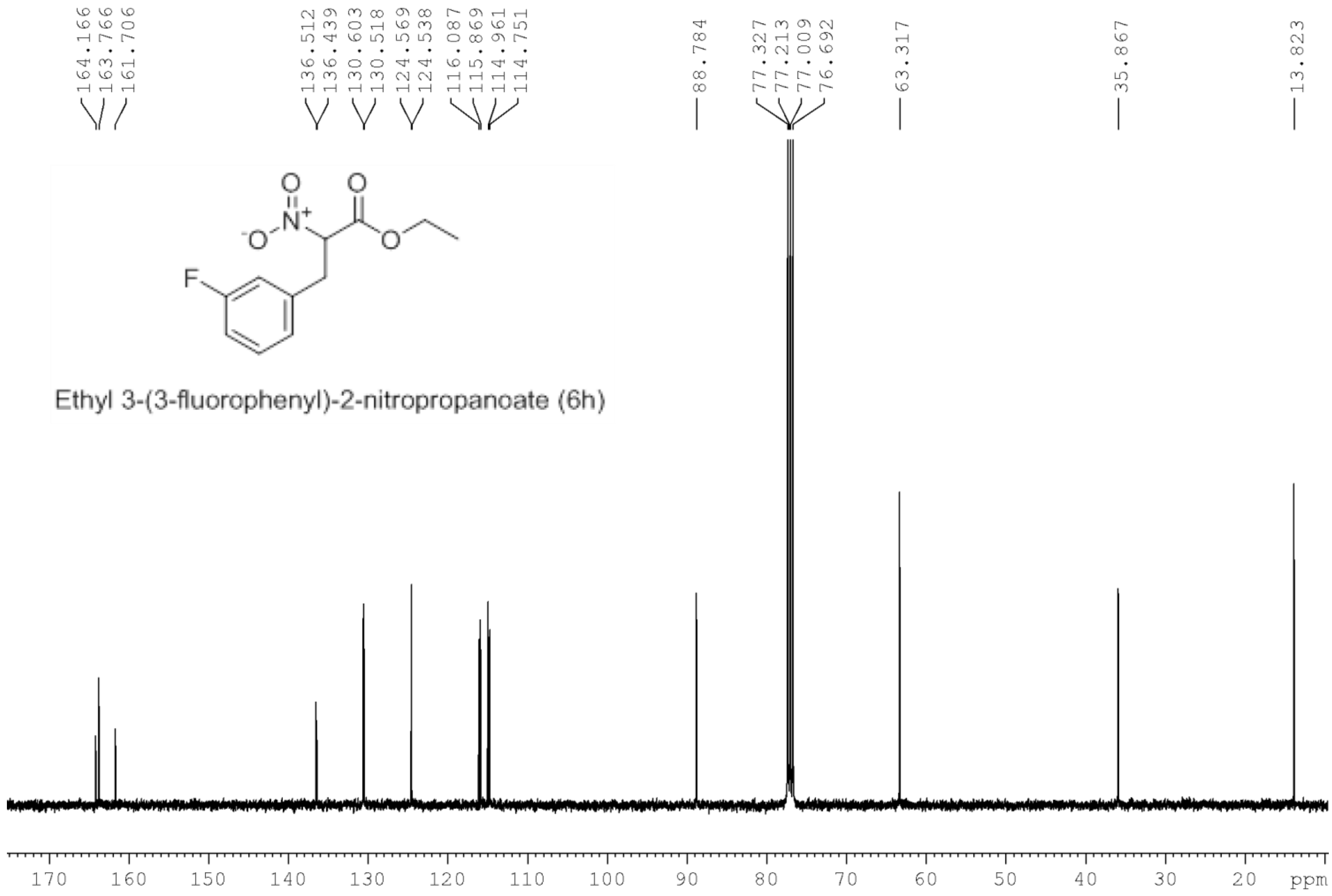
164.166
163.766
161.706
136.512
136.439
130.603
130.518
124.569
124.538
116.087
115.869
114.961
114.751
88.784
77.327
77.213
77.009
76.692
63.317
35.867
13.823
F
O
N+
-O
O
O
Ethyl 3-(3-fluorophenyl)-2-nitropropanoate (6h)
170
160
150
140
130
120
110
100
90
80
70
60
50
40
30
20
ppm

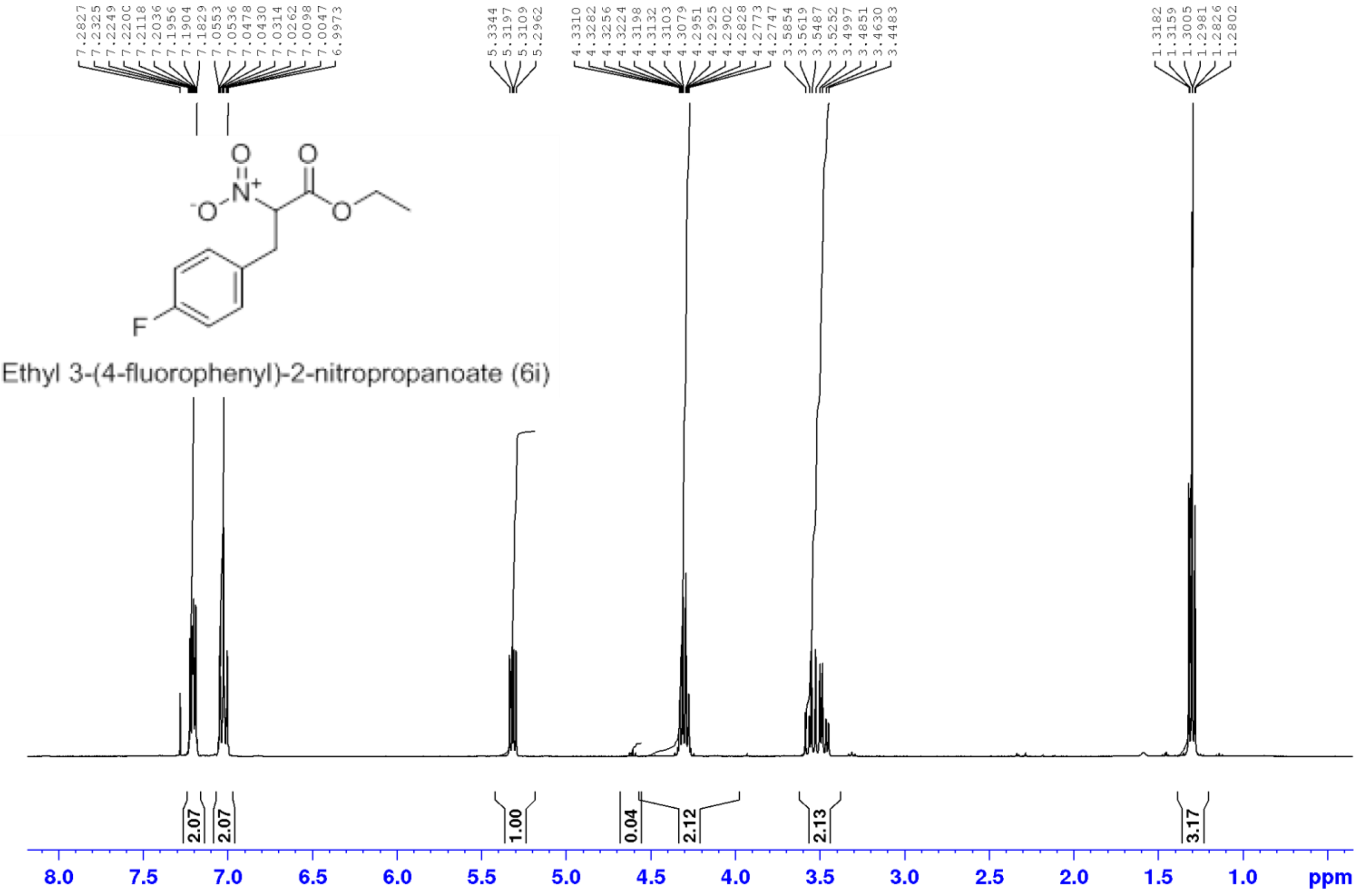
7.2827
7.2325
7.2249
7.2200
7.2118
7.2036
7.1956
7.1904
7.1829
7.0553
7.0536
7.0478
7.0430
7.0314
7.0262
7.0098
7.0047
6.9973
5.3344
5.3197
5.3109
5.2962
4.3310
4.3282
4.3256
4.3224
4.3198
4.3132
4.3103
4.3079
4.2951
4.2925
4.2902
4.2828
4.2773
4.2747
3.5854
3.5619
3.5487
3.5252
3.4997
3.4851
3.4630
3.4483
1.3182
1.3159
1.3005
1.2981
1.2826
1.2802
Ethyl 3-(4-fluorophenyl)-2-nitropropanoate (6i)
2.07
2.07
1.00
0.04
2.12
2.13
3.17
8.0
7.5
7.0
6.5
6.0
5.5
5.0
4.5
4.0
3.5
3.0
2.5
2.0
1.5
1.0
ppm

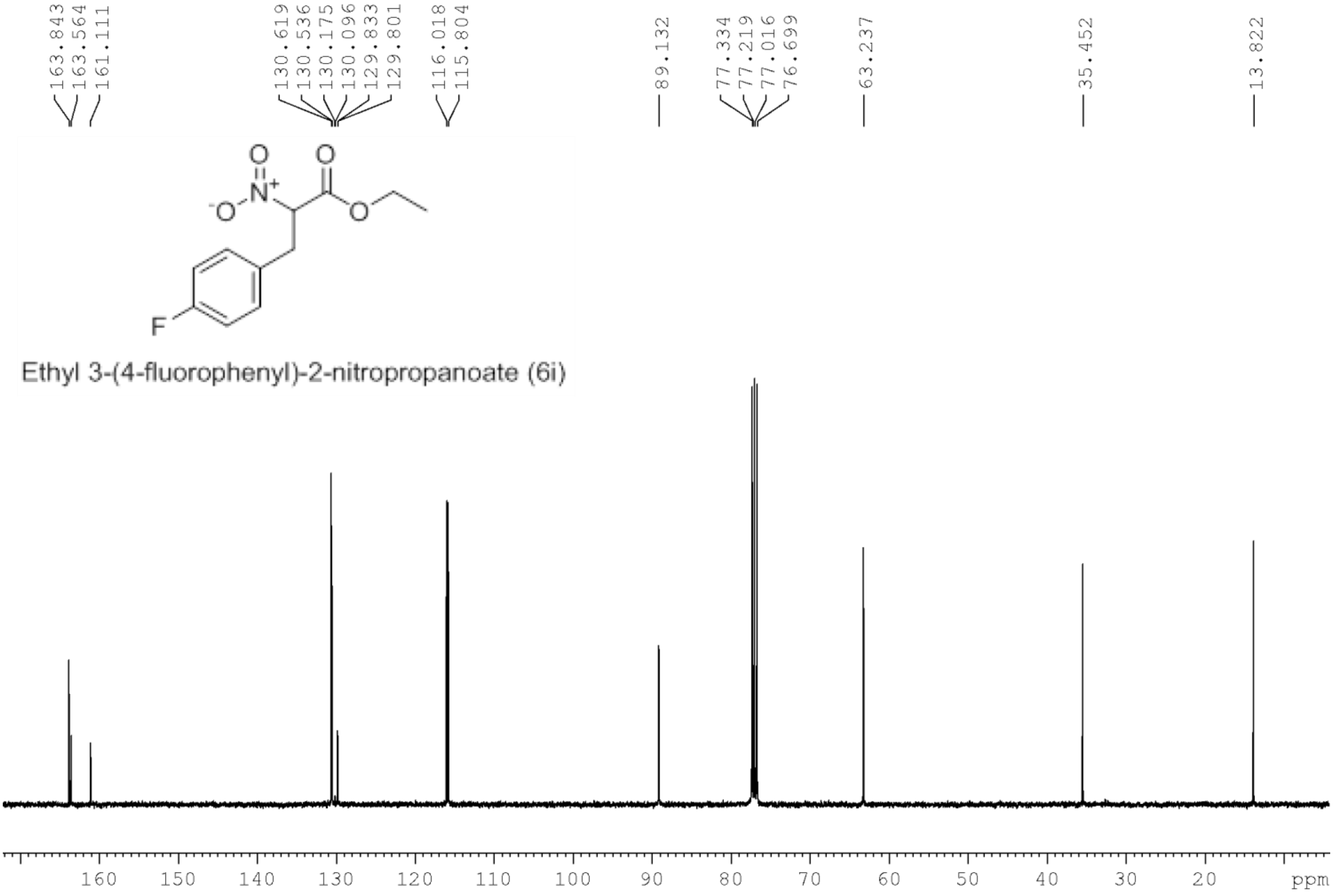

Ethyl 3-(4-fluorophenyl)-2-nitropropanoate (6i)

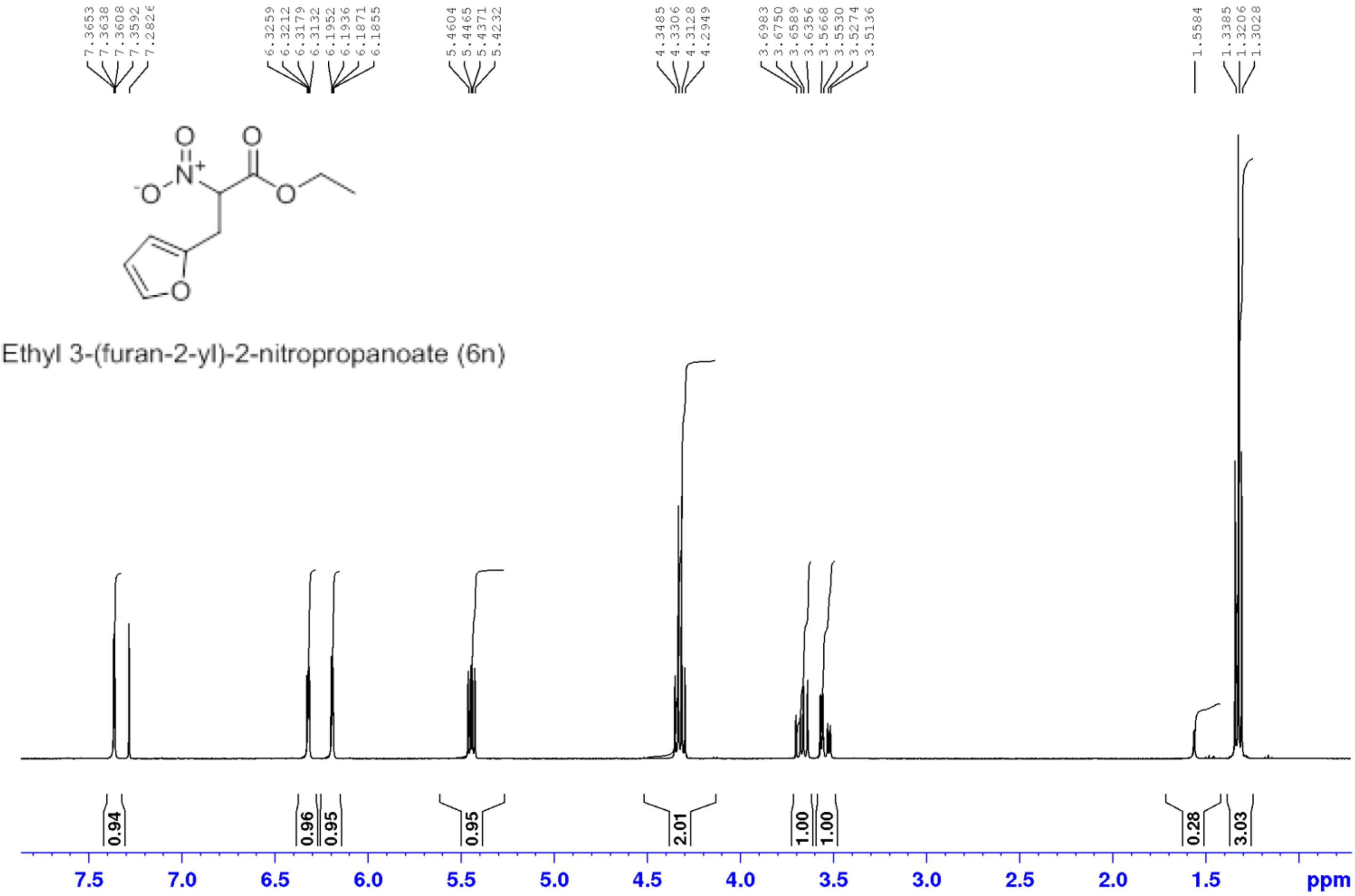

Ethyl 3-(furan-2-yl)-2-nitropropanoate (6n)

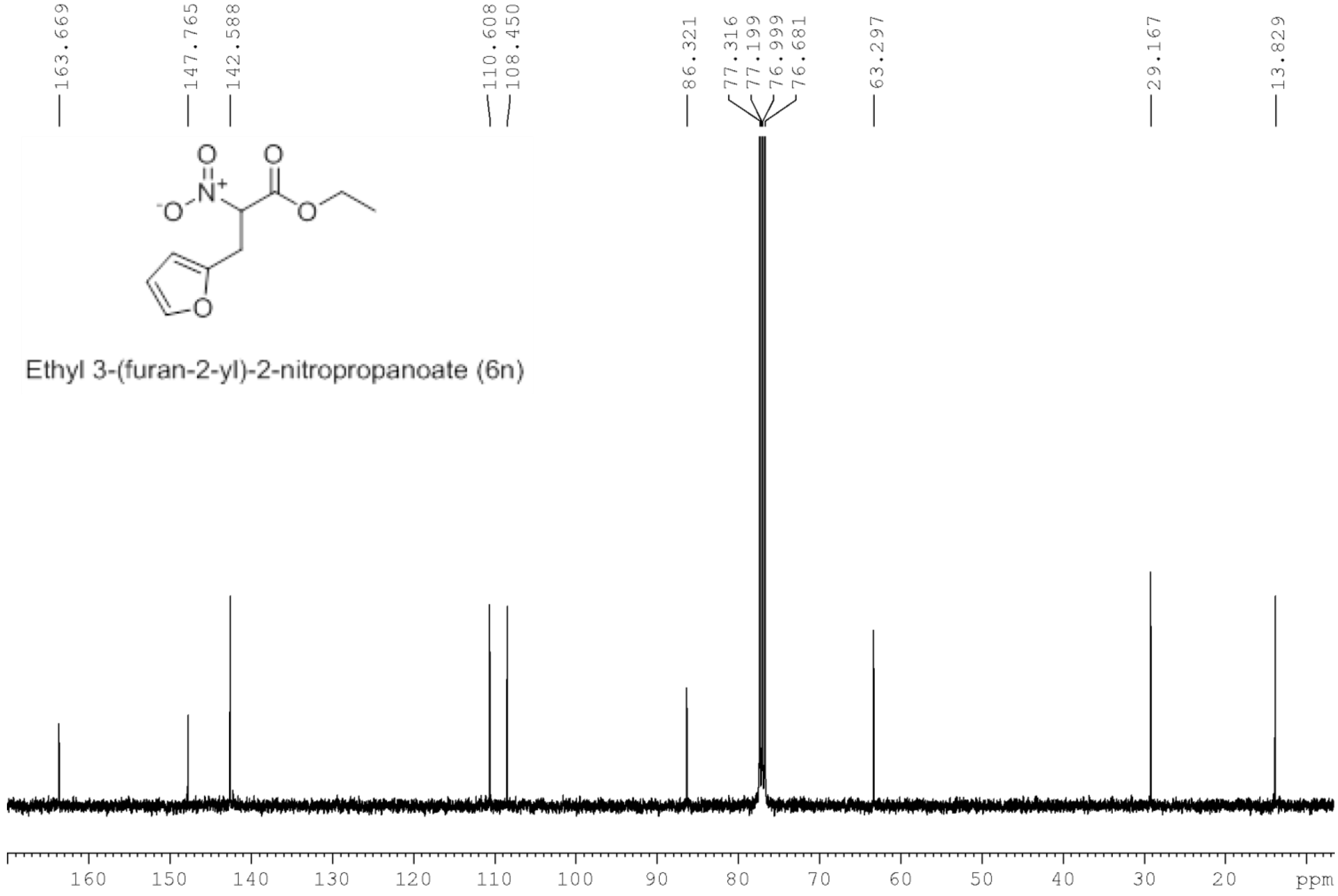
163.669
147.765
142.588
110.608
108.450
86.321
77.316
77.199
76.999
76.681
63.297
29.167
13.829
Ethyl 3-(furan-2-yl)-2-nitropropanoate (6n)
160 150 140 130 120 110 100 90 80 70 60 50 40 30 20 ppm

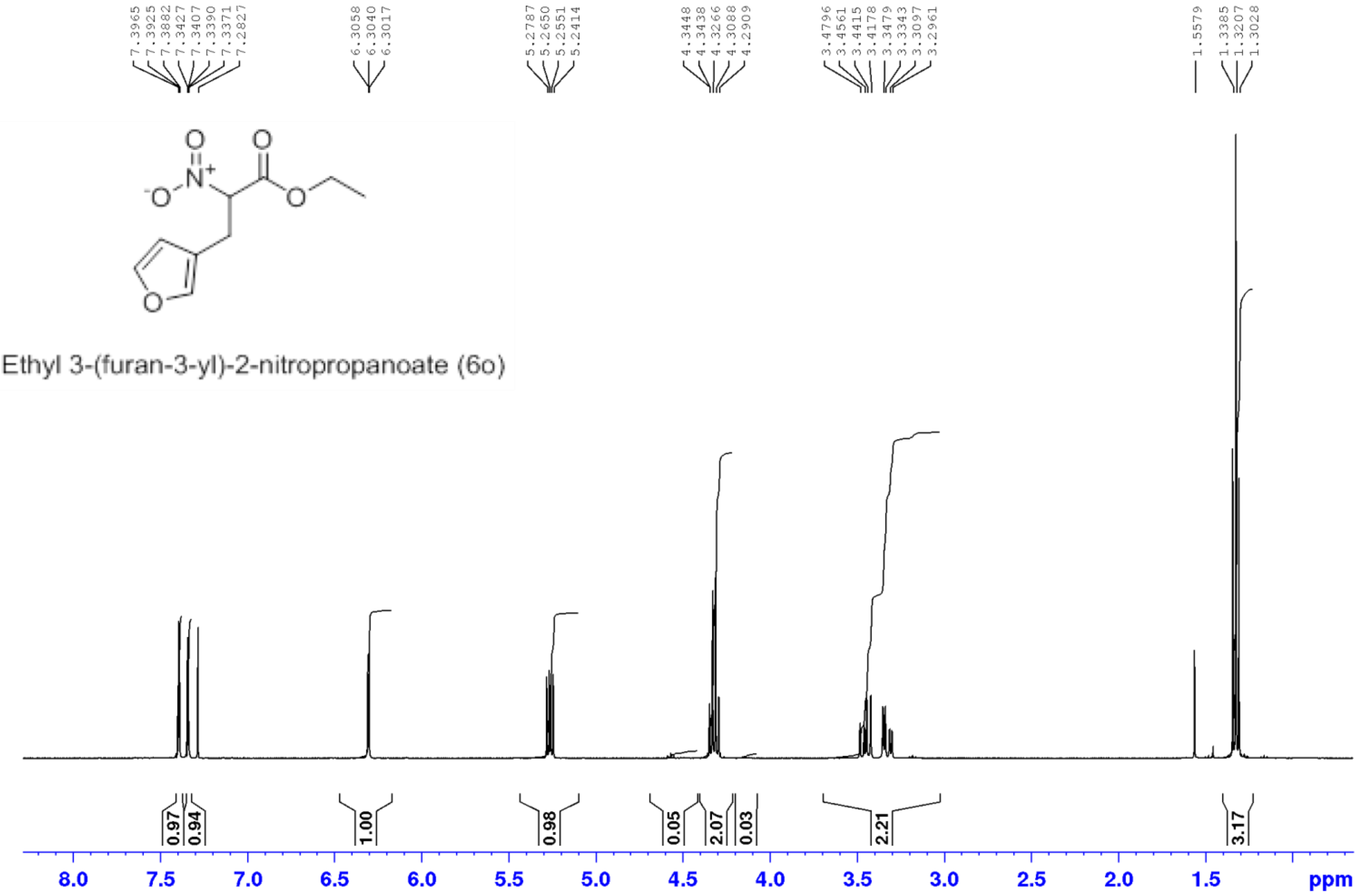
7.3965
7.3925
7.3882
7.3427
7.3407
7.3390
7.3371
7.2827
6.3058
6.3040
6.3017
5.2787
5.2650
5.2551
5.2414
4.3448
4.3438
4.3266
4.3088
4.2909
3.4796
3.4561
3.4415
3.4178
3.3479
3.3343
3.3097
3.2961
1.5579
1.3385
1.3207
1.3028
Ethyl 3-(furan-3-yl)-2-nitropropanoate (6o)
0.97
0.94
1.00
0.98
0.05
2.07
0.03
2.21
3.17
8.0
7.5
7.0
6.5
6.0
5.5
5.0
4.5
4.0
3.5
3.0
2.5
2.0
1.5
ppm

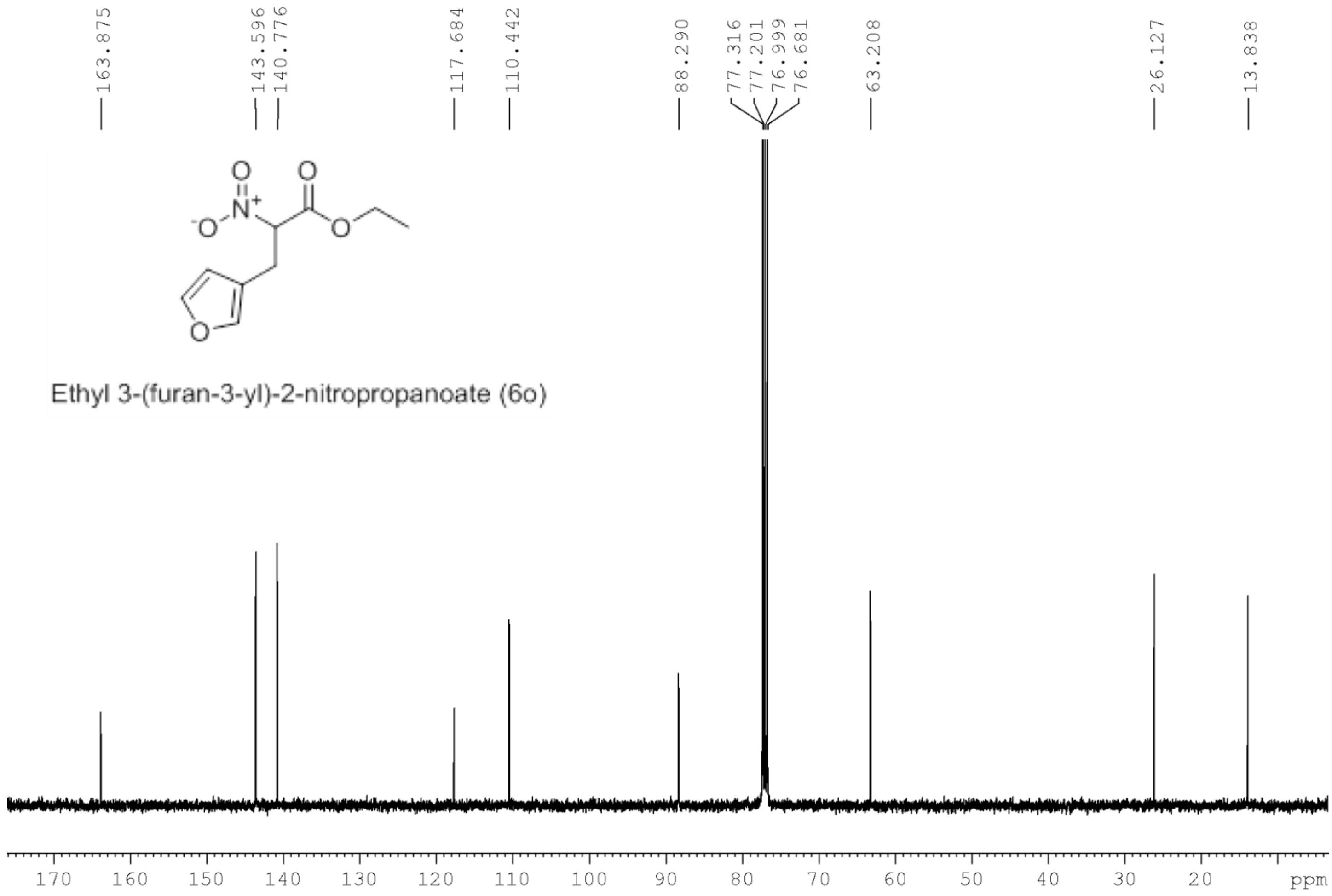
163.875
143.596
140.776
117.684
110.442
88.290
77.316
77.201
76.999
76.681
63.208
26.127
13.838
Ethyl 3-(furan-3-yl)-2-nitropropanoate (6o)
170 160 150 140 130 120 110 100 90 80 70 60 50 40 30 20 ppm

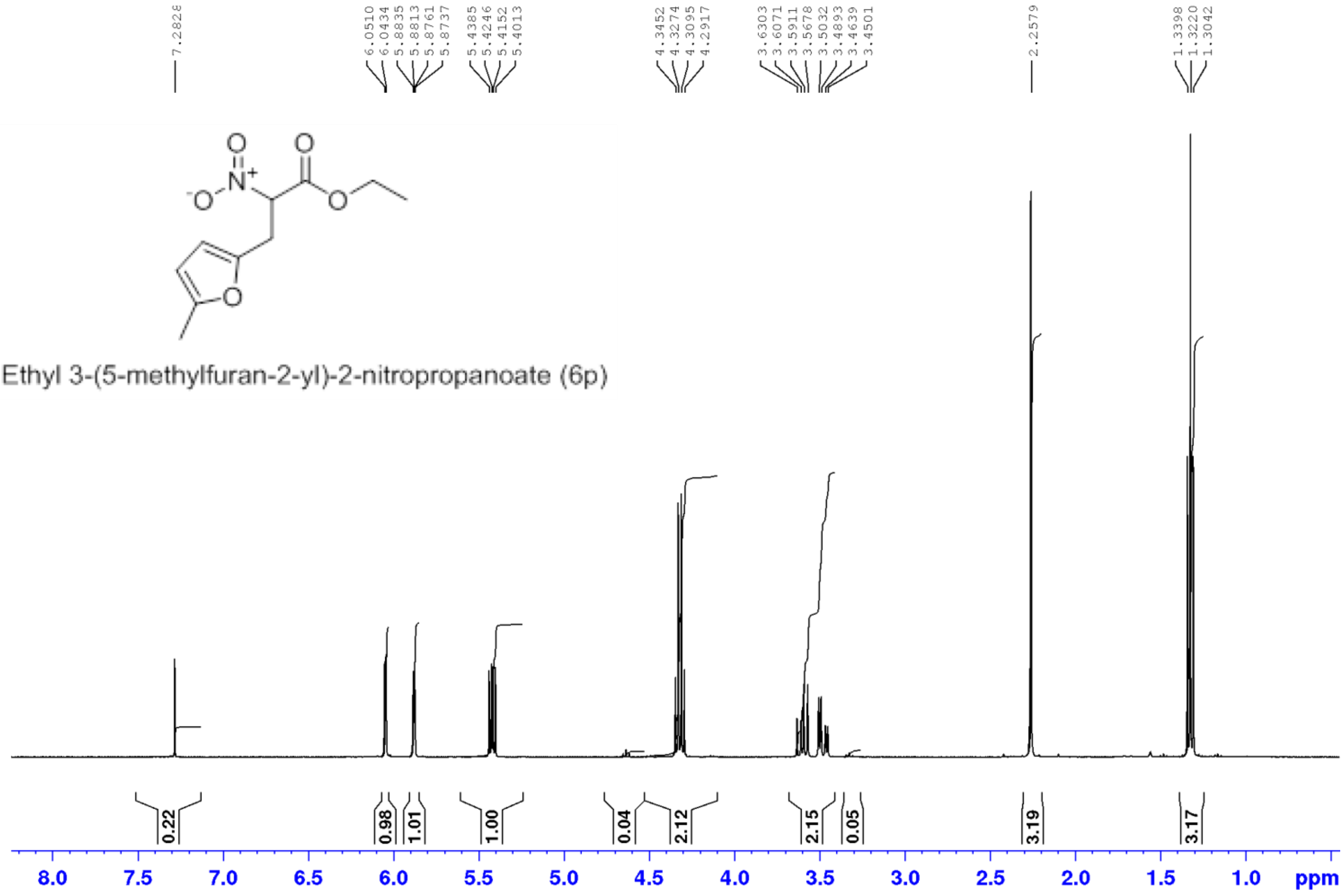
Ethyl 3-(5-methylfuran-2-yl)-2-nitropropanoate (6p)
7.2828
6.0510
6.0434
5.8835
5.8813
5.8761
5.8737
5.4385
5.4246
5.4152
5.4013
4.3452
4.3274
4.3095
4.2917
3.6303
3.6071
3.5911
3.5678
3.5032
3.4893
3.4639
3.4501
2.2579
1.3398
1.3220
1.3042
0.22
0.98
1.01
1.00
0.04
2.12
2.15
0.05
3.19
3.17
8.0 7.5 7.0 6.5 6.0 5.5 5.0 4.5 4.0 3.5 3.0 2.5 2.0 1.5 1.0 ppm

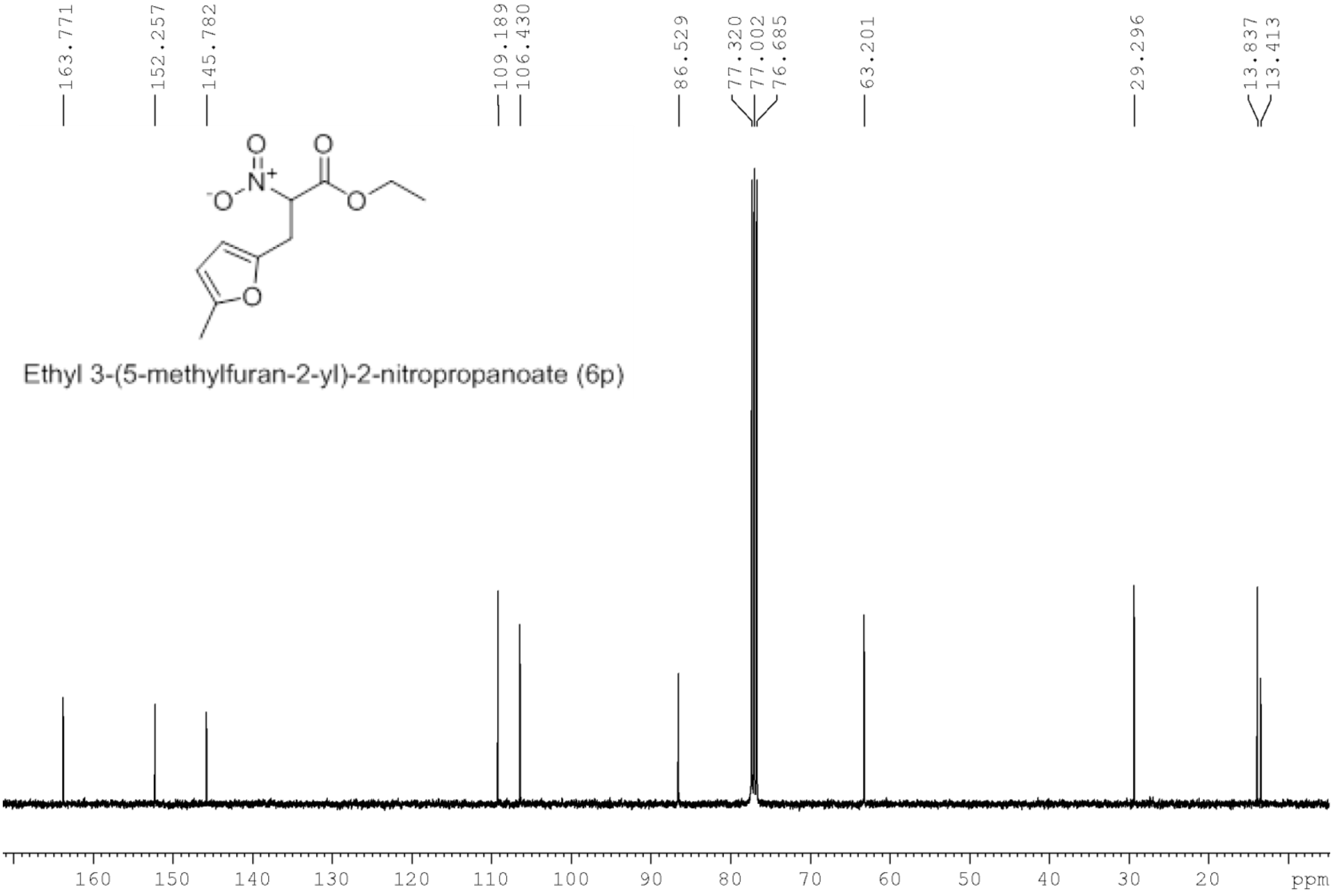
163.771
152.257
145.782
109.189
106.430
86.529
77.320
77.002
76.685
63.201
29.296
13.837
13.413
Ethyl 3-(5-methylfuran-2-yl)-2-nitropropanoate (6p)
160
150
140
130
120
110
100
90
80
70
60
50
40
30
20
ppm

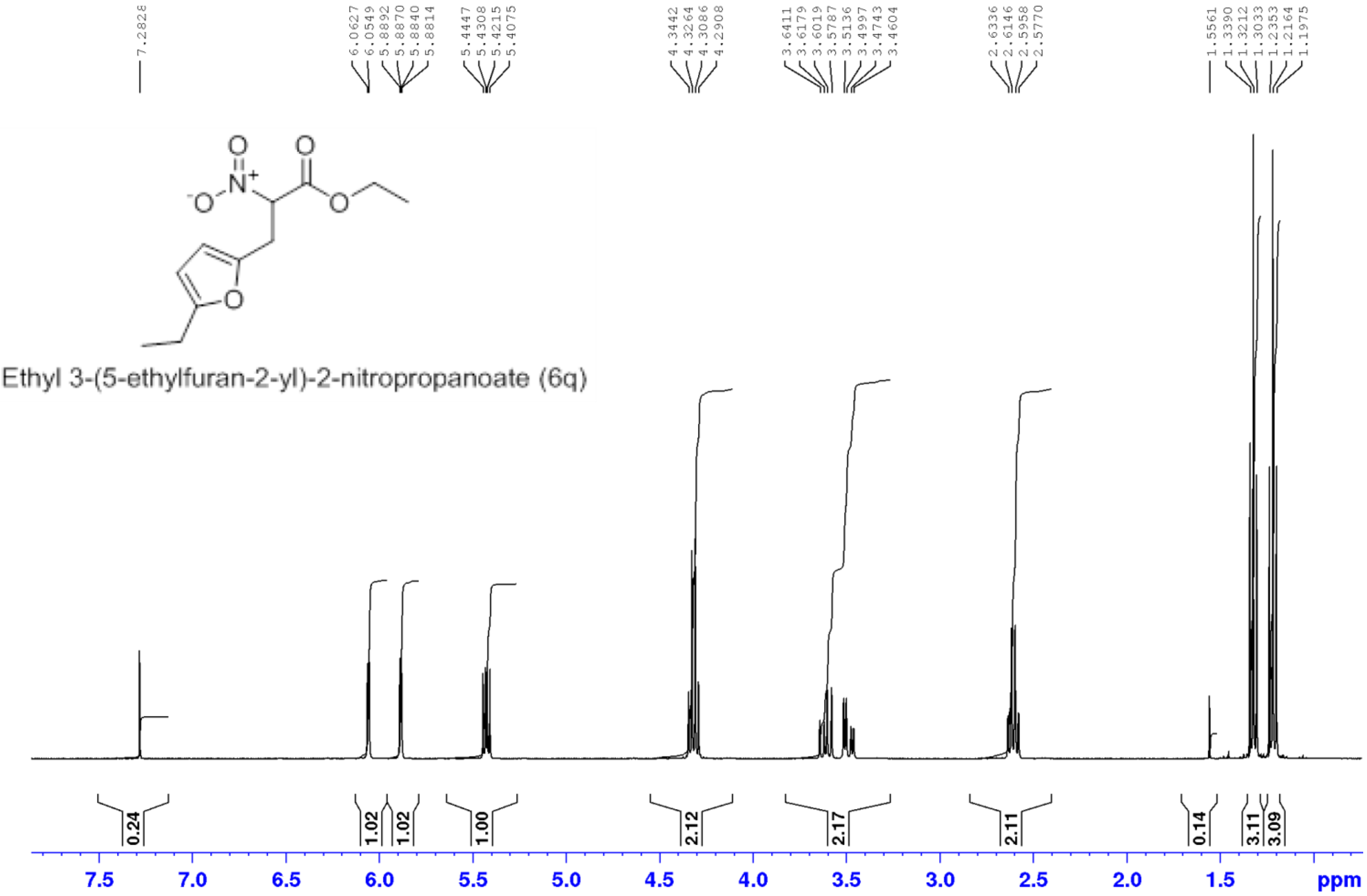

Ethyl 3-(5-ethylfuran-2-yl)-2-nitropropanoate (6q)

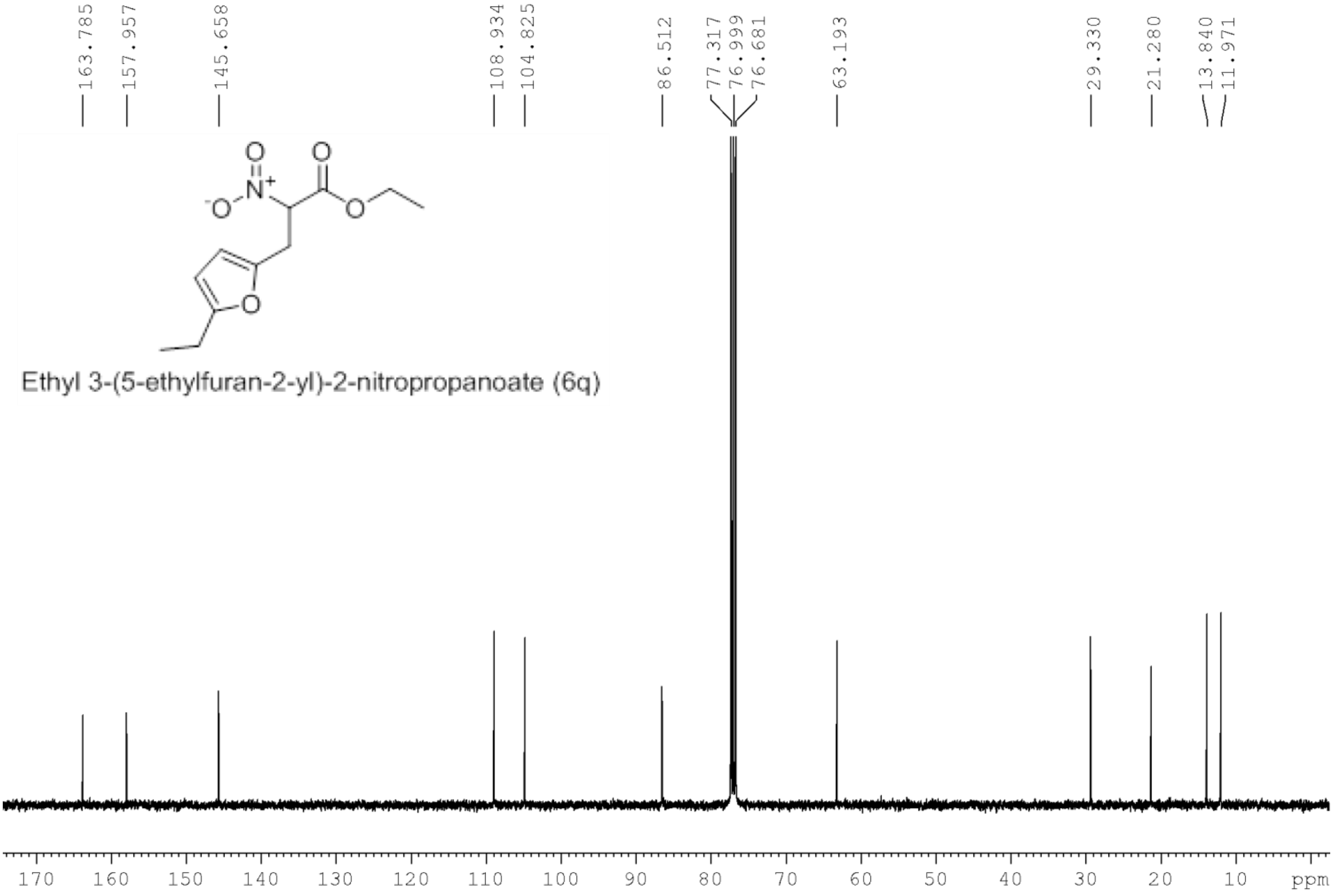
163.785
157.957
145.658
108.934
104.825
86.512
77.317
76.999
76.681
63.193
29.330
21.280
13.840
11.971
Ethyl 3-(5-ethylfuran-2-yl)-2-nitropropanoate (6q)
170 160 150 140 130 120 110 100 90 80 70 60 50 40 30 20 10 ppm

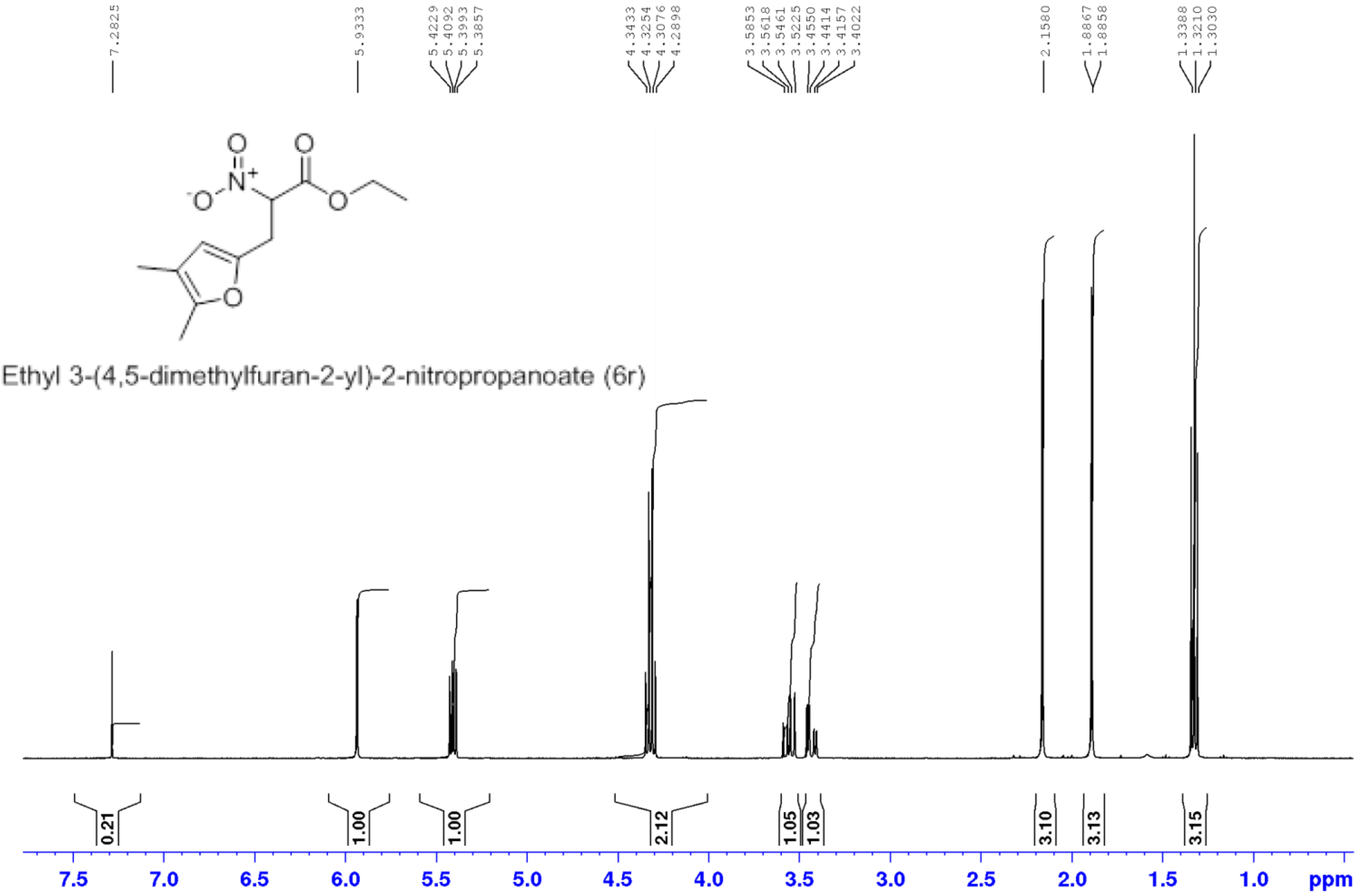

Ethyl 3-(4,5-dimethylfuran-2-yl)-2-nitropropanoate (6r)

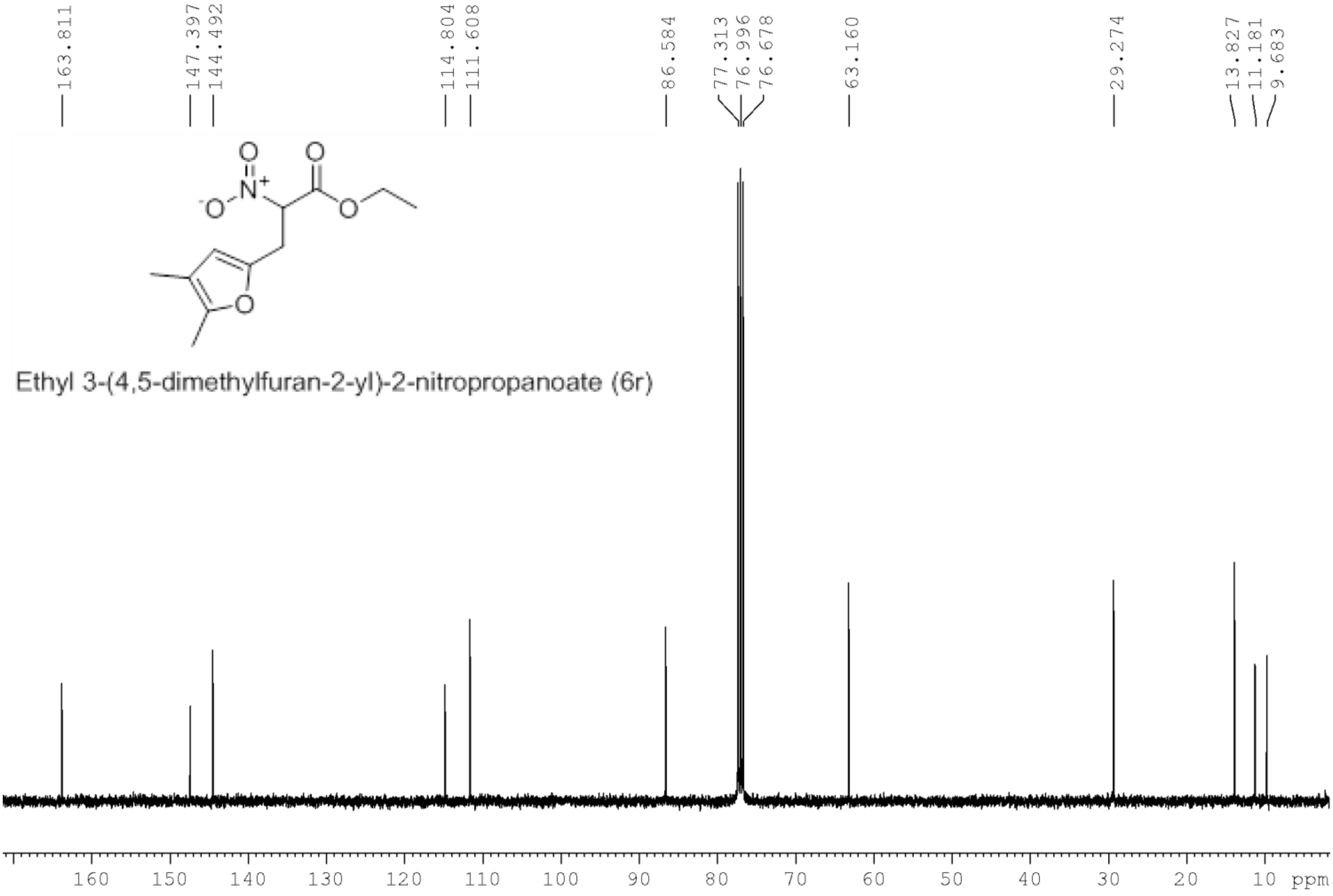

Ethyl 3-(4,5-dimethylfuran-2-yl)-2-nitropropanoate (6r)

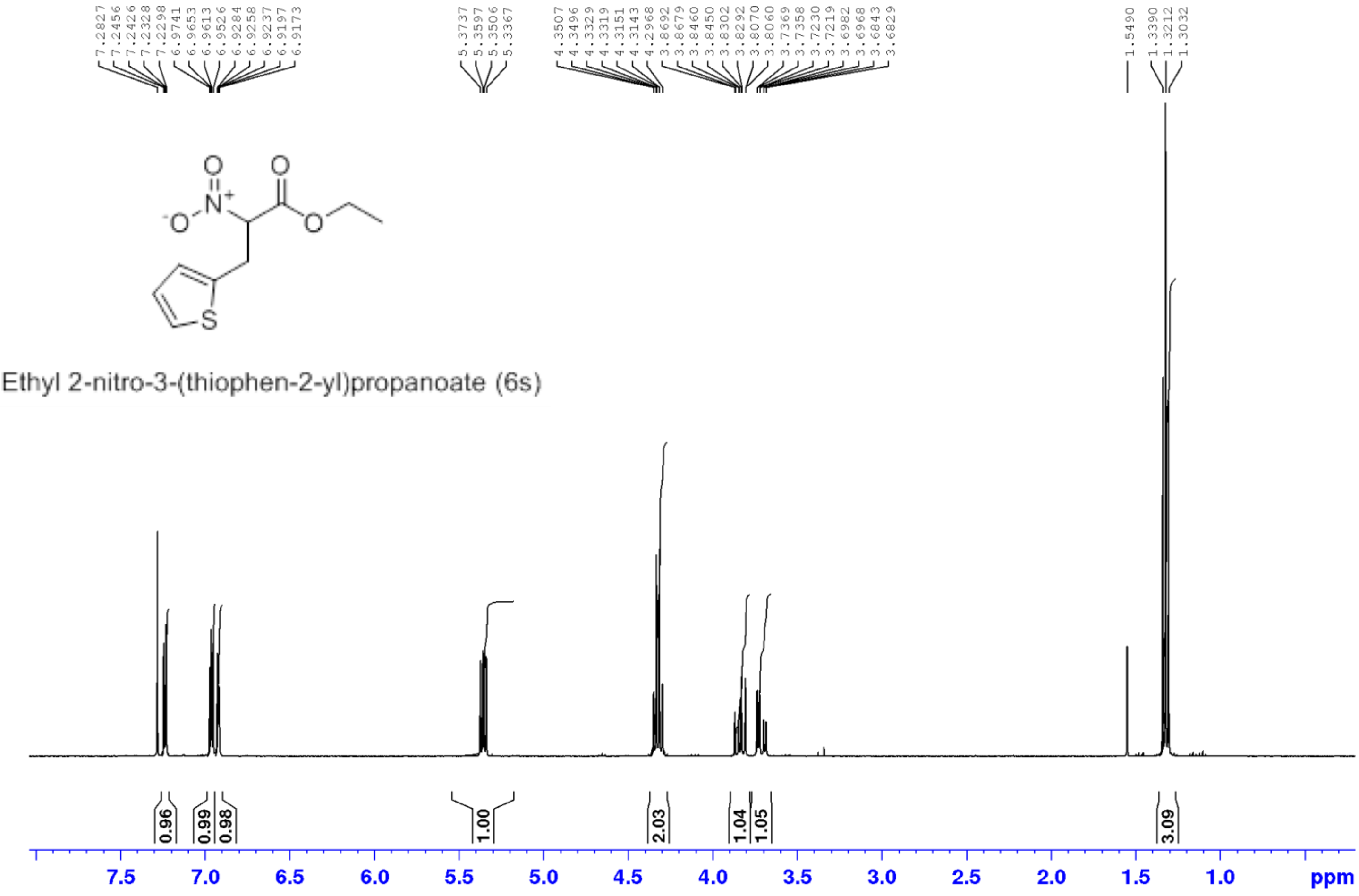

Ethyl 2-nitro-3-(thiophen-2-yl)propanoate (6s)

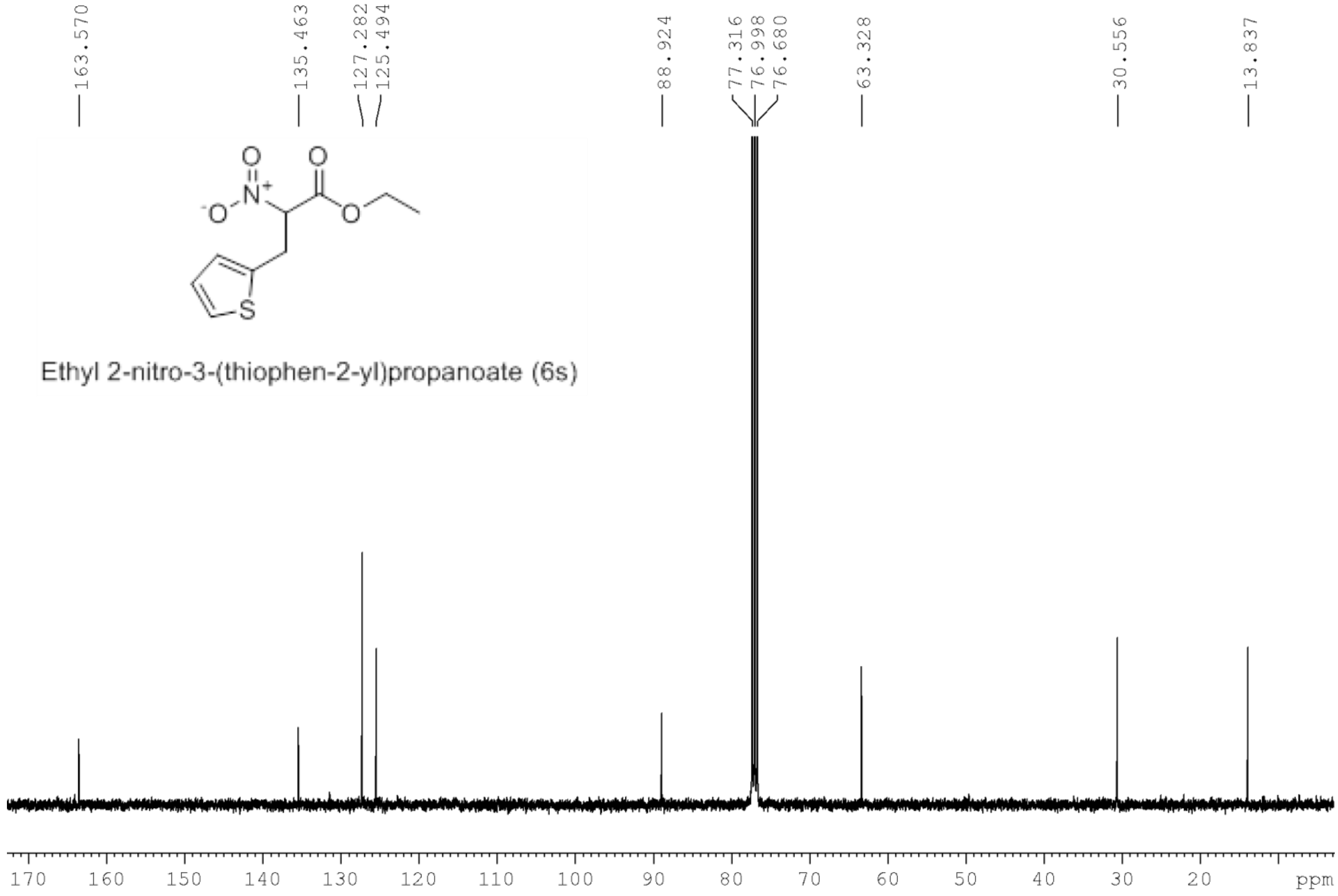

163.570
135.463
127.282
125.494
88.924
77.316
76.998
76.680
63.328
30.556
13.837
Ethyl 2-nitro-3-(thiophen-2-yl)propanoate (6s)
170
160
150
140
130
120
110
100
90
80
70
60
50
40
30
20
ppm

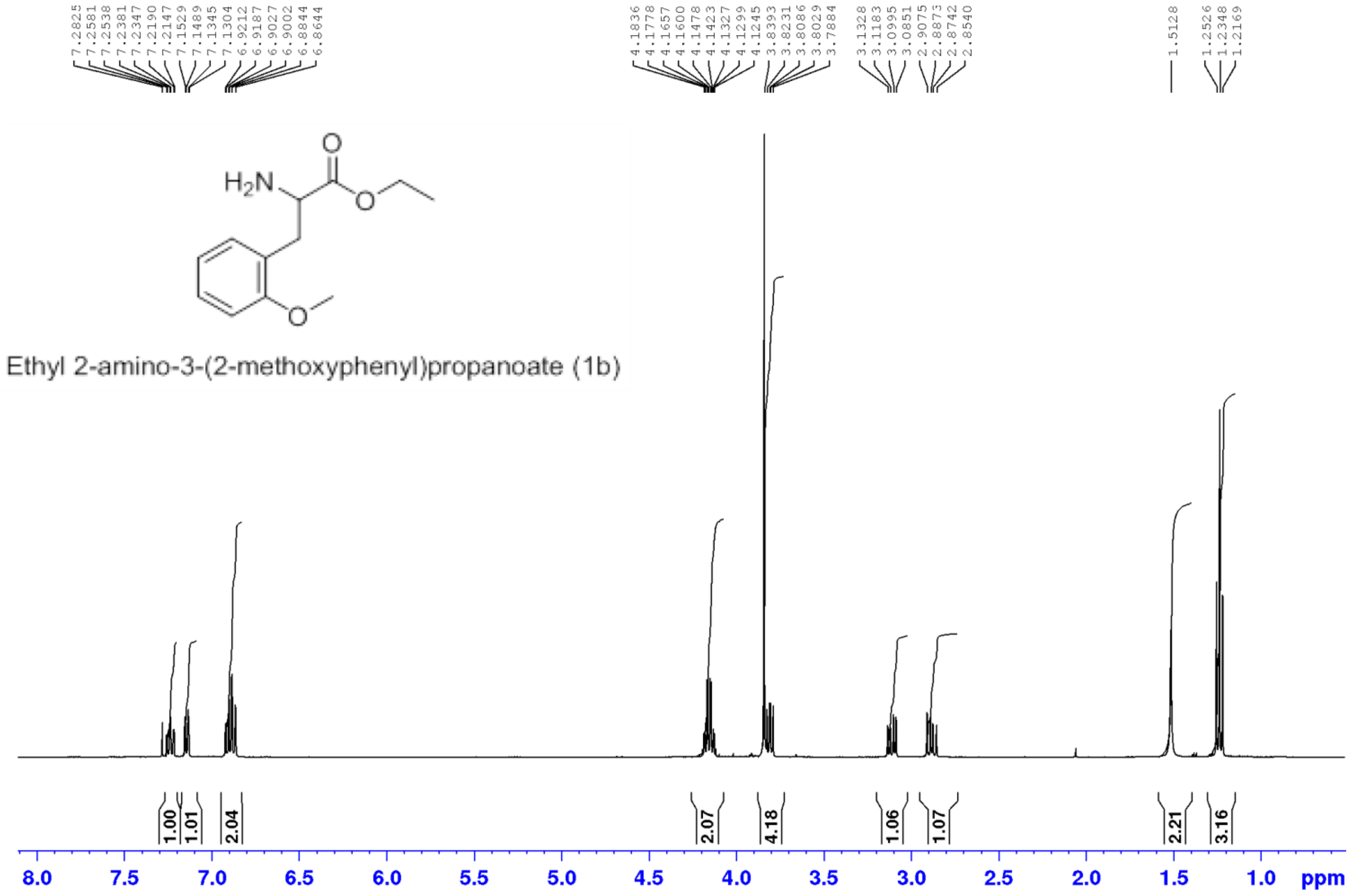

7.2825
7.2581
7.2538
7.2381
7.2347
7.2190
7.2147
7.1529
7.1489
7.1345
7.1304
6.9212
6.9187
6.9027
6.9002
6.8844
6.8644
4.1836
4.1778
4.1657
4.1600
4.1478
4.1423
4.1327
4.1299
4.1245
3.8393
3.8231
3.8086
3.8029
3.7884
3.1328
3.1183
3.0995
3.0851
2.9075
2.8873
2.8742
2.8540
1.5128
1.2526
1.2348
1.2169
Ethyl 2-amino-3-(2-methoxyphenyl)propanoate (1b)
1.00
1.01
2.04
2.07
4.18
1.06
1.07
2.21
3.16
8.0
7.5
7.0
6.5
6.0
5.5
5.0
4.5
4.0
3.5
3.0
2.5
2.0
1.5
1.0
ppm

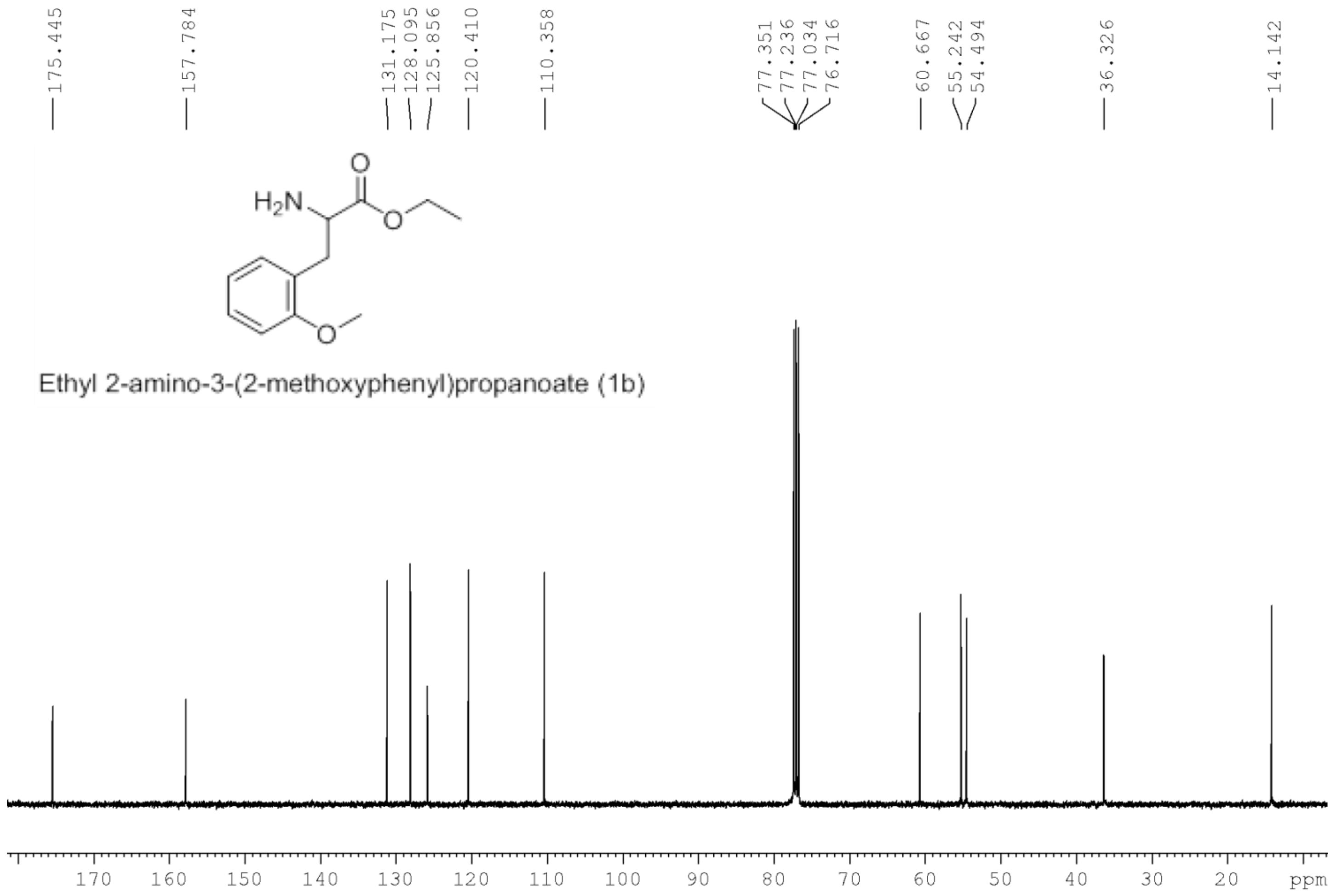
175.445
157.784
131.175
128.095
125.856
120.410
110.358
77.351
77.236
77.034
76.716
60.667
55.242
54.494
36.326
14.142
Ethyl 2-amino-3-(2-methoxyphenyl)propanoate (1b)
170
160
150
140
130
120
110
100
90
80
70
60
50
40
30
20
ppm

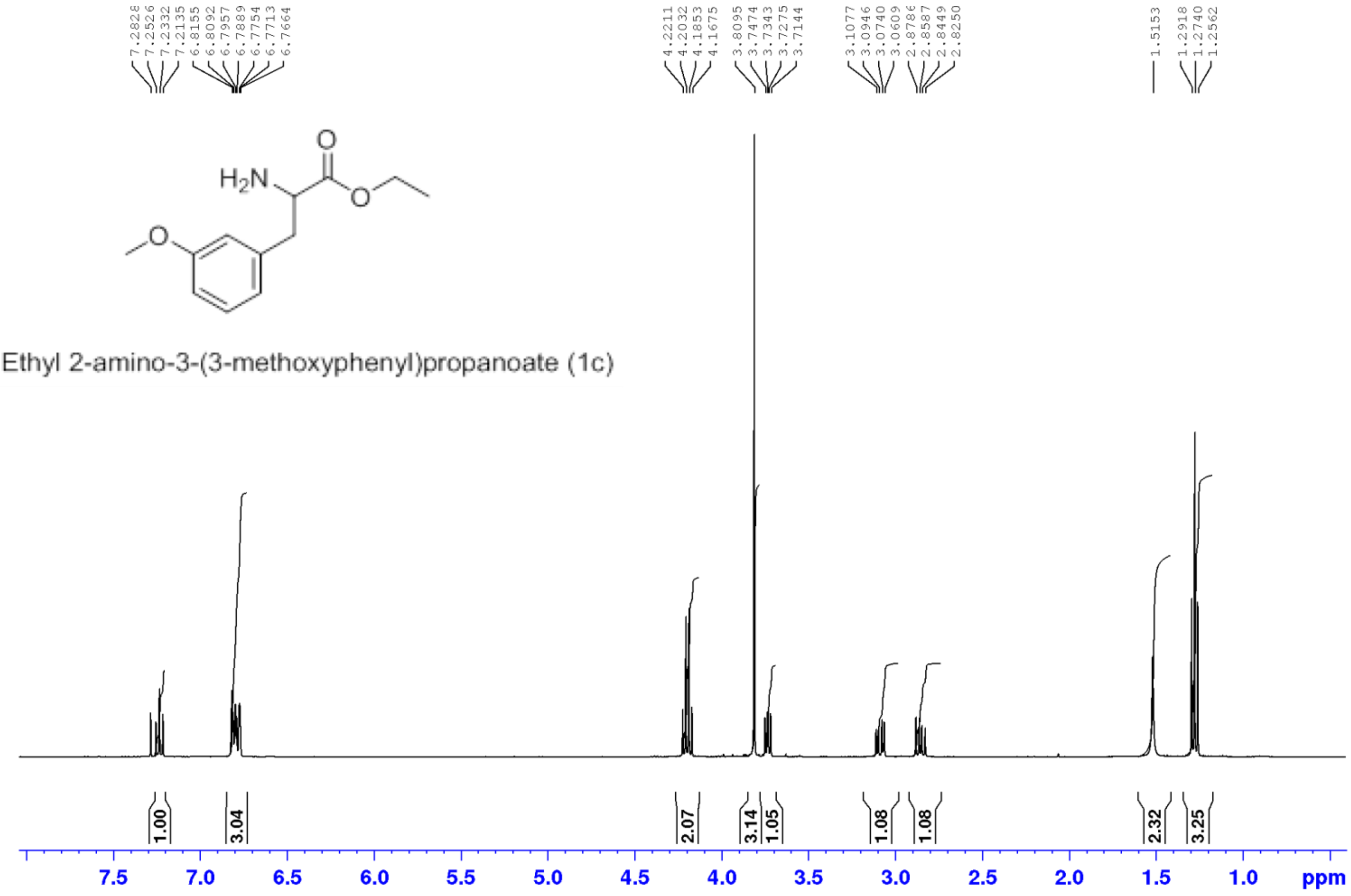

Ethyl 2-amino-3-(3-methoxyphenyl)propanoate (1c)

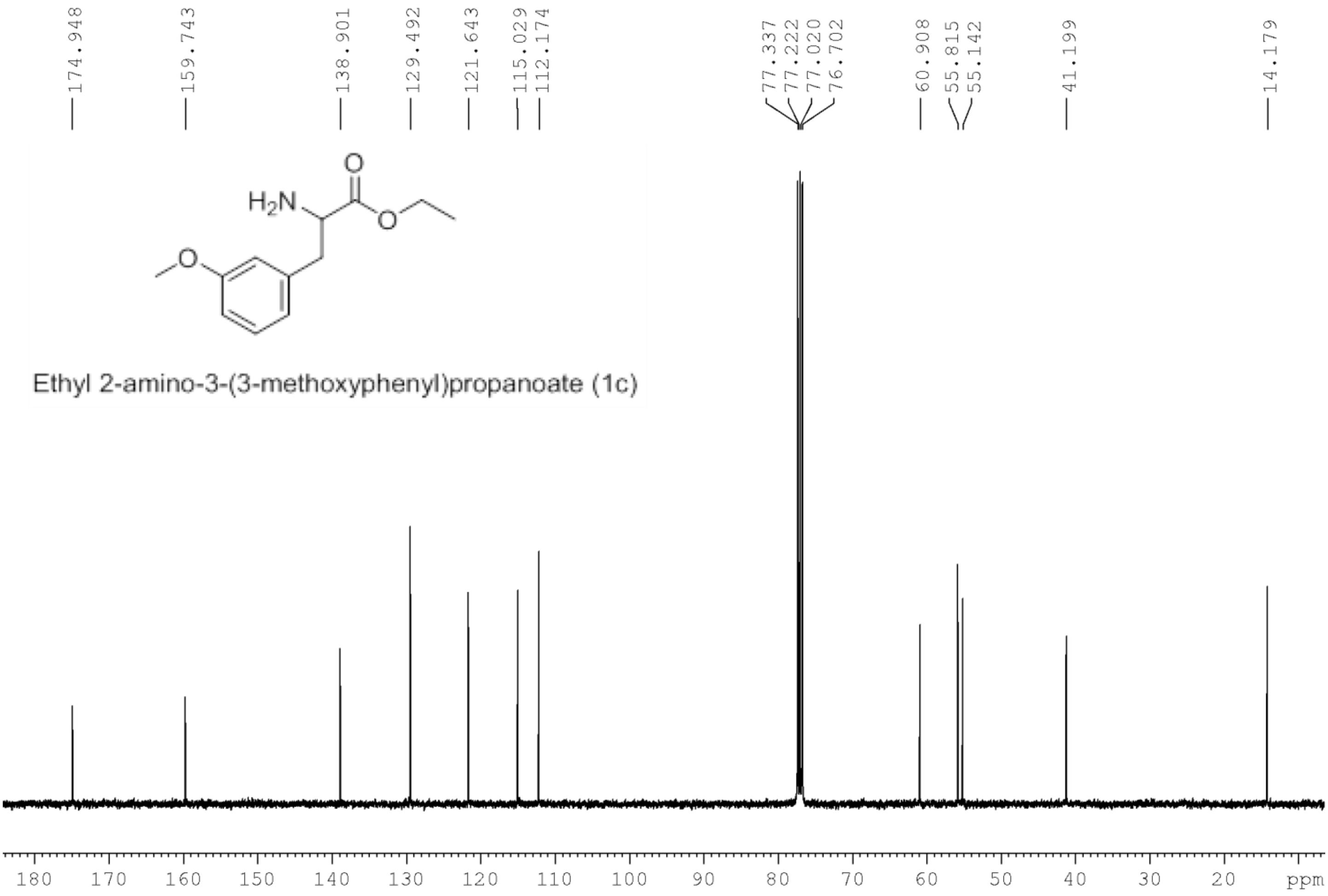
174.948
159.743
138.901
129.492
121.643
115.029
112.174
77.337
77.222
77.020
76.702
60.908
55.815
55.142
41.199
14.179
180
170
160
150
140
130
120
110
100
90
80
70
60
50
40
30
20
ppm

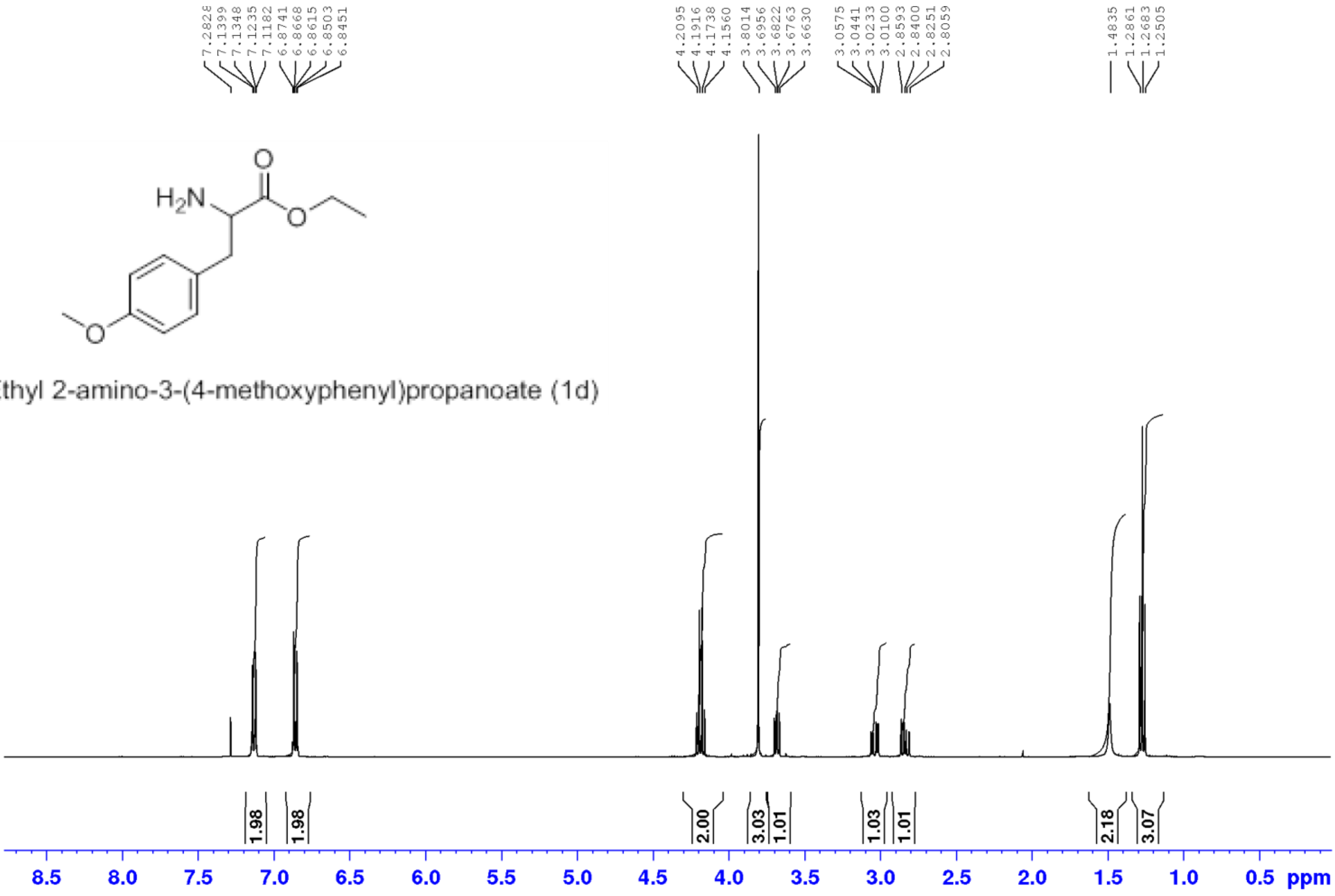

Ethyl 2-amino-3-(4-methoxyphenyl)propanoate (1d)

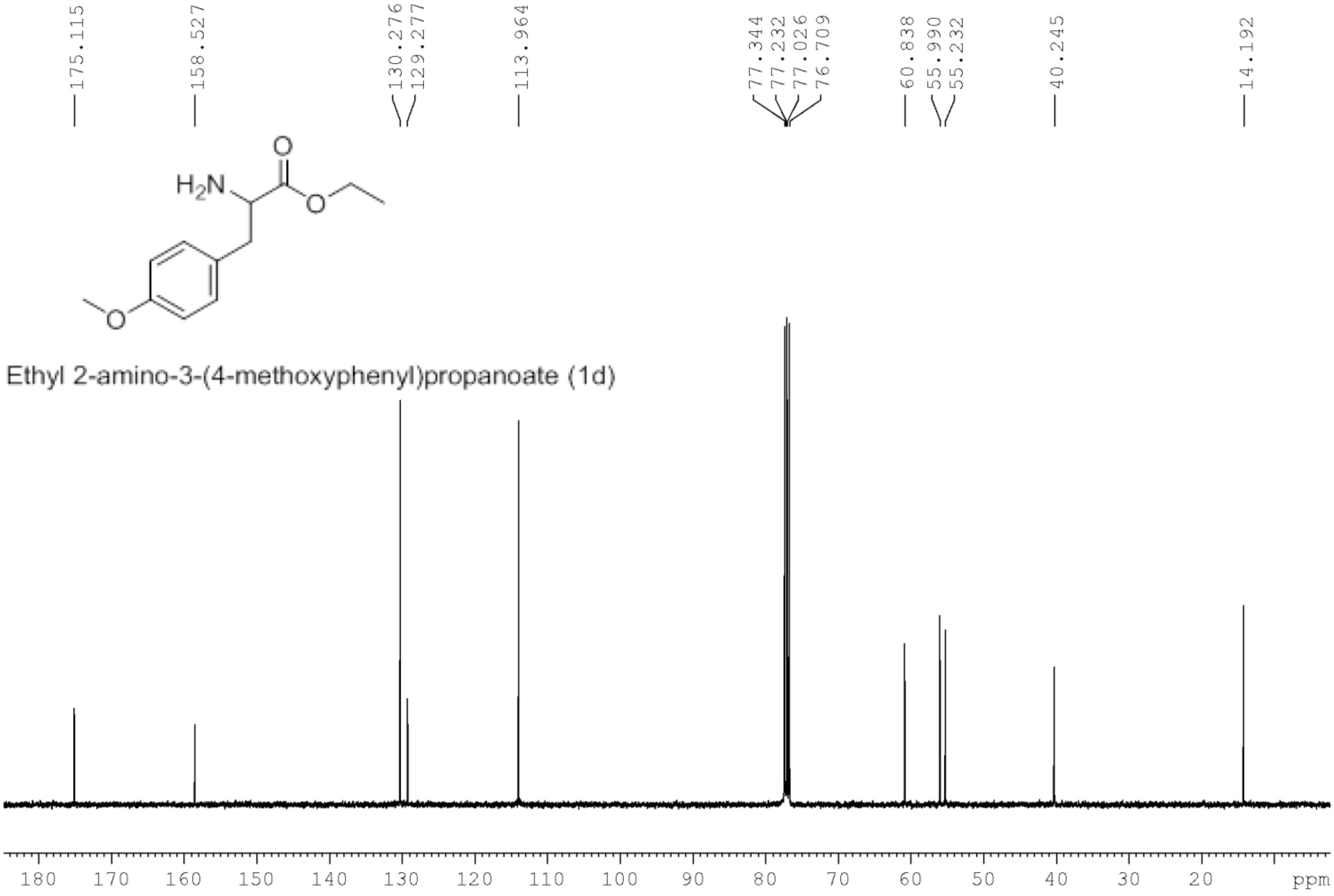

Ethyl 2-amino-3-(4-methoxyphenyl)propanoate (1d)

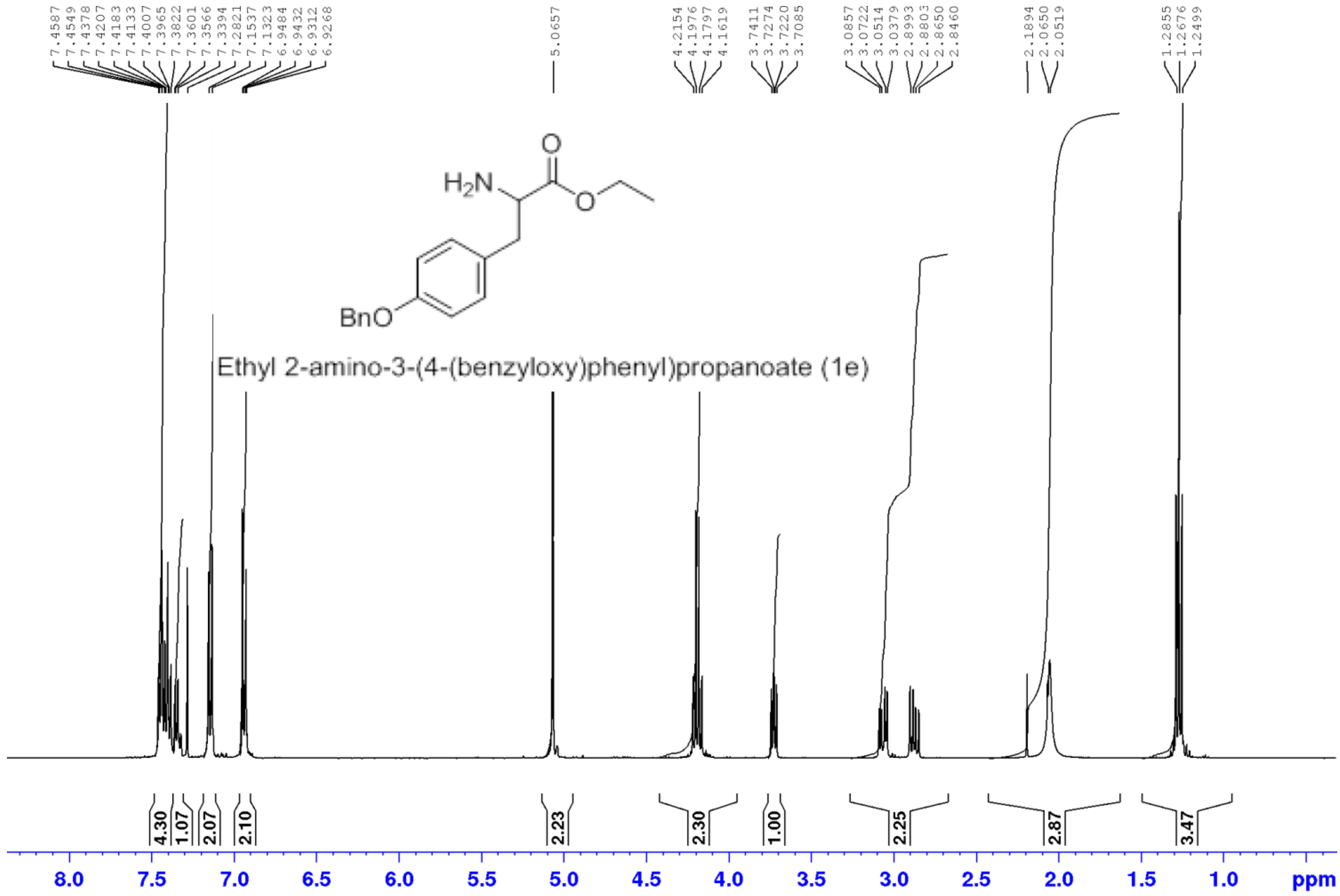

7.4587
7.4549
7.4378
7.4207
7.4183
7.4133
7.4007
7.3965
7.3822
7.3601
7.3566
7.3394
7.2821
7.1537
7.1323
6.9484
6.9432
6.9312
6.9268
5.0657
4.2154
4.1976
4.1797
4.1619
3.7411
3.7274
3.7220
3.7085
3.0857
3.0722
3.0514
3.0379
2.8993
2.8803
2.8650
2.8460
2.1894
2.0650
2.0519
1.2855
1.2676
1.2499
H_2N
O
O
BnO
Ethyl 2-amino-3-(4-(benzyloxy)phenyl)propanoate (1e)
4.30
1.07
2.07
2.10
2.23
2.30
1.00
2.25
2.87
3.47
8.0
7.5
7.0
6.5
6.0
5.5
5.0
4.5
4.0
3.5
3.0
2.5
2.0
1.5
1.0
ppm

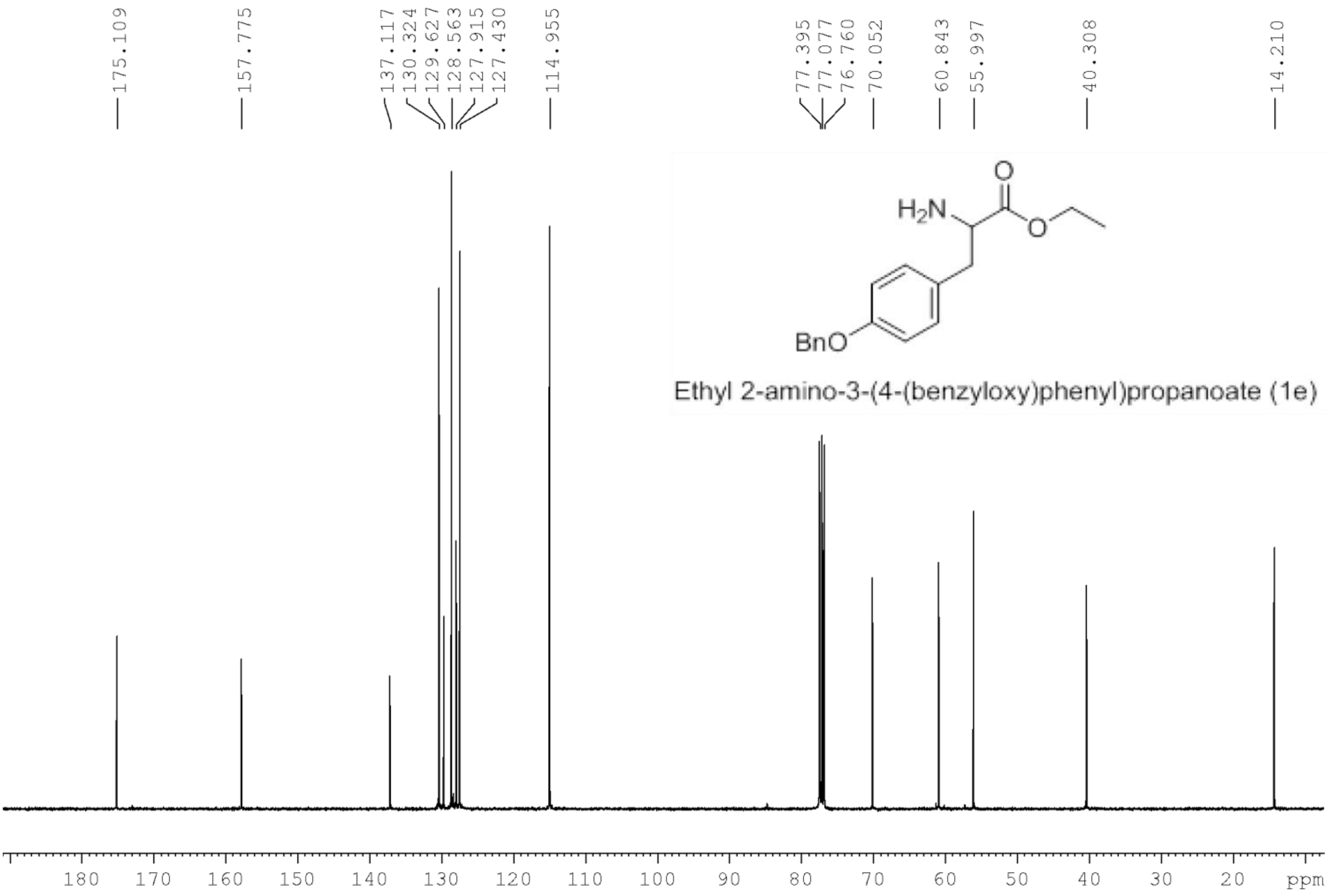
175.109
157.775
137.117
130.324
129.627
128.563
127.915
127.430
114.955
77.395
77.077
76.760
70.052
60.843
55.997
40.308
14.210
H2N
O
O
BnO
Ethyl 2-amino-3-(4-(benzyloxy)phenyl)propanoate (1e)
180
170
160
150
140
130
120
110
100
90
80
70
60
50
40
30
20
ppm

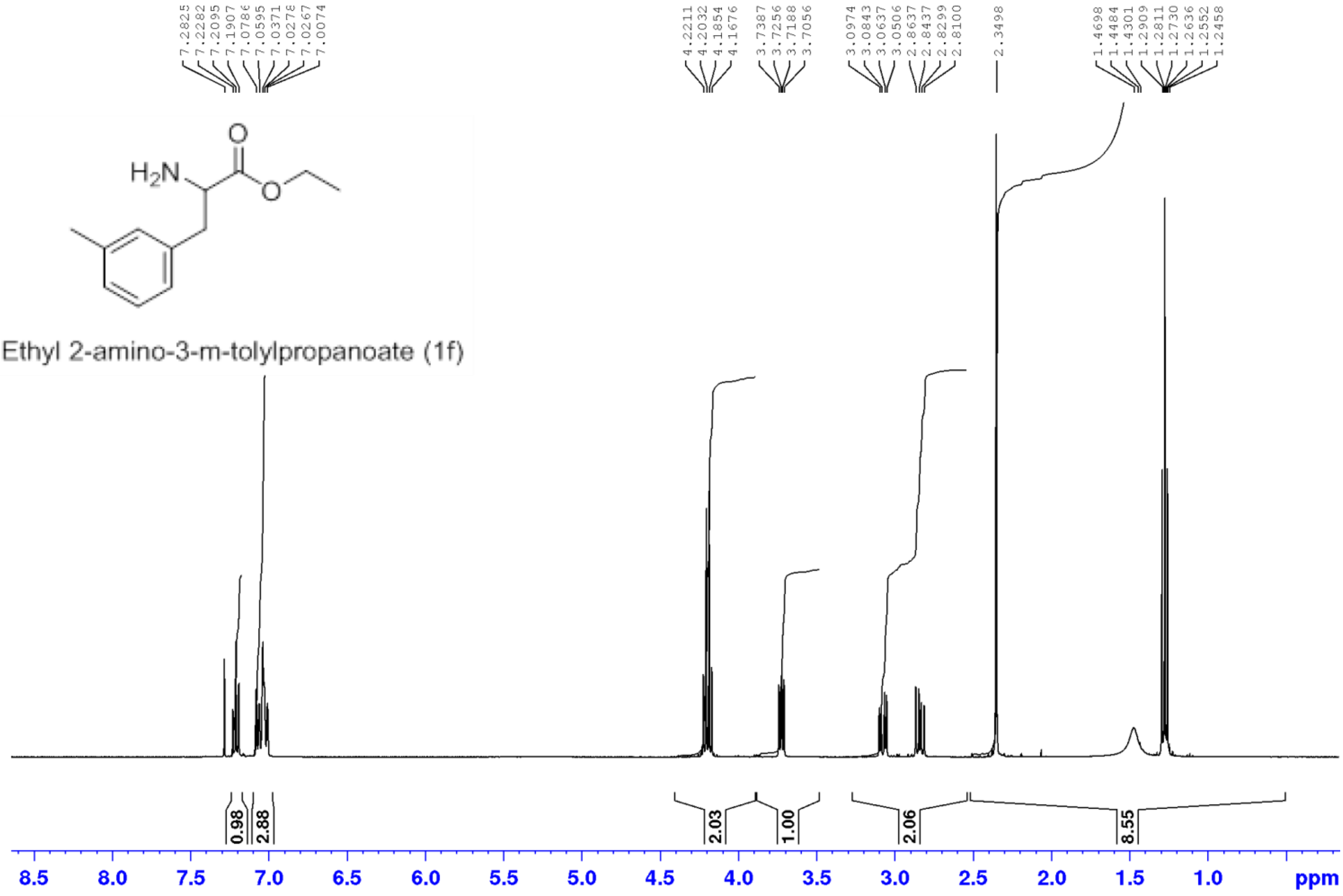
7.2825
7.2282
7.2095
7.1907
7.0786
7.0595
7.0371
7.0278
7.0267
7.0074
4.2211
4.2032
4.1854
4.1676
3.7387
3.7256
3.7188
3.7056
3.0974
3.0843
3.0637
3.0506
2.8637
2.8437
2.8299
2.8100
2.3498
1.4698
1.4484
1.4301
1.2909
1.2811
1.2730
1.2636
1.2552
1.2458
Ethyl 2-amino-3-m-tolylpropanoate (1f)
0.98
2.88
2.03
1.00
2.06
8.55
8.5
8.0
7.5
7.0
6.5
6.0
5.5
5.0
4.5
4.0
3.5
3.0
2.5
2.0
1.5
1.0
ppm

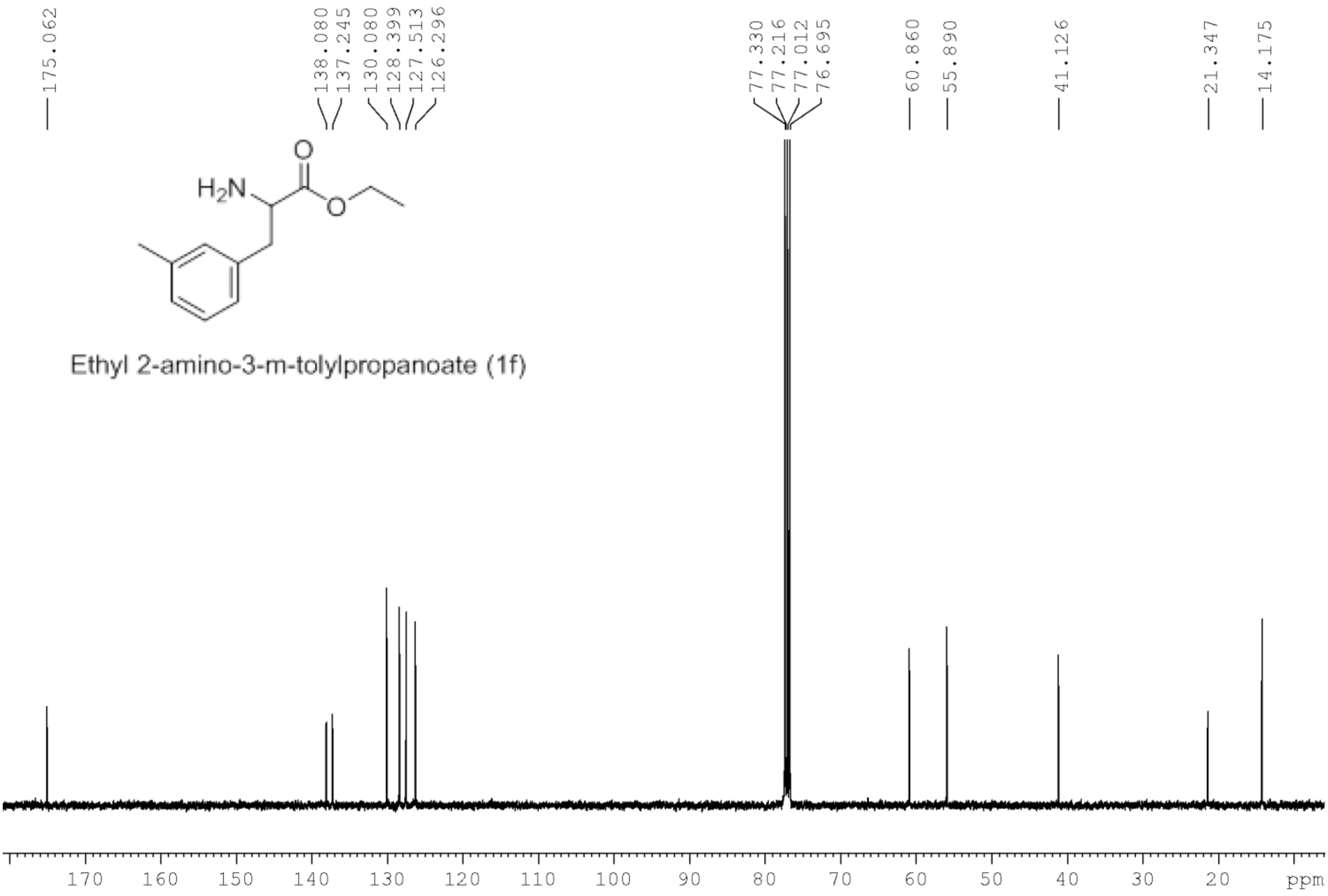

Ethyl 2-amino-3-m-tolylpropanoate (1f)

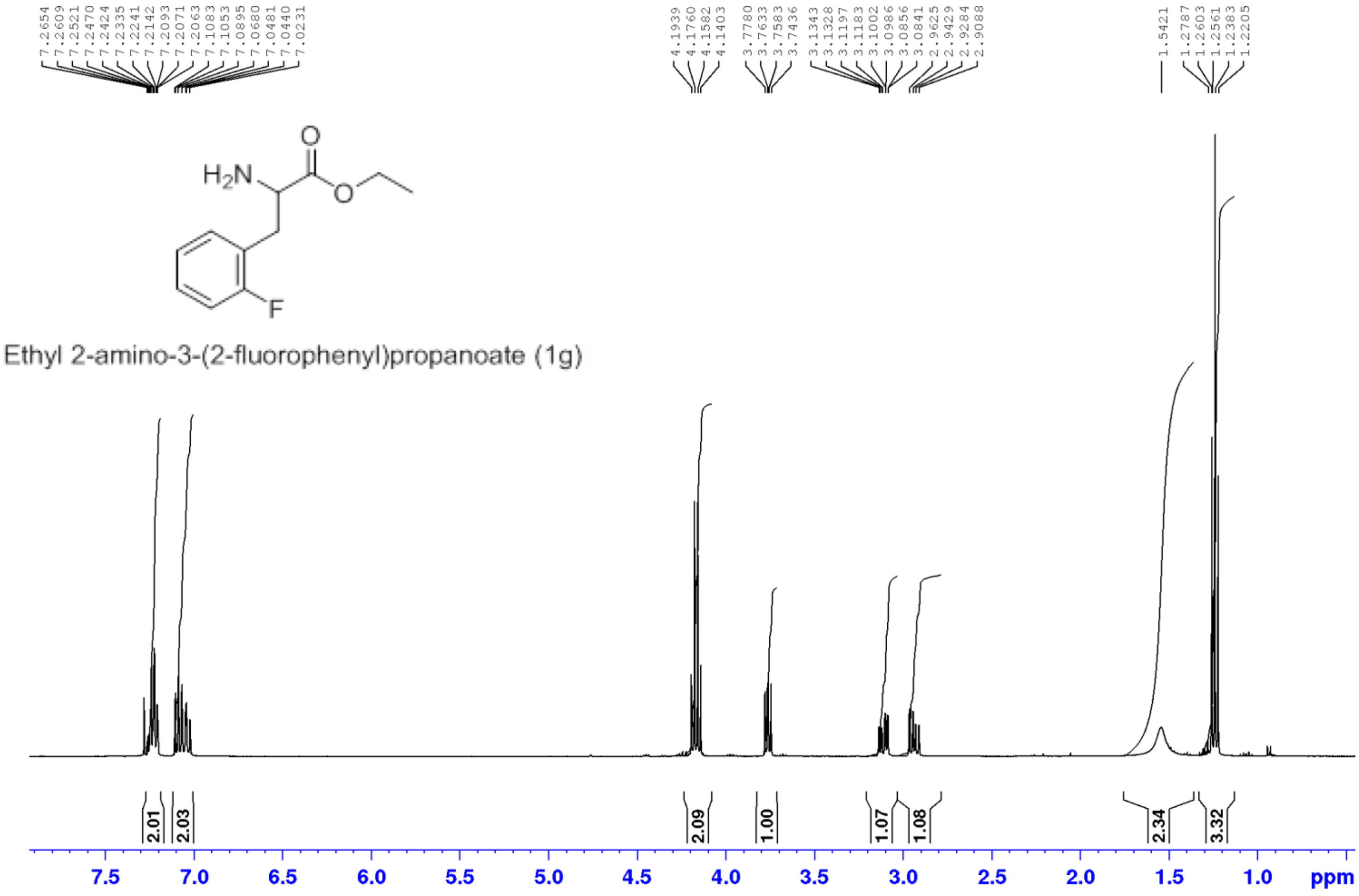
7.2654
7.2609
7.2521
7.2470
7.2424
7.2335
7.2241
7.2142
7.2093
7.2071
7.2063
7.1083
7.1053
7.0895
7.0680
7.0481
7.0440
7.0231
4.1939
4.1760
4.1582
4.1403
3.7780
3.7633
3.7583
3.7436
3.1343
3.1328
3.1197
3.1183
3.1002
3.0986
3.0856
3.0841
2.9625
2.9429
2.9284
2.9088
1.5421
1.2787
1.2603
1.2561
1.2383
1.2205
H_2N
O
O
F
Ethyl 2-amino-3-(2-fluorophenyl)propanoate (1g)
2.01
2.03
2.09
1.00
1.07
1.08
2.34
3.32
7.5
7.0
6.5
6.0
5.5
5.0
4.5
4.0
3.5
3.0
2.5
2.0
1.5
1.0
ppm

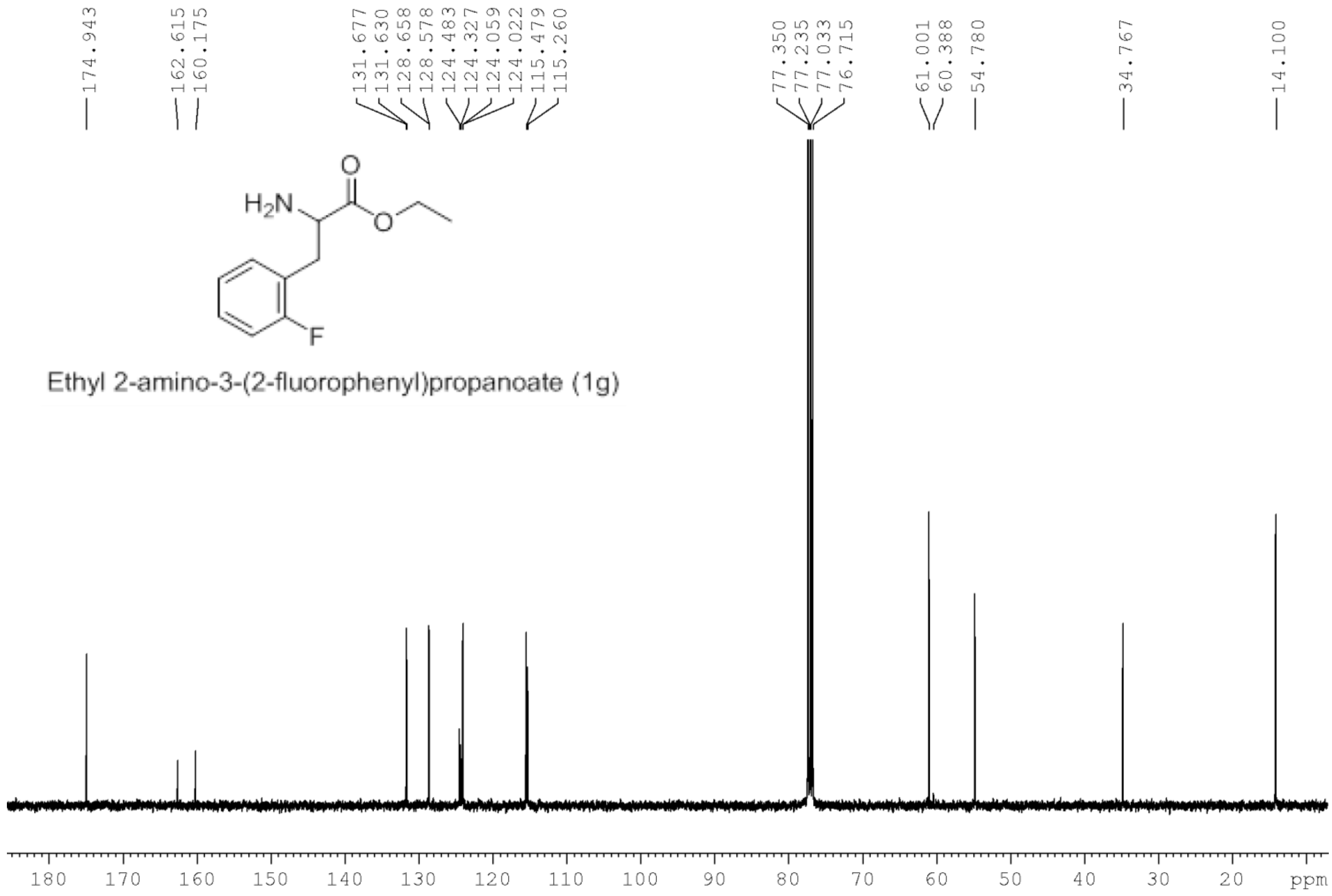

Ethyl 2-amino-3-(2-fluorophenyl)propanoate (1g)

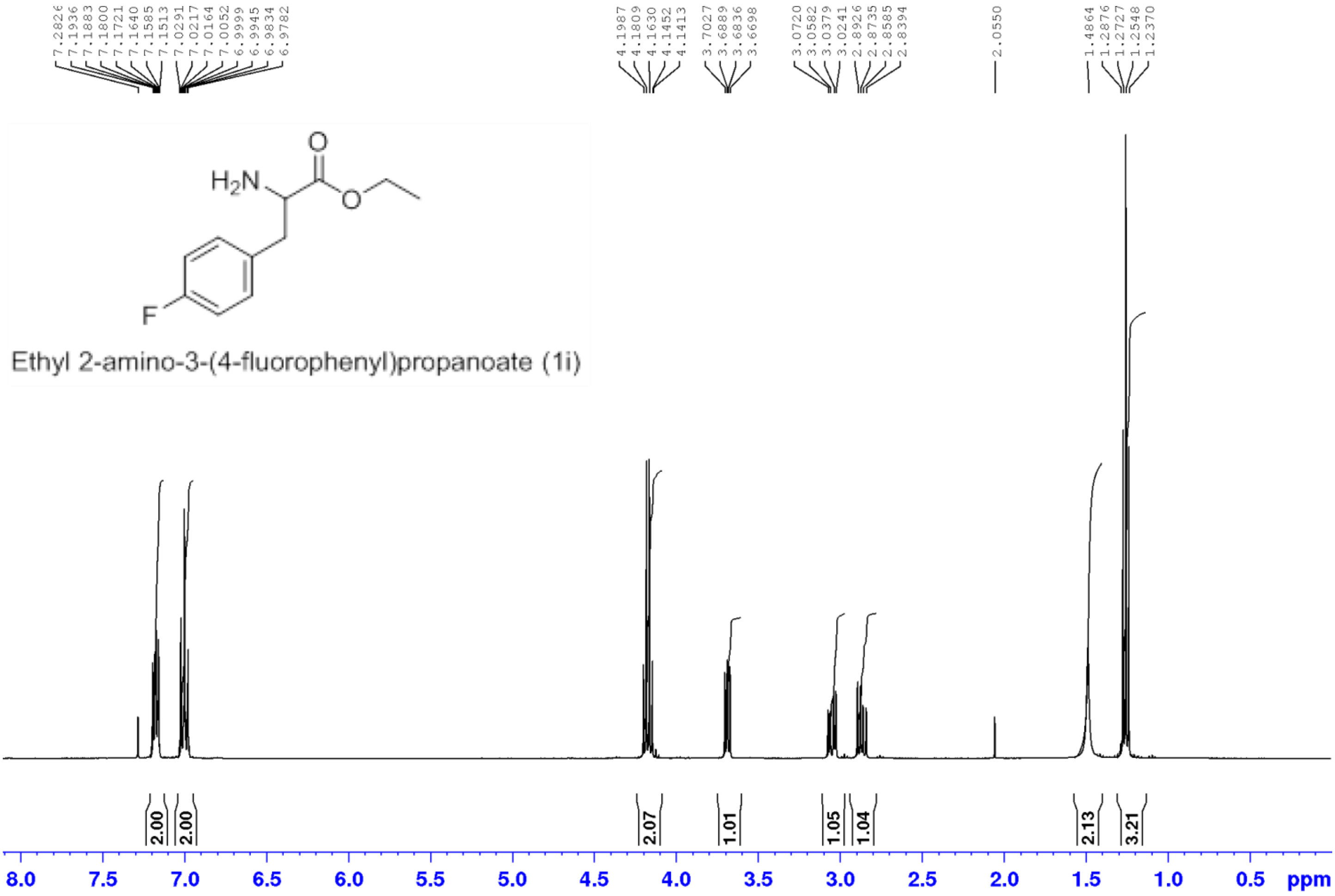
7.2826
7.1936
7.1883
7.1800
7.1721
7.1640
7.1585
7.1513
7.0291
7.0217
7.0164
7.0052
6.9999
6.9945
6.9834
6.9782
4.1987
4.1809
4.1630
4.1452
4.1413
3.7027
3.6889
3.6836
3.6698
3.0720
3.0582
3.0379
3.0241
2.8926
2.8735
2.8585
2.8394
2.0550
1.4864
1.2876
1.2727
1.2548
1.2370
H_2N
O
O
F
Ethyl 2-amino-3-(4-fluorophenyl)propanoate (1i)
2.00
2.00
2.07
1.01
1.05
1.04
2.13
3.21
8.0
7.5
7.0
6.5
6.0
5.5
5.0
4.5
4.0
3.5
3.0
2.5
2.0
1.5
1.0
0.5
ppm

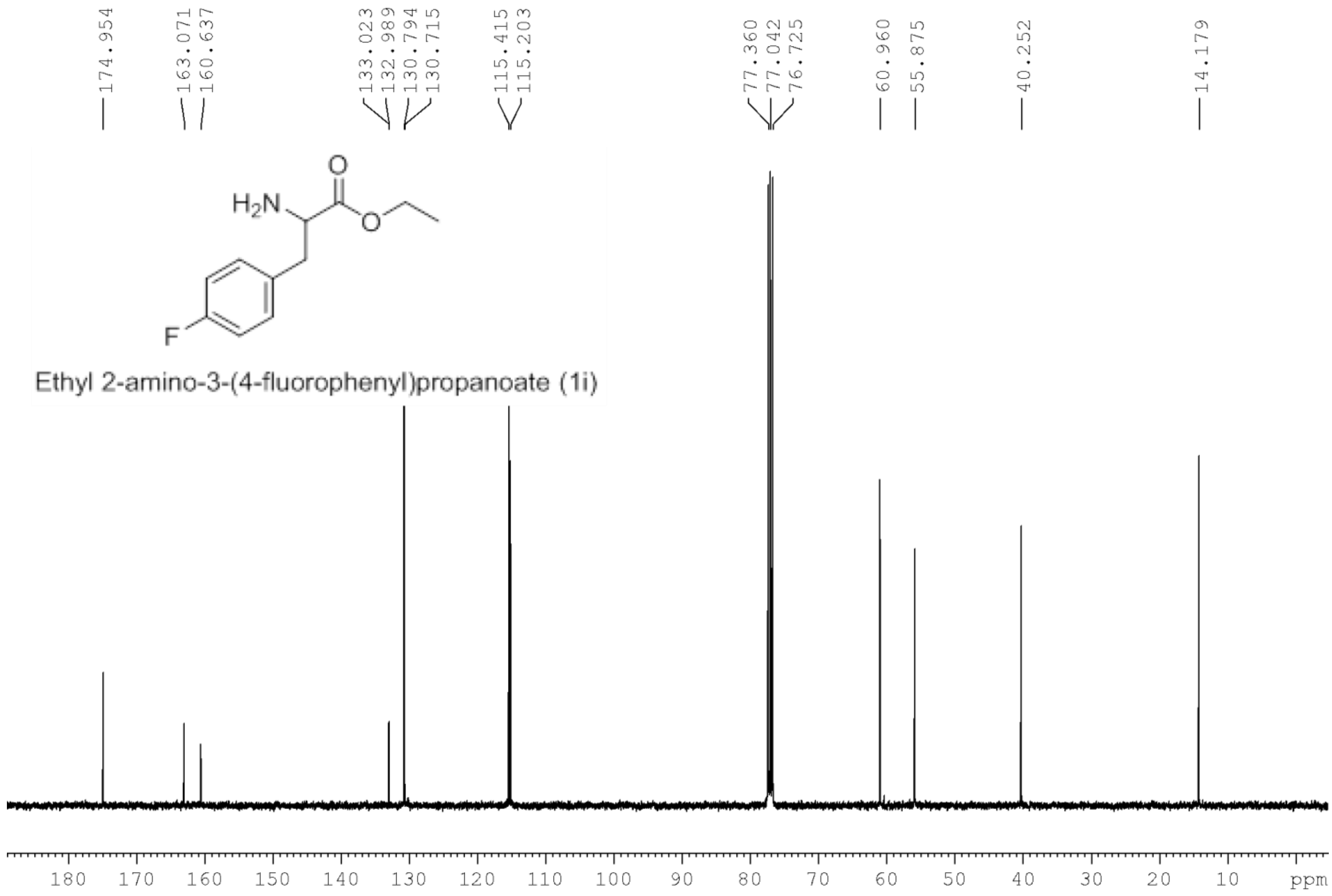

Ethyl 2-amino-3-(4-fluorophenyl)propanoate (1i)

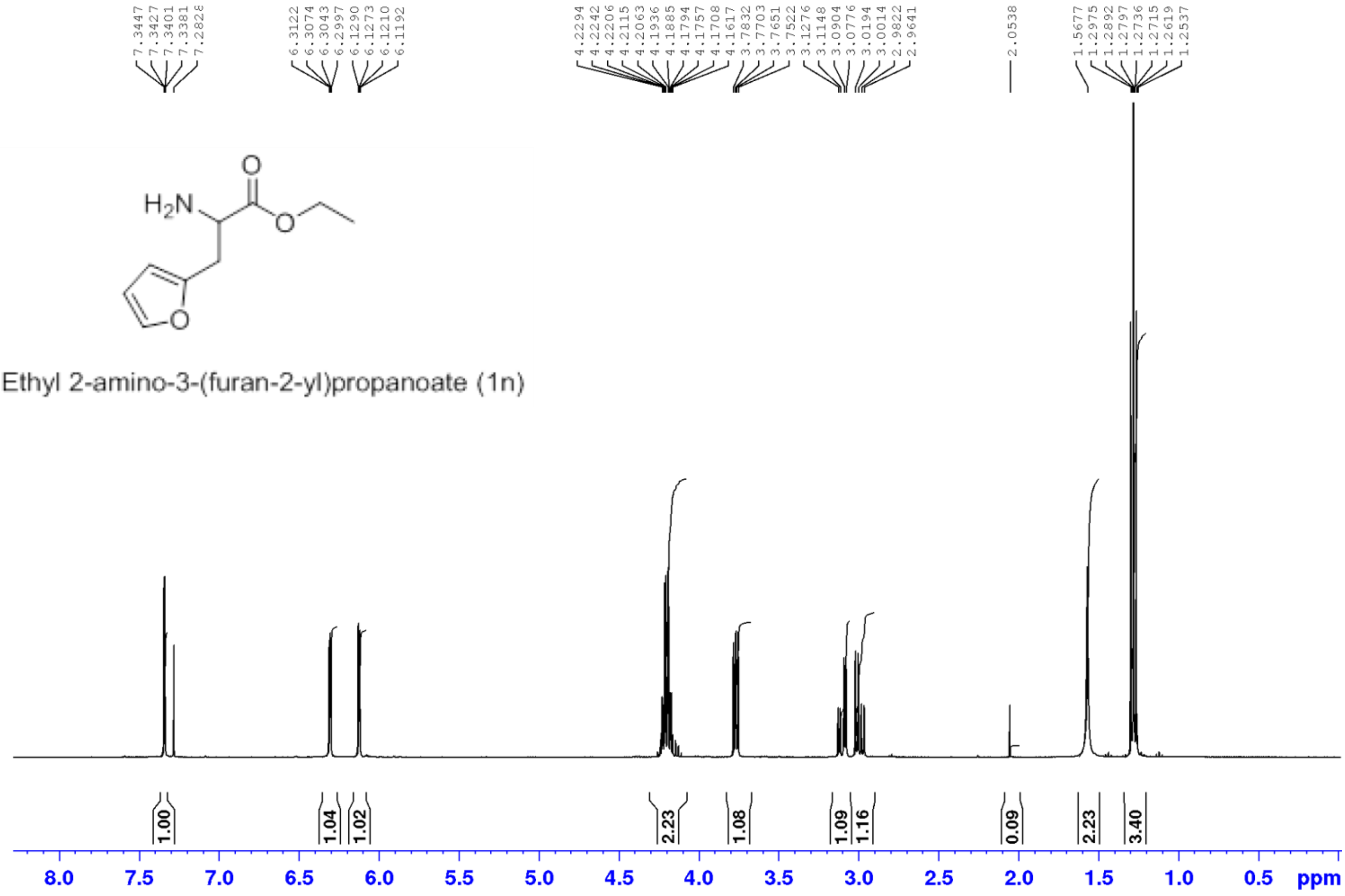

Ethyl 2-amino-3-(furan-2-yl)propanoate (1n)

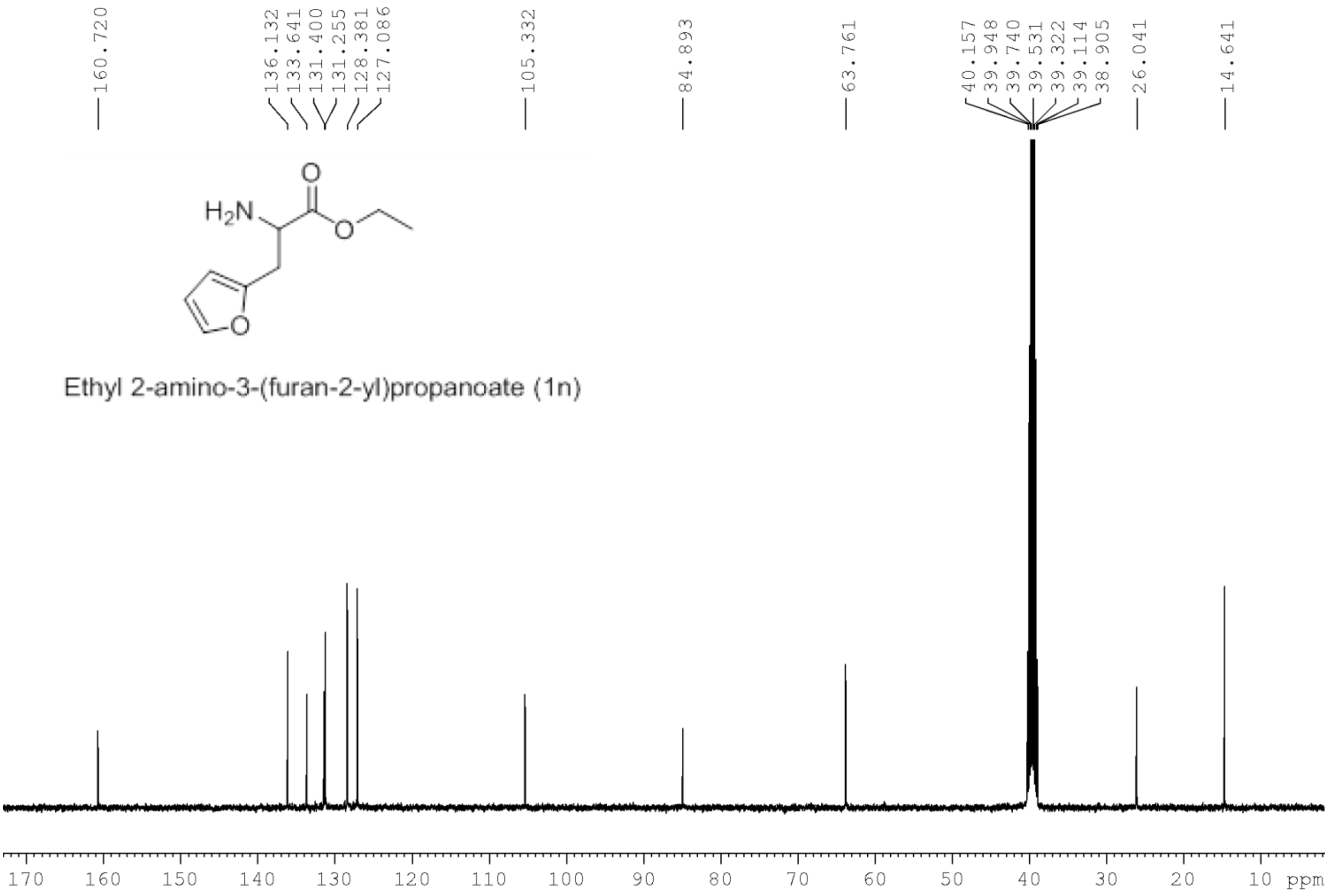

Ethyl 2-amino-3-(furan-2-yl)propanoate (1n)

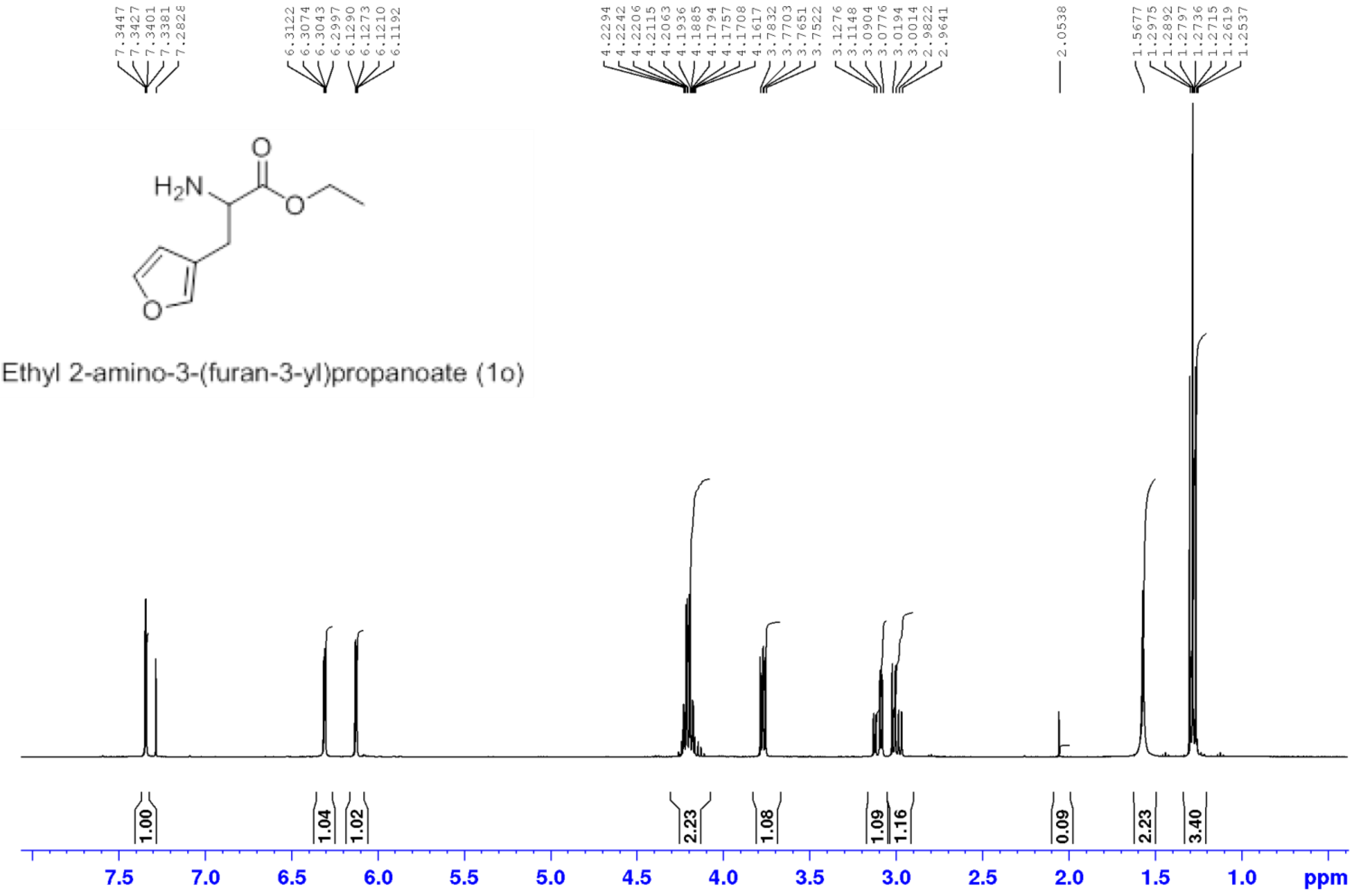

Ethyl 2-amino-3-(furan-3-yl)propanoate (1o)

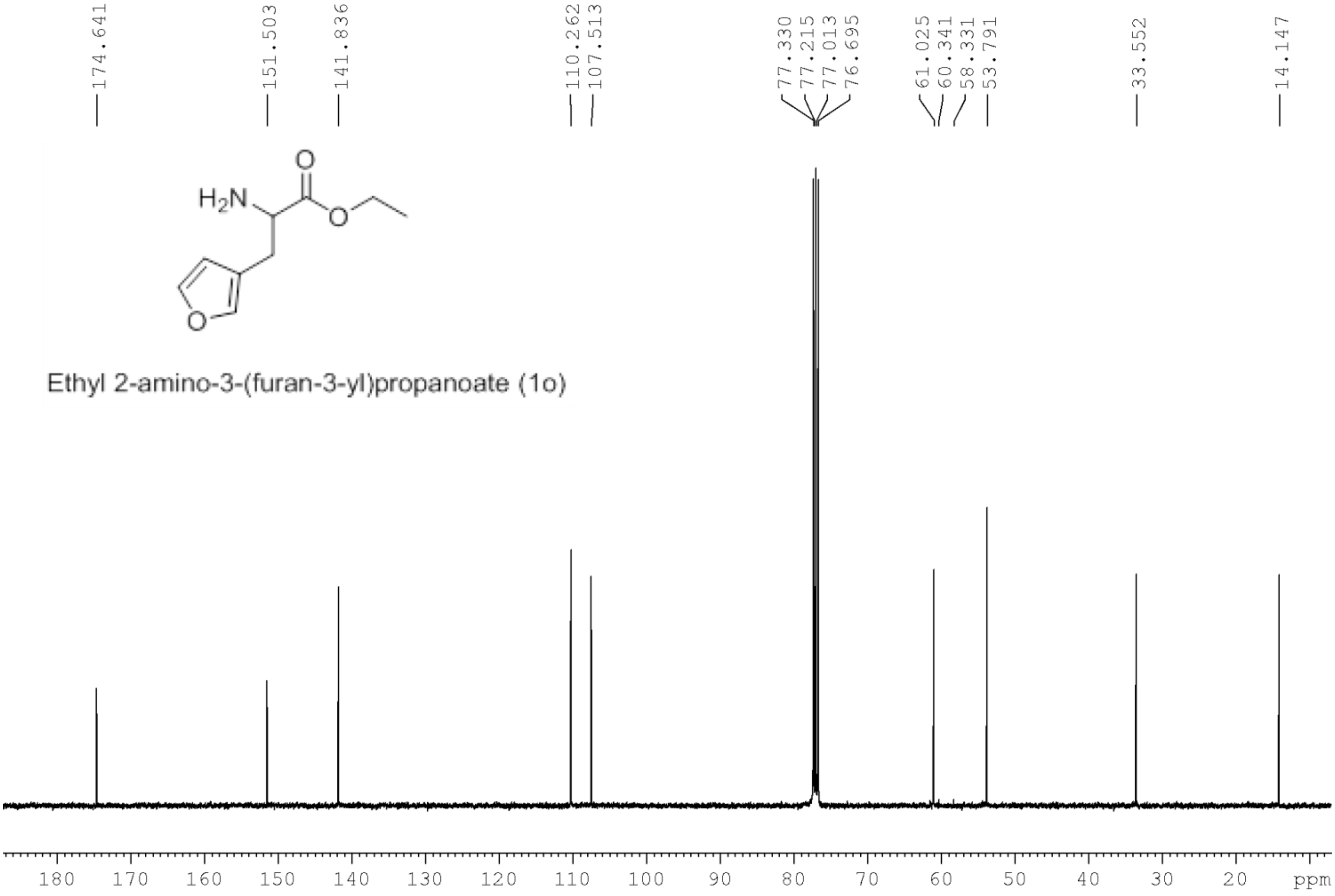

Ethyl 2-amino-3-(furan-3-yl)propanoate (1o)

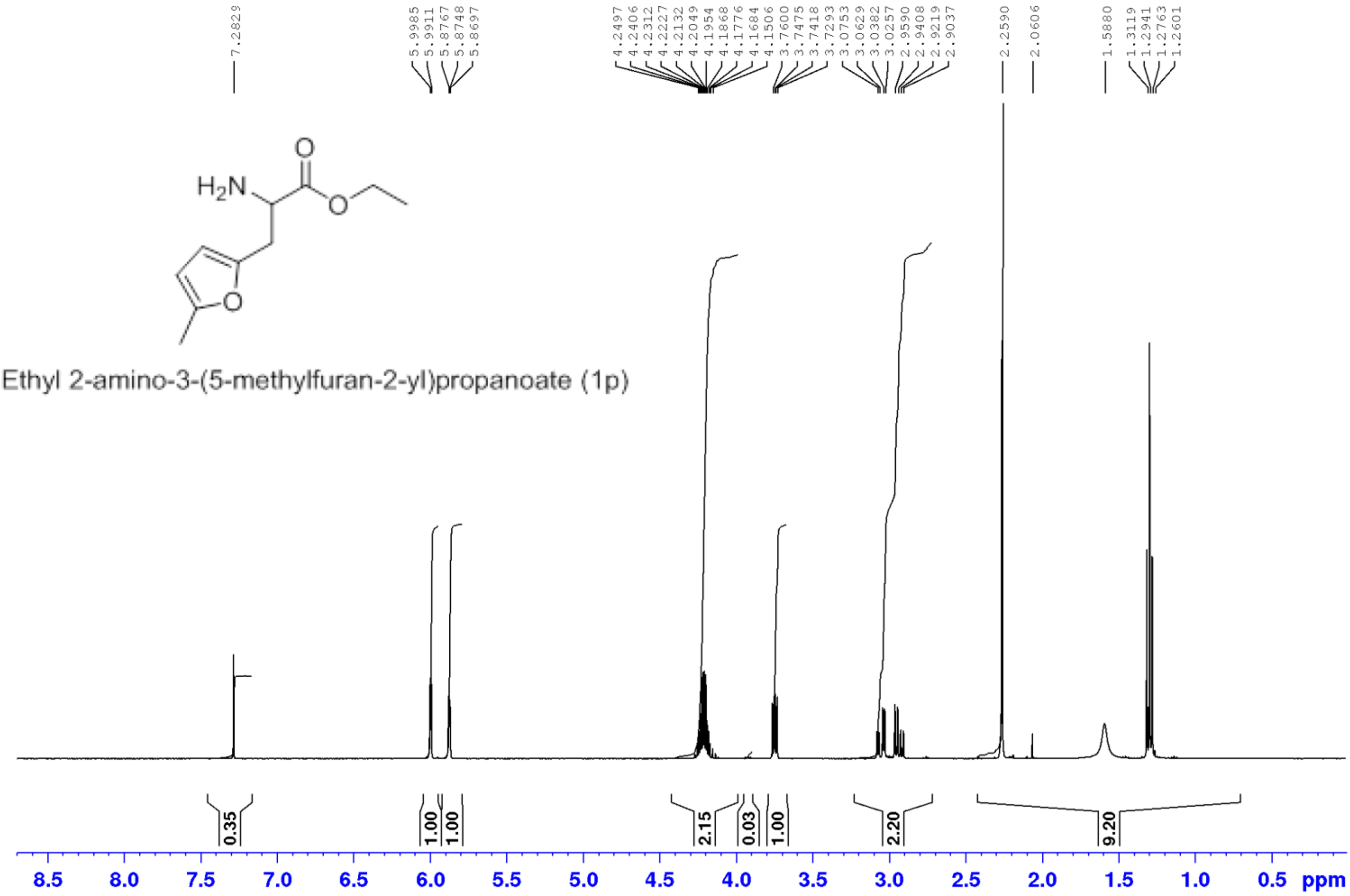

Ethyl 2-amino-3-(5-methylfuran-2-yl)propanoate (1p)

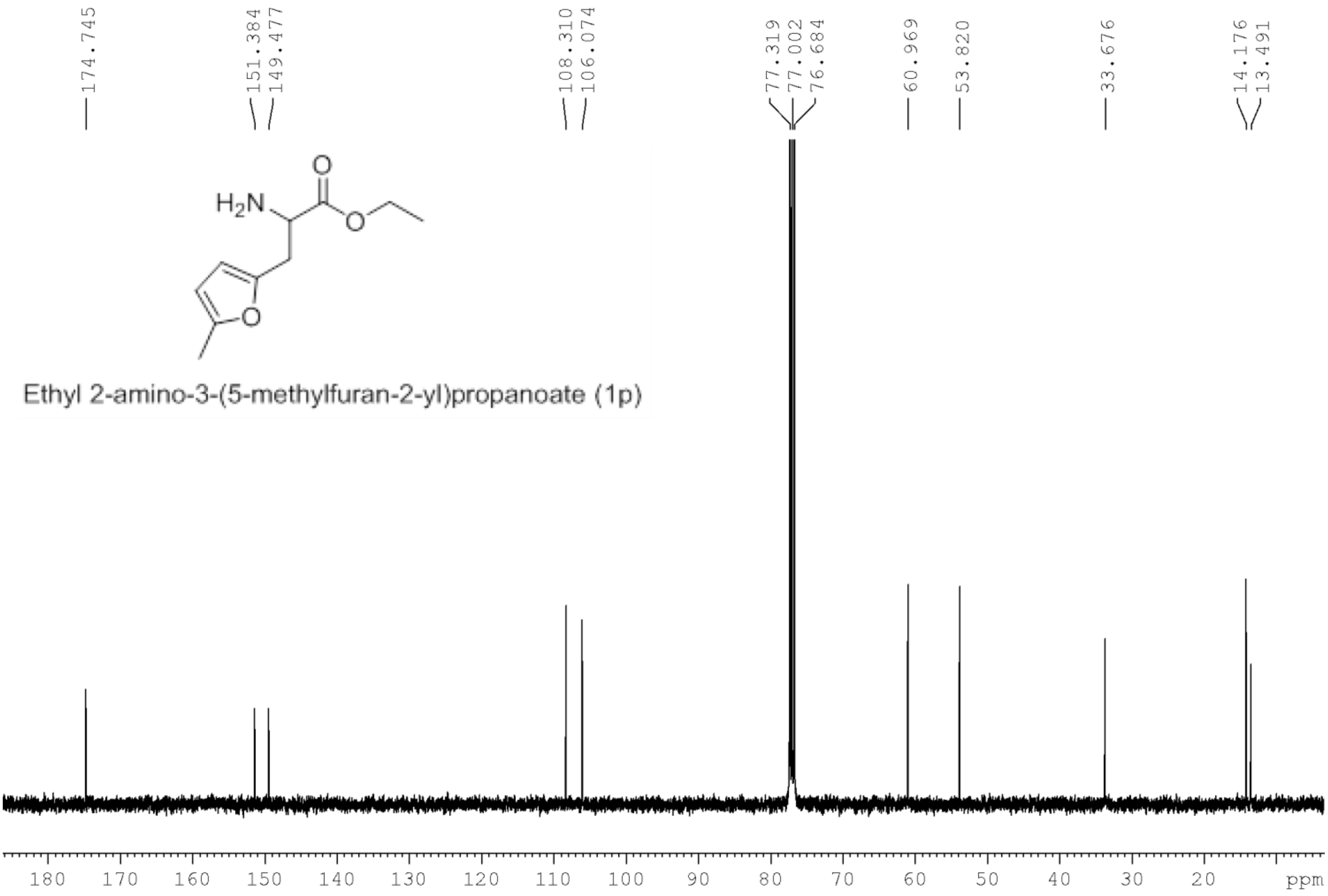

Ethyl 2-amino-3-(5-methylfuran-2-yl)propanoate (1p)

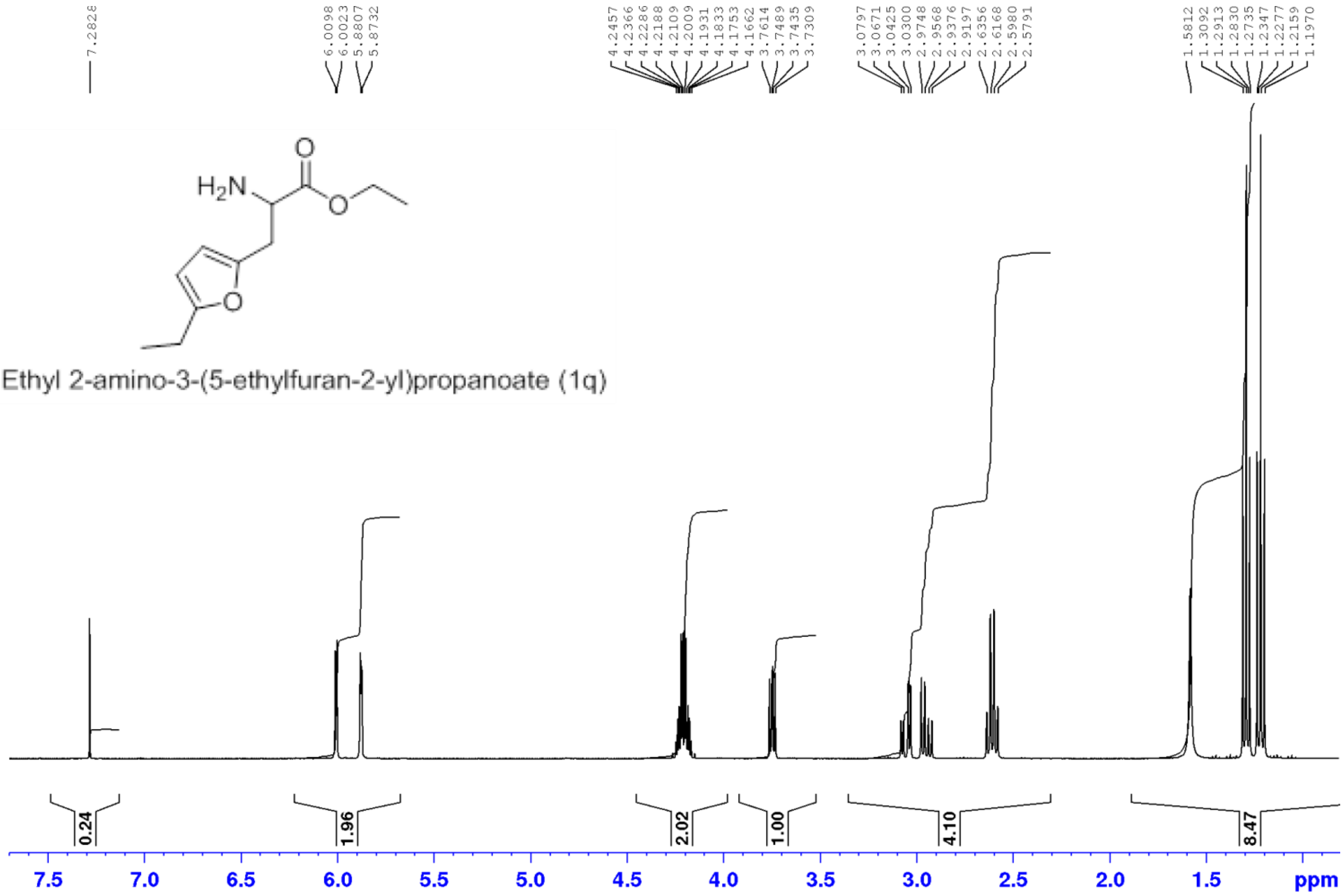

Ethyl 2-amino-3-(5-ethylfuran-2-yl)propanoate (1q)

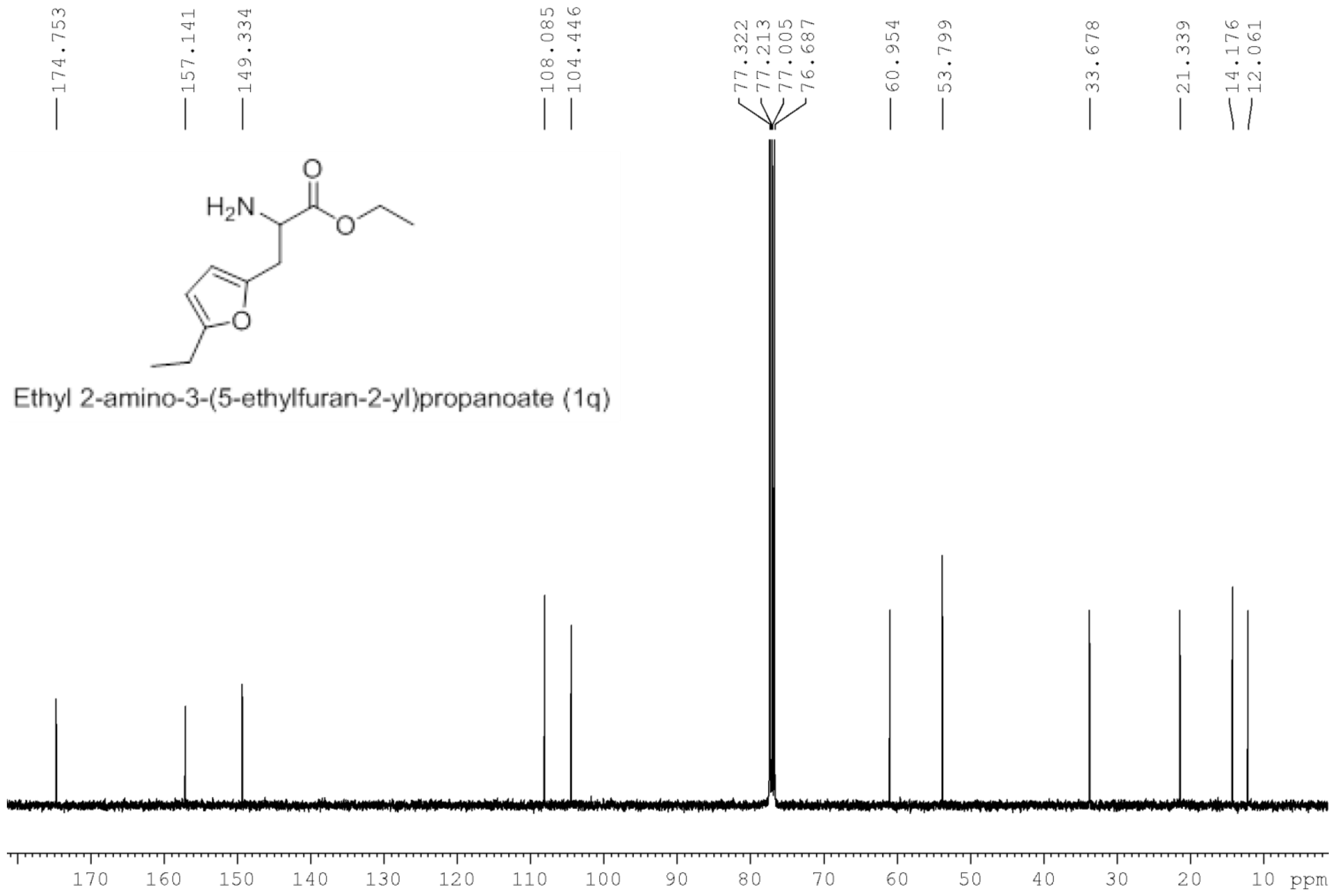
174.753
157.141
149.334
108.085
104.446
77.322
77.213
77.005
76.687
60.954
53.799
33.678
21.339
14.176
12.061
H2N
O
O
O
Ethyl 2-amino-3-(5-ethylfuran-2-yl)propanoate (1q)
170
160
150
140
130
120
110
100
90
80
70
60
50
40
30
20
10 ppm

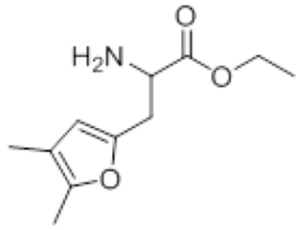

Ethyl 2-amino-3-(4,5-dimethylfuran-2-yl)propanoate (1r)

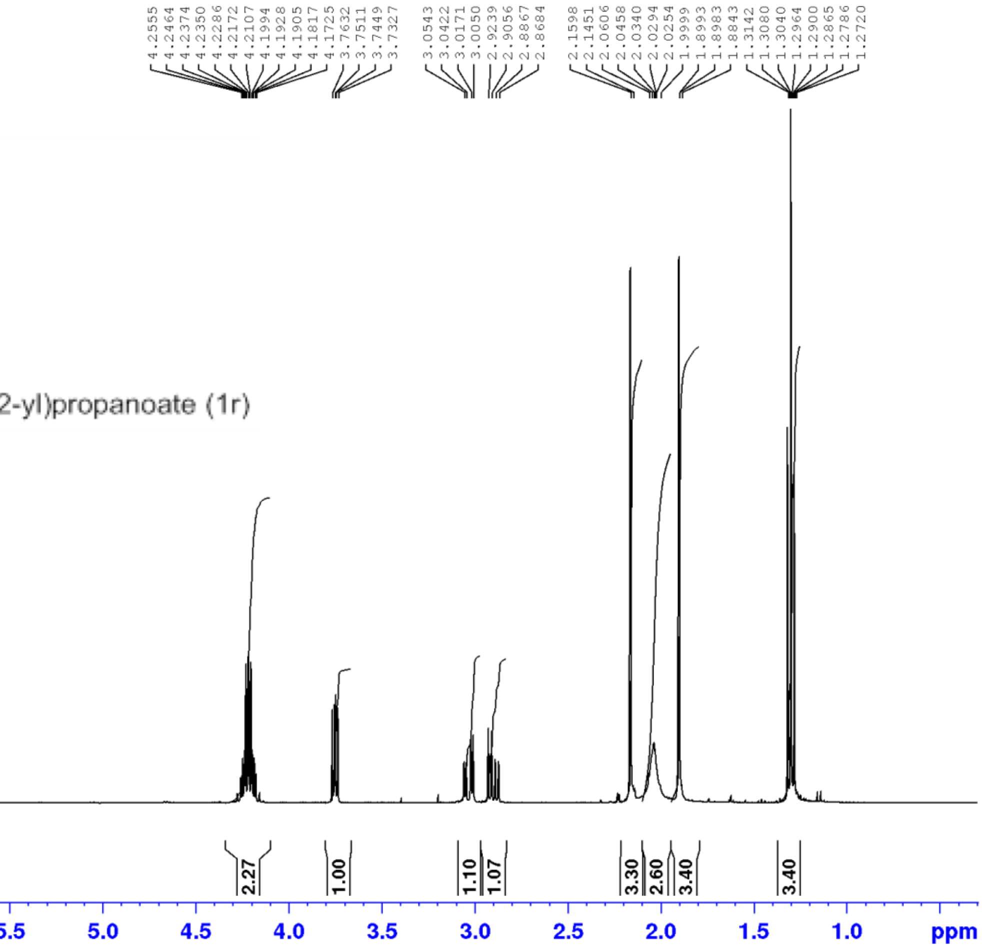

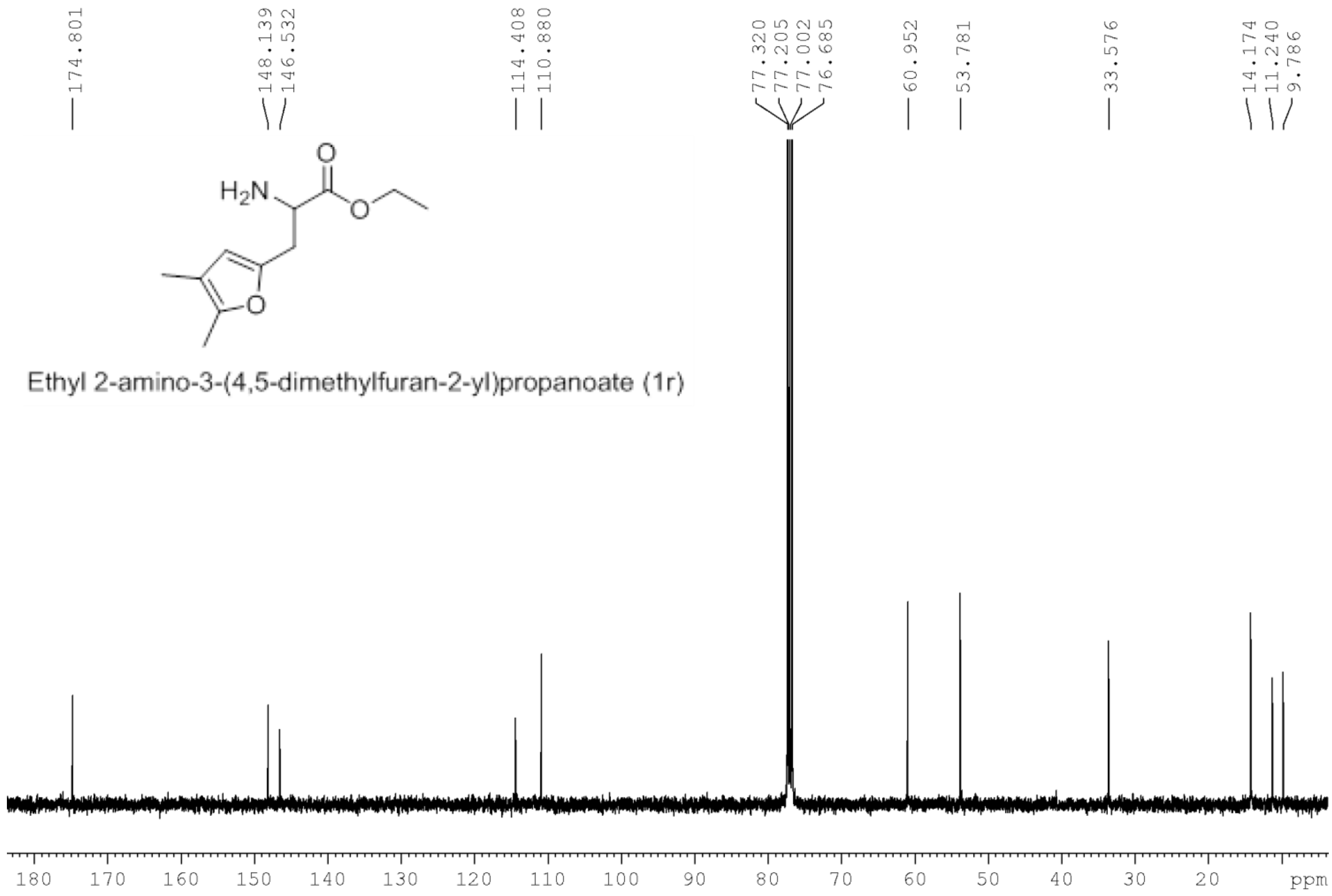
174.801
148.139
146.532
114.408
110.880
77.320
77.205
77.002
76.685
60.952
53.781
33.576
14.174
11.240
9.786
H2N
O
O
O
Ethyl 2-amino-3-(4,5-dimethylfuran-2-yl)propanoate (1r)
180
170
160
150
140
130
120
110
100
90
80
70
60
50
40
30
20
ppm

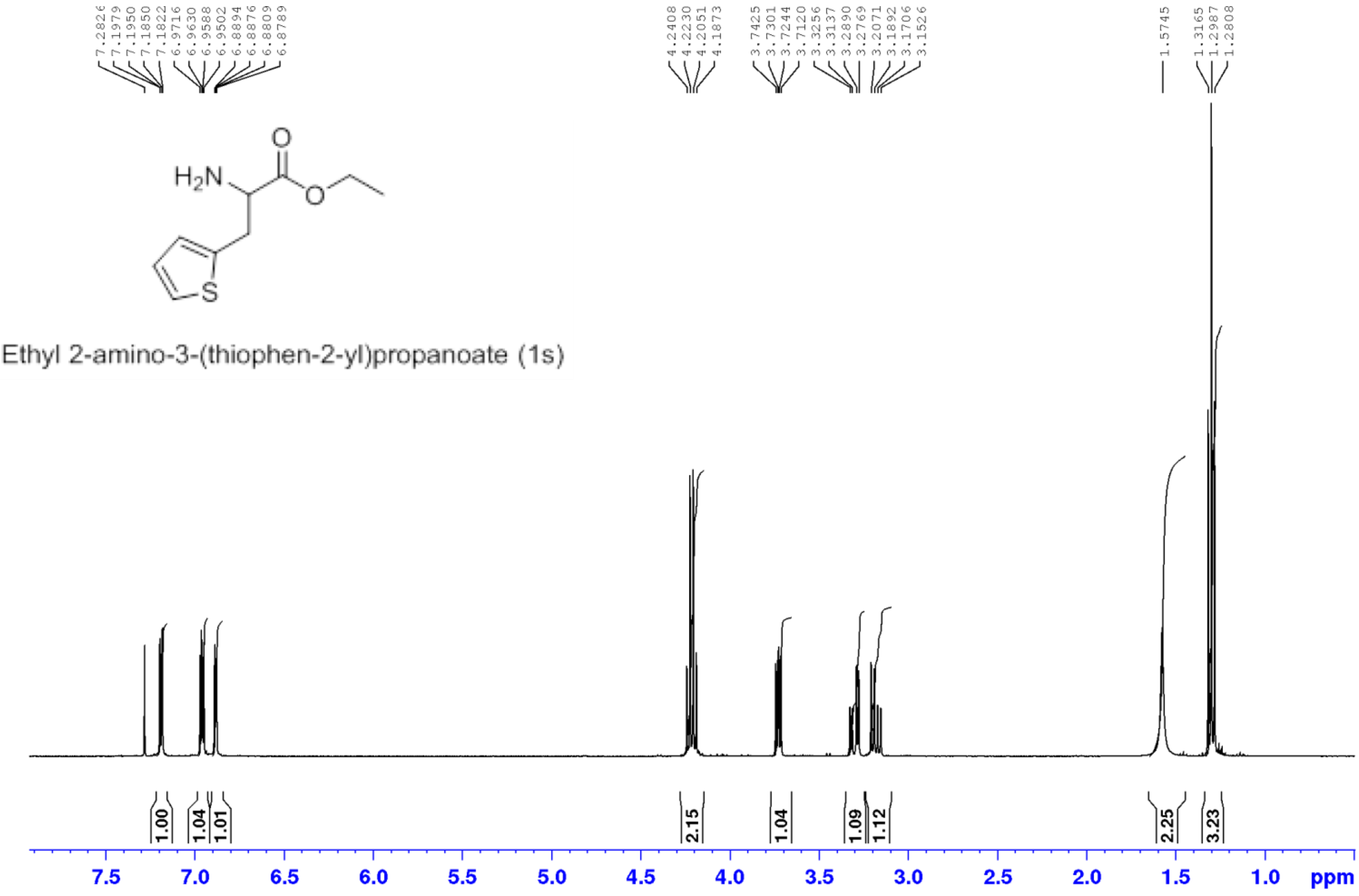

Ethyl 2-amino-3-(thiophen-2-yl)propanoate (1s)

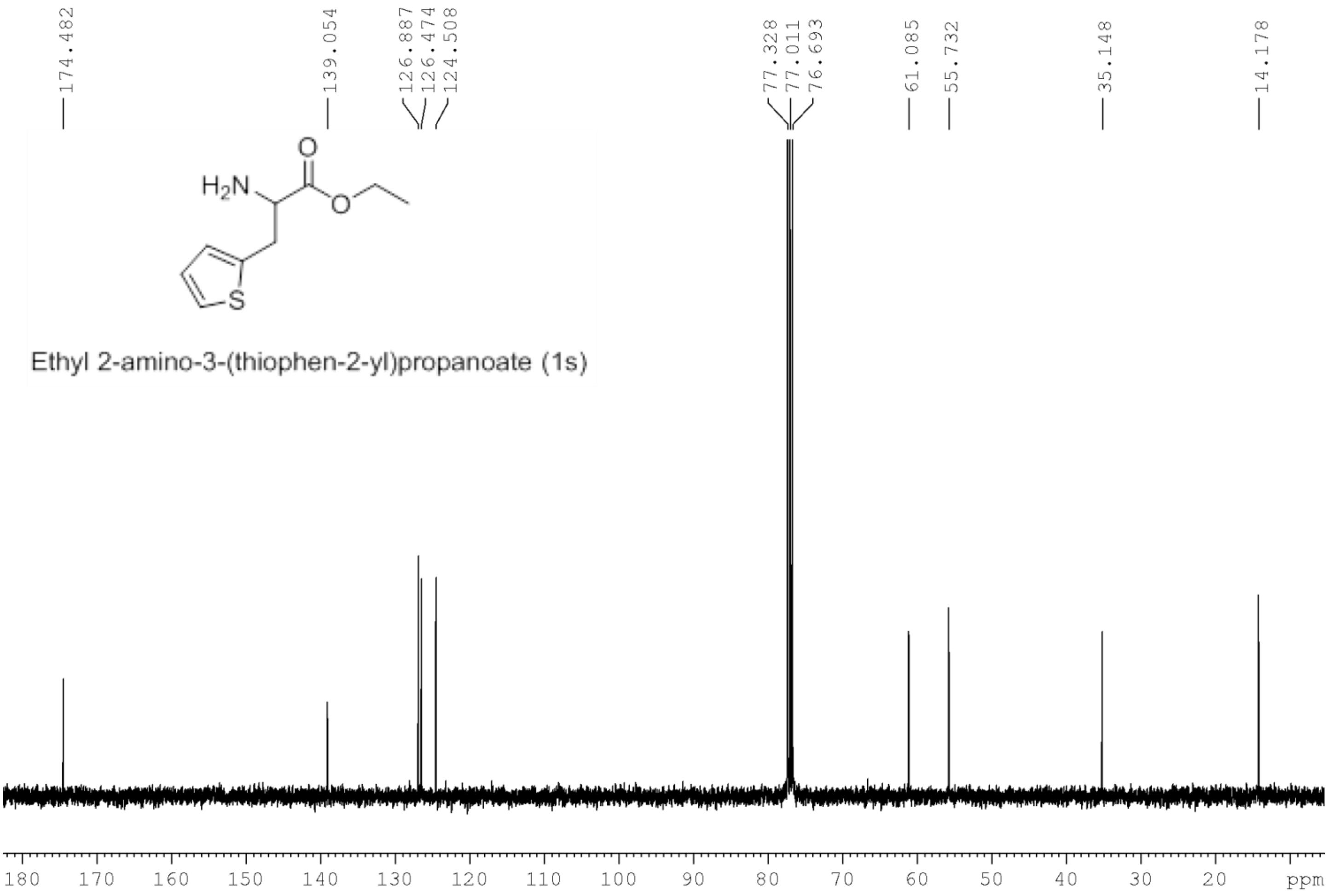

Ethyl 2-amino-3-(thiophen-2-yl)propanoate (1s)

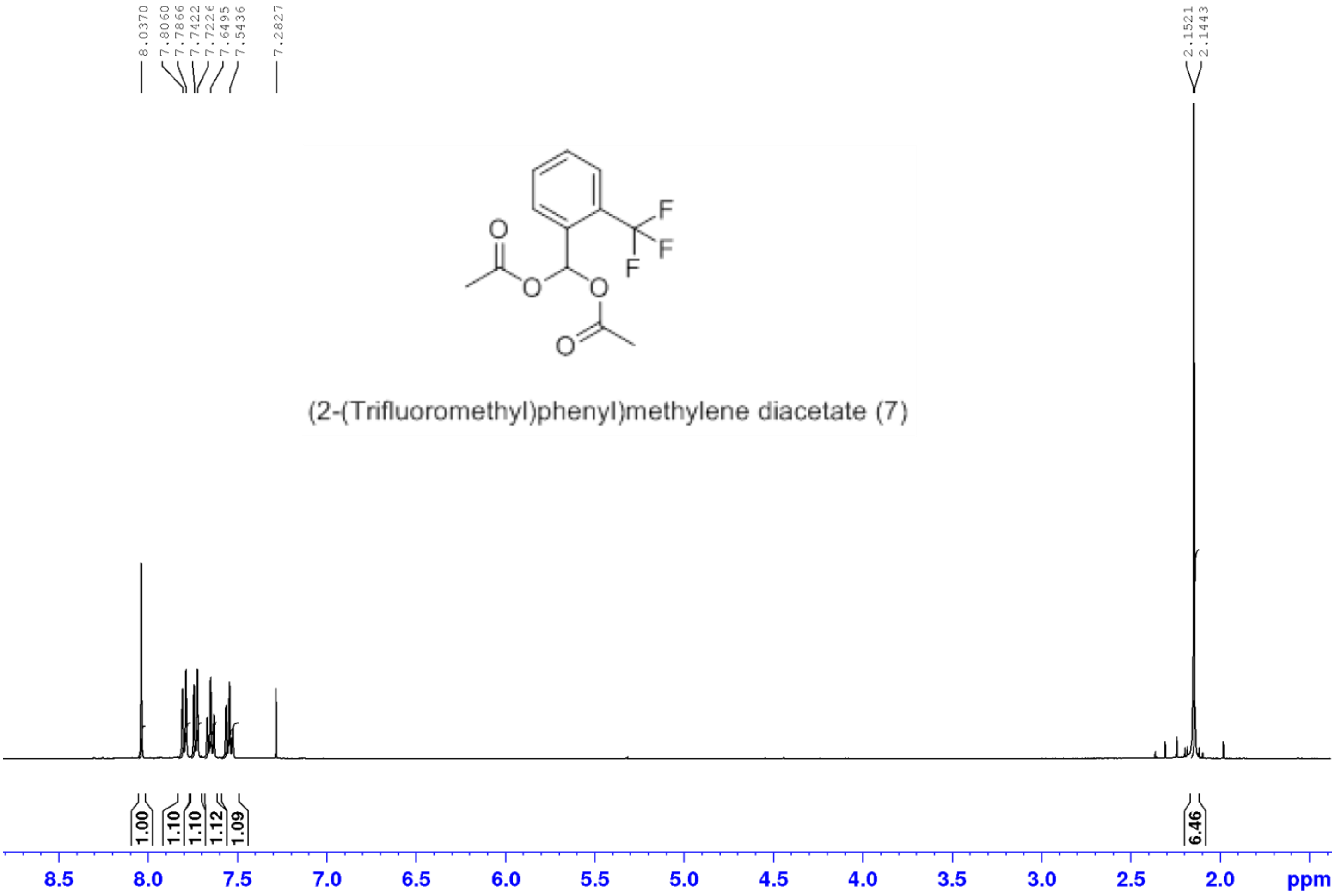
8.0370
7.8060
7.7866
7.7422
7.7226
7.6495
7.5436
7.2827
2.1521
2.1443
(2-(Trifluoromethyl)phenyl)methylene diacetate (7)
1.00
1.10
1.10
1.12
1.09
6.46
8.5
8.0
7.5
7.0
6.5
6.0
5.5
5.0
4.5
4.0
3.5
3.0
2.5
2.0
ppm

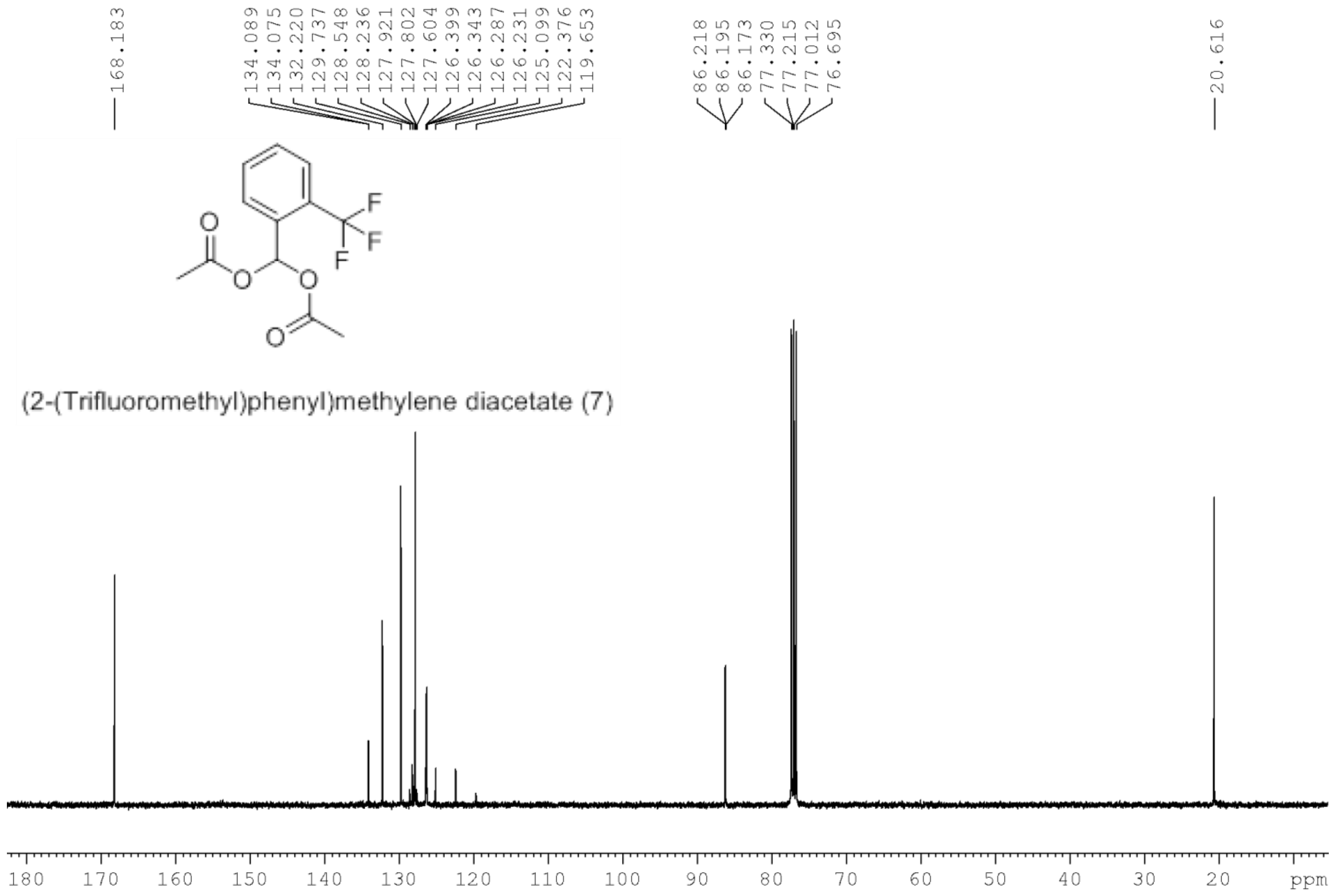
168.183
134.089
134.075
132.220
129.737
128.548
128.236
127.921
127.802
127.604
126.399
126.343
126.287
126.231
125.099
122.376
119.653
86.218
86.195
86.173
77.330
77.215
77.012
76.695
20.616
F
F
F
O
O
O
O
(2-(Trifluoromethyl)phenyl)methylene diacetate (7)
180
170
160
150
140
130
120
110
100
90
80
70
60
50
40
30
20
ppm

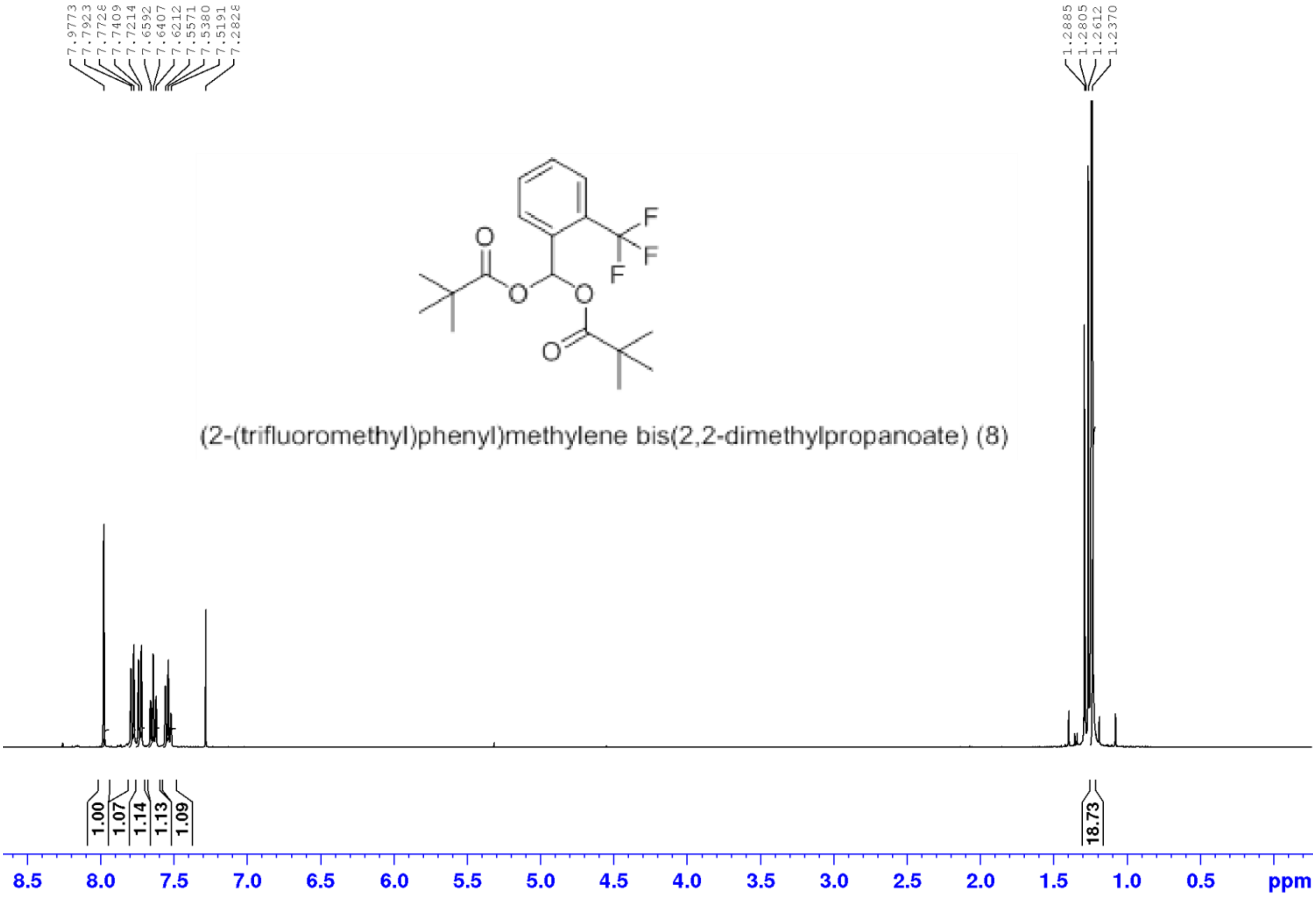

(2-(trifluoromethyl)phenyl)methylene bis(2,2-dimethylpropanoate) (8)

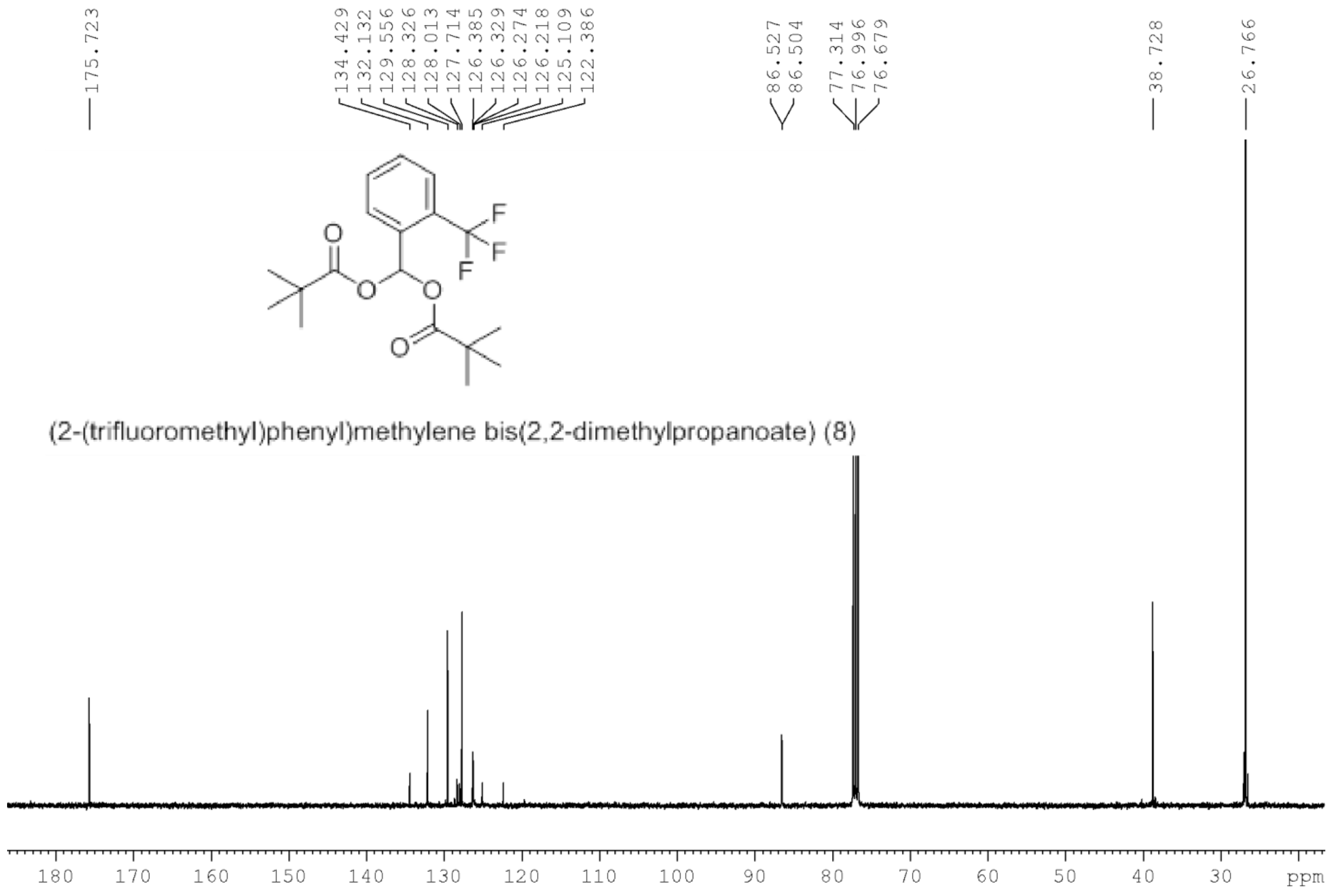

(2-(trifluoromethyl)phenyl)methylene bis(2,2-dimethylpropanoate) (8)

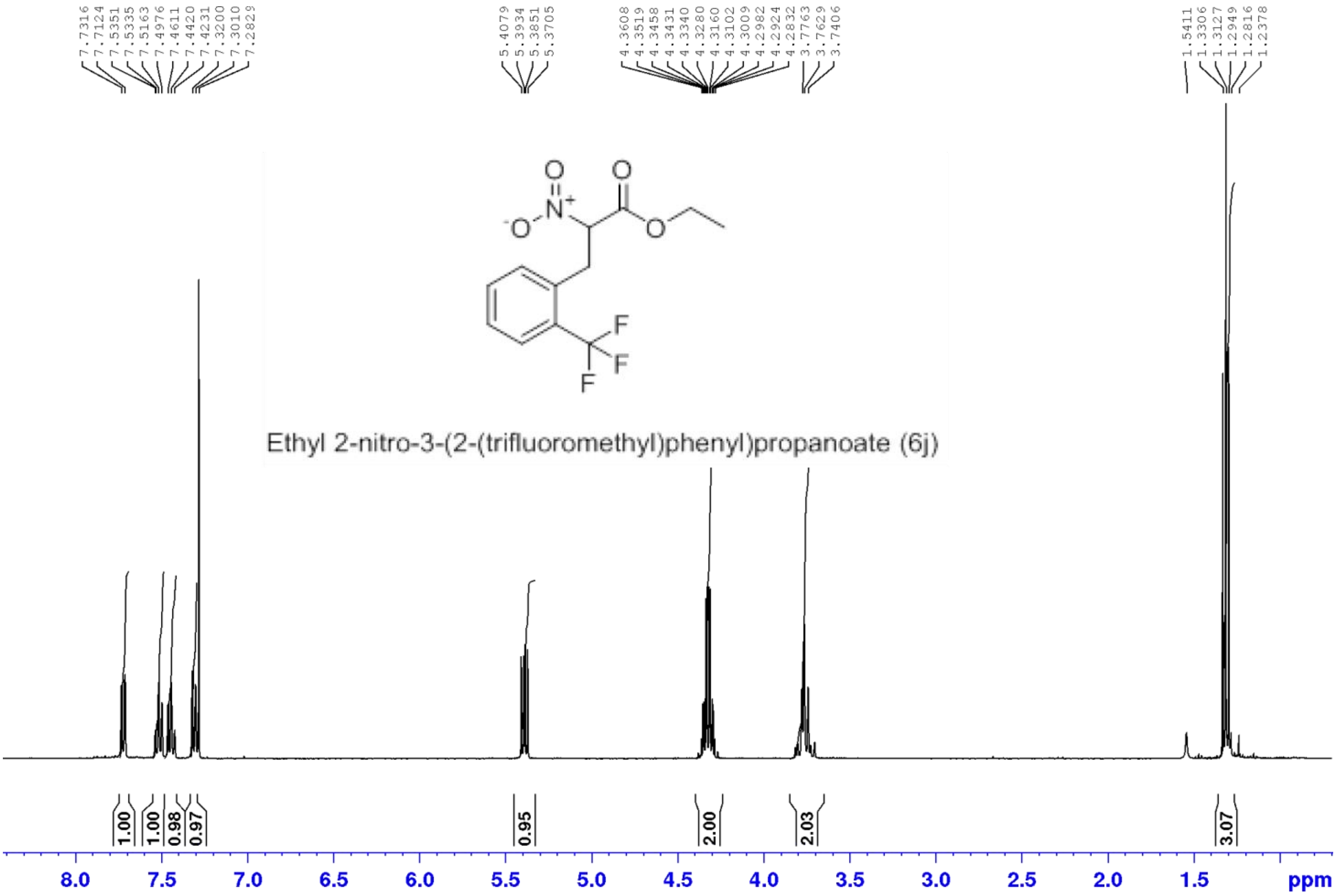

Ethyl 2-nitro-3-(2-(trifluoromethyl)phenyl)propanoate (6j)

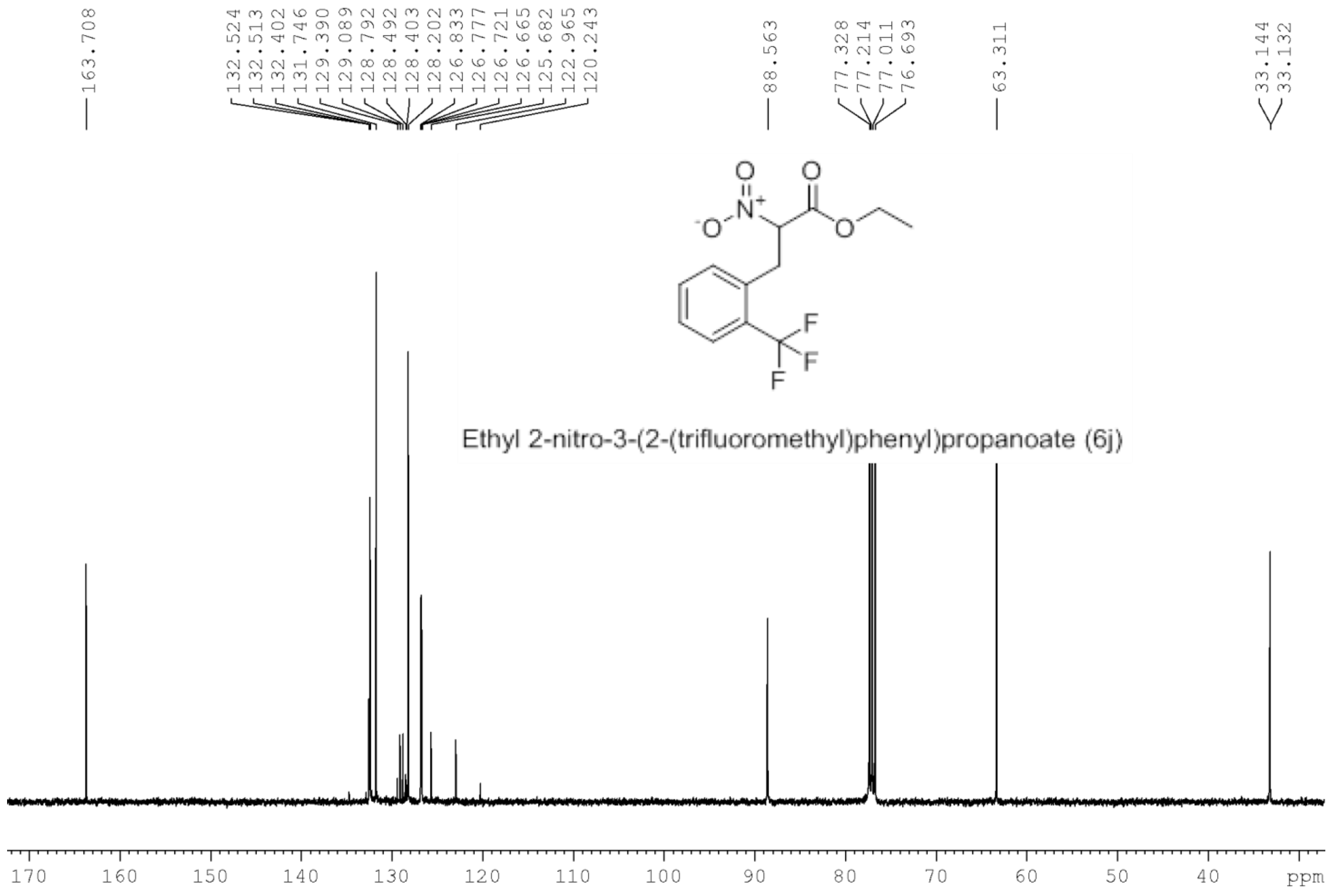
163.708
132.524
132.513
132.402
131.746
129.390
129.089
128.792
128.492
128.403
128.202
126.833
126.777
126.721
126.665
125.682
122.965
120.243
88.563
77.328
77.214
77.011
76.693
63.311
33.144
33.132
Ethyl 2-nitro-3-(2-(trifluoromethyl)phenyl)propanoate (6j)
170
160
150
140
130
120
110
100
90
80
70
60
50
40
ppm

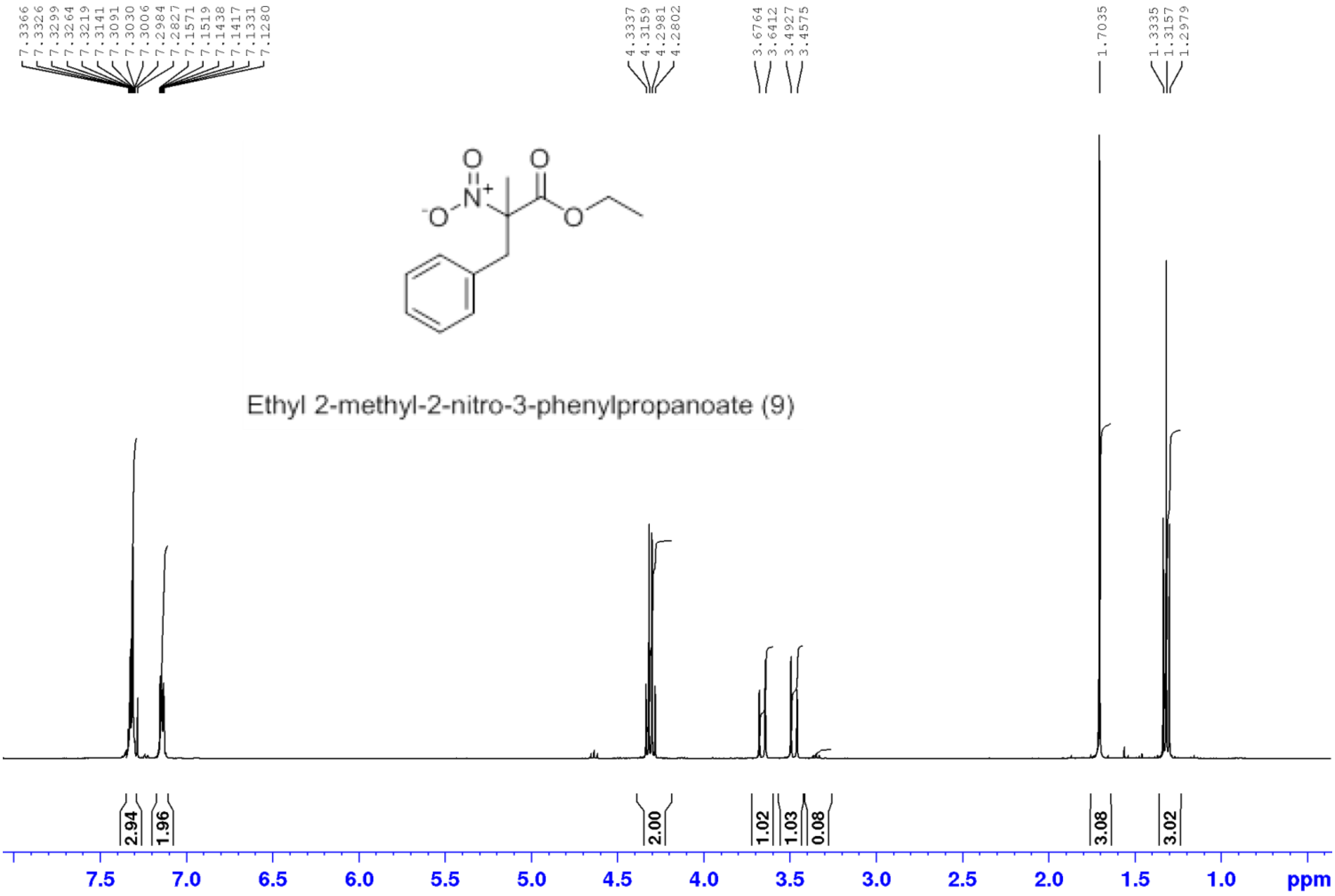
7.3366
7.3326
7.3299
7.3264
7.3219
7.3141
7.3091
7.3030
7.3006
7.2984
7.2827
7.1571
7.1519
7.1438
7.1417
7.1331
7.1280
4.3337
4.3159
4.2981
4.2802
3.6764
3.6412
3.4927
3.4575
1.7035
1.3335
1.3157
1.2979
Ethyl 2-methyl-2-nitro-3-phenylpropanoate (9)
2.94
1.96
2.00
1.02
1.03
0.08
3.08
3.02
7.5
7.0
6.5
6.0
5.5
5.0
4.5
4.0
3.5
3.0
2.5
2.0
1.5
1.0
ppm

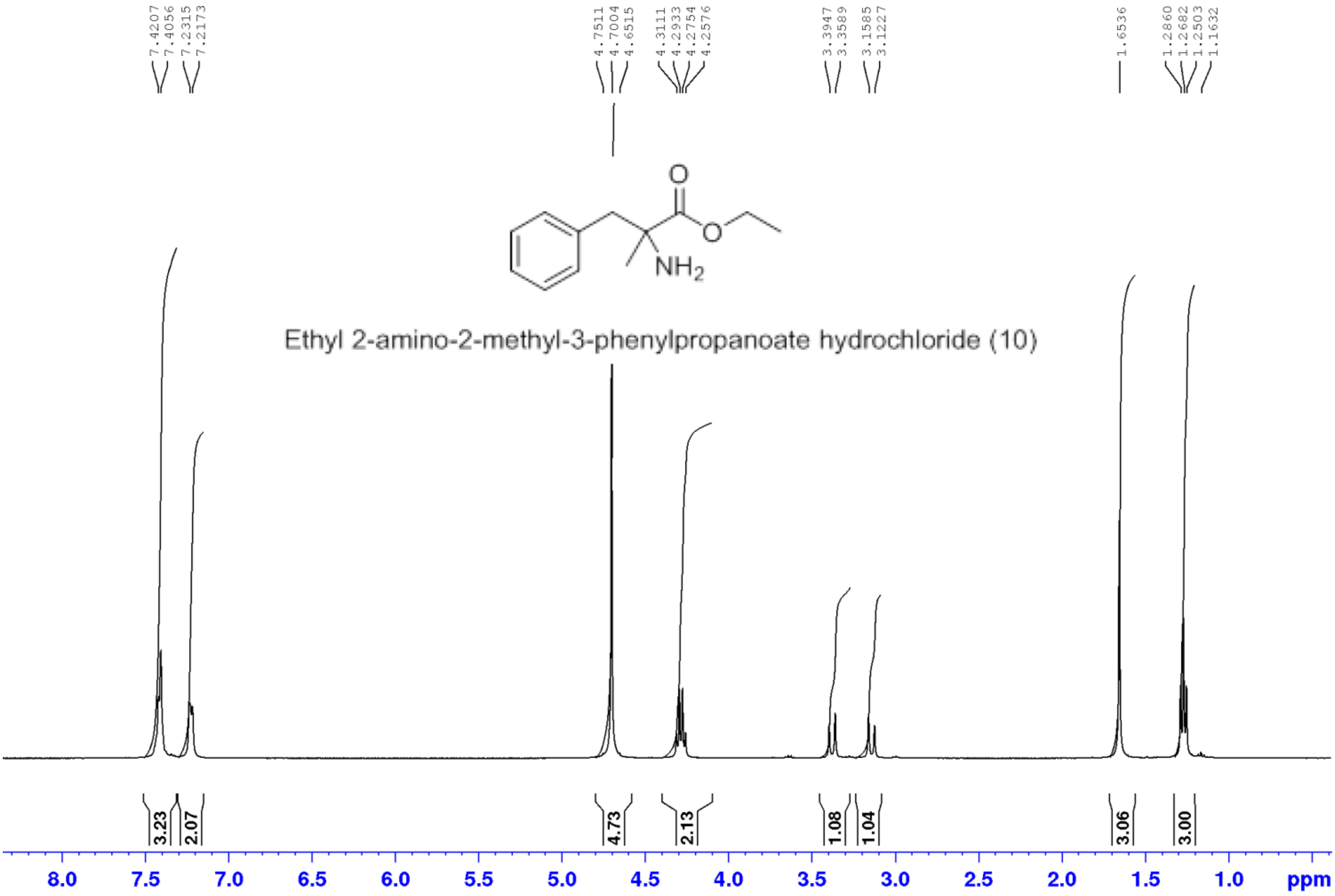

Ethyl 2-amino-2-methyl-3-phenylpropanoate hydrochloride (10)

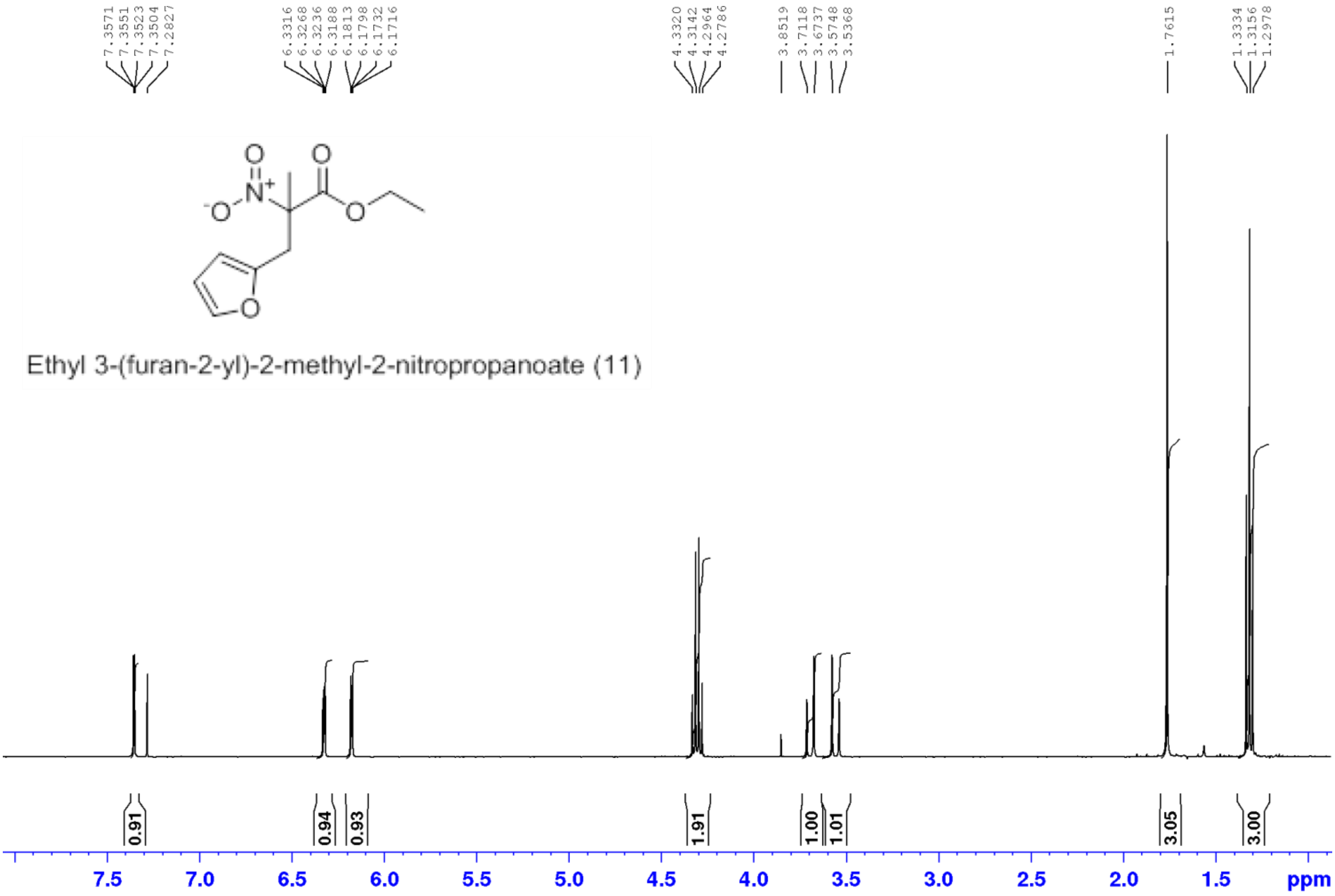
Ethyl 3-(furan-2-yl)-2-methyl-2-nitropropanoate (11)
7.3571
7.3551
7.3523
7.3504
7.2827
6.3316
6.3268
6.3236
6.3188
6.1813
6.1798
6.1732
6.1716
4.3320
4.3142
4.2964
4.2786
3.8519
3.7118
3.6737
3.5748
3.5368
1.7615
1.3334
1.3156
1.2978
0.91
0.94
0.93
1.91
1.00
1.01
3.05
3.00
7.5
7.0
6.5
6.0
5.5
5.0
4.5
4.0
3.5
3.0
2.5
2.0
1.5
ppm

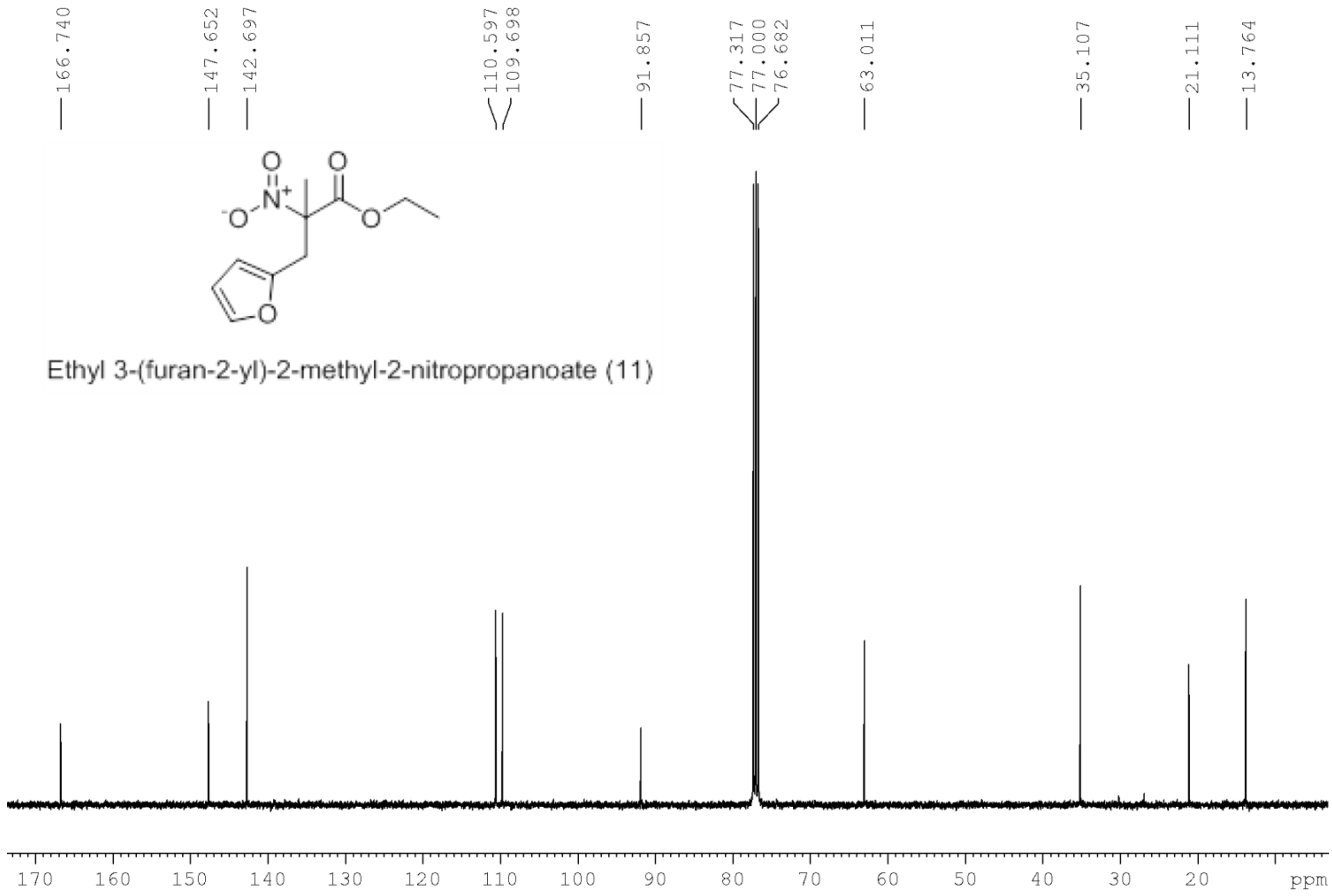
166.740
147.652
142.697
110.597
109.698
91.857
77.317
77.000
76.682
63.011
35.107
21.111
13.764
Ethyl 3-(furan-2-yl)-2-methyl-2-nitropropanoate (11)
170
160
150
140
130
120
110
100
90
80
70
60
50
40
30
20
ppm

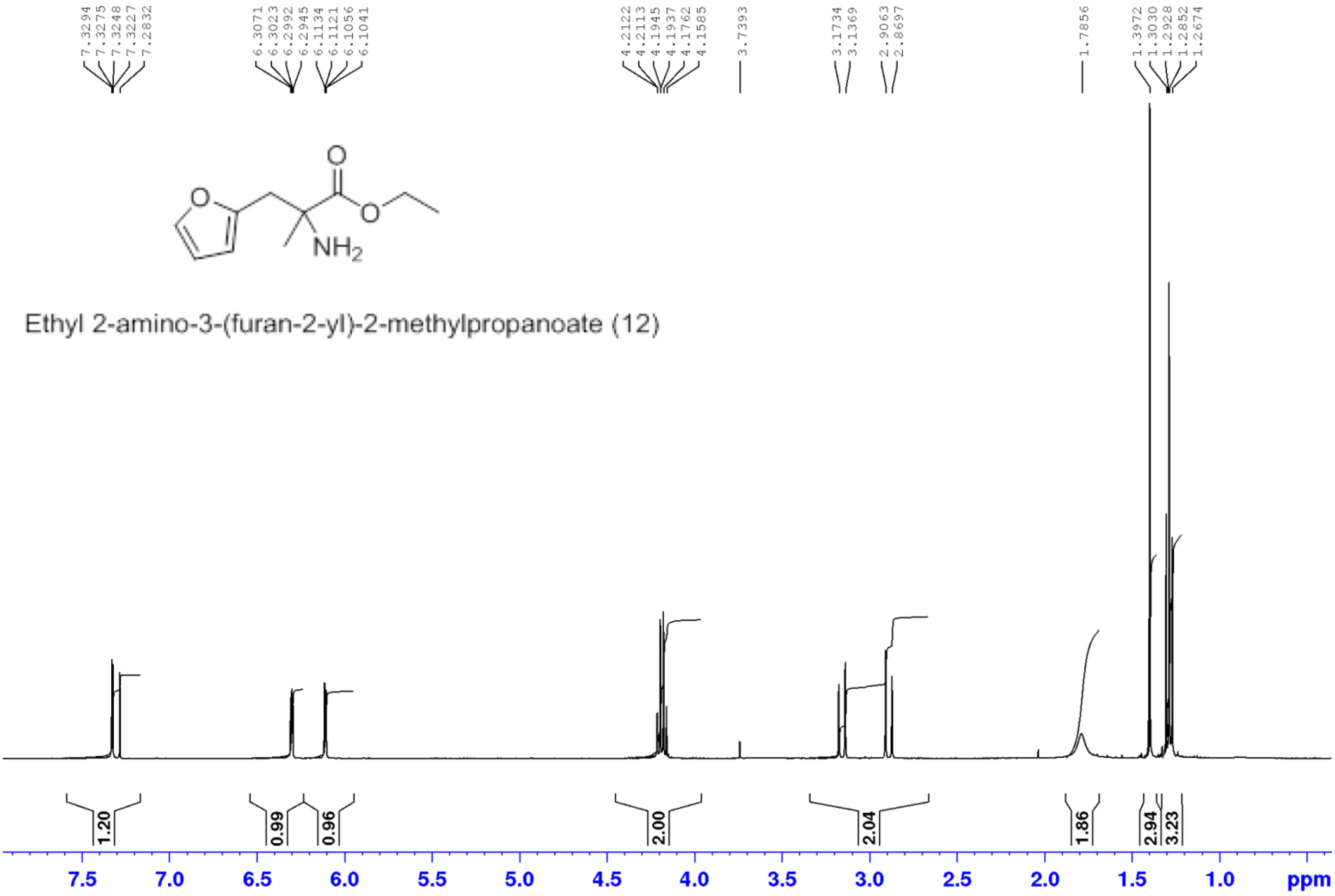

Ethyl 2-amino-3-(furan-2-yl)-2-methylpropanoate (12)

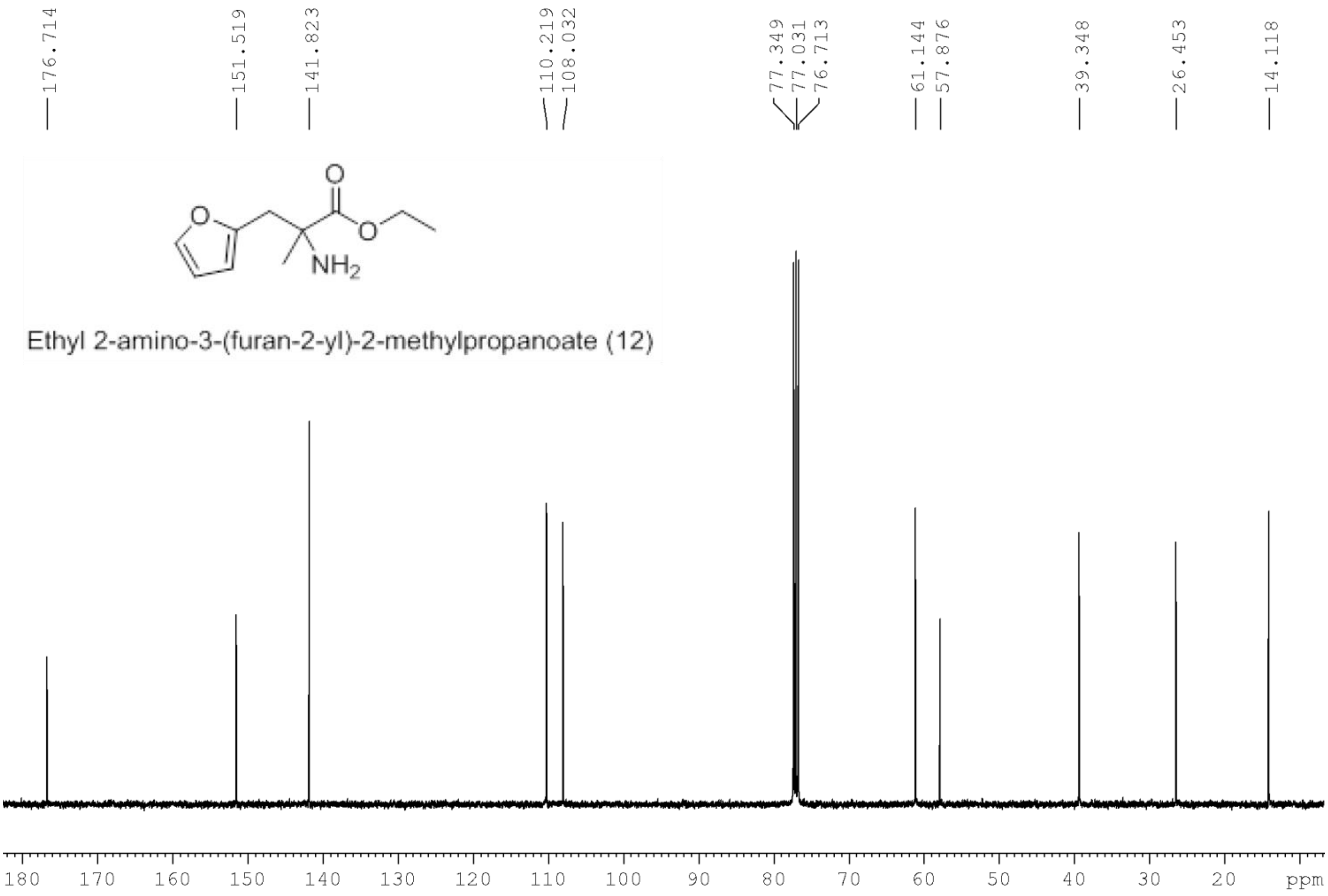

Ethyl 2-amino-3-(furan-2-yl)-2-methylpropanoate (12)

Ethyl 3-(furan-2-yl)-2-nitrobutanoate (13)

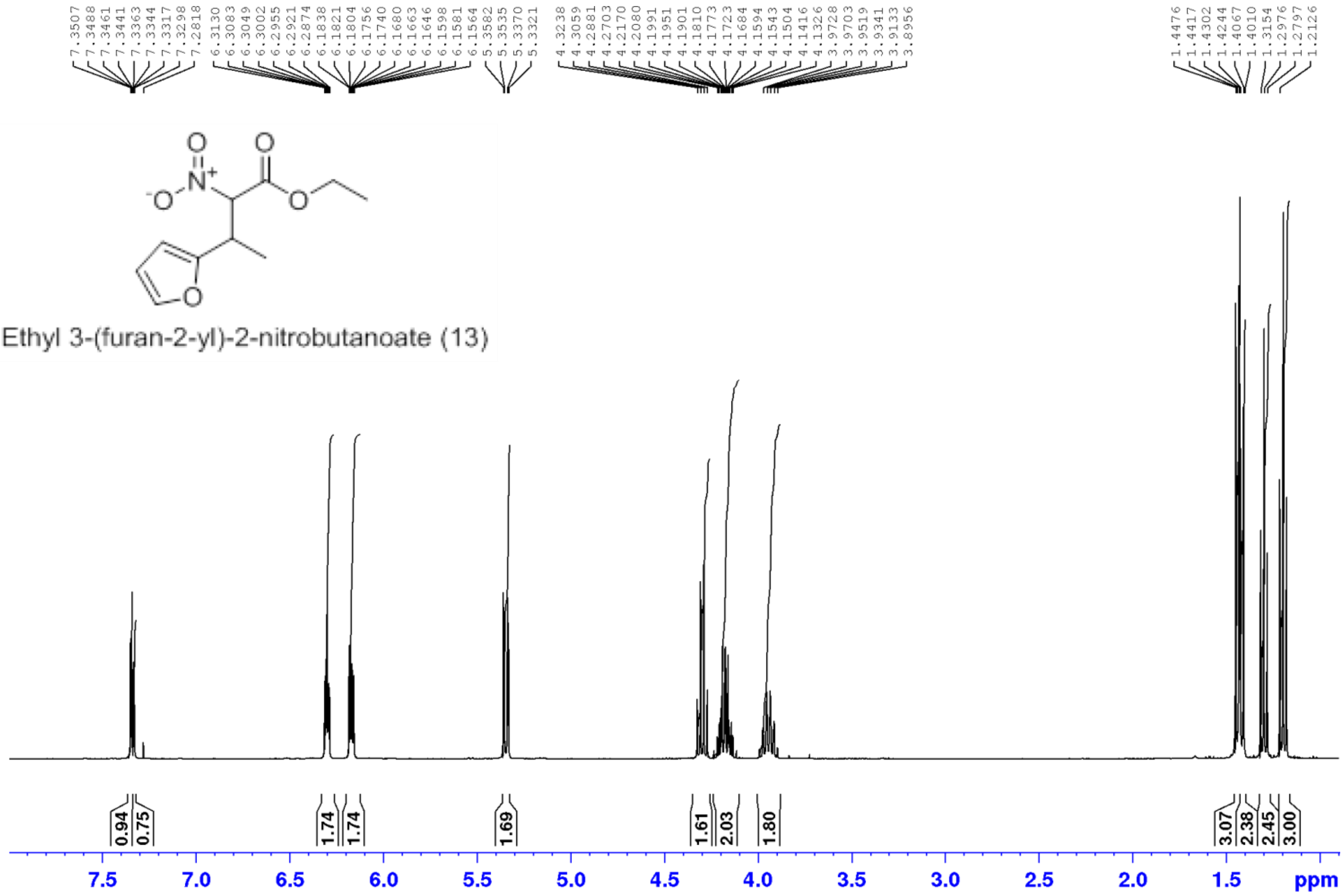

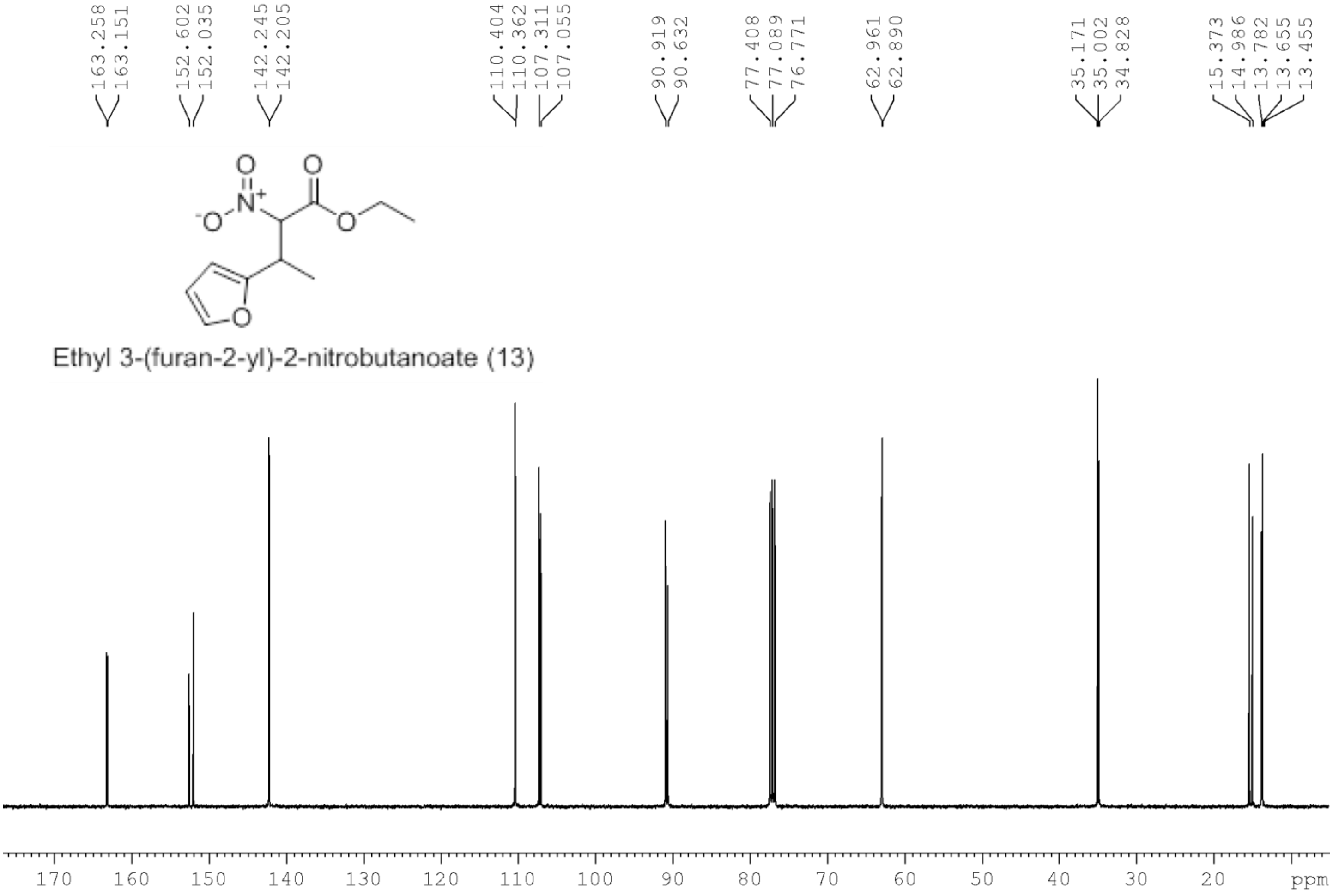
163.258
163.151
152.602
152.035
142.245
142.205
110.404
110.362
107.311
107.055
90.919
90.632
77.408
77.089
76.771
62.961
62.890
35.171
35.002
34.828
15.373
14.986
13.782
13.655
13.455
Ethyl 3-(furan-2-yl)-2-nitrobutanoate (13)
170
160
150
140
130
120
110
100
90
80
70
60
50
40
30
20
ppm

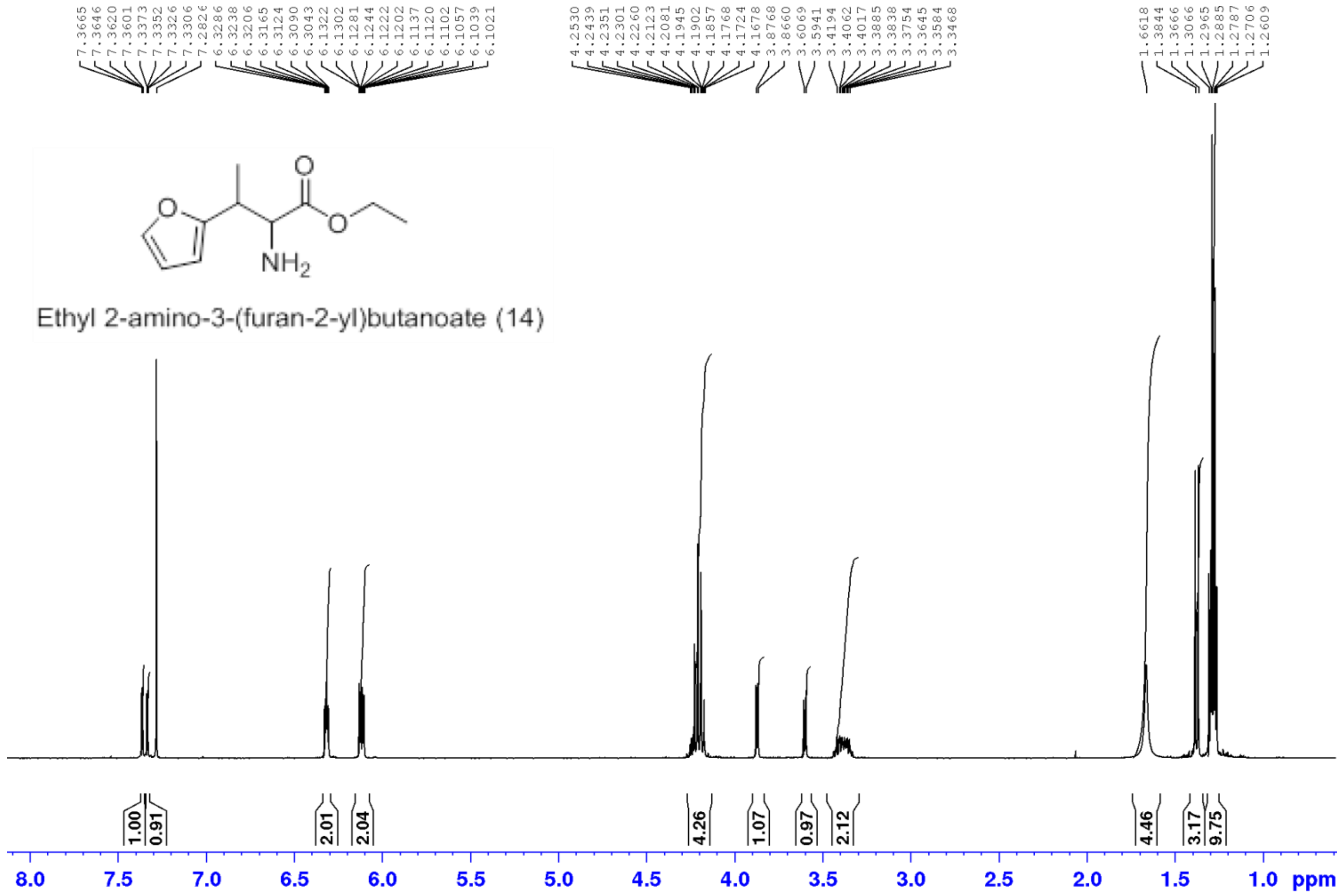

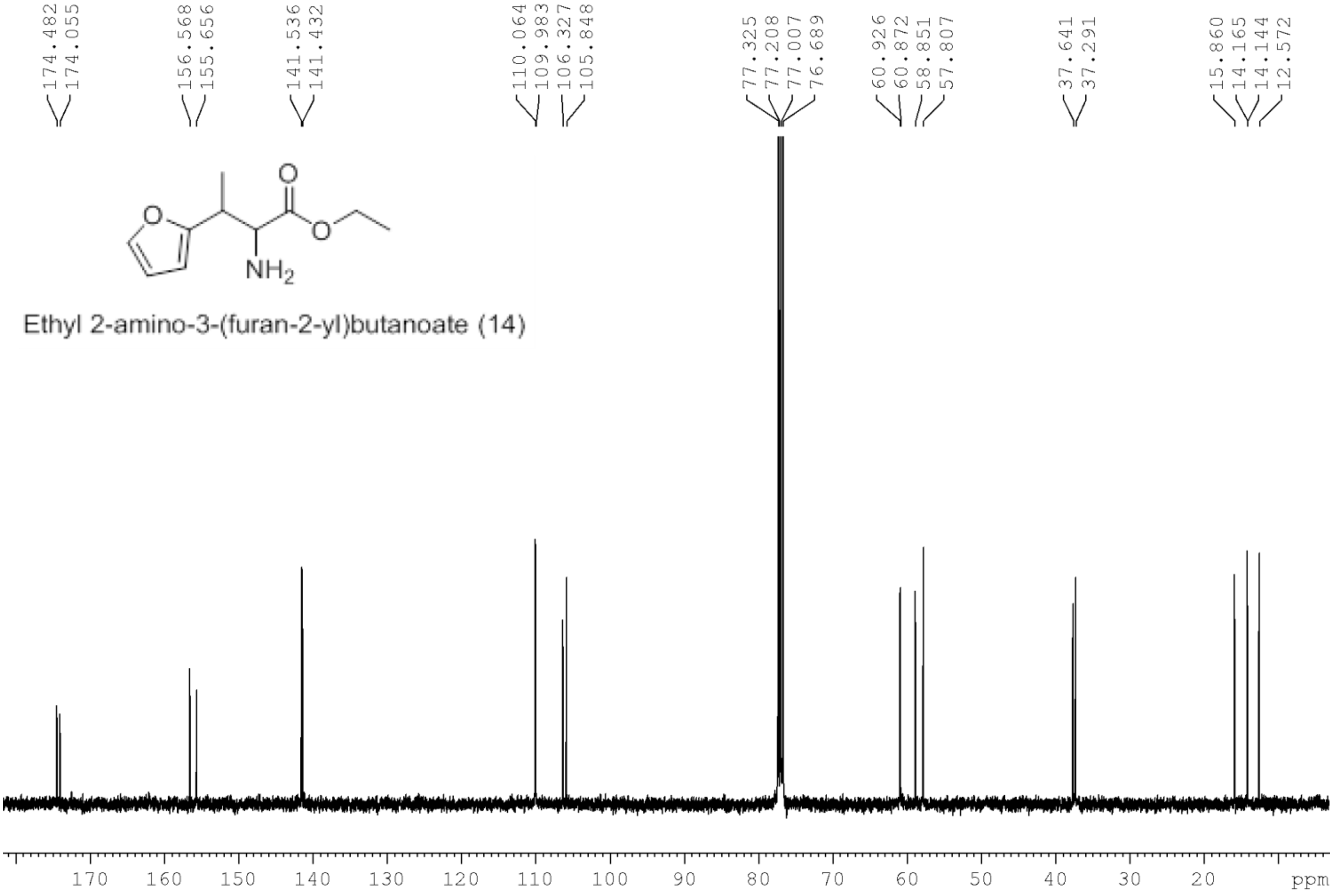

174.482
174.055
156.568
155.656
141.536
141.432
110.064
109.983
106.327
105.848
77.325
77.208
77.007
76.689
60.926
60.872
58.851
57.807
37.641
37.291
15.860
14.165
14.144
12.572
O
O
O
NH2
Ethyl 2-amino-3-(furan-2-yl)butanoate (14)
170
160
150
140
130
120
110
100
90
80
70
60
50
40
30
20
ppm

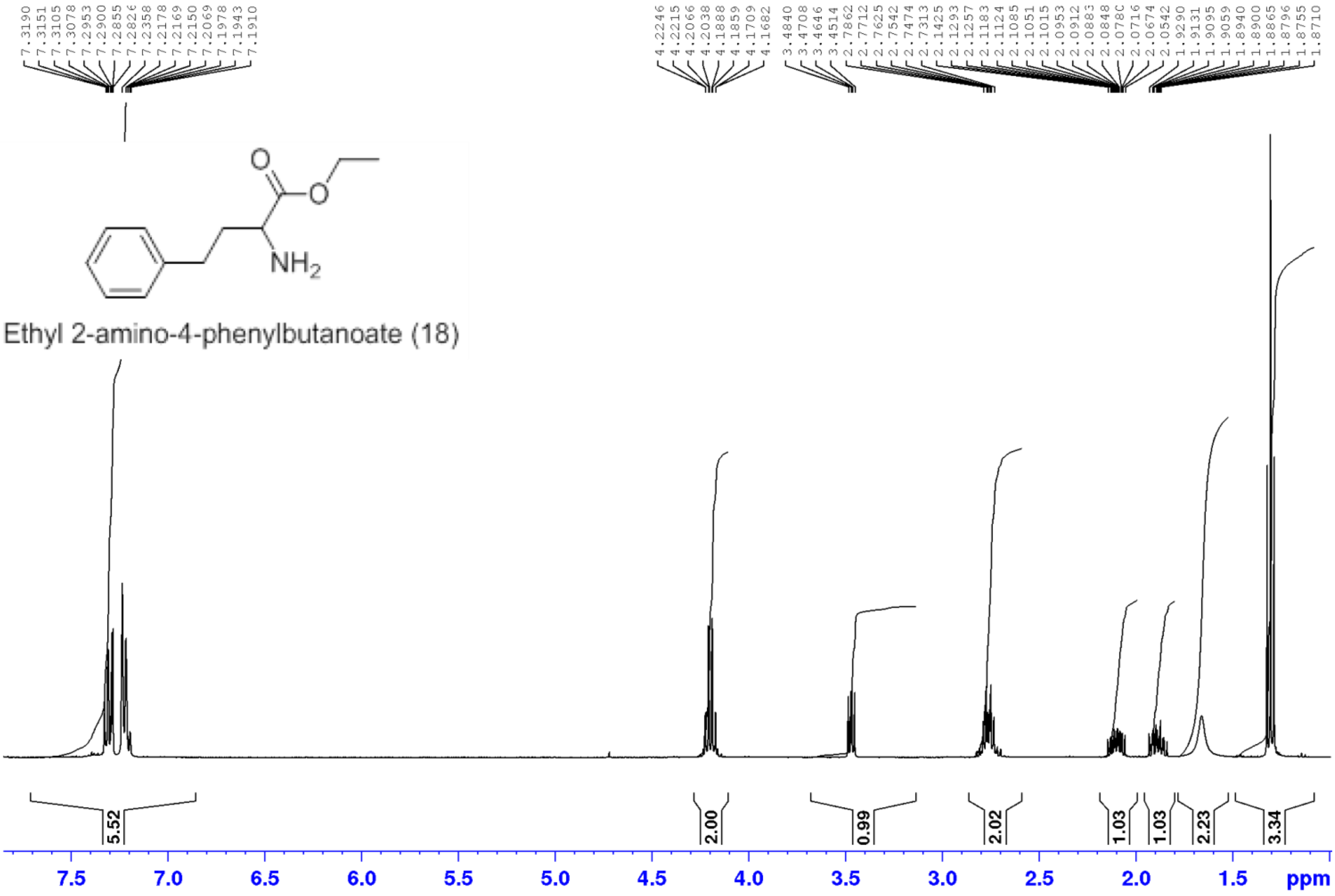
7.3190 7.3151 7.3105 7.3078 7.2953 7.2900 7.2855 7.2826 7.2358 7.2178 7.2169 7.2150 7.2069 7.1978 7.1943 7.1910
4.2246 4.2215 4.2066 4.2038 4.1888 4.1859 4.1709 4.1682
3.4840 3.4708 3.4646 3.4514 2.7862 2.7712 2.7625 2.7542 2.7474 2.7313 2.1425 2.1293 2.1257 2.1183 2.1124 2.1085 2.1051 2.1015 2.0953 2.0912 2.0883 2.0848 2.0780 2.0716 2.0674 2.0542 1.9290 1.9131 1.9095 1.9059 1.8940 1.8900 1.8865 1.8796 1.8755 1.8710
NH2
Ethyl 2-amino-4-phenylbutanoate (18)
5.52
2.00
0.99
2.02
1.03
1.03
2.23
3.34
7.5 7.0 6.5 6.0 5.5 5.0 4.5 4.0 3.5 3.0 2.5 2.0 1.5 ppm

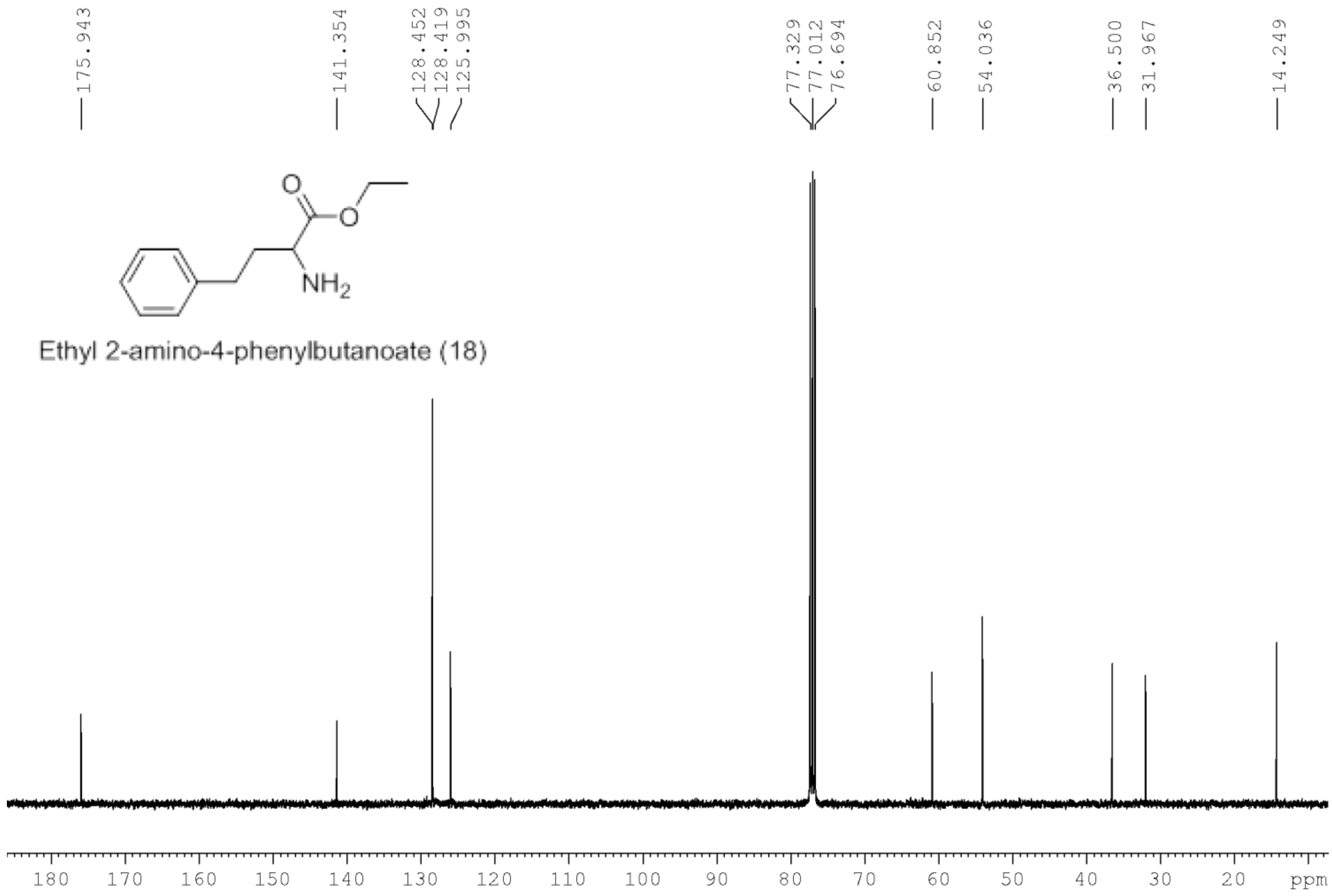
175.943
141.354
128.452
128.419
125.995
77.329
77.012
76.694
60.852
54.036
36.500
31.967
14.249
Ethyl 2-amino-4-phenylbutanoate (18)
180
170
160
150
140
130
120
110
100
90
80
70
60
50
40
30
20
ppm

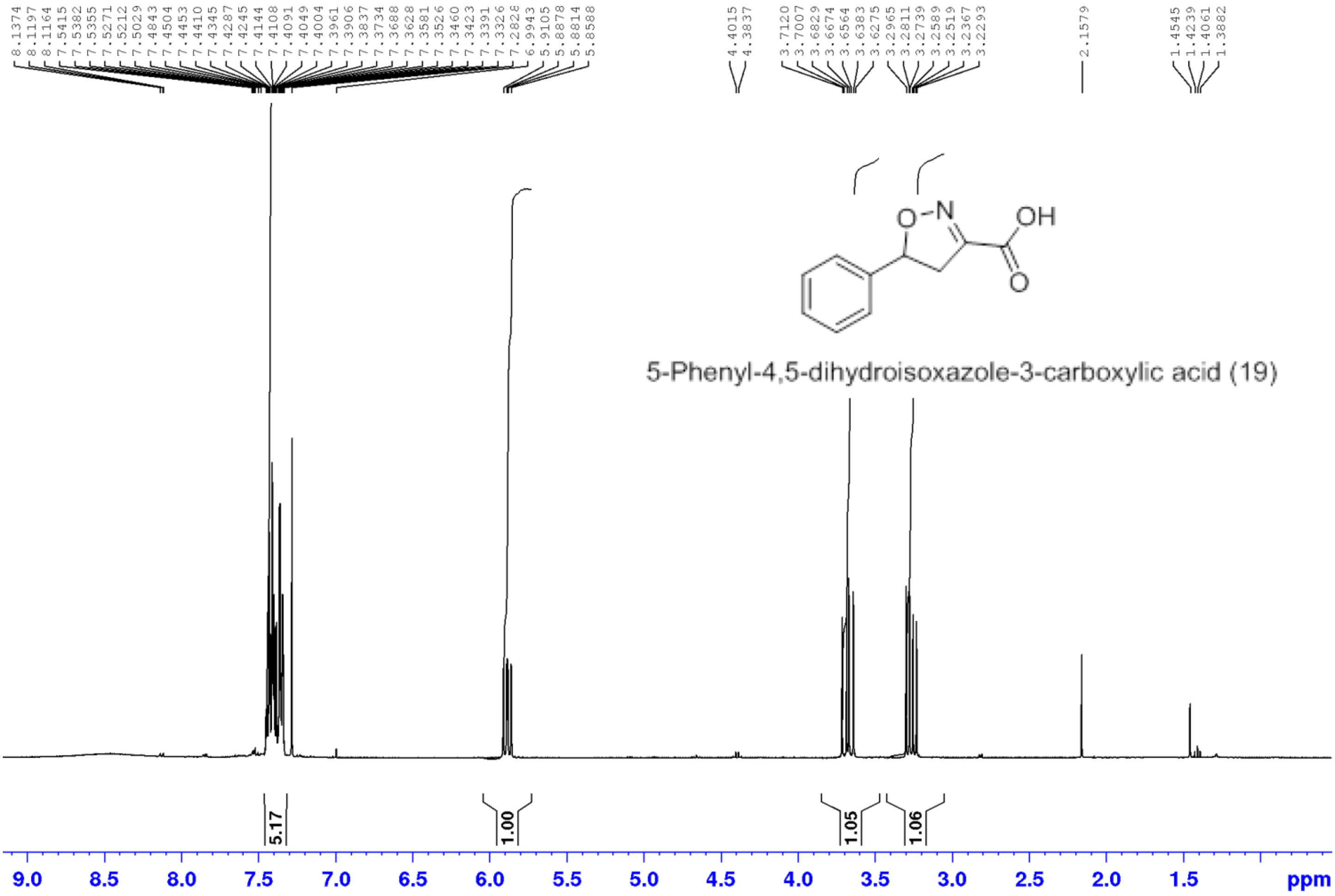

5-Phenyl-4,5-dihydroisoxazole-3-carboxylic acid (19)

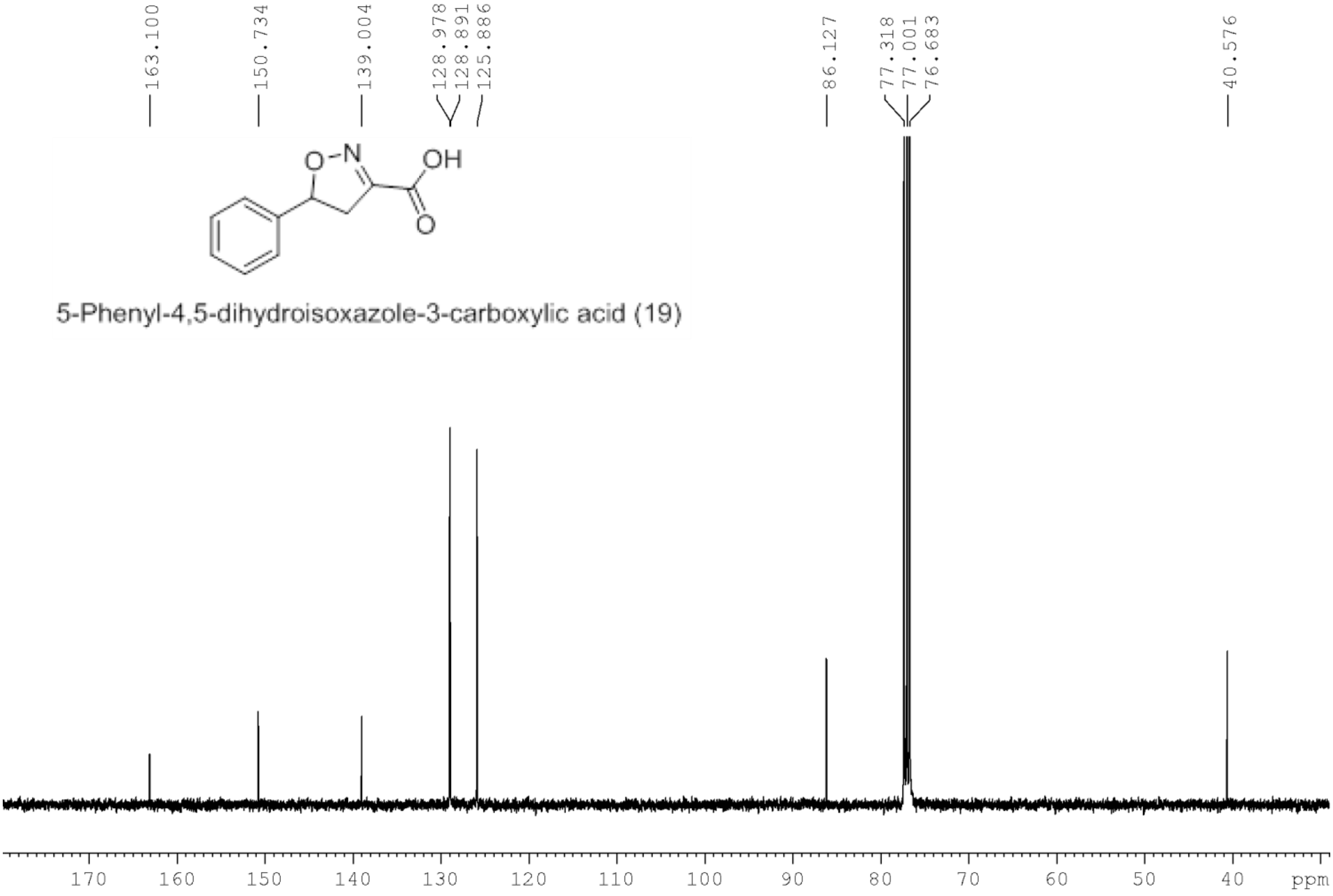

5-Phenyl-4,5-dihydroisoxazole-3-carboxylic acid (19)

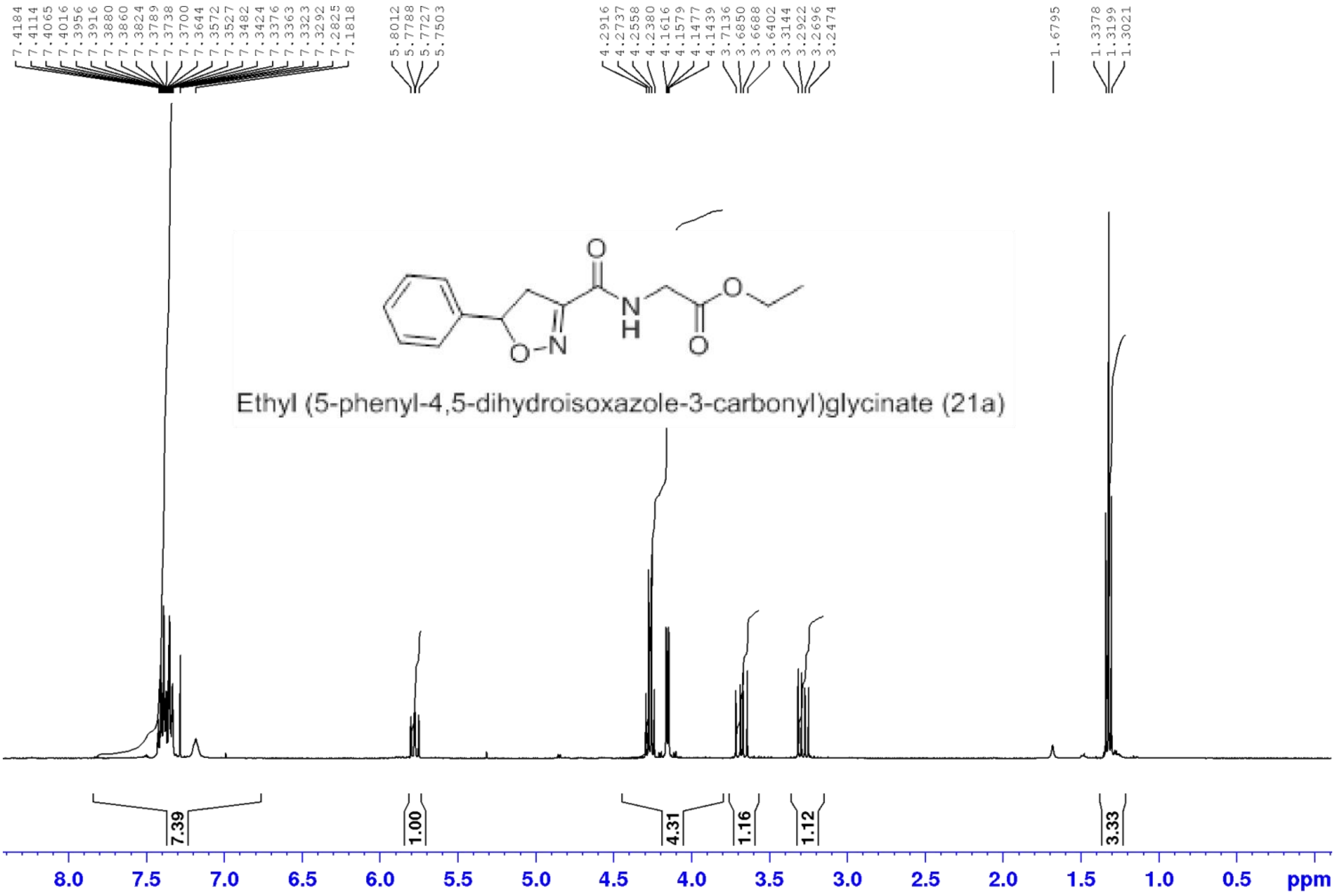
Ethyl (5-phenyl-4,5-dihydroisoxazole-3-carbonyl)glycinate (21a)
7.4184 7.4114 7.4065 7.4016 7.3956 7.3916 7.3880 7.3860 7.3824 7.3789 7.3738 7.3700 7.3644 7.3572 7.3527 7.3482 7.3424 7.3376 7.3363 7.3323 7.3292 7.2825 7.1818
5.8012 5.7788 5.7727 5.7503
4.2916 4.2737 4.2558 4.2380 4.1616 4.1579 4.1477 4.1439 3.7136 3.6850 3.6688 3.6402 3.3144 3.2922 3.2696 3.2474
1.6795
1.3378 1.3199 1.3021
7.39
1.00
4.31
1.16
1.12
3.33
8.0 7.5 7.0 6.5 6.0 5.5 5.0 4.5 4.0 3.5 3.0 2.5 2.0 1.5 1.0 0.5 ppm

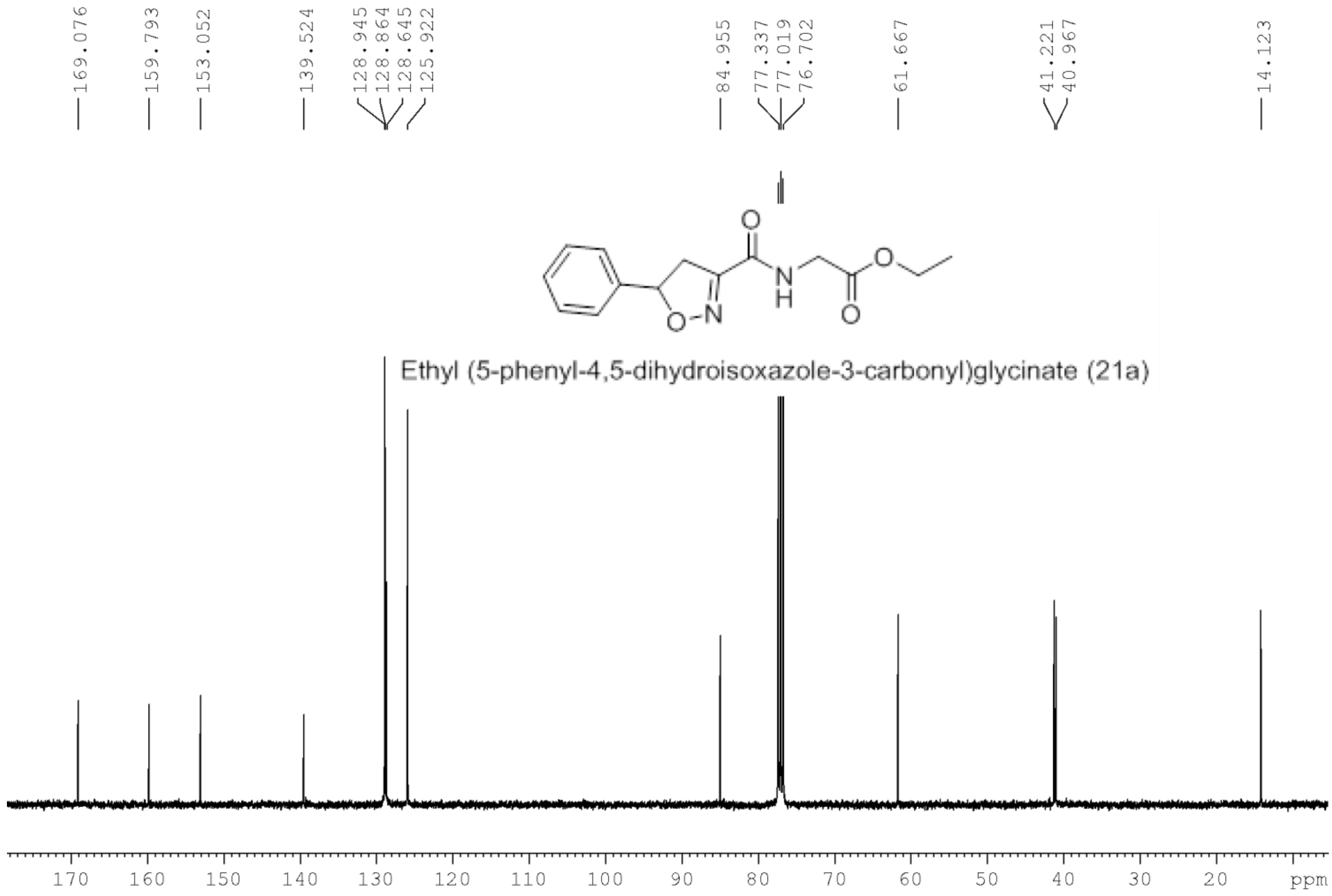

169.076
159.793
153.052
139.524
128.945
128.864
128.645
125.922
84.955
77.337
77.019
76.702
61.667
41.221
40.967
14.123
O
N
H
O
O
O-N
Ethyl (5-phenyl-4,5-dihydroisoxazole-3-carbonyl)glycinate (21a)
170
160
150
140
130
120
110
100
90
80
70
60
50
40
30
20
ppm

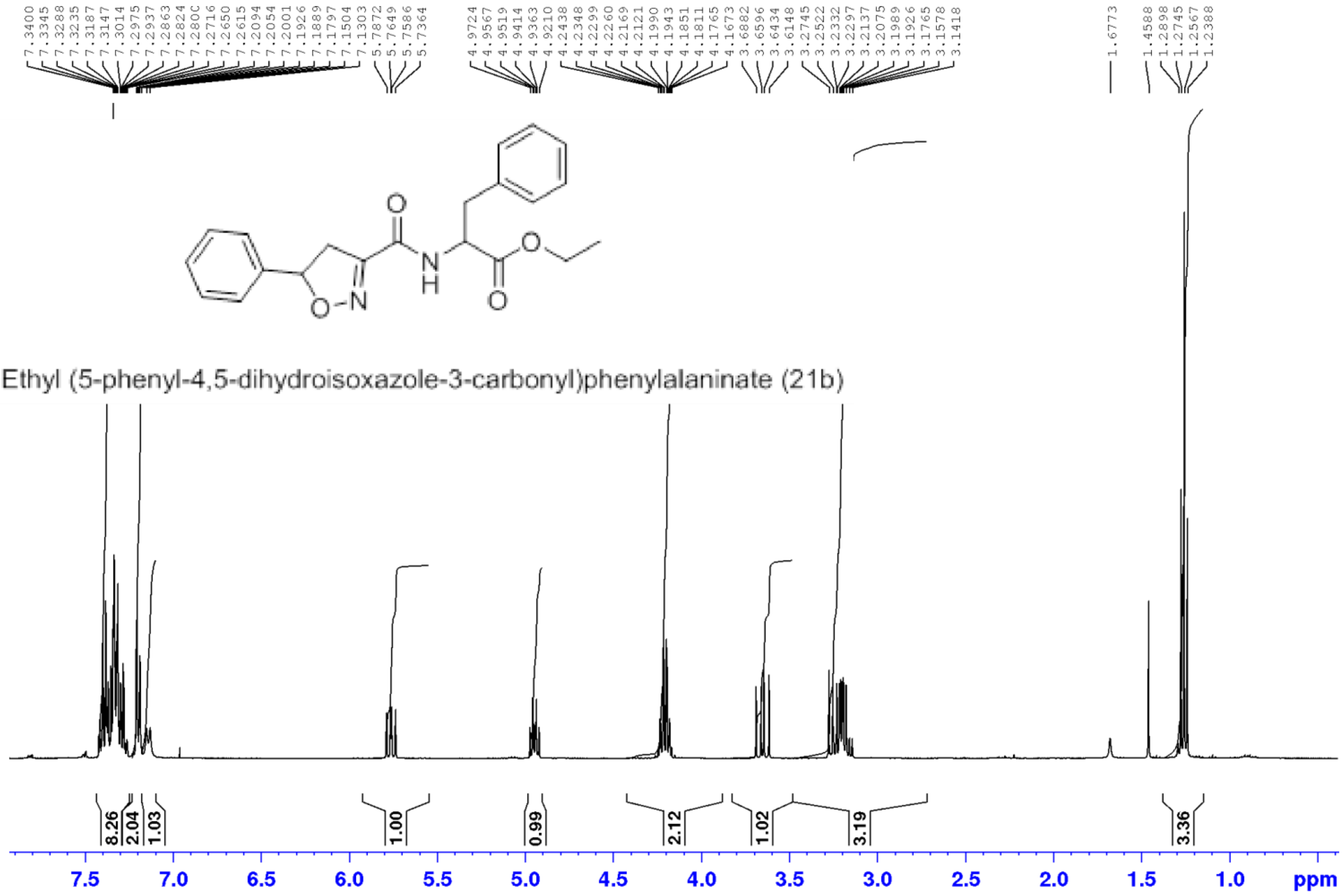
7.3400 7.3345 7.3288 7.3235 7.3187 7.3147 7.3014 7.2975 7.2937 7.2863 7.2824 7.280C 7.2716 7.2650 7.2615 7.2094 7.2054 7.2001 7.1926 7.1889 7.1797 7.1504 7.1303 5.7872 5.7649 5.7586 5.7364
4.9724 4.9567 4.9519 4.9414 4.9363 4.9210 4.2438 4.2348 4.2299 4.2260 4.2169 4.2121 4.1990 4.1943 4.1851 4.1811 4.1765 4.1673 3.6882 3.6596 3.6434 3.6148 3.2745 3.2522 3.2332 3.2297 3.2137 3.2075 3.1989 3.1926 3.1765 3.1578 3.1418
1.6773 1.4588 1.2898 1.2745 1.2567 1.2388
O
N
H
O
O
O
N
Ethyl (5-phenyl-4,5-dihydroisoxazole-3-carbonyl)phenylalaninate (21b)
8.26
2.04
1.03
1.00
0.99
2.12
1.02
3.19
3.36
7.5
7.0
6.5
6.0
5.5
5.0
4.5
4.0
3.5
3.0
2.5
2.0
1.5
1.0
ppm

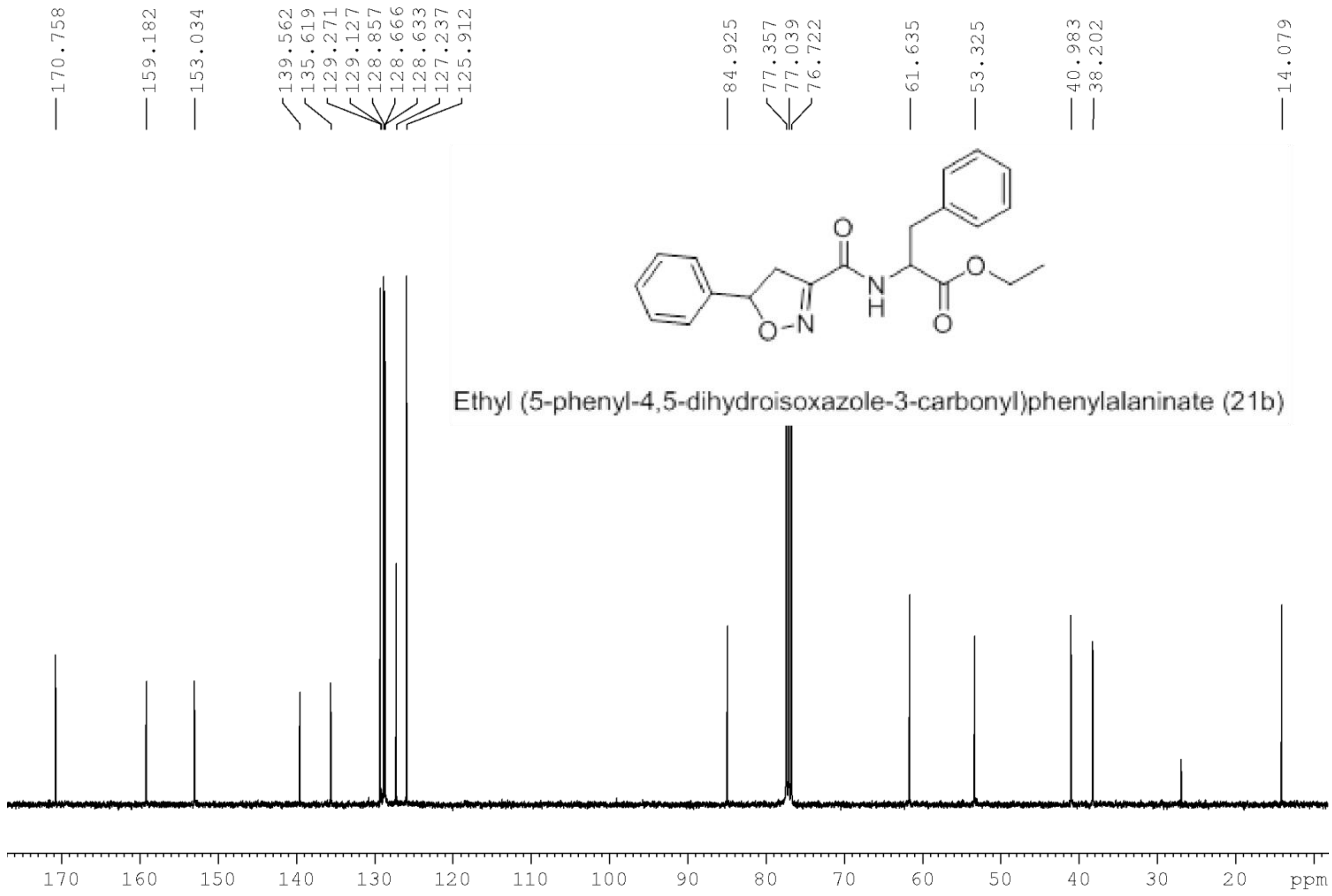
170.758
159.182
153.034
139.562
135.619
129.271
129.127
128.857
128.666
128.633
127.237
125.912
84.925
77.357
77.039
76.722
61.635
53.325
40.983
38.202
14.079
O
N
H
O
O
O–N
Ethyl (5-phenyl-4,5-dihydroisoxazole-3-carbonyl)phenylalaninate (21b)
170
160
150
140
130
120
110
100
90
80
70
60
50
40
30
20
ppm

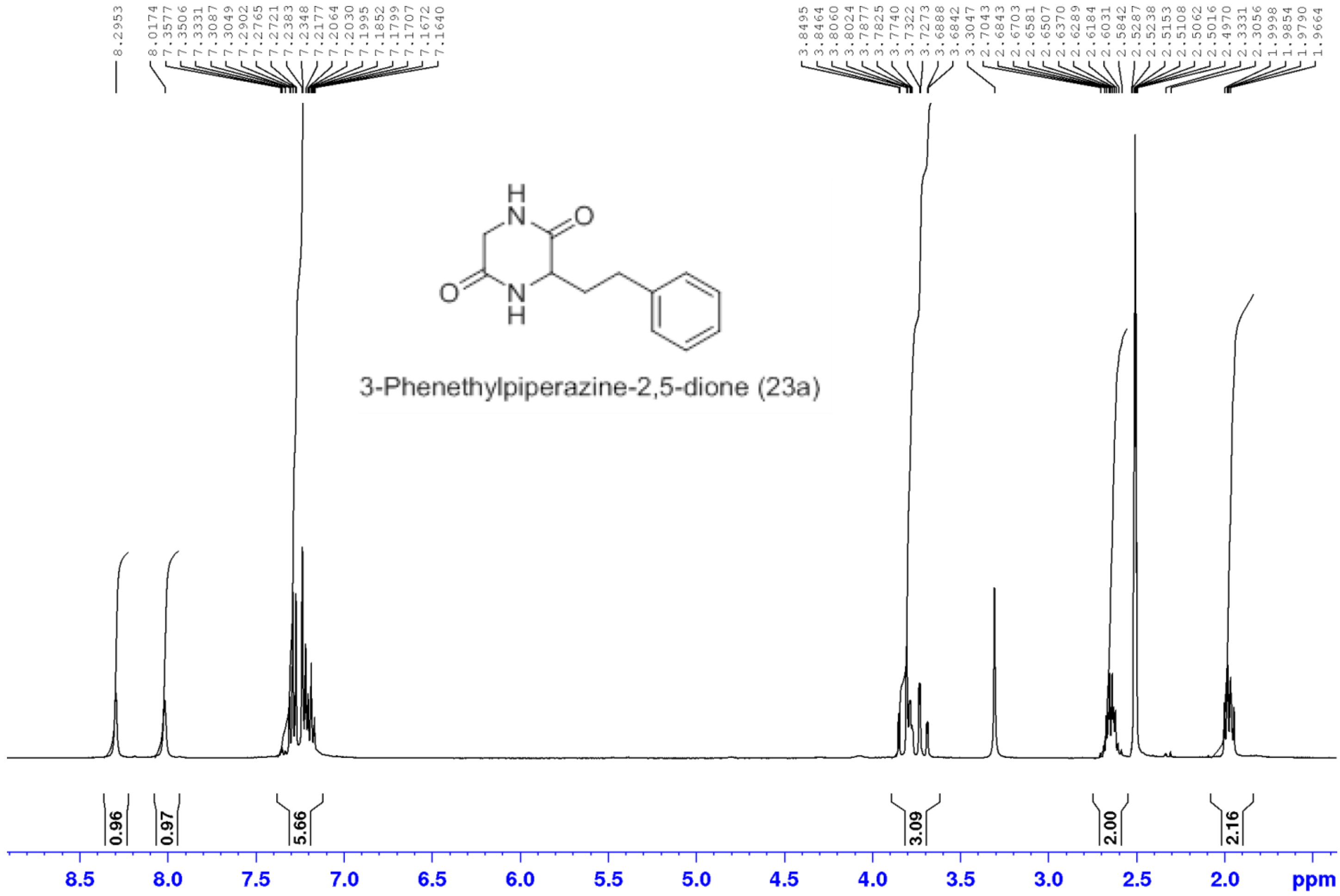
8.2953
8.0174
7.3577
7.3506
7.3331
7.3087
7.3049
7.2902
7.2765
7.2721
7.2383
7.2348
7.2177
7.2064
7.2030
7.1995
7.1852
7.1799
7.1707
7.1672
7.1640
3.8495
3.8464
3.8060
3.8024
3.7877
3.7825
3.7740
3.7322
3.7273
3.6888
3.6842
3.3047
2.7043
2.6843
2.6703
2.6581
2.6507
2.6370
2.6289
2.6184
2.6031
2.5842
2.5287
2.5238
2.5153
2.5108
2.5062
2.5016
2.4970
2.3331
2.3056
1.9998
1.9854
1.9790
1.9664
3-Phenethylpiperazine-2,5-dione (23a)
0.96
0.97
5.66
3.09
2.00
2.16
8.5
8.0
7.5
7.0
6.5
6.0
5.5
5.0
4.5
4.0
3.5
3.0
2.5
2.0
ppm

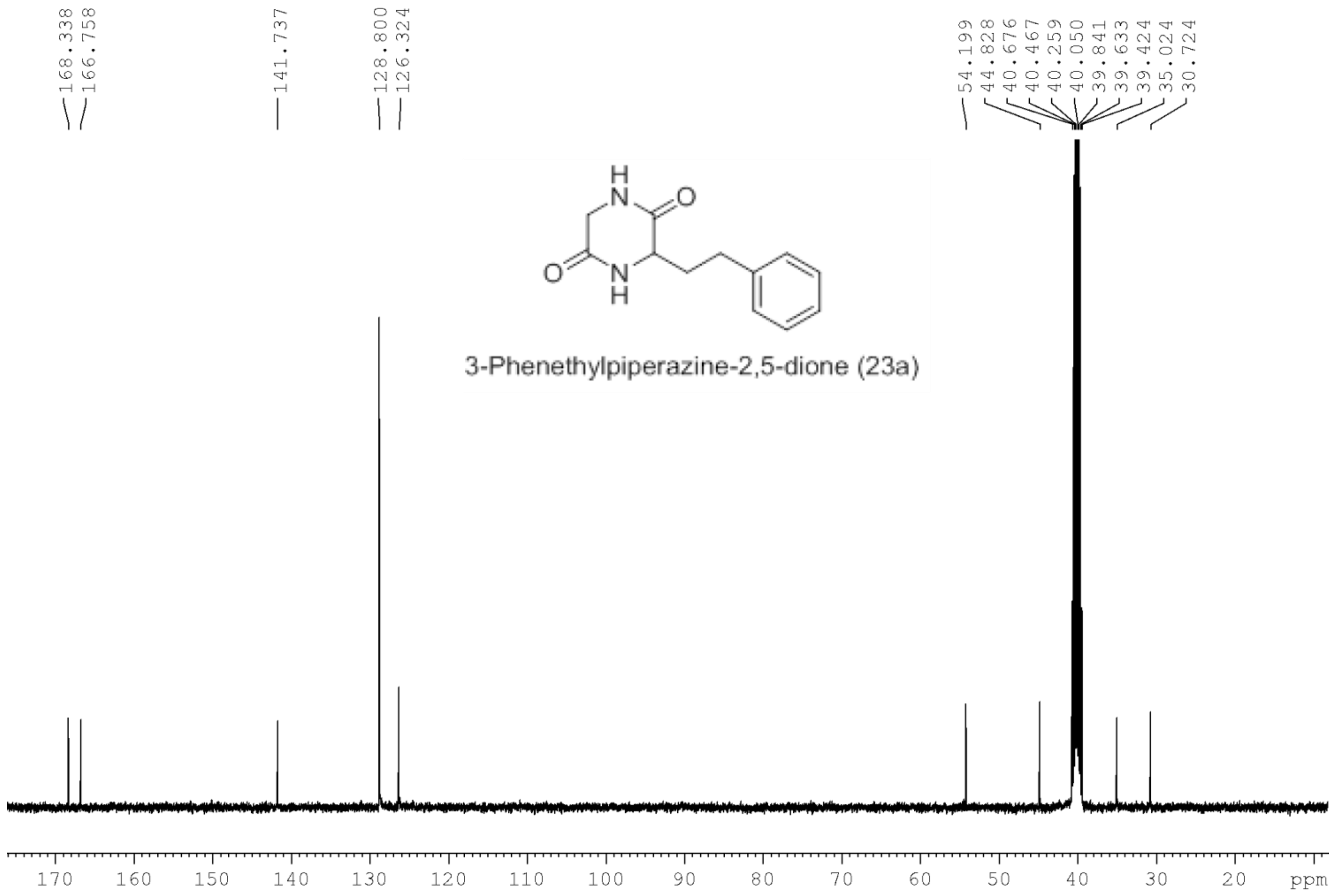

168.338
166.758
141.737
128.800
126.324
54.199
44.828
40.676
40.467
40.259
40.050
39.841
39.633
39.424
35.024
30.724
3-Phenethylpiperazine-2,5-dione (23a)
170 160 150 140 130 120 110 100 90 80 70 60 50 40 30 20 ppm

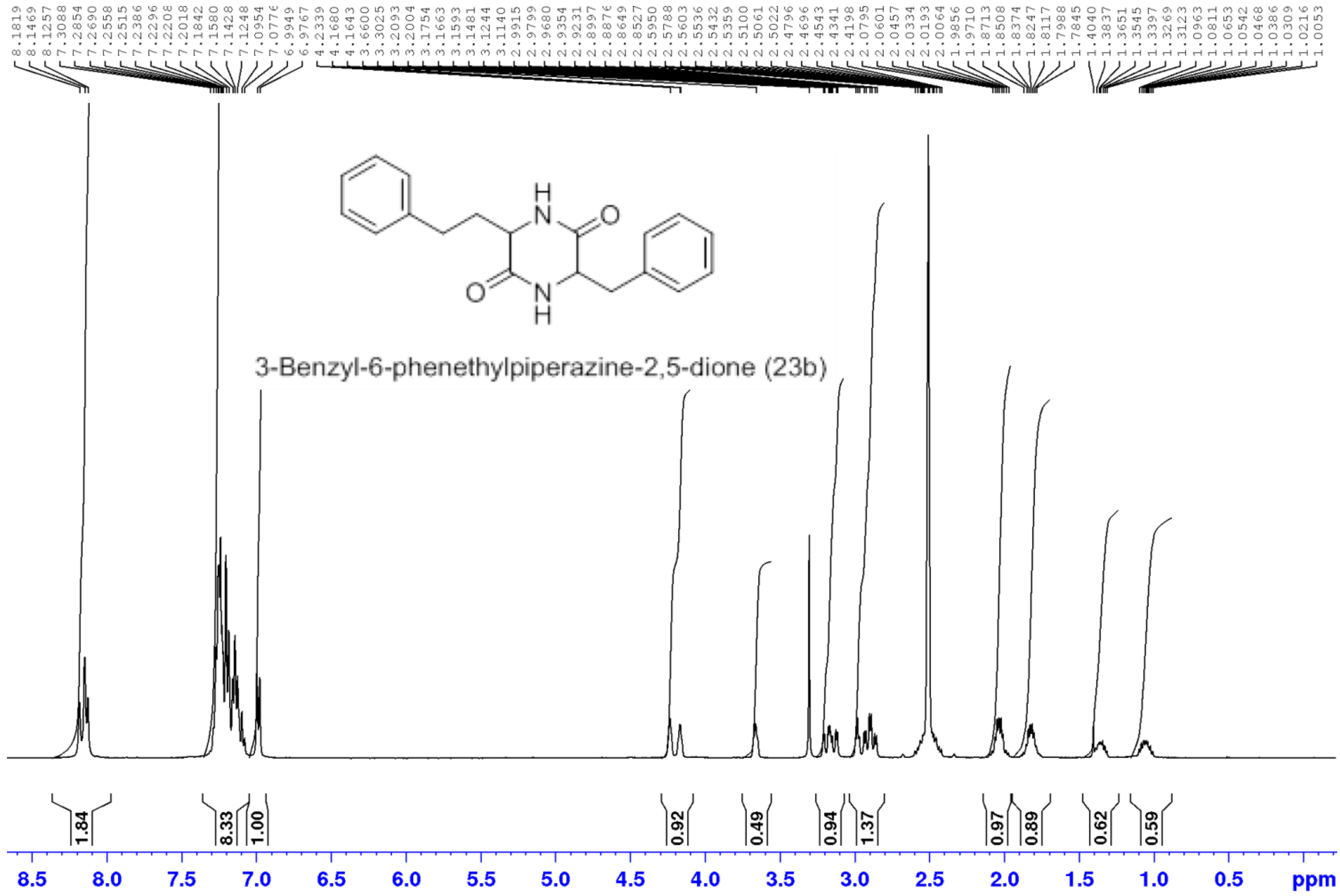

3-Benzyl-6-phenethylpiperazine-2,5-dione (23b)

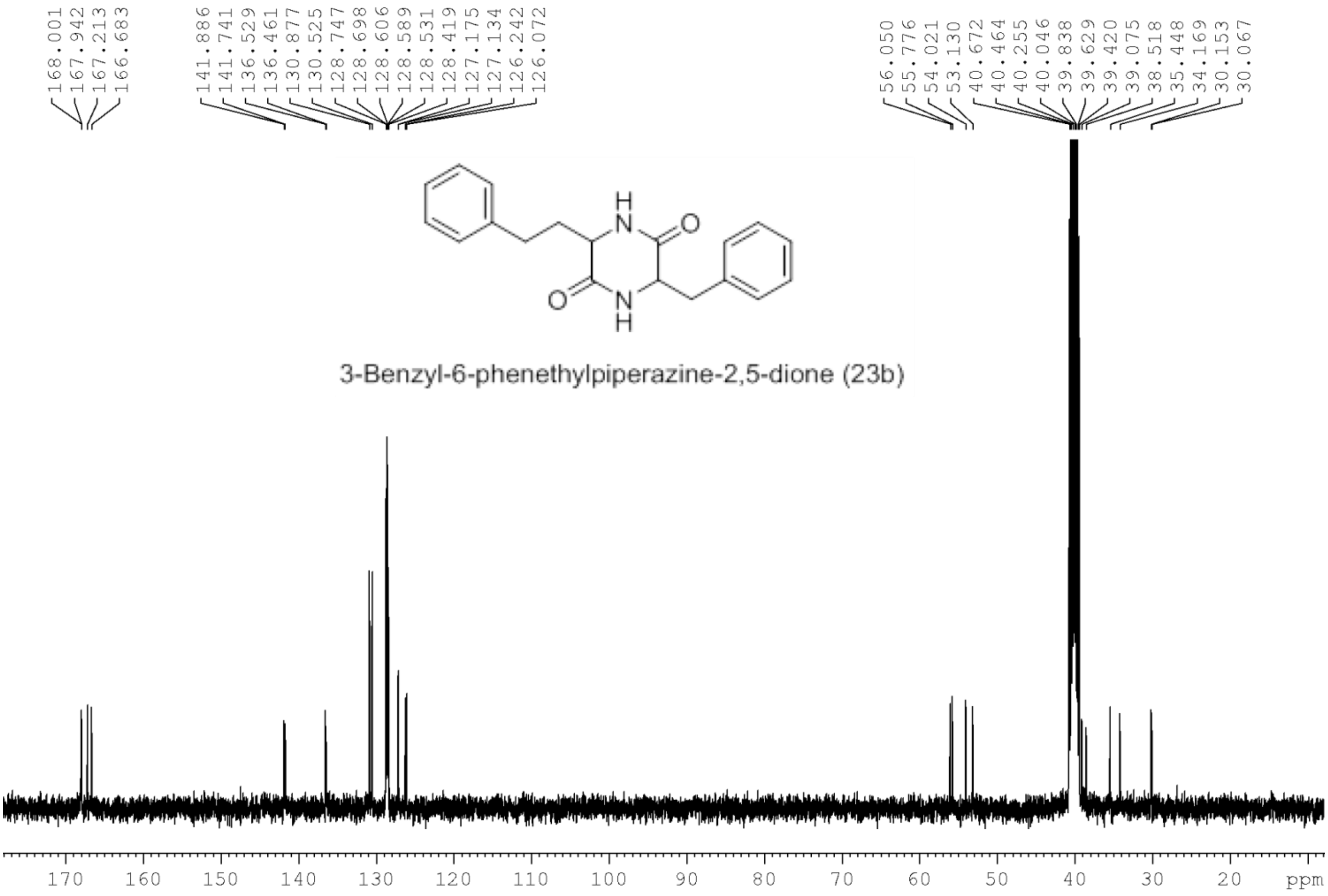

3-Benzyl-6-phenethylpiperazine-2,5-dione (23b)

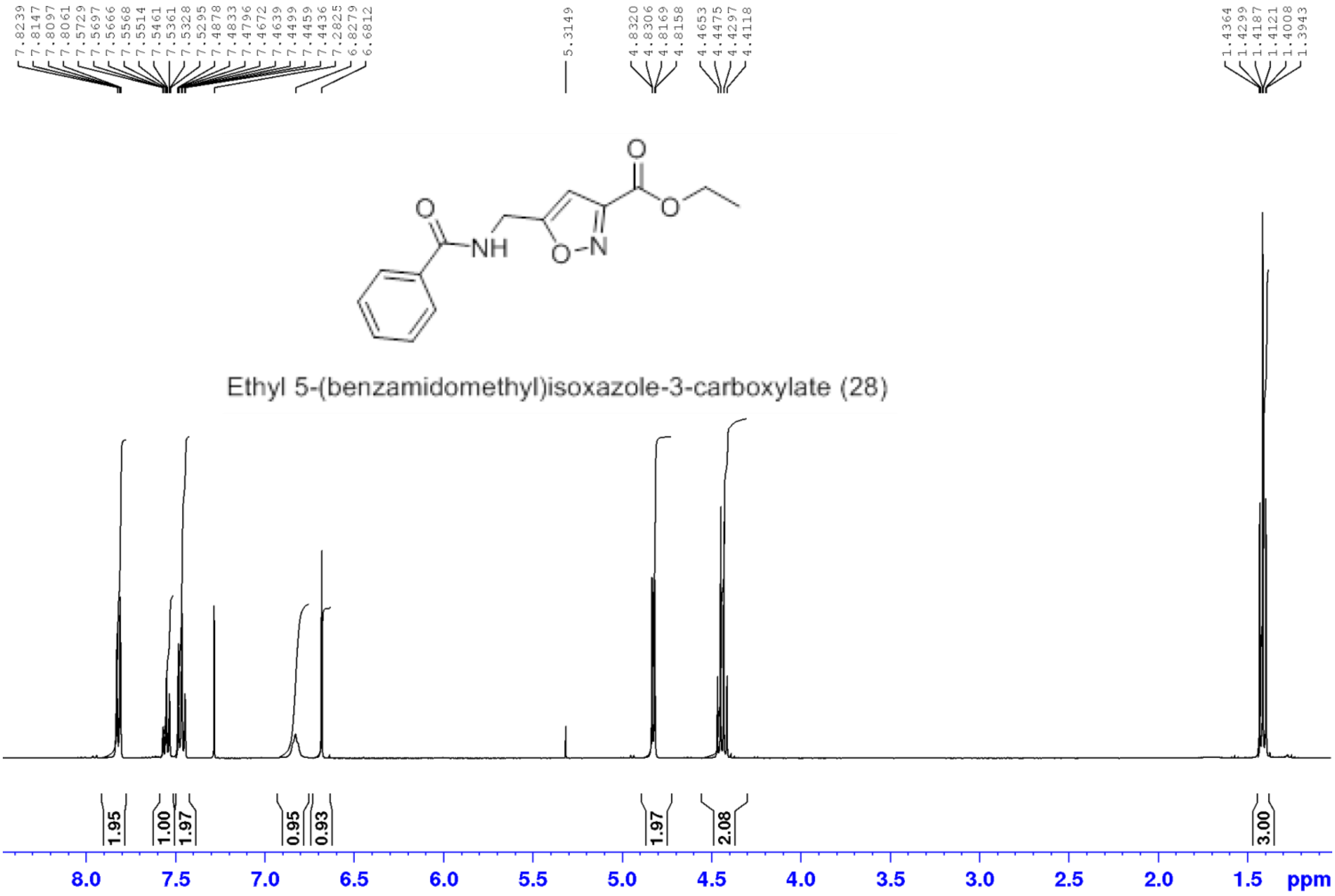
7.8239
7.8147
7.8097
7.8061
7.5729
7.5697
7.5666
7.5568
7.5514
7.5461
7.5361
7.5328
7.5295
7.4878
7.4833
7.4796
7.4672
7.4639
7.4499
7.4459
7.4436
7.2825
6.8279
6.6812
5.3149
4.8320
4.8306
4.8169
4.8158
4.4653
4.4475
4.4297
4.4118
1.4364
1.4299
1.4187
1.4121
1.4008
1.3943
O
NH
O-N
O
O
Ethyl 5-(benzamidomethyl)isoxazole-3-carboxylate (28)
1.95
1.00
1.97
0.95
0.93
1.97
2.08
3.00
8.0
7.5
7.0
6.5
6.0
5.5
5.0
4.5
4.0
3.5
3.0
2.5
2.0
1.5
ppm

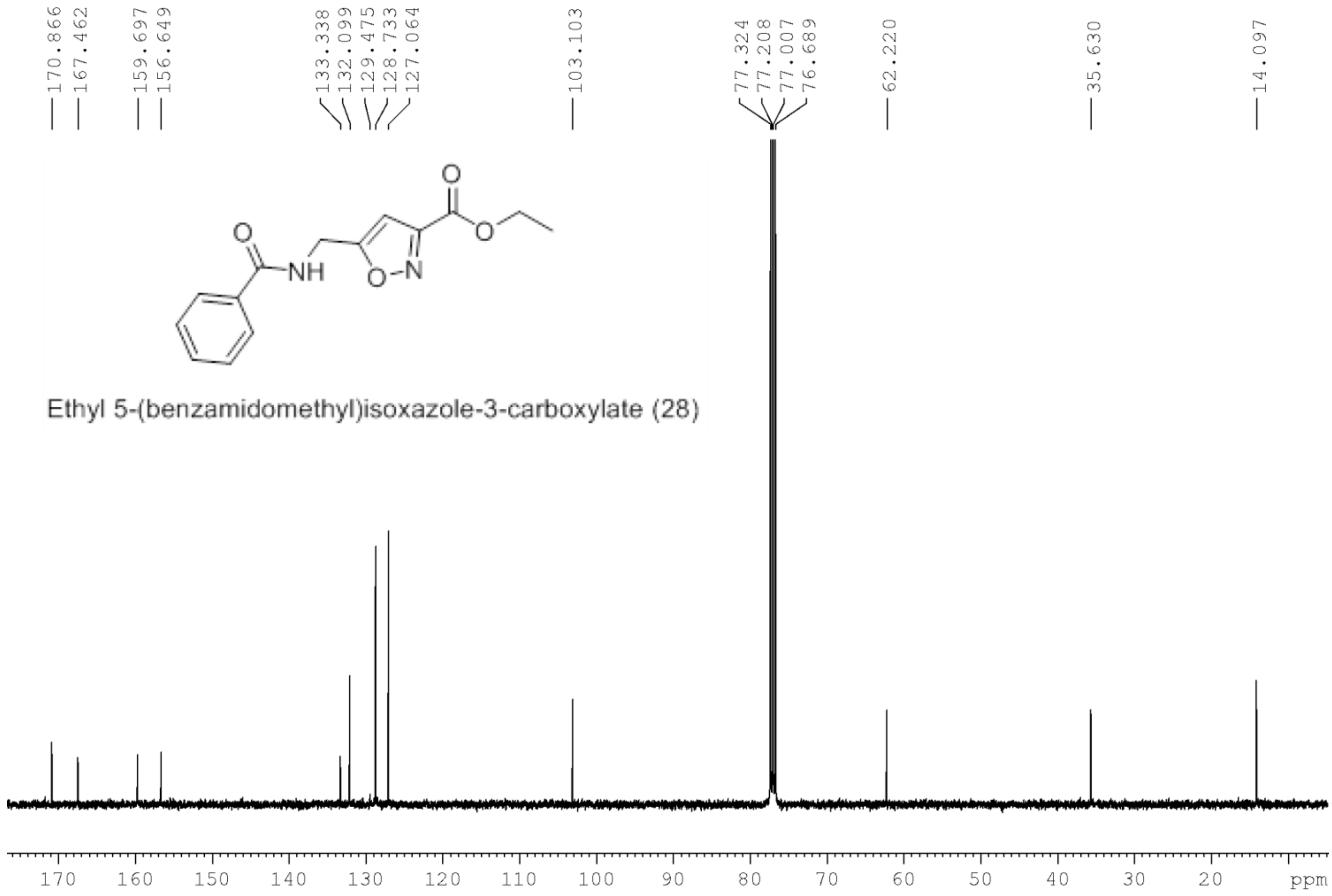
170.866
167.462
159.697
156.649
133.338
132.099
129.475
128.733
127.064
103.103
77.324
77.208
77.007
76.689
62.220
35.630
14.097
O
NH
O
N
O
O
Ethyl 5-(benzamidomethyl)isoxazole-3-carboxylate (28)
170
160
150
140
130
120
110
100
90
80
70
60
50
40
30
20
ppm

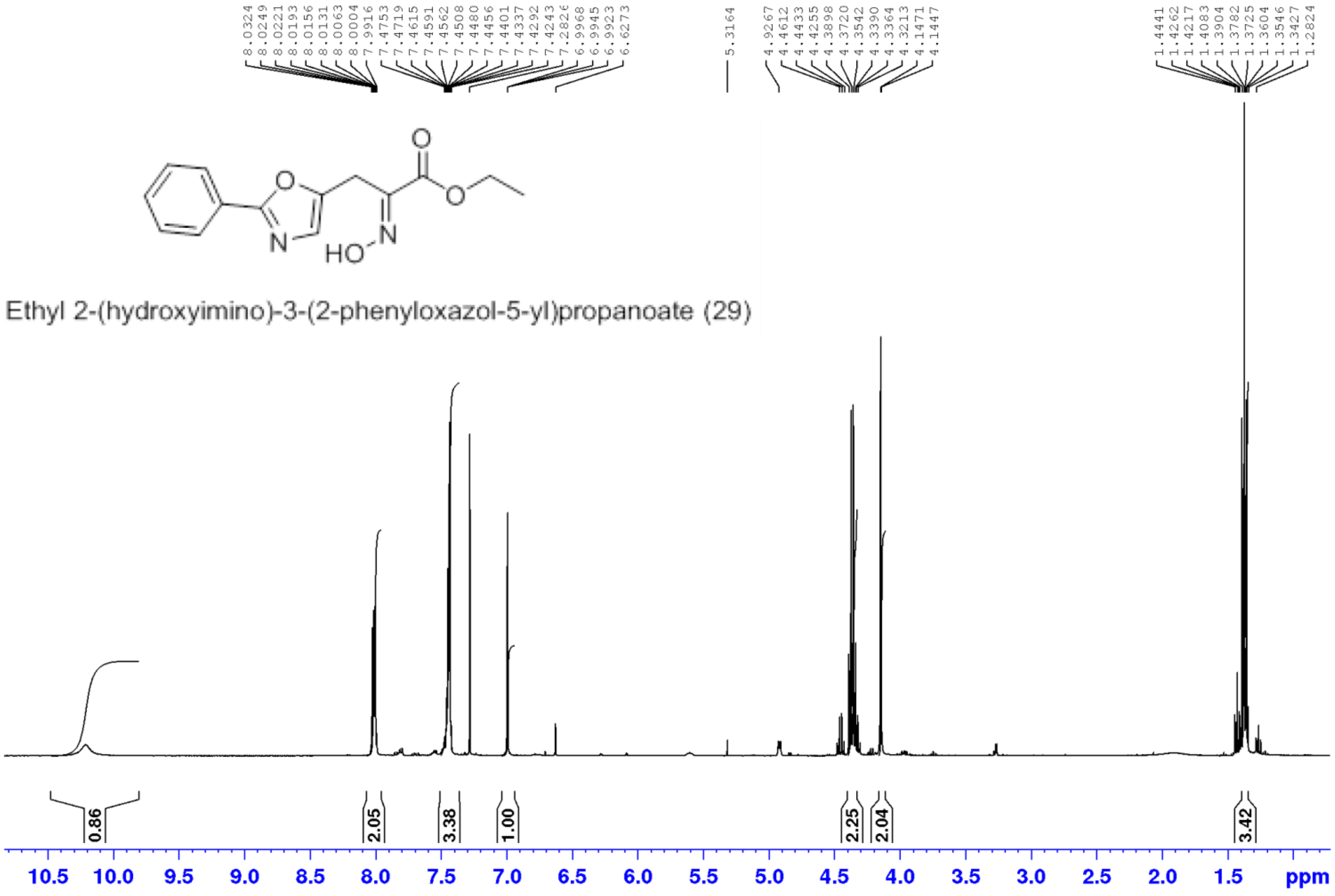
8.0324
8.0249
8.0221
8.0193
8.0156
8.0131
8.0063
8.0004
7.9916
7.4753
7.4719
7.4615
7.4591
7.4562
7.4508
7.4480
7.4456
7.4401
7.4337
7.4292
7.4243
7.2826
6.9968
6.9945
6.9923
6.6273
5.3164
4.9267
4.4612
4.4433
4.4255
4.3898
4.3720
4.3542
4.3390
4.3364
4.3213
4.1471
4.1447
1.4441
1.4262
1.4217
1.4083
1.3904
1.3782
1.3725
1.3604
1.3546
1.3427
1.2824
Ethyl 2-(hydroxyimino)-3-(2-phenyloxazol-5-yl)propanoate (29)
0.86
2.05
3.38
1.00
2.25
2.04
3.42
10.5 10.0 9.5 9.0 8.5 8.0 7.5 7.0 6.5 6.0 5.5 5.0 4.5 4.0 3.5 3.0 2.5 2.0 1.5 ppm

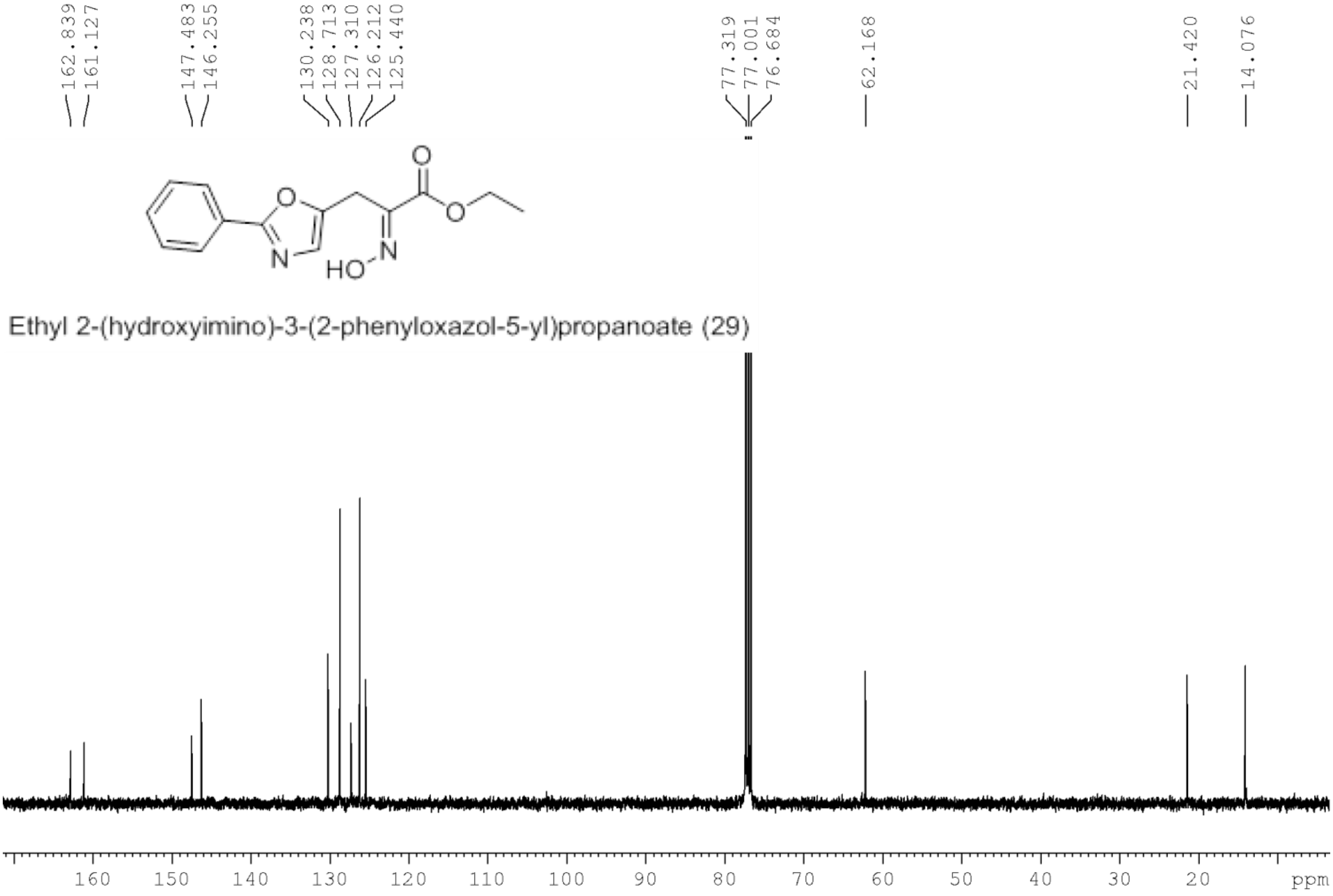

Ethyl 2-(hydroxyimino)-3-(2-phenyloxazol-5-yl)propanoate (29)

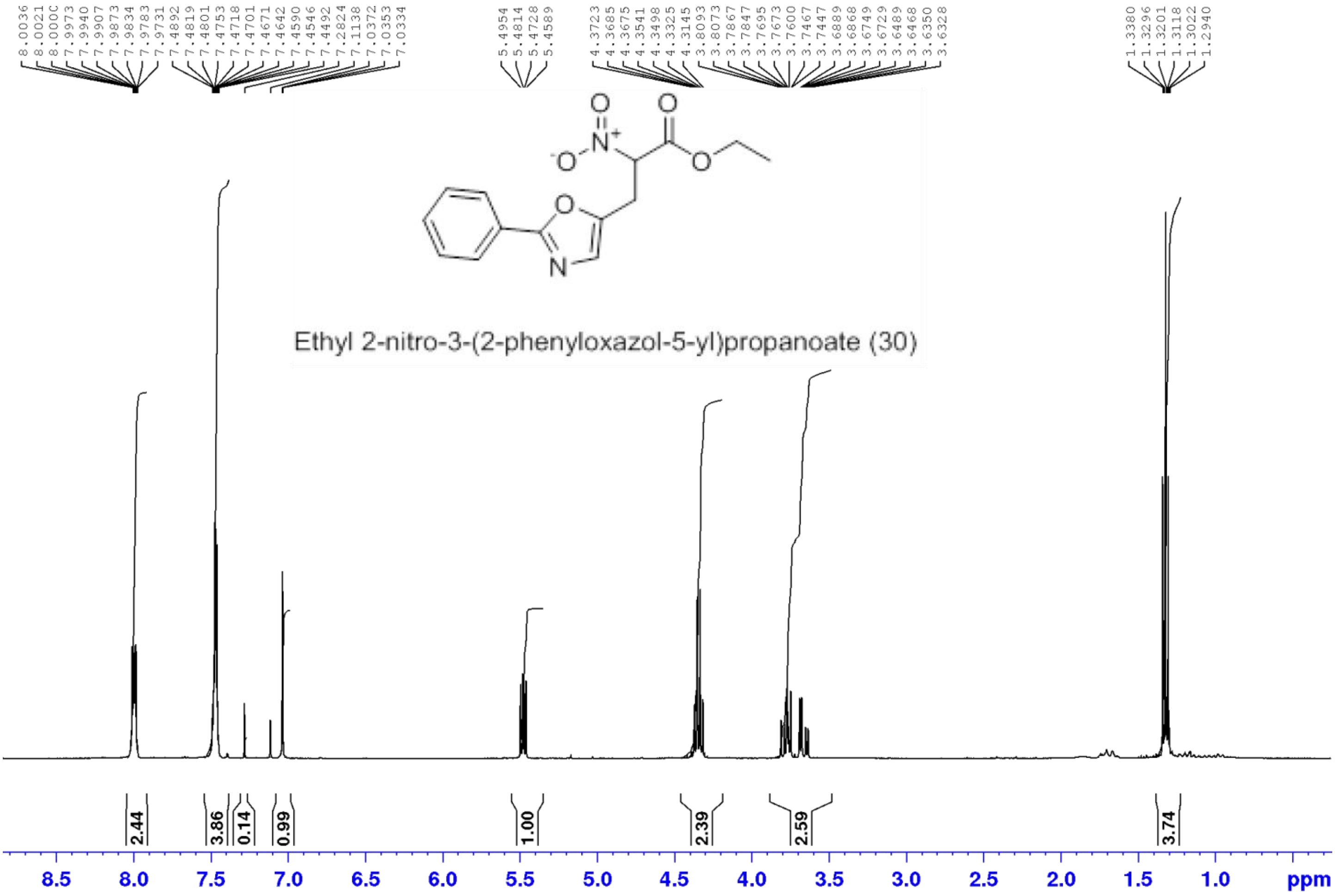
8.0036
8.0021
8.000C
7.9973
7.9940
7.9907
7.9873
7.9834
7.9783
7.9731
7.4892
7.4819
7.4801
7.4753
7.4718
7.4701
7.4671
7.4642
7.4590
7.4546
7.4492
7.2824
7.1138
7.0372
7.0353
7.0334
5.4954
5.4814
5.4728
5.4589
4.3723
4.3685
4.3675
4.3541
4.3498
4.3325
4.3145
3.8093
3.8073
3.7867
3.7847
3.7695
3.7673
3.7600
3.7467
3.7447
3.6889
3.6868
3.6749
3.6729
3.6489
3.6468
3.6350
3.6328
1.3380
1.3296
1.3201
1.3118
1.3022
1.2940
Ethyl 2-nitro-3-(2-phenyloxazol-5-yl)propanoate (30)
2.44
3.86
0.14
0.99
1.00
2.39
2.59
3.74
8.5
8.0
7.5
7.0
6.5
6.0
5.5
5.0
4.5
4.0
3.5
3.0
2.5
2.0
1.5
1.0
ppm

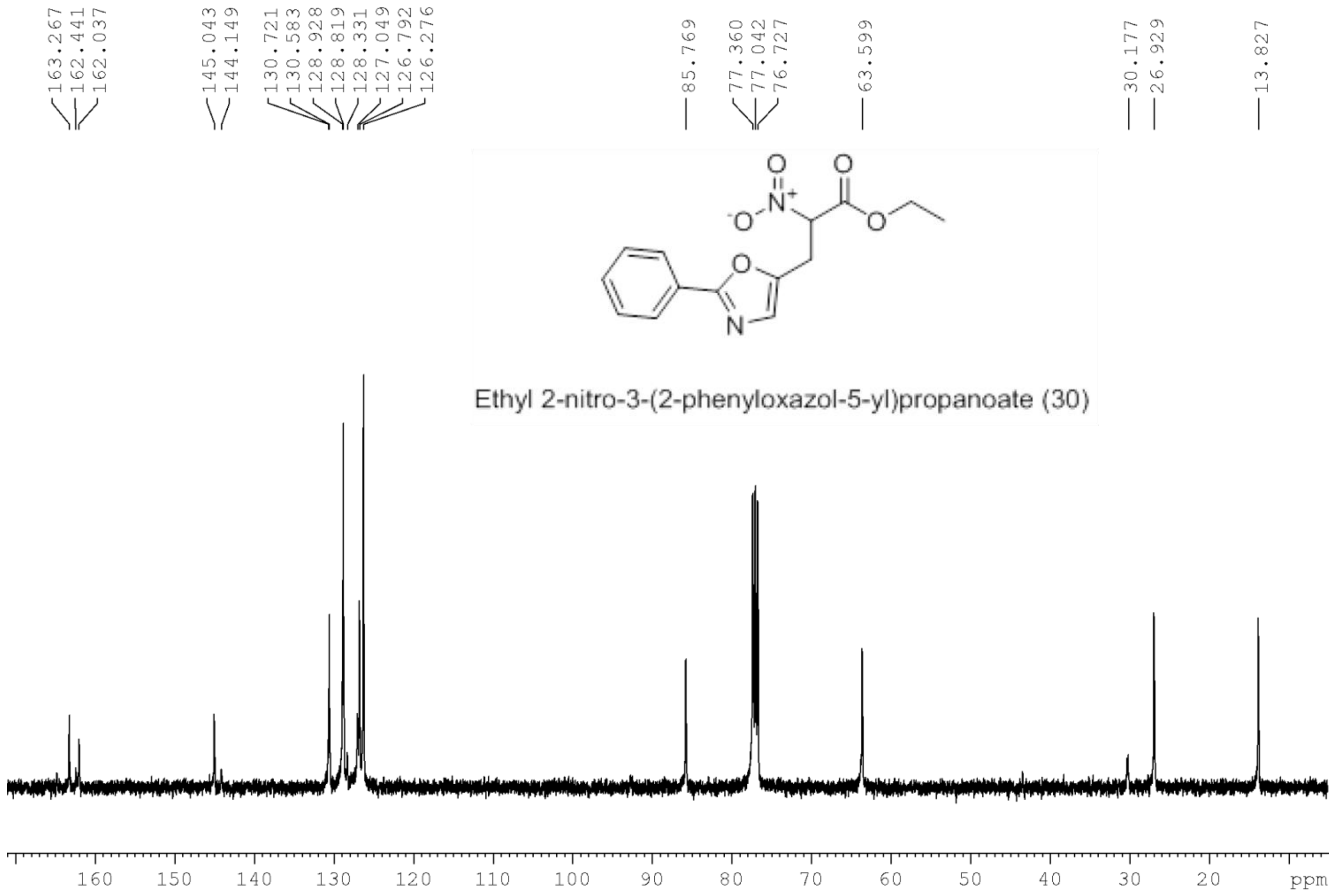

163.267
162.441
162.037
145.043
144.149
130.721
130.583
128.928
128.819
128.331
127.049
126.792
126.276
85.769
77.360
77.042
76.727
63.599
30.177
26.929
13.827
Ethyl 2-nitro-3-(2-phenyloxazol-5-yl)propanoate (30)
160
150
140
130
120
110
100
90
80
70
60
50
40
30
20
ppm

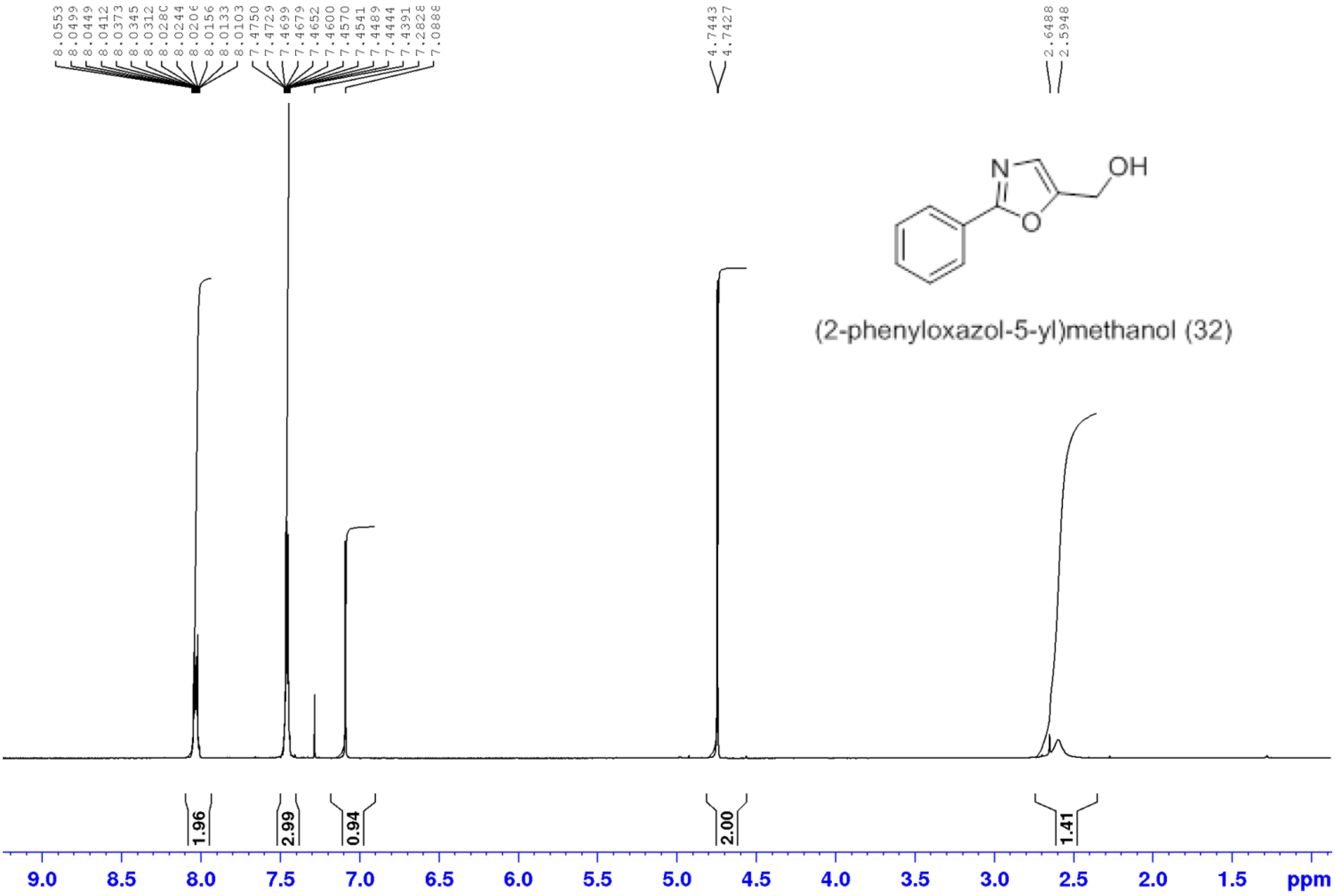

8.0553
8.0499
8.0449
8.0412
8.0373
8.0345
8.0312
8.0280
8.0244
8.0206
8.0156
8.0133
8.0103
7.4750
7.4729
7.4699
7.4679
7.4652
7.4600
7.4570
7.4541
7.4489
7.4444
7.4391
7.2828
7.0888
4.7443
4.7427
2.6488
2.5948
N
OH
O
(2-phenyloxazol-5-yl)methanol (32)
1.96
2.99
0.94
2.00
1.41
9.0
8.5
8.0
7.5
7.0
6.5
6.0
5.5
5.0
4.5
4.0
3.5
3.0
2.5
2.0
1.5
ppm

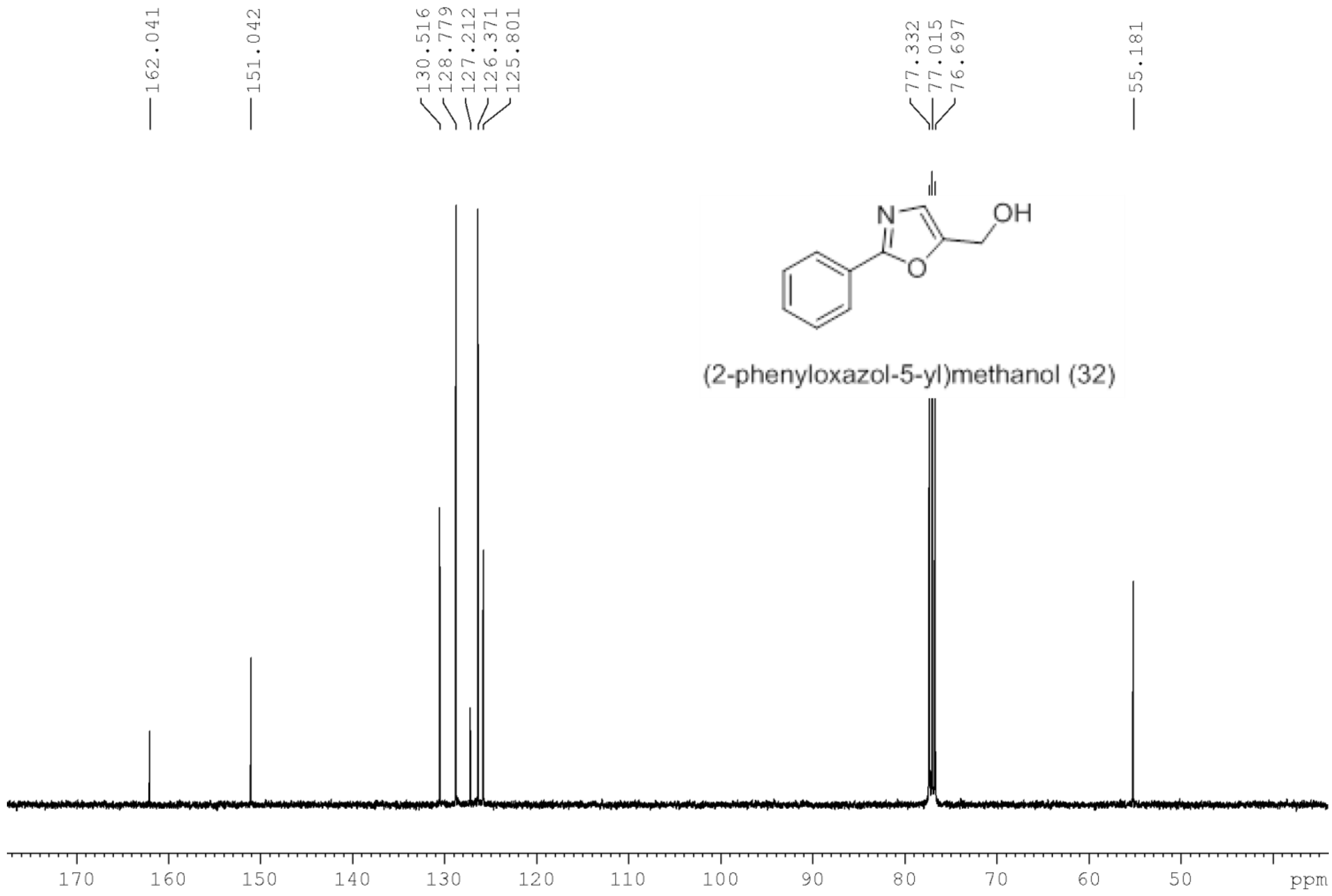

(2-phenyloxazol-5-yl)methanol (32)

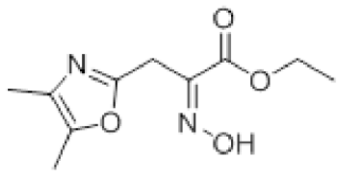

Ethyl 3-(4,5-dimethyloxazol-2-yl)-2-(hydroxyimino)propanoate (35)

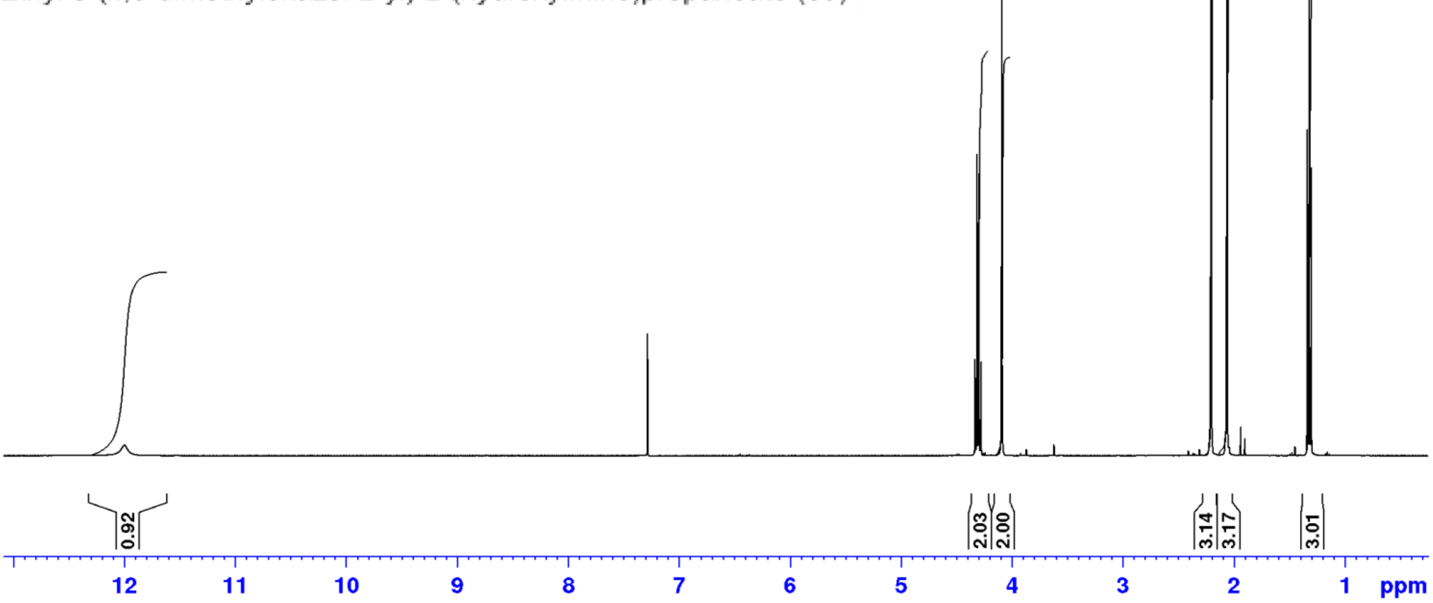

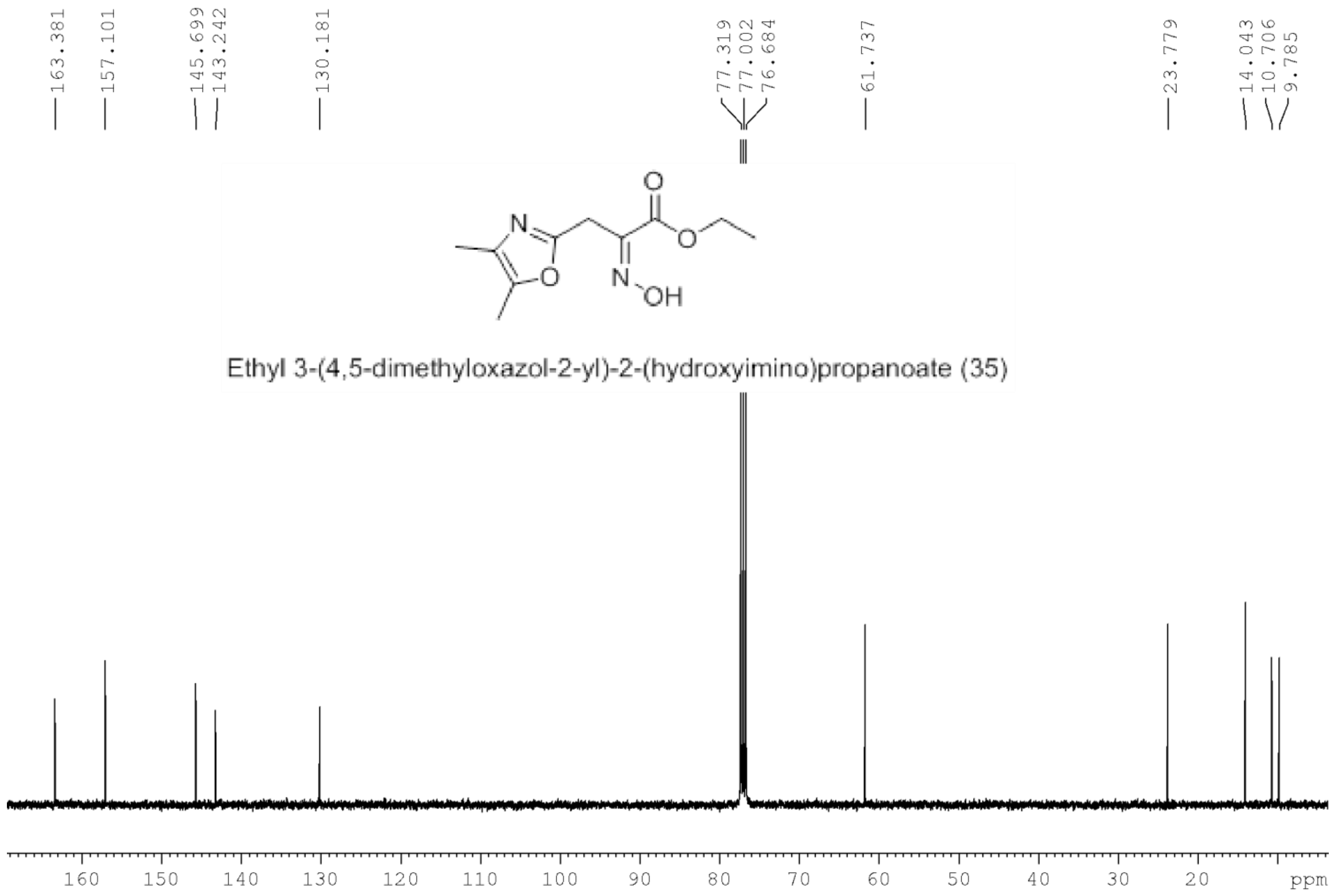

Ethyl 3-(4,5-dimethyloxazol-2-yl)-2-(hydroxyimino)propanoate (35)

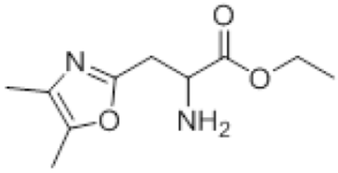

Ethyl 2-amino-3-(4,5-dimethyloxazol-2-yl)propanoate (36)

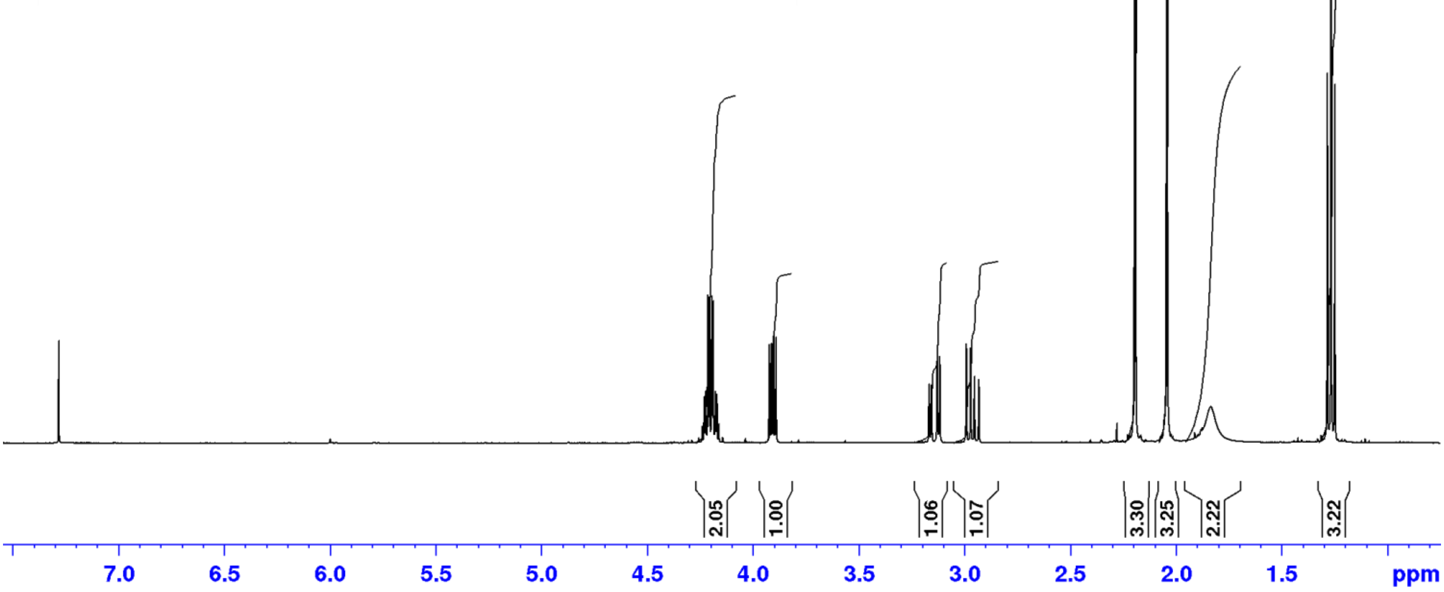

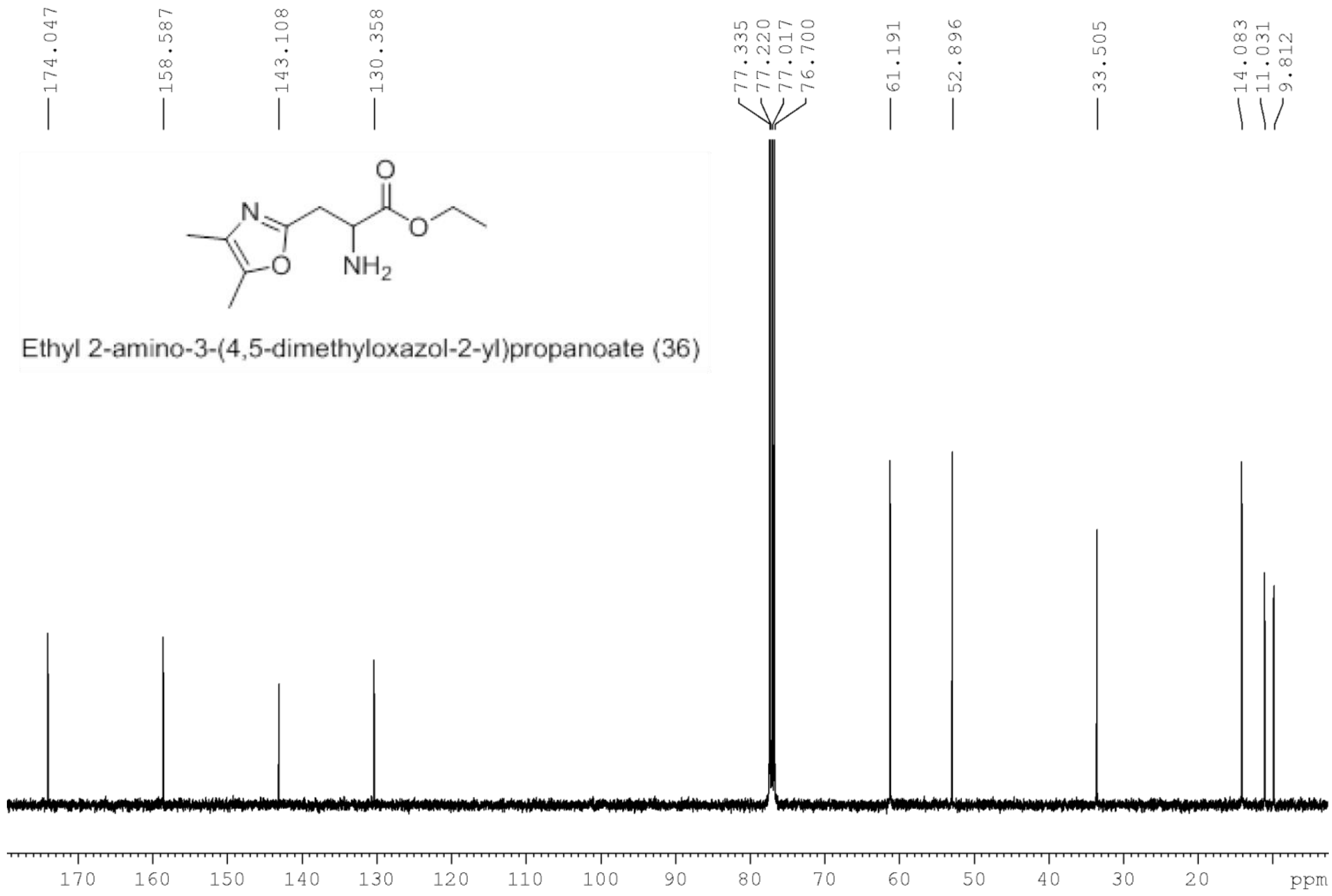
174.047
158.587
143.108
130.358
77.335
77.220
77.017
76.700
61.191
52.896
33.505
14.083
11.031
9.812
N
O
O
O
NH2
Ethyl 2-amino-3-(4,5-dimethyloxazol-2-yl)propanoate (36)
170
160
150
140
130
120
110
100
90
80
70
60
50
40
30
20
ppm

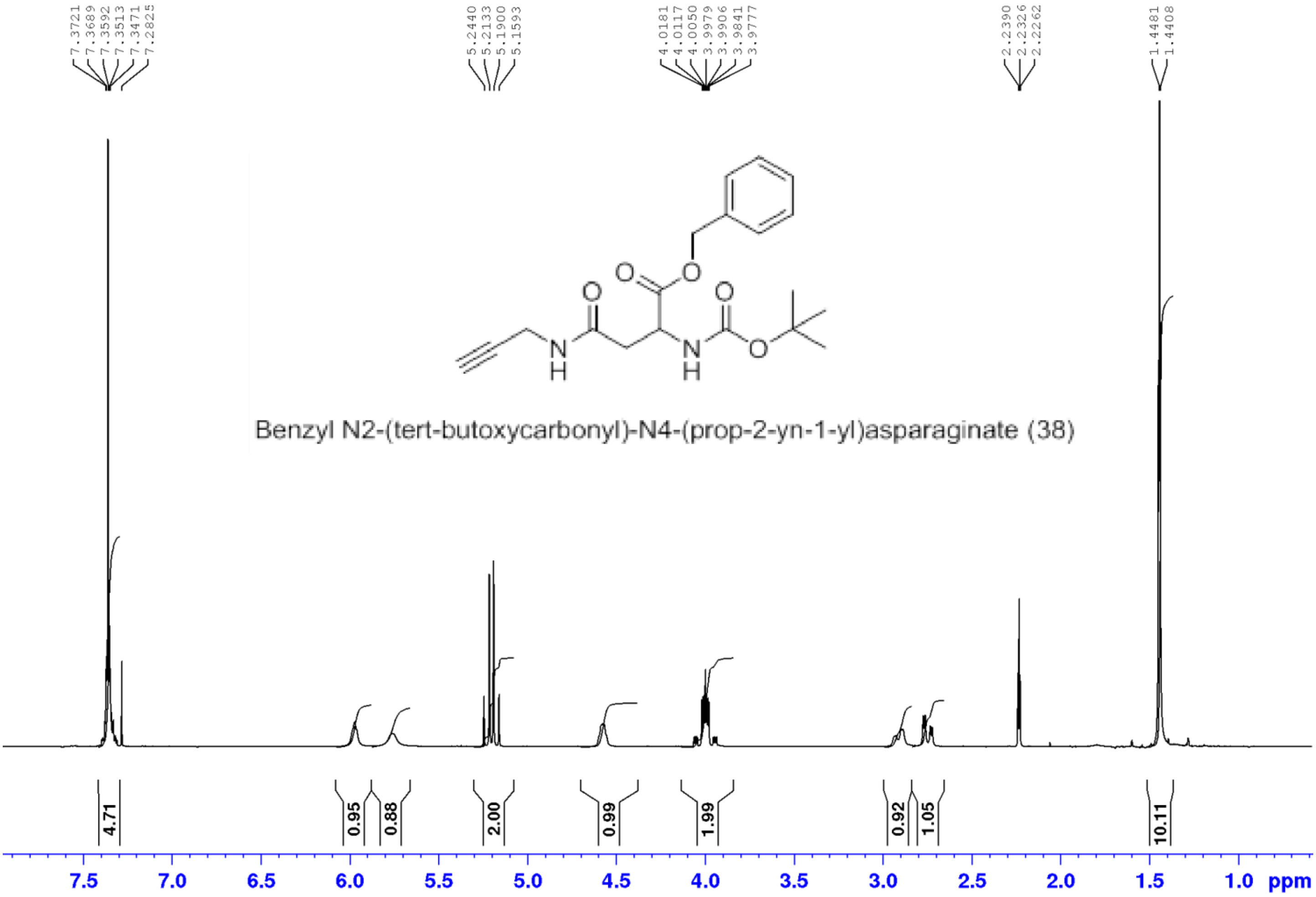

Benzyl N2-(tert-butoxycarbonyl)-N4-(prop-2-yn-1-yl)asparaginate (38)

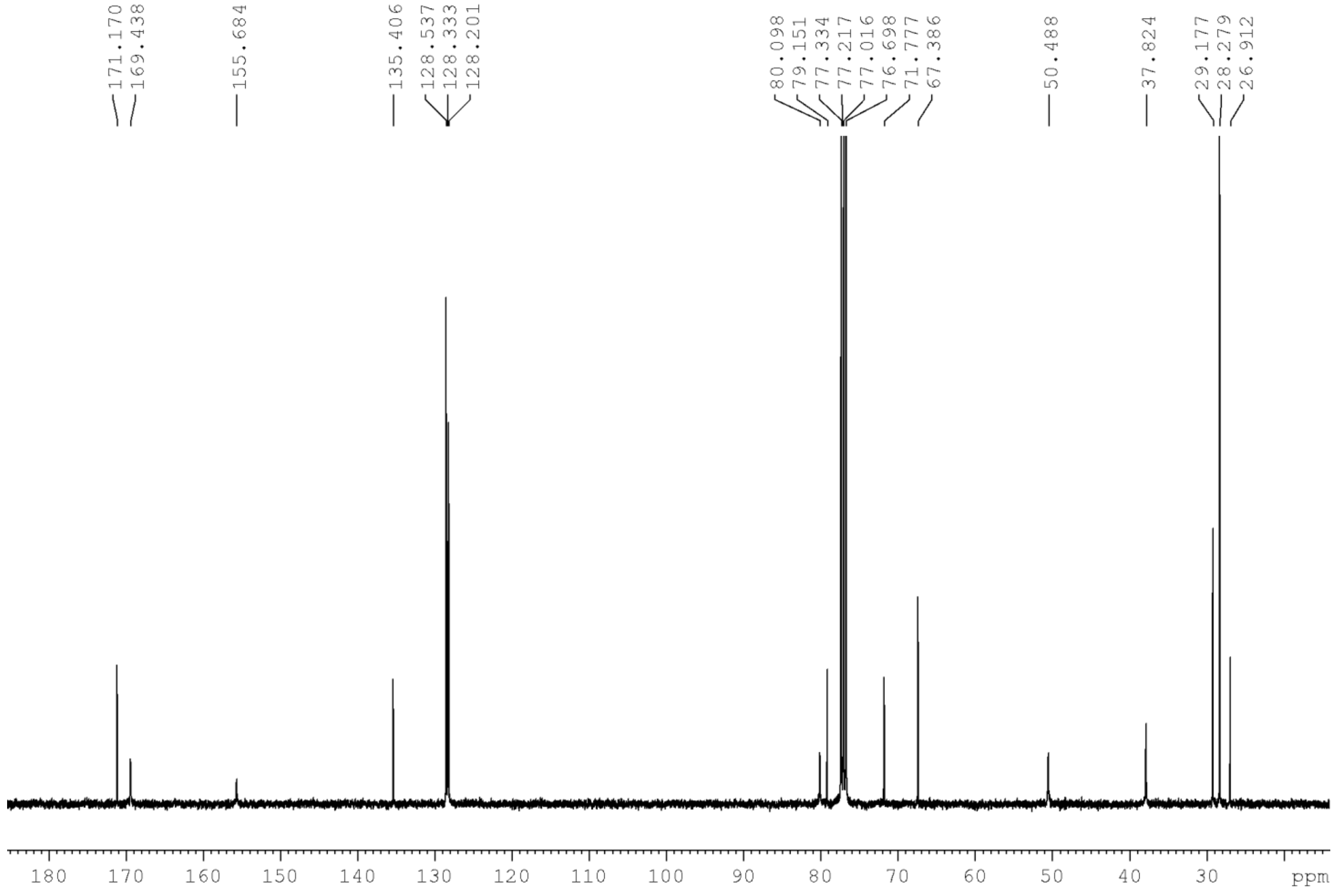
171.170
169.438
155.684
135.406
128.537
128.333
128.201
80.098
79.151
77.334
77.217
77.016
76.698
71.777
67.386
50.488
37.824
29.177
28.279
26.912
180 170 160 150 140 130 120 110 100 90 80 70 60 50 40 30 ppm

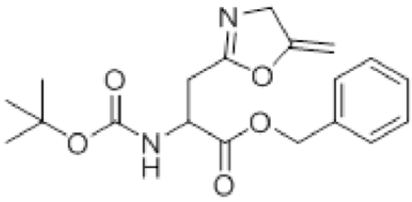

Benzyl 2-((tert-butoxycarbonyl)amino)-3-(5-methylene-4,5-dihydrooxazol-2-yl)propanoate (40)

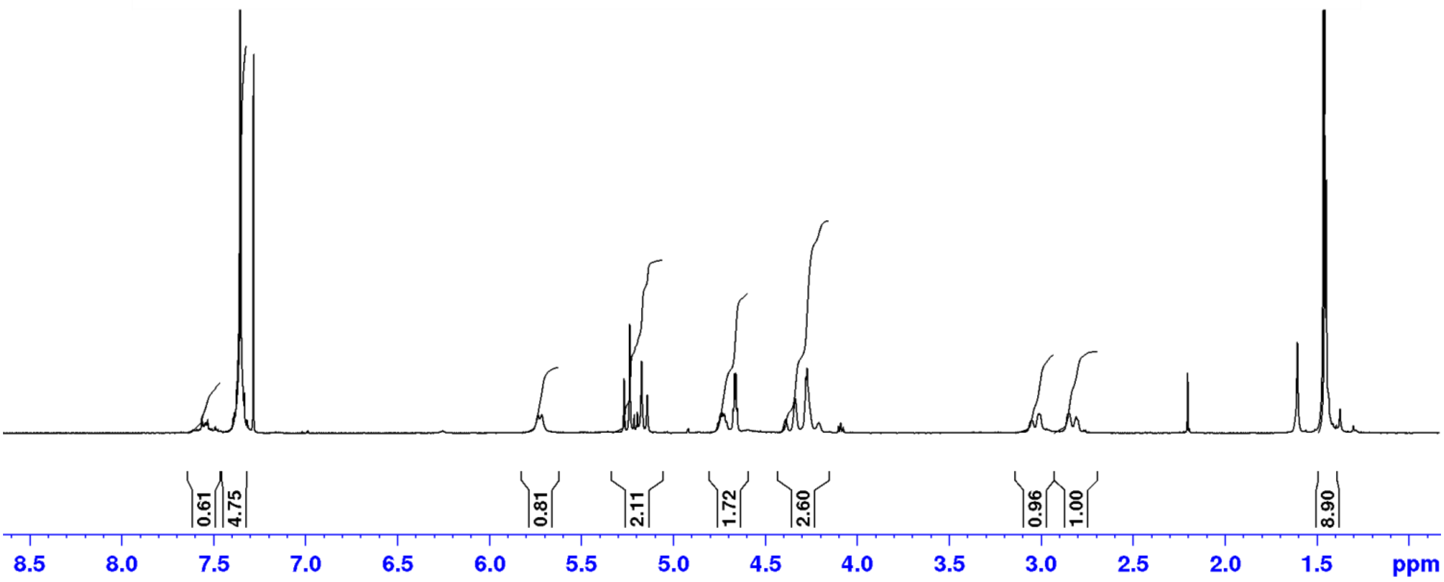

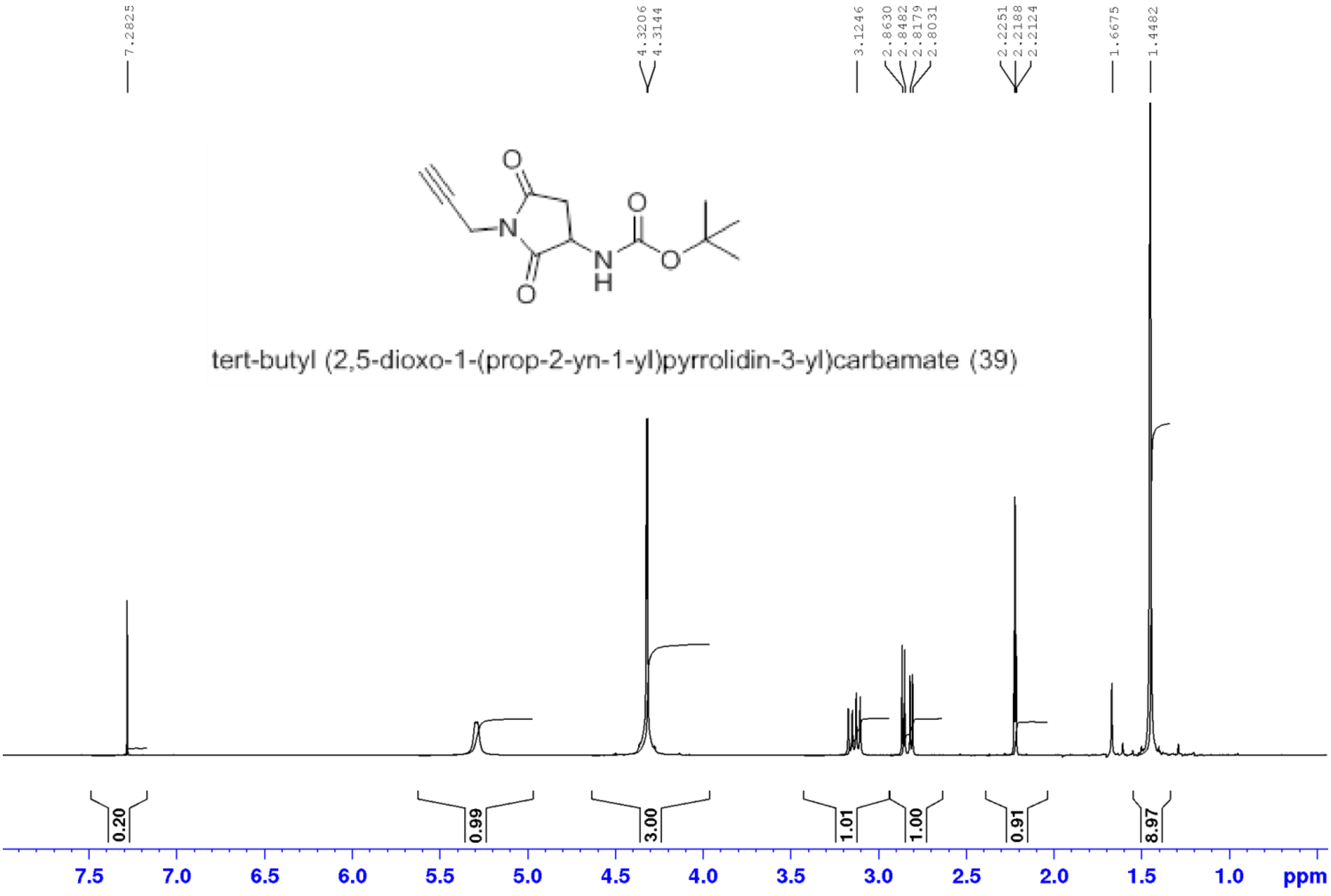
7.2825
4.3206
4.3144
3.1246
2.8630
2.8482
2.8179
2.8031
2.2251
2.2188
2.2124
1.6675
1.4482
tert-butyl (2,5-dioxo-1-(prop-2-yn-1-yl)pyrrolidin-3-yl)carbamate (39)
0.20
0.99
3.00
1.01
1.00
0.91
8.97
7.5
7.0
6.5
6.0
5.5
5.0
4.5
4.0
3.5
3.0
2.5
2.0
1.5
1.0
ppm

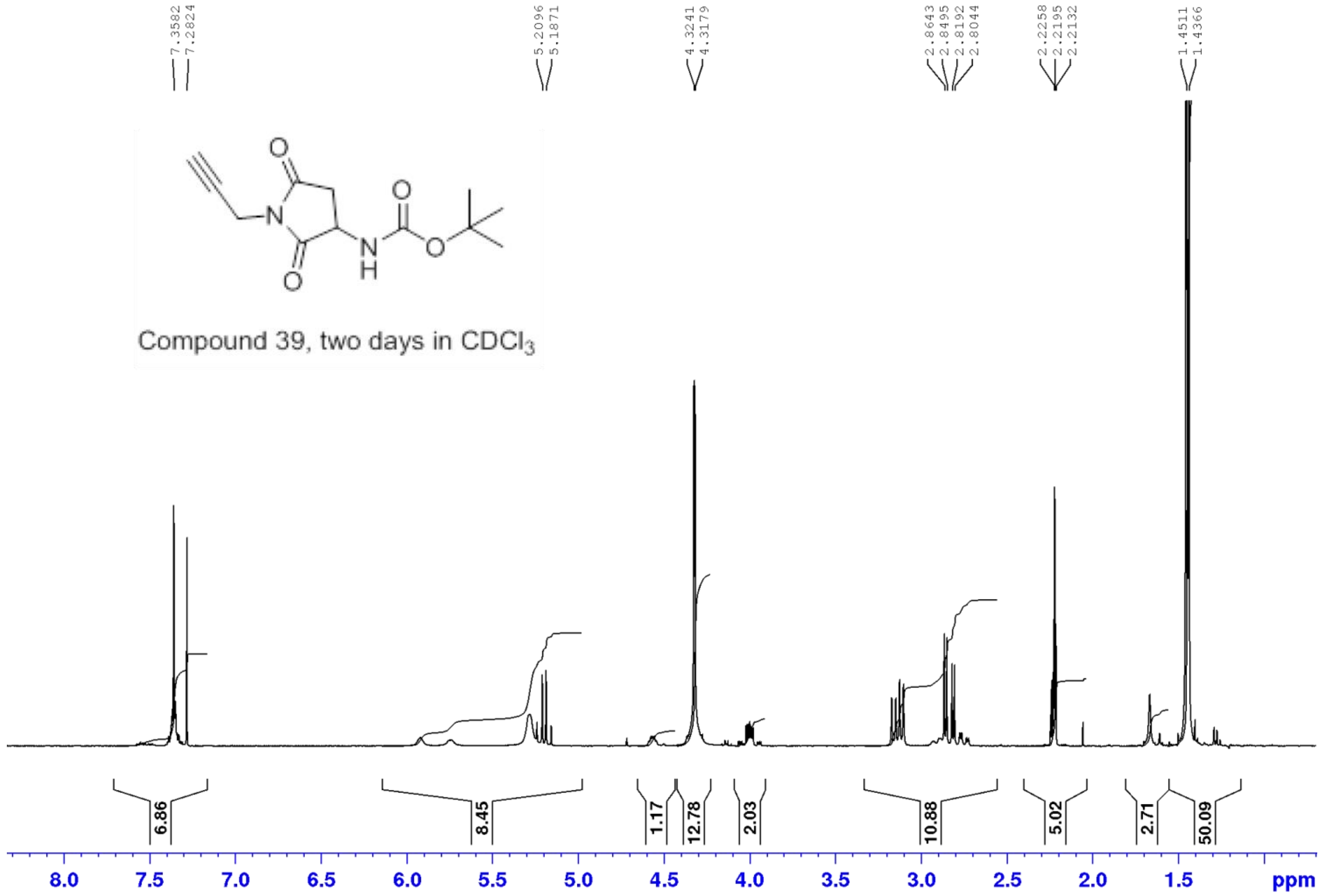
7.3582
7.2824
5.2096
5.1871
4.3241
4.3179
2.8643
2.8495
2.8192
2.8044
2.2258
2.2195
2.2132
1.4511
1.4366
Compound 39, two days in $CDCl_3$
6.86
8.45
1.17
12.78
2.03
10.88
5.02
2.71
50.09
8.0
7.5
7.0
6.5
6.0
5.5
5.0
4.5
4.0
3.5
3.0
2.5
2.0
1.5
ppm

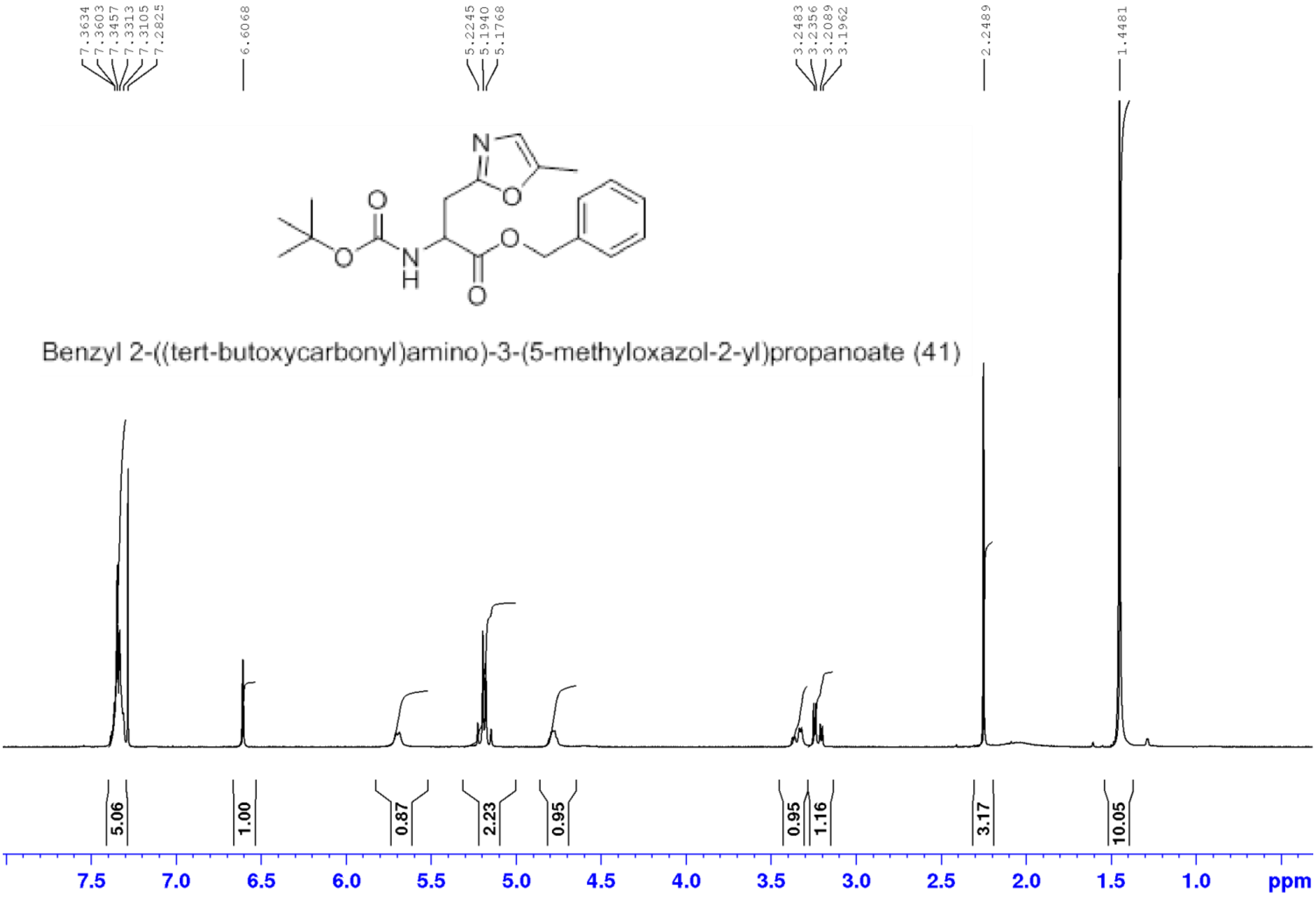

Benzyl 2-((tert-butoxycarbonyl)amino)-3-(5-methyloxazol-2-yl)propanoate (41)

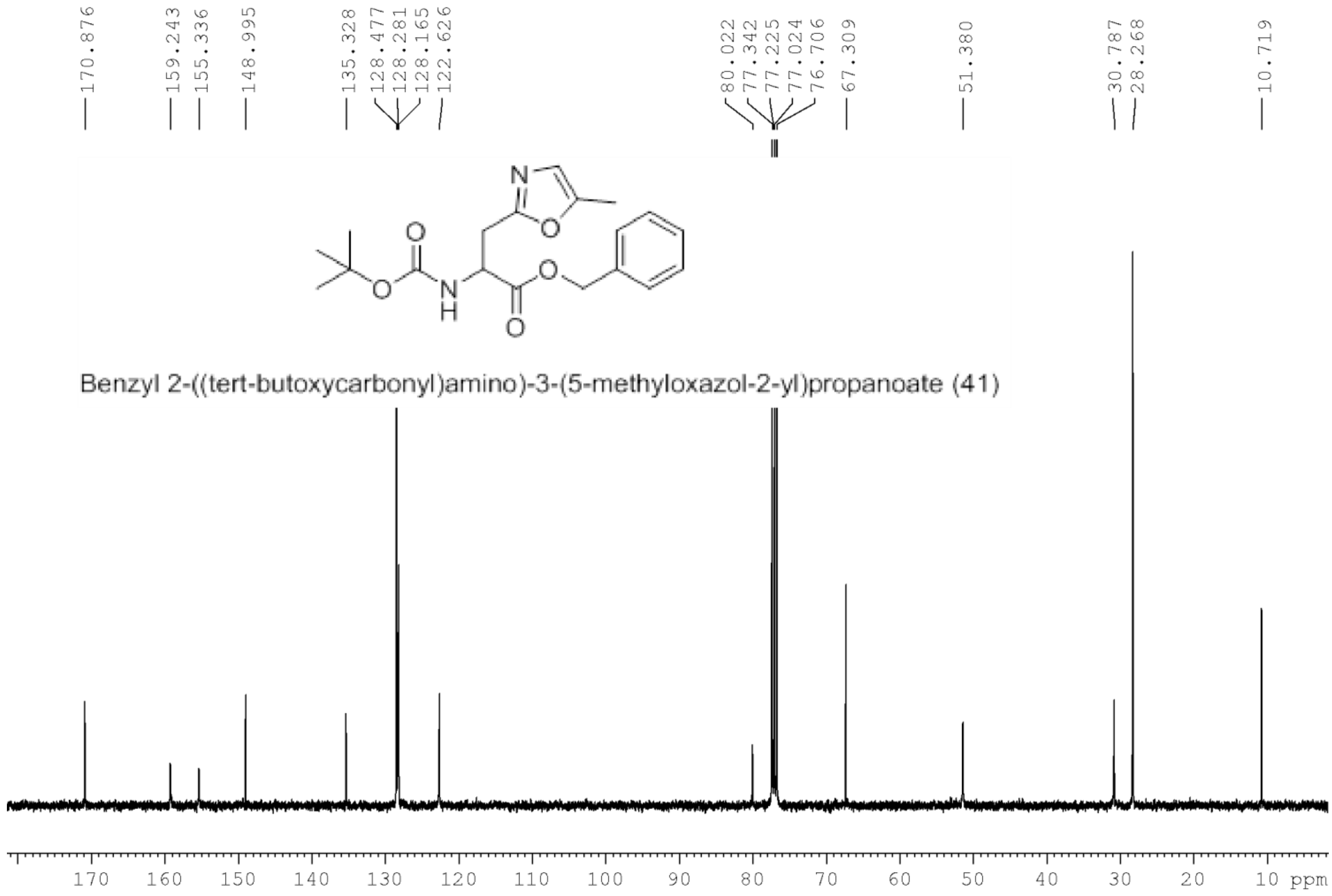

Benzyl 2-((tert-butoxycarbonyl)amino)-3-(5-methyloxazol-2-yl)propanoate (41)

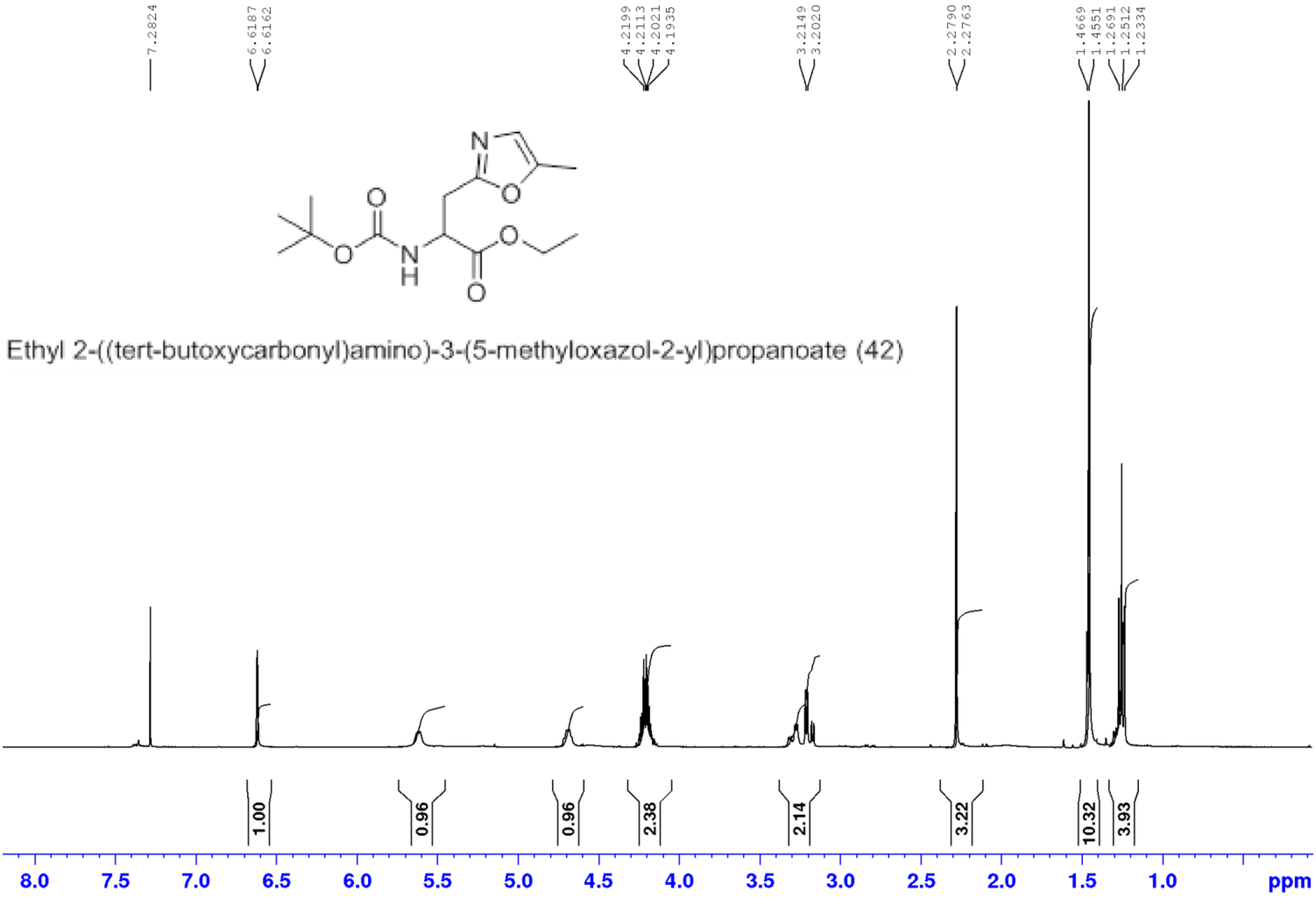

Ethyl 2-((tert-butoxycarbonyl)amino)-3-(5-methyloxazol-2-yl)propanoate (42)

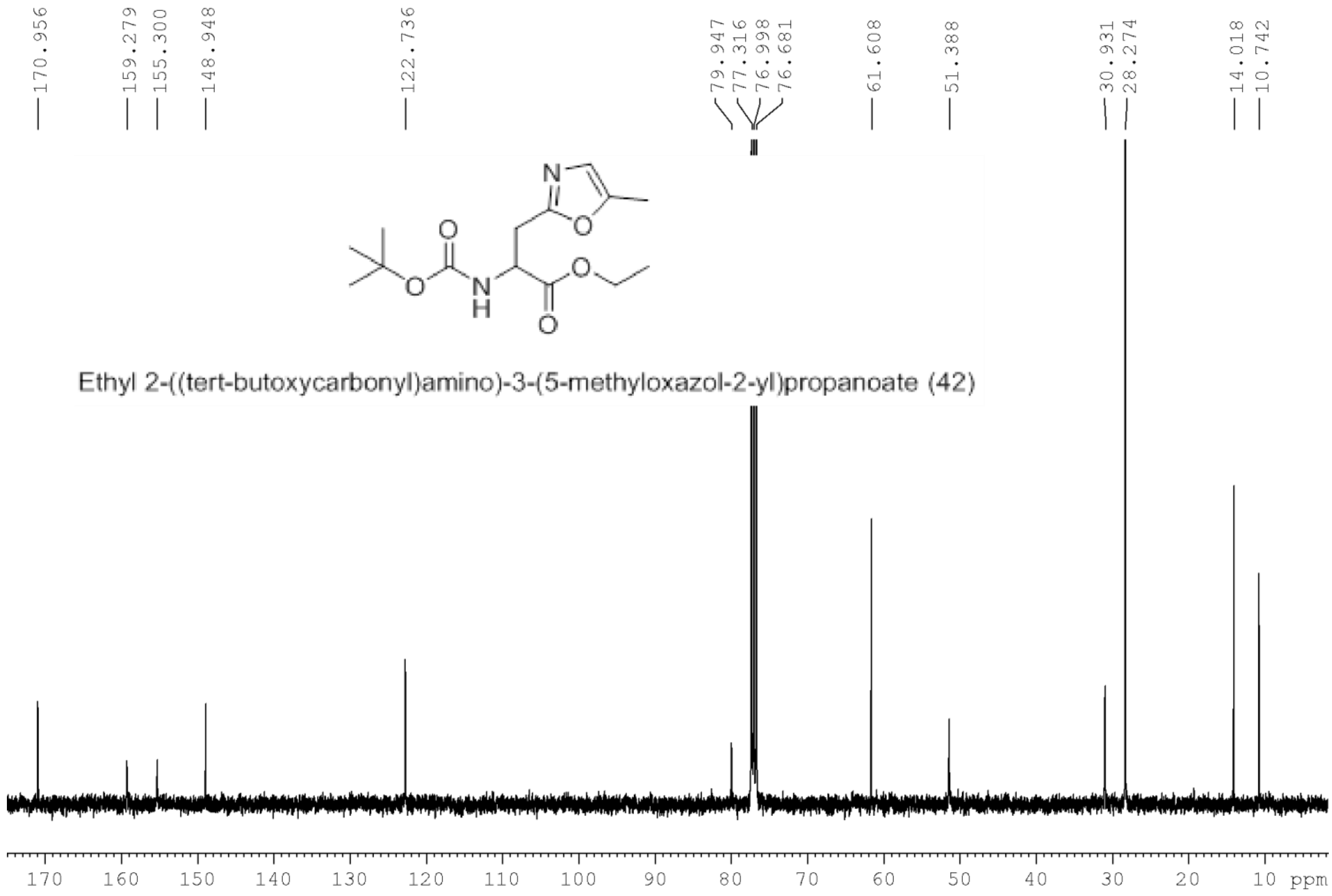

Ethyl 2-((tert-butoxycarbonyl)amino)-3-(5-methyloxazol-2-yl)propanoate (42)

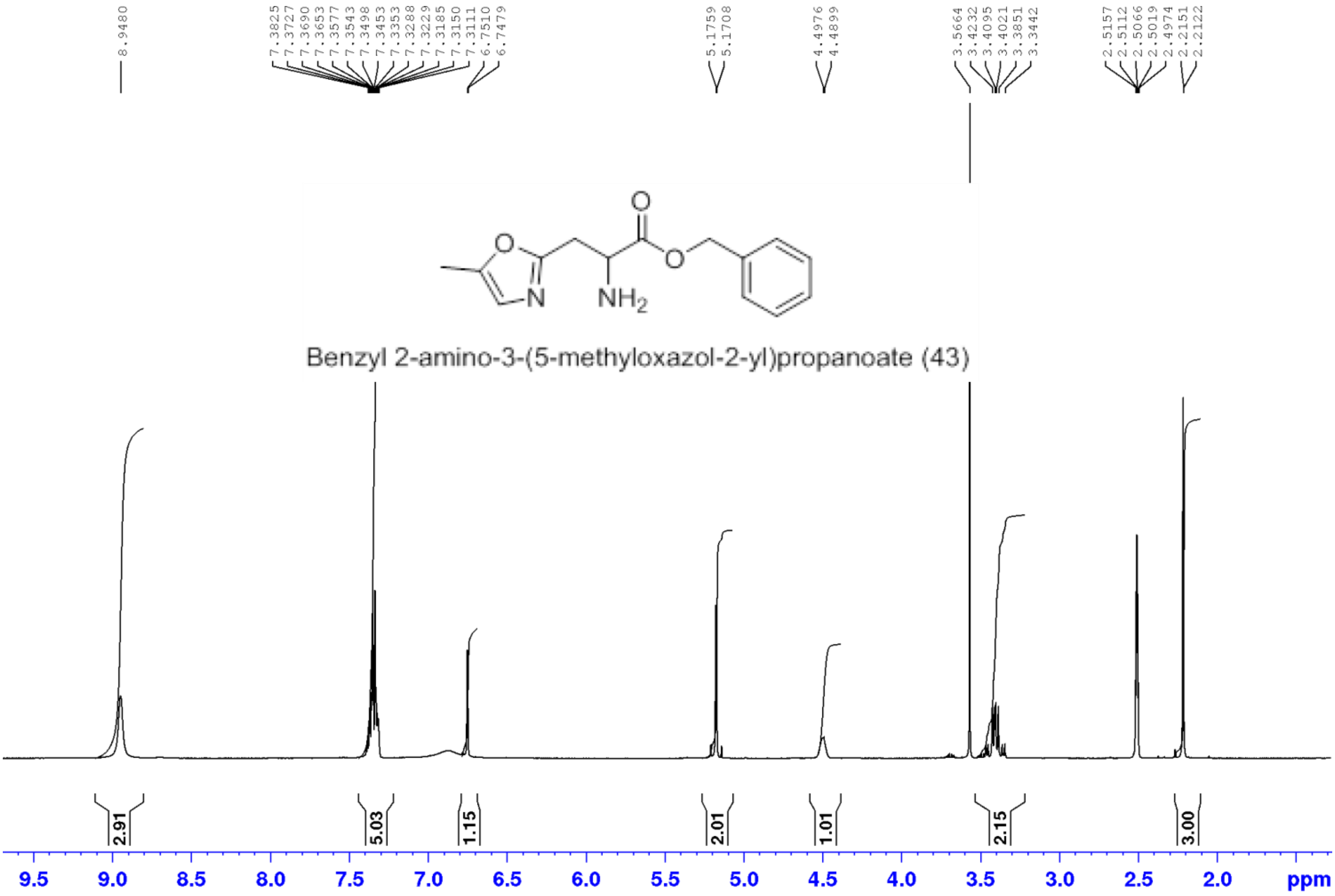
8.9480
7.3825
7.3727
7.3690
7.3653
7.3577
7.3543
7.3498
7.3453
7.3353
7.3288
7.3229
7.3185
7.3150
7.3111
6.7510
6.7479
5.1759
5.1708
4.4976
4.4899
3.5664
3.4232
3.4095
3.4021
3.3851
3.3442
2.5157
2.5112
2.5066
2.5019
2.4974
2.2151
2.2122
O
O
N
O
NH2
Benzyl 2-amino-3-(5-methyloxazol-2-yl)propanoate (43)
2.91
5.03
1.15
2.01
1.01
2.15
3.00
9.5
9.0
8.5
8.0
7.5
7.0
6.5
6.0
5.5
5.0
4.5
4.0
3.5
3.0
2.5
2.0
ppm

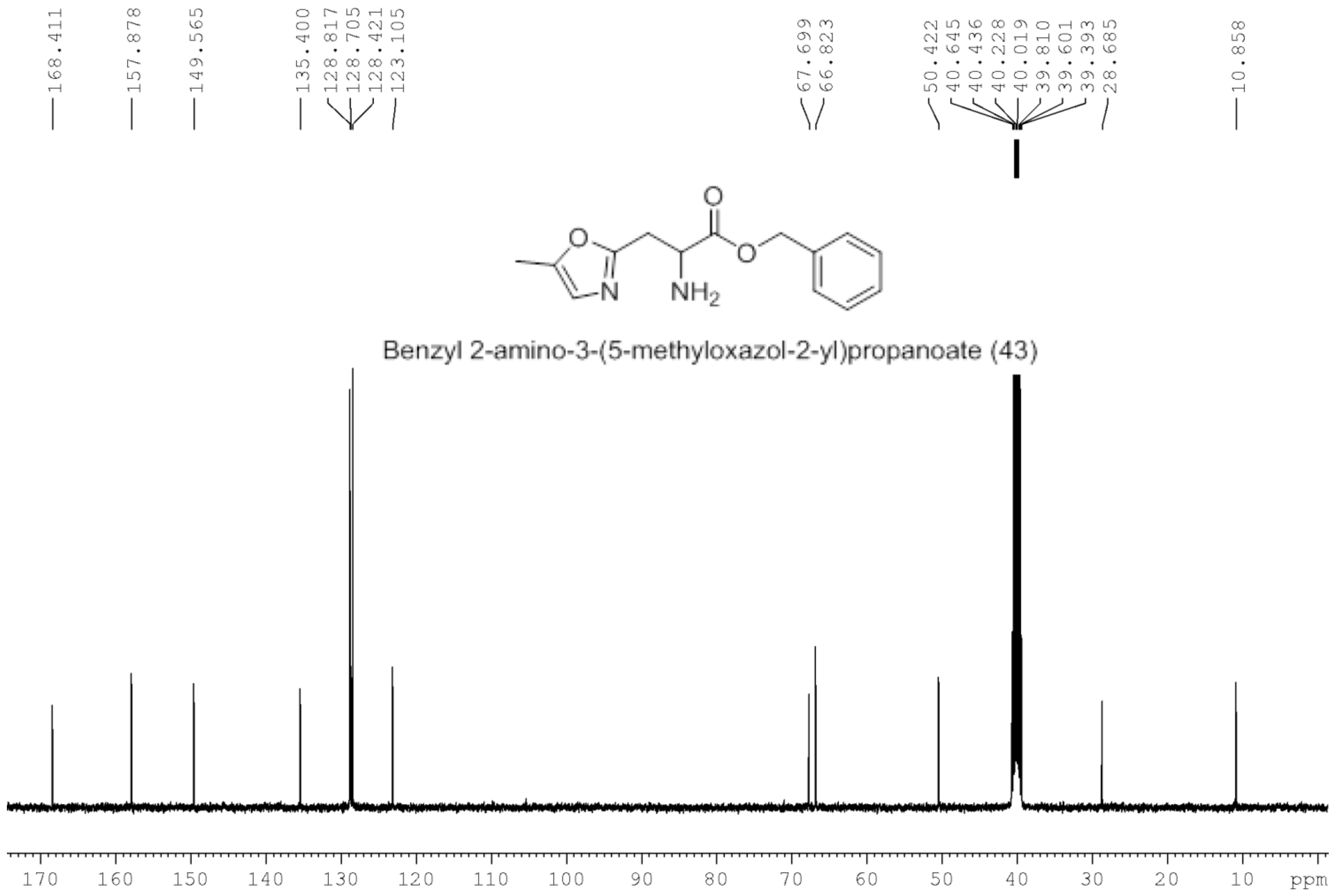

Benzyl 2-amino-3-(5-methyloxazol-2-yl)propanoate (43)

References

1. Fioravanti, S.; Pellacani, L.; Vergari, M. C. *Org. Biomol. Chem.* **2012,** *10*, 524-528.

2. Niyazymbetov, M. E.; Evans, D. H. *J. Org. Chem.* **1993,** *58*, 779-783.

3. Xue, D.; Chen, Y. C.; Cui, X.; Wang, Q. W.; Zhu, J.; Deng, J. G. *J. Org. Chem.* **2005,** *70*, 3584-3591.

4. Harel, T.; Rozen, S. *J. Org. Chem.* **2007,** *72*, 6500-6503.

5. Griffioen, G.; Van Doorren, T.; Rojas de la Parra, V.; Marchand, A.; Allasia, S.; Kilonda, A.; Chaltin, P. Indole amide derivatives and related compounds for use in the treatment of neurodegenerative diseases. WO20100142801, publication date: 16/12/2010..

6. Cecchi, L.; De Sarlo, F.; Machetti, F. *Eur. J. Org. Chem.* **2006,** *21*, 4852-4860.

7. Leslie-Smith, M. G.; Paton, R. M.; Webb, N. *Tetrahedron Lett.* **1994,** *49*, 9251-9254.

8. Xiao, X.; Zhang, W.; Lu, X.; Deng, Y.; Jiang, H.; Zeng, W. *Adv. Synth. Catal.* **2016,** *358*, 2497-2509.

9. Hashmi, A. S. K.; Blanco Jaimes, M. C.; Schuster, A. M.; Rominger, F. *J. Org. Chem.* **2012,** *77*, 6394-6408.

10. Skinner, G. S. *J. Am. Chem. Soc.* **1924,** *46*, 731-741.
